# Identification of Novel Potential Inhibitors of Pteridine Reductase 1 in *Trypanosoma brucei* via Computational Structure-Based Approaches and in Vitro Inhibition Assays

**DOI:** 10.3390/molecules24010142

**Published:** 2019-01-01

**Authors:** Magambo Phillip Kimuda, Dustin Laming, Heinrich C. Hoppe, Özlem Tastan Bishop

**Affiliations:** 1 Research Unit in Bioinformatics (RUBi), Department of Biochemistry and Microbiology, Rhodes University, P.O. Box 94, Grahamstown 6140, South Africa; pjkanywa@gmail.com; 2 College of Veterinary Medicine, Animal Resources and Biosecurity (COVAB), Makerere University, P.O. Box 7062, Kampala 00256, Uganda; 3 Department of Biochemistry and Microbiology, Rhodes University, Grahamstown 6140, South Africa; dustinlaming89@gmail.com (D.L.); H.Hoppe@ru.ac.za (H.C.H.); 4 Centre for Chemico- and Biomedicinal Research, Rhodes University, Grahamstown 6140, South Africa

**Keywords:** Human African Trypanosomiasis, pteridine reductase 1, PTR1, DHFR, anti-folates, anti-trypanosomal agents, molecular dynamics, dynamic residue network analysis, binding free energy, isobologram assay

## Abstract

Pteridine reductase 1 (PTR1) is a trypanosomatid multifunctional enzyme that provides a mechanism for escape of dihydrofolate reductase (DHFR) inhibition. This is because PTR1 can reduce pterins and folates. Trypanosomes require folates and pterins for survival and are unable to synthesize them de novo. Currently there are no anti-folate based Human African Trypanosomiasis (HAT) chemotherapeutics in use. Thus, successful dual inhibition of *Trypanosoma brucei* dihydrofolate reductase (*Tb*DHFR) and *Trypanosoma brucei* pteridine reductase 1 (*Tb*PTR1) has implications in the exploitation of anti-folates. We carried out molecular docking of a ligand library of 5742 compounds against *Tb*PTR1 and identified 18 compounds showing promising binding modes. The protein-ligand complexes were subjected to molecular dynamics to characterize their molecular interactions and energetics, followed by in vitro testing. In this study, we identified five compounds which showed low micromolar Trypanosome growth inhibition in in vitro experiments that might be acting by inhibition of *Tb*PTR1. Compounds RUBi004, RUBi007, RUBi014, and RUBi018 displayed moderate to strong antagonism (mutual reduction in potency) when used in combination with the known *Tb*DHFR inhibitor, WR99210. This gave an indication that the compounds might inhibit both *Tb*PTR1 and *Tb*DHFR. RUBi016 showed an additive effect in the isobologram assay. Overall, our results provide a basis for scaffold optimization for further studies in the development of HAT anti-folates.

## 1. Introduction

African trypanosomes are flagellated hemo-parasites, transmitted by Tsetse flies, and cause zoonotic infection in mammalian hosts [1]. In animals the disease is known as Nagana while in humans it is known as Human African Trypanosomiasis (HAT) [2,3]. Acute HAT is caused by *Trypanosoma brucei rhodesiense* (*Tbr*) while chronic HAT is caused by *Trypanosoma brucei gambiense* (*Tbg*). This neglected tropical disease (NTD) remains of considerable public health and animal production concern [4,5].

Trypanosomes are unable to synthesize folates and pterins de novo [6]. Reduced folate and pterin cofactors are essential for parasite survival where they are critical in pathways such as protein and nucleic acid biosynthesis [7]. In order to survive, trypanosomes scavenge extracellular folate and pterin precursors from their hosts [8,9]. Hence, the pathway is an interesting drug target. Drugs targeting the folate pathway have been used in the treatment of several infections, most notably in the treatment of bacterial and malarial infections [10]. However, their use in the treatment and management of HAT has not been successful to date.

**Figure 1:**
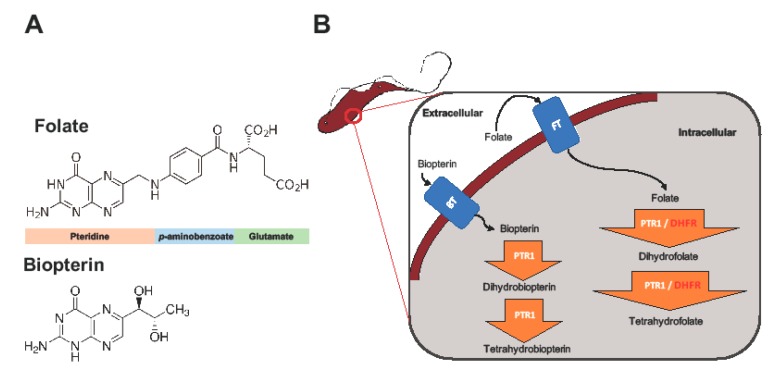
The role of dihydrofolate reductase (DHFR) and pteridine reductase 1 (PTR1) in trypanosome folate and pterin metabolism. Trypanosomes, which are auxotrophic for folates and pterins, salvage them from the host. The structures of folate and biopterin are shown (**A**). Folates and pterins are taken up by transporters (the folate-biopterin transporter superfamily includes biopterin transporter 1 (BT1) and folate transporter 1 (FT1)) after which they are reduced to their functional cofactors (**B**).

The key enzymes involved in trypanosome folate metabolism are dihydrofolate reductase (DHFR) and pteridine reductase 1 (PTR1) (Figure 1) [11,12,13]. DHFR (EC 1.5.1.3) is an NADPH-dependent enzyme that catalyzes reduction of folate to dihydrofolate (H_2_F), and H_2_F to tetrahydrofolate (H_4_F) (Figure 1) [11,12]. Folate is essentially a pteridine that has been conjugated to *p*-aminobenzoic acid (pABA) that is glutamylated (Figure 1) [14]. DHFR is a validated and primary target of most anti-folate drugs [12]. However, the use of traditional anti-folates against DHFR in trypanosomatids such as *Trypanosoma* and *Leishmania* has been largely unsuccessful [12,13,15,16].

PTR1 (EC 1.5.1.33), which is a short-chain dehydrogenase reductase family member and an NADPH-dependent enzyme, is unique to trypanosomatids [8]. It is important in the reduction of biopterin to dihydrobiopterin (H_2_B), and of H_2_B to tetrahydrobiopterin (H_4_B) (Figure 1). PTR1 also reduces folate to H_2_F, and H_2_F to H_4_F (Figure 1) [8]. In trypanosomatids, PTR1, which is less susceptible to traditional anti-folate inhibition, contributes about 10% to total folate metabolism [13]. It is important to note that studies have shown that under DHFR inhibition PTR1 is over-expressed, thus promoting anti-folate resistance in *Leishmania major* and *Trypanosoma cruzi* [8,13,15,16]. This has been proposed as the key mechanism by which trypanosomatids are able to resist anti-folates targeting DHFR [8,13,15,16]. Gene knock down and knock out studies in *T. brucei* have shown that PTR1 is essential for parasite survival. As such, its inhibition alone might be sufficient to negatively impact parasite survival [17,18].

There are several studies that have reported successful combination of PTR1 and DHFR inhibitors in order to achieve synergistic inhibition of the trypanosomatid folate pathway in *T. cruzi*, *L. major* and *T. brucei* [18,19,20,21,22]. However, the identification of a single inhibitor motif that can target both enzymes has remained largely elusive. It has been hampered by poor selectivity against human DHFR as has been the case with PTR1 inhibitors that contain functional groups derived from DHFR inhibitors, such as 2,4 diaminoquinazoline, 2,4 diaminopteridine, or 2,4 diaminopyrimidine moieties [18,20,21]. Further, the current drugs used to treat HAT are old, toxic, and reducing in efficacy due to resistance [23,24]. A recent development in African HAT chemotherapy is the promising oral drug fexinidazole that is currently in clinical testing for the treatment of late stage chronic HAT (*Tb gambiense*) [25].

In this study, we sought to identify novel *T. brucei* PTR1 (*Tb*PTR1) inhibitors that can be used in conjunction with known DHFR inhibitors or single inhibitors that target both enzymes with minimal human toxicity. Here, we performed structure-based virtual screening of 5742 small ligand molecules against *Tb*PTR1 and its orthologs from *T. cruzi* (*Tc*PTR1), *L. major* (*Lm*PTR1) and *H. sapiens* (*Hs*DHRS4). In silico docking experiments identified 18 compounds preferentially bound to the trypanosomatid PTR1s and not the human DHRS4 ortholog. These promising 18 potential hits which complexed with *Tb*PTR1 were then subjected to molecular dynamics (MD) simulations, molecular mechanics Poisson–Boltzmann surface area (MM-PBSA) binding free energy calculations, and dynamic residue network (DRN) analysis. Based on their computational binding modes, selectivity, dynamic stability during MD simulations, DRN analysis, free energy of binding, and commercial availability, 13 compounds were subsequently subjected to a blood stream form (BSF) *T. brucei* in vitro inhibition assay and an *H. sapiens* in vitro cytotoxicity assay. Five out of the 13 compounds, named RUBi004, RUBi007, RUBi014, RUBi016 and RUBi018, exhibited anti-trypanosomal activities against trypanosomes in culture with IC_50_ values of 9.6 ± 3.2 μM, 34.9 ± 17.1 μM, 14.6 ± 9.9 μM, 25.4 ± 4.7 μM, and 12.7 ± 3.7 μM, respectively. Compounds RUBi007, RUBi016, and RUBi018 showed no significant human cell cytotoxicity at 100 µM while RUBi004 and RUBi014 had cytotoxicity IC_50_s of 23.6 ± 5.8 μM and 32.9 ± 2.2 μM, respectively.

Compounds RUBi004, RUBi007, RUBi014, and RUBi018 showed reduced inhibition when used in combination with a known DHFR inhibitor (WR99210), which may be suggestive of competitive inhibition of *Tb*DHFR confounded by the upregulation of *Tb*PTR1 expression as a result of simultaneous inhibition. Compound RUBi016 showed an additive effect when used in combination with WR99210, suggesting that it preferentially inhibits *Tb*PTR1 and the addition of WR99210 further contributes to the reduction of available reduced folates, resulting in reduced parasite viability. In summary, the current study reports five compounds with inhibitory activities at low μM levels, and their scaffolds may be further optimized to design safe and effective HAT chemotherapeutics targeting the folate pathway.

## 2. Results

Please note that unless otherwise indicated, *Tb*PTR1 numbering is used throughout the article.

### 2.1. Overview of PTR1 Structure and Conservation

**Figure 2:**
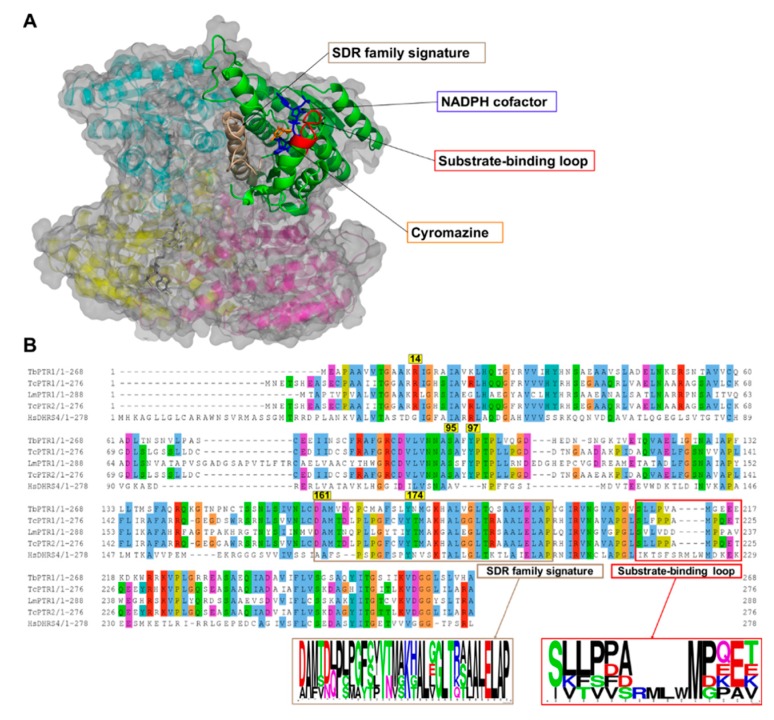
A cartoon representation of the *Trypanosoma brucei* pteridine reductase 1 (*Tb*PTR1) protein structure (PDB: 2X9N) and a multiple sequence alignment of *T. brucei* PTR1, *T. cruzi* PTR1, *T. cruzi* PTR2, *L. major* PTR1, and *H. sapiens* dehydrogenase/reductase (SDR family) member four (DHRS4). (**A**) The protein is colored by chain, with the NADPH cofactor colored blue and the co-crystallized ligand cyromazine colored orange. *Tb*PTR1 is a tetramer and the α/β single domain subunit (chain A) is shown in green. The substrate binding loop is colored red and was composed of residues SER207–GLU215, while the SDR family signature, which is colored brown, was composed of residues ASP161–ALA193. (**B**) The multiple sequence alignment (MSA) showed notable conservation in the SDR family signature, as shown by the sequence logo of the extracted motif. The MSA also showed that within the substrate binding loop there was a four residue deletion that was present only among the trypanosomatids.

PTR1 is a short-chain dehydrogenase/reductase (SDR) family member that has the ability to catalyze the NADPH-dependent two-stage reduction of biopterins to their 7,8-dihydro and 5,6,7,8-tetrahydro forms as well as folates to their H_2_F and H_4_F forms [8] (Figure 1). PTR1 is a homotetramer with four equivalent active sites and four NADPH binding sites (Figure 2A). Each single α/β-domain subunit is constructed around an NADPH binding Rossman-fold repeat that is composed of seven parallel β-sheets that are between three α-helices on either side (Figure 2A) [16].

Multiple sequence alignment (MSA) of the *Tb*PTR1, *Tc*PTR1, *Lm*PTR1, and *Hs*DHRS4 ortholog protein sequences showed conservation of several key residues within the SDR family signature (residues ASP161-ALA193) as well as in the substrate binding loop (residues SER207-GLU215) present in trypanosomatids (Figure 2B). Pterin and folate substrates, along with inhibitors, interact with PTR1 complexes quite similarly, often via binding in a π-sandwich between the NADPH nicotinamide ring and residue PHE97 [19,26]. The NADPH cofactor is known to be essential in creating both the substrate binding site as well as the catalytic center [19,27]. ARG14, SER95, PHE97, ASP161, and TYR174 are important residues that interact with the folate and pterin substrates, and are well conserved among the trypanosomatids [19,26] (Figure 2B). In *T. cruzi*, *Tc*PTR1 and *Tc*PTR2 are isoforms that show very high sequence homology but also display varied enzymatic activity [28]. *Tc*PTR1 in comparison to *Tc*PTR2 shows higher activity with biopterin and folate than with H_2_F or H_2_B [28]. *Tc*PTR1 has no crystal structure, so for this study a structure was calculated using homology modeling with *Tc*PTR2 as the template [29].

The whole protein structural superimposition of *Tb*PTR1 (Protein Data Bank (PDB): 2X9N) [26] with *T. cruzi* PTR2 (PDB: 1MXH) [28], *Lm*PTR1 (PDB: 1E92) [16], and *H. sapiens* DHRS4 (PDB: 304R) gave root mean square deviation (RMSD) values of 0.4, 0.5, and 1.6, indicating that the trypanosomatid PTR1s are structurally very similar.

### 2.2. Eighteen Potential Hits Out of 5742 Compounds are Identified via Virtual Screening

*Tb*PTR1 (PDB: 2X9N) [26], *Lm*PTR1 (PDB: 1E92) [16], *Hs*DHRS4 (PDB: 304R), and a homology model of *Tc*PTR1 were used in in silico virtual screening investigations. All structures included the NADPH cofactor, which is essential for arrangement of the substrate binding site and catalytic center. 5742 compounds were docked against four proteins using Autodock Vina, as is described in the Materials and Methods section, in order to identify potential hits. Top compounds were selected based on their Autodock Vina energy score of <−8.0 kcal/mol and their hydrogen bonding profiles. Eighteen compounds showed good selectivity for trypanosomatid PTR1 as presented in Table 1. Out of 18 compounds, only RUBi006 bound to the *Hs*DHRS4 active site but with a weaker binding energy than the trypanosomatids. The docked complexes were analyzed using PyMOL [30] and Discovery Studio [31]. The docking energy scores were also evaluated using Xscore [32]. A summary of the compounds with the top binding modes and their corresponding energies are shown in Table S1. The top binding modes involved ligand interactions with residues that are known to be of catalytic importance, i.e., ARG14, SER95, PHE97, ASP161, and TYR174 (Table S1) [16,19]. Residues ARG14, SER95, PHE97, and ASP161 were conserved among all the trypanosomatids, while TYR174 was conserved in all the PTR1 orthologs (Figure 2). Furthermore, residues ASP161 and TYR174 are located within the SDR family signature, which is important in the NADPH cofactor and substrate binding (Figure 2). The IUPAC names of the ligands are shown in Table S2.

### 2.3. Drug Likeness

**Figure 3:**
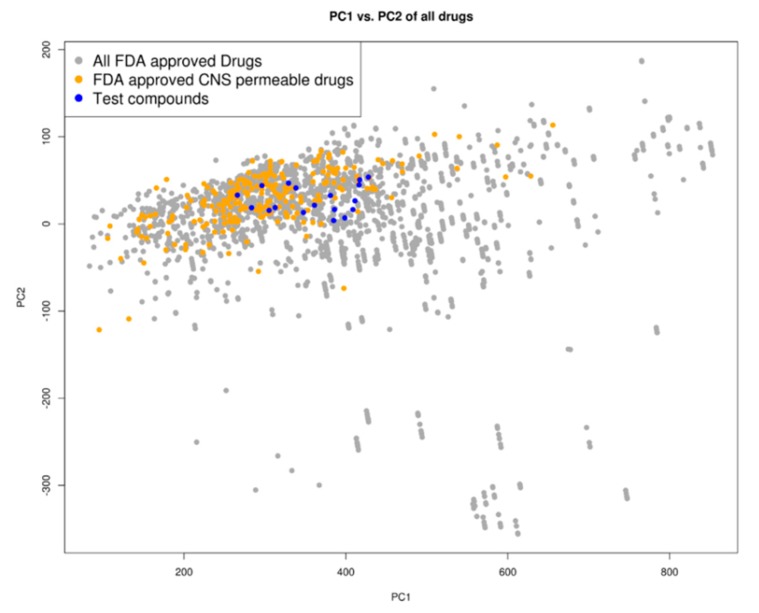
Chemical space distribution of the test compounds (*n* = 18), Food and Drug Administration (FDA)-approved drugs (*n* = 3180), and FDA-approved central nervous system (CNS)-permeable drugs (*n* = 221). The principal component analysis (PCA) of the molecular descriptors showed that the test compounds clustered well with FDA-approved CNS-permeable drugs. The PCA scatter plot was based on the first (PC1) and second (PC2) components as shown on the two axes. PC1 explained 76% of the variance while PC2 explained 18%. All FDA-approved drugs are shown as grey dots, while FDA-approved CNS-permeable drugs are shown as yellow dots and the test compounds are shown as blue dots.

A principal component analysis (PCA) of the compounds based on their molecular descriptors, as is listed in the Materials and Methods section, showed that the top docking compounds clustered well with known Food and Drug Administration (FDA)-approved central nervous system (CNS)-permeable drugs (Figure 3) occupying the same chemical space as FDA-approved drugs as well as FDA-approved CNS-permeable drugs. The compounds are of ‘drug-like’ desirability and are likely to cross the blood-brain barrier (BBB), making them good candidates for HAT chemotherapeutics [33].

### 2.4. Five Hit Compounds Show Anti-Trypanosomal Activity In Vitro

**Figure 4:**
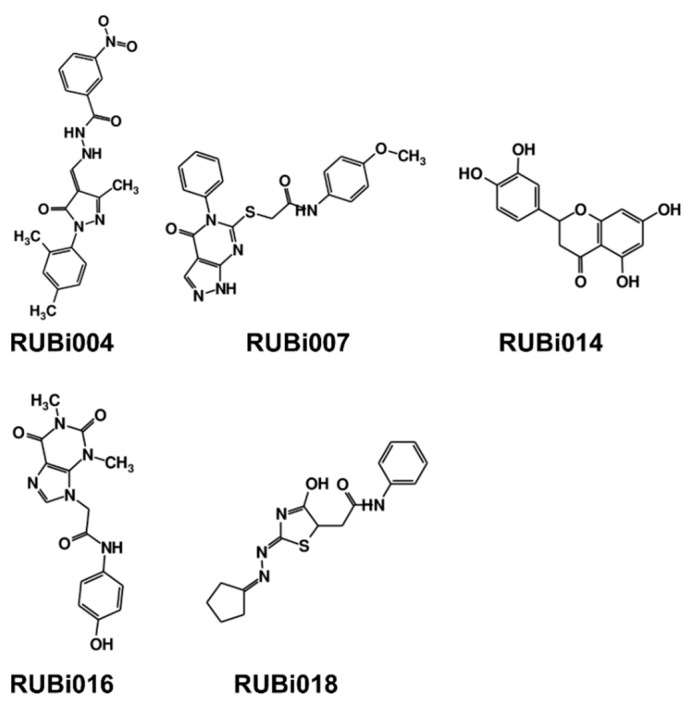
Structures of the five *Tb*PTR1 hit compounds.

**Figure 5:**
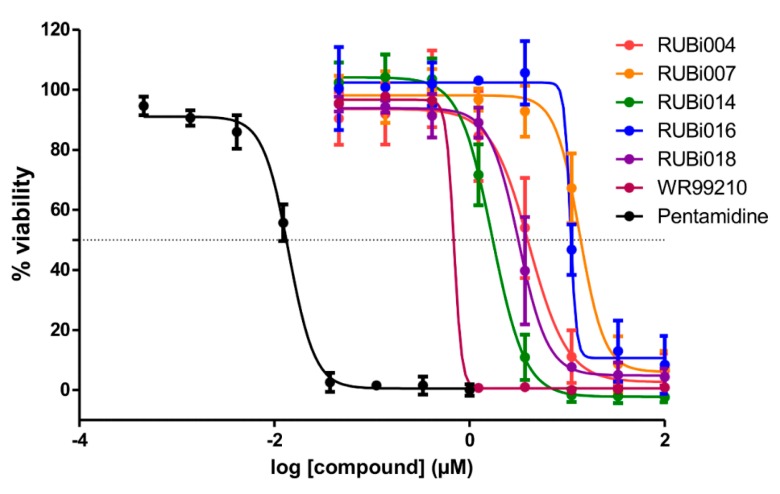
Dose-response plots to derive IC_50_ values for RUBi004 (red), RUBi014 (orange), RUBi007 (green), RUBi016 (blue), RUBi018 (purple), WR99210 (maroon), and pentamidine (black). Pentamidine was used as the positive control anti-trypanosomal compound. Parasite % viability was determined using the resazurin method. Error bars indicate the standard deviation of % viability obtained in three replicate wells. The above graph represents one of three independent experiments that were performed on separate occasions. The combined IC_50_ values (± standard deviation) of RUBi004, RUBi007, RUBi014, RUBi016, and RUBi018 were determined to be 9.6 ± 3.2 μM, 34.9 ± 17.1 μM, 14.6 ± 9.9 μM, 25.4 ± 4.7 μM, and 12.7 ± 3.7 μM, respectively. The IC_50_ values of WR99210 and pentamidine were determined to be 0.5 ± 0.4 µM and 0.014 µM, respectively.

As a next step, a total of 18 *Tb*PTR1-ligand complexes were subjected to 200 ns all-atom MD simulations followed by MM-PBSA free energy calculations. All the compounds showed linear stable MD trajectories as observed in RMSD (Figures S1–S7), radius of gyration (Rg) calculations (Figures S1–S6 and S8), and promising hydrogen bonding features (Figures S1–S6 and S9). Root mean square fluctuation (RMSF) values (Figures S1–S6 and S10) and binding free energies (Figure S11) were also calculated. PCA was also carried out to investigate the overall dynamics of the protein systems [34]. Overall, in silico analysis indicated that all 18 compounds could be further studied for in vitro analysis. However, only 13 of these were commercially available and tested for anti-trypanosomal activity against *T. brucei* BSF in culture. Compounds RUBi003, RUBi006, RUBi009, RUBi013, and RUBi017 could not be purchased, and as such were not used in the in vitro inhibition assays, even though they showed similar binding modes to folate, pterins, and known *Tb*PTR1 inhibitors. In spite of good binding modes and stable MD trajectories, compounds RUBi001, RUBi002, RUBi005, RUBi008, RUBi010, RUBi011, and RUBi012 did not show anti-trypanosomal activity in in vitro experiments when used at 20 µM. The five compounds shown in Figure 4 that showed significant inhibition of *T. brucei* viability at this concentration were subjected to dose-response assays to derive their IC_50_ values against *T. brucei* (Figure 5), and are discussed further below.

#### 2.4.1. *Tb*PTR1 Hit Compounds Have Either Antagonist or Additive Activity When Used in Combination with a *Tb*DHFR Inhibitor

**Figure 6:**
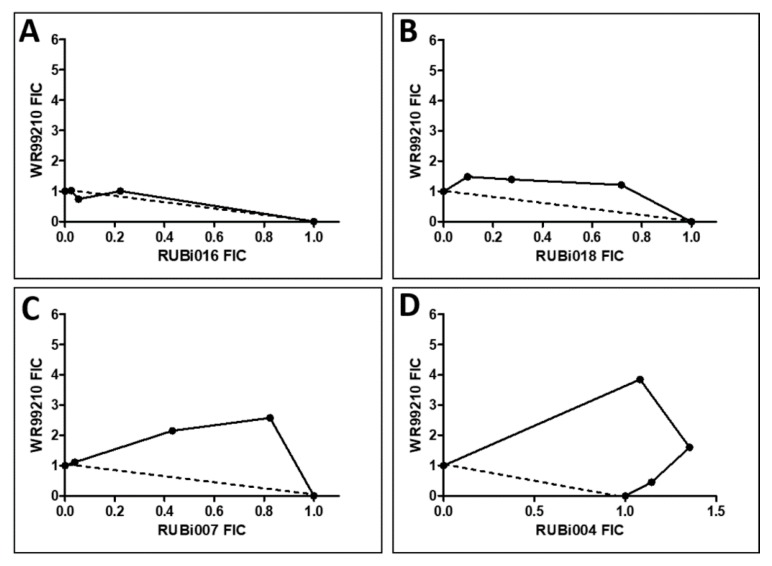

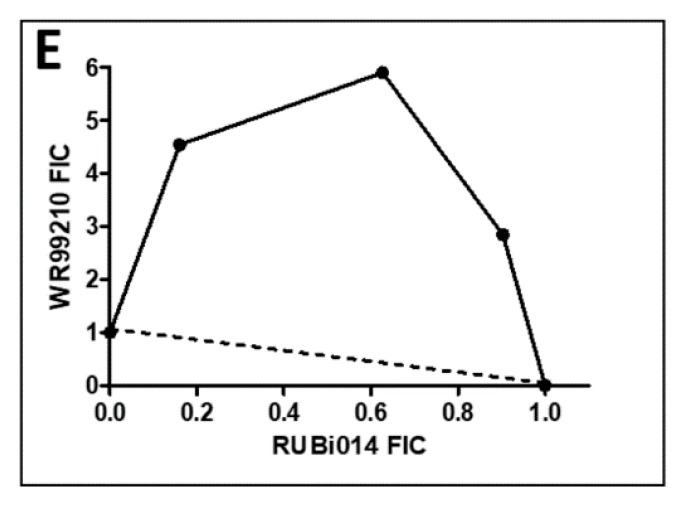
Isobologram analysis of compounds RUBi016 (**A**), RUBi018 (**B**), RUBi007 (**C**), RUBi004 (**D**) and RUBi014 (**E**) in combination with WR99210. RUBi compounds and WR99210 were employed alone at starting concentrations of 100 µM and 20 µM, respectively, and in combination ratios of 75:25, 50:50, and 25:75. IC_50_ values obtained for the RUBi compounds and WR99210 alone and in combination were used to calculate and plot their fractional inhibitory concentrations (FIC). The dotted line in the graphs denotes an additive effect; FIC values above the line indicate compound antagonism, while values below the line indicate synergism.

RUBi016 inhibited blood stream form trypanosome growth in culture with an IC_50_ value of 25.4 ± 4.7 μM (Figure 5) and it was the only compound which showed an additive effect when used in combination with WR99210 (Figure 6A). RUBi004, RUBi007, RUBi014, and RUBi018 inhibited BSF trypanosome growth in culture with IC_50_ values of 9.6 ± 3.2 μM, 34.9 ± 17.1 μM, 14.6 ± 9.9 μM, and 12.7 ± 3.7 μM, respectively (Figure 5). When used in combination with the known DHFR inhibitor WR99210, these compounds showed antagonism, with RUBi018 to the least and RUBi014 to the highest extent (Figure 6).

**Figure 7:**
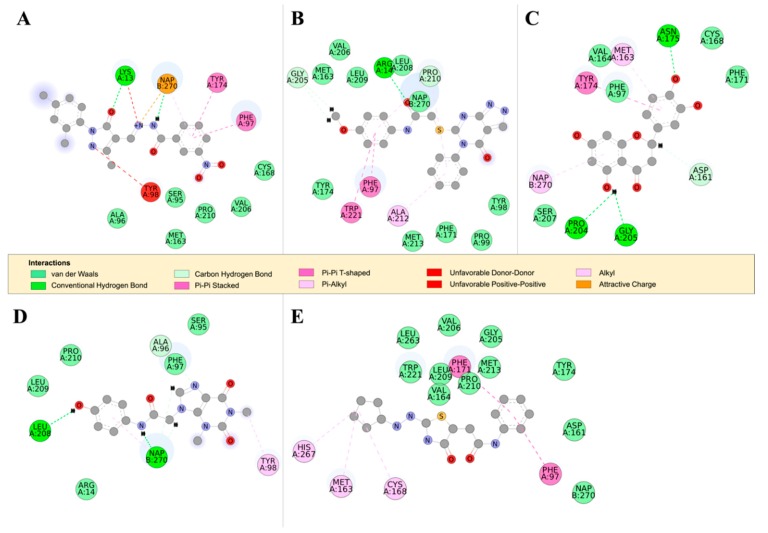
The 2D structures of RUBi004, RUBi007, RUBi014, RUBi016, and RUBi018, along with their binding modes in the *Tb*PTR1 protein. (**A**) A 2D representation of RUBi004 and its binding mode with *Tb*PTR1; (**B**) a 2D representation of RUBi007 and its binding mode with *Tb*PTR1; (**C**) a 2D representation of RUBi014 and its binding mode with *Tb*PTR1; (**D**) a 2D representation of RUBi016 and its binding mode with *Tb*PTR1; and (**E**) a 2D representation of RUBi018 and its binding mode with *Tb*PTR1.

**Figure 8:**
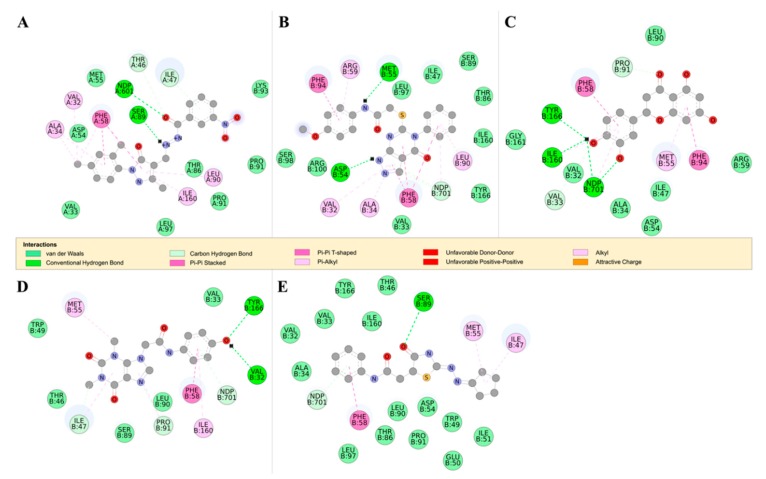
The 2D structures of RUBi004, RUBi007, RUBi014, RUBi016, and RUBi018, along with their binding modes in the *Tb*DHFR protein. (**A**) A 2D representation of RUBi004 and its binding mode with *Tb*DHFR; (**B**) a 2D representation of RUBi007 and its binding mode with *Tb*DHFR; (**C**) a 2D representation of RUBi014 and its binding mode with *Tb*DHFR; (**D**) a 2D representation of RUBi016 and its binding mode with *Tb*DHFR; and (**E**) a 2D representation of RUBi018 and its binding mode with *Tb*DHFR.

*Tb*PTR1 and *Tb*DHFR both catalyze the reduction of folate to H_2_F and H_4_F; the substrate binding sites, however, are differently ordered [35]. Additionally, *Tb*DHFR is capable of undergoing significant conformational changes when in complex with thymidylate synthase (TS), while *Tb*PTR1 is more rigid [36,37,38]. All the compounds appear to bind in similar patterns to pterin, folate, and known *Tb*PTR1 inhibitors interacting with the NADPH cofactor and the *Tb*PTR1 protein [19] (Figure 7). As a next step, all the hit compounds were docked against *Tb*DHFR active sites to see potential binding activity. RUBi004 (Figure 8A), RUBi007 (Figure 8B), RUBi014 (Figure 8C), and RUBi018 (Figure 8E) molecular *Tb*DHFR dockings showed high binding affinity towards the protein, with −9.4 kcal/mol, −9.5 kcal/mol, −8.7 kcal/mol, and −7.9 kcal/mol, respectively. RUBi016 bound to *Tb*DHFR with a binding affinity of −7.6 kcal/mol (Figure 8D). Interestingly, *Tb*DHFR compound binding energy scores were in agreement with isobologram results. Three compounds (RUBi004, RUBi007, and RUBi014) with the greatest antagonist activities also had the highest binding energies towards *Tb*DHFR. The possibility that RUBi004, RUBi007, and RUBi014 also inhibit DHFR may explain why they show more marked antagonism when used in combination with DHFR inhibitor; WR99210 and each of these three compounds both inhibit *Tb*DHFR, thus possibly enhancing the upregulation of *Tb*PTR1.

Compound RUBi018, with the least antagonist activity, had a similar *Tb*DHFR binding energy to RUBi016 that showed an additive effect with WR99210. RUBi018 also displayed the highest binding energy for *Tb*PTR1 among all five compounds (−8.4 kcal/mol; Table 1). Interestingly, with the exception of RUBi018 which was more active than predicted, the anti-trypanosomal IC_50_ values of the remaining four compounds correlated with their *Tb*PTR1 binding energies. RUBi004, with the highest affinity (−10.4 kcal/mol), displayed the most potent activity (with an IC_50_ of 9.6 ± 3.2 µM).

The use of *Tb*PTR1 inhibitors in combination with *Tb*DHFR inhibitors has long been proposed as a viable avenue for the generation of new anti-trypanosomal anti-folate drugs [19]. It is important to note, however, that studies have shown that under DHFR inhibition, PTR1 is over-expressed, thus promoting anti-folate resistance in *Leishmania major* and *Trypanosoma cruzi* [8,13,15,16]. This likely contributes to the antagonism of the RUBi compounds’ activity by WR99210, an effect that might be exacerbated by the additional possible inhibition of *Tb*DHFR by RUBi004, RUBi007, RUBi014, and RUBi018.

#### 2.4.2. Detailed in Silico Compound Analysis

Overall, our in silico rational-based drug discovery approach was able to identify five compounds that showed anti-trypanosomal in vitro activity. Compounds RUBi007, RUBi016, and RUBi018 showed no significant human cell cytotoxicity at 100 µM, while RUBi004 and RUBi014 had cytotoxicity IC_50_s of 23.6 ± 5.8 μM and 32.9 ± 2.2 μM, respectively (Figure S12). RUBi014, also known as eriodictyol, is a flavonoid that has previously been reported to be selectively anti-protozoal with activity against *T. brucei* in culture [39,40]. RUBi018 is related to 4-aryl-2-(1-substituted ethylidene) thiazoles that have been previously reported to show anti-bacterial activity [41]. To our knowledge, there has been no specific activity reported in the literature for the rest of the compounds.

The detailed in silico analysis for each compound is presented below.

**TbPTR1 ligand docking analysis**: Docking analysis of the *Tb*PTR1-ligand complexes showed that RUBi004 formed a π-sandwich between the NADPH nicotinamide ring and PHE97 (Figure 7A). It also formed a T-shaped π-π interaction with TYR174 and two hydrogen bonds with LYS13 and NADPH (Figure 7A). RUBi004 formed van der Waals (vdW) interactions with SER95, ALA96, CYS168, VAL206, and PRO210 (Figure 7A). Unfavorable interactions (colored in red) included positive-positive interactions between LYS13 NZ and RUBi004 N2, along with a donor-donor interaction between TYR98 HH and RUBi004 N1 (Figure 7A). RUBi007 formed T-shaped π-π interactions with PHE97 and TRP221. It also formed π-alkyl interactions with PRO210 and ALA212. It formed hydrogen bonds with ARG14 and GLY205 (Figure 7B). RUBi007 formed vdW interactions with TYR98, PRO99, MET163, PHE171, TY174, GLY205, VAL206, LEU208, LEU209, PRO210, MET213, and the NADPH cofactor (Figure 7B). RUBi014 formed a T-shaped π-π interaction with TYR174, π-alkyl interactions with MET163, and a π-alkyl interaction with the NADPH nicotinamide ring (Figure 7C). It also formed hydrogen bonds with ASP161, ASN175, PRO204, and GLY205 (Figure 7C). RUBi014 also formed vdW interactions with ASP161, VAL164, CYS168, PHE171, and SER207 (Figure 7C). RUBi016 formed a π-alkyl interaction with the NADPH nicotinamide ring and TYR98. It formed hydrogen bonds with ALA96 and LEU208 (Figure 7D). RUBi016 formed vdW interactions with ARG14, SER95, PHE97, LEU209, and PRO210 (Figure 7D). RUBi016 appears to be the only ligand that formed a hydrogen bond with the NADPH cofactor (Figure 7D). Lastly, RUBi018 formed T-shaped π-π interactions with PHE97 and PHE171. RUBi018 also formed alkyl and π-alkyl interactions with MET163, CYS168, and HIS267 (Figure 7E). RUBi018 formed vdW interactions with ASP161, VAL164, TYR174, GLY205, VAL206, PRO210, MET213, TRP221, LEU263, and the NADPH cofactor (Figure 7E).

**TbDHFR ligand docking analysis**: This docking analysis showed that the compounds bound to *Tb*DHFR in similar binding modes to known *Tb*DHFR inhibitors [42]. We observed that all the ligands in the *Tb*DHFR-complexes formed π-π interactions with residue PHE58 that was involved in both WR99210 and pyrimethamine inhibitor binding (Figure 8) [42]. *Tb*DHFR residues ALA34, VAL32, MET55, PHE58, SER89, PHE94, TYR166, and the NADPH cofactor are known to be involved in the binding of *Tb*DHFR inhibitors such as pyrimethamine and WR99210 [42]. RUBi004 formed hydrogen bonds with the NADPH cofactor and residues SER89, THR46, and ILE47 (Figure 8A). RUBi004 also formed π-π interactions with residues VAL32, ALA34, THR46, PHE58, LEU90, PRO91, and ILE169 (Figure 8A). The RUBi007 complex formed hydrogen bonds with the NADPH cofactor, THR46, ILE47, and SER89, and formedπ-π interactions with VAL32, ALA34, PHE58, LEU90, and ILE160 (Figure 8B). RUBi014 formed hydrogen bonds with the NADPH cofactor and VAL33, PRO91, ILE160, and TYR166 (Figure 8C). It also formed π-π interactions with PHE58, MET55, and PHE94 (Figure 8C). RUBi016 formed hydrogen bonds with the NADPH cofactor, TYR166, and VAL32, and π-π interactions with PHE58 and ILE160 (Figure 8D). RUBi018 formed hydrogen bonds with the NADPH cofactor and SER89, and π-π interactions with PHE58 in a similar binding mode to known *Tb*DHFR inhibitors [42] (Figure 8E).

**Molecular dynamics**: The RMSD calculations revealed that the protein backbone and the NADPH cofactors in the *Tb*PTR1-ligand complexes showed conformational changes relative to their initial structures, deviating by 0.3 nm–0.5 nm (Figures S1a, S2a, S3a, S4a, S6a and S7a). Rg analysis showed that the binding of the ligand led to increased compactness in *Tb*PTR1-RUBi004 and *Tb*PTR1-RUBi007 (Figures S2b and S3b). In the *Tb*PTR1-RUBi004 complex we observed slight increases in the flexibility of residues LYS13, PHE97, TYR98, and TYR174 via RMSF calculations (Figure S2d). Loop residues MET169 and ALA170 showed increased flexibility, while helix residues ALA188 and ALA189 showed reduced flexibility (Figure S2d). The substrate binding loop SER207–GLU217 was stable (Figure S2d). In the *Tb*PTR1-RUBi007 complex we observed decreased flexibility in ARG14, PHE97, GLY205, PRO210, ALA212, and TRP221 (Figure S3d), and increased flexibility in GLY247, SER248, and ALA249 (Figure S3d). In the *Tb*PTR1-RUBi014 and *Tb*PTR1-RUBi016 complexes we observed a significant increase in the flexibility of the substrate binding loop and the small α6 helix (Figures S4d and S5d). Lastly, in the *Tb*PTR1-RUBi018 complex there was also an increase in the flexibility of the substrate binding loop (PRO210–MET213), but to a lesser extent (Figure S6d).

**Figure 9:**
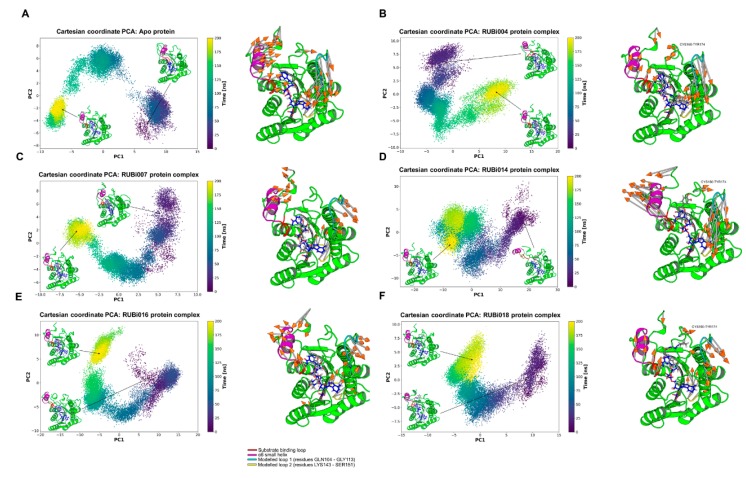
Principal component analysis of the *Tb*PTR1 apo protein and *Tb*PTR1-ligand complexes. The motion of the protein during 200 ns of the all-atom MD simulation is shown along the first and second principal components (PC1 and PC2). The substrate binding loop (residues SER207–GLU215) is colored red, the α6 alpha helix (residues GLY214–VAL225) is colored magenta, the modelled missing residues loop 1 is colored cyan, the modelled missing residues loop 2 is colored yellow, and the NADPH cofactor is colored blue. (**A**) Apo protein, (**B**) *Tb*PTR1-RUBi004 complex, (**C**) *Tb*PTR1-RUBi007 complex, (**D**) *Tb*PTR1-RUBi014 complex, (**E**) *Tb*PTR1-RUBi016 complex, and (**F**) *Tb*PTR1-RUBi018 complex. For each of these, on the left is the projection of the protein-ligand complex dynamics along the PC1 and PC2, and on the right are the differential motions described by PC1 and PC2, shown by light gray arrows with orange tips.

**PCA analysis**: From the PCA we observed differences in motion between the apo *Tb*PTR1 and the ligand-bound proteins (Figure 9). In both the *Tb*PTR1-RUBi004 and apo protein systems the largest motions were in the substrate binding loop (residues SER207–GLU215), the α6 helix (residues GLY214–VAL225), the CYS160–TY174 loop region, the C-terminal residues HIS267–ALA268, the modelled loop 1 (residues GLN104–GLY113), and the modelled loop 2 (residues LYS143–SER151) (Figure 9A,B). The modelled missing residues in modelled loop 1 and modelled loop 2 showed high variability in motion (Figure 9).

In the apo protein PC1 explained 54% of the variance while PC2 covered 18%. The protein-ligand complexes had the following PC1 and PC2 variances, respectively; *Tb*PTR1-RUBi004: 46% and 26%, *Tb*PTR1-RUBi007: 40% and 21%, *Tb*PTR1-RUBi014: 66% and 12%, *Tb*PTR1-RUBi016: 57% and 20%, and *Tb*PTR1: 44% and 21%.

There were no large motions observed in the substrate binding loop in the *Tb*PTR1-RUBi007 complex and the largest motions were in the α6 helix, the C-terminal residues HIS267–ALA268, the modelled loop 1, and the modelled loop 2 (Figure 9C).

The *Tb*PTR1-RUBi014 and *Tb*PTR1-RUBi016 complexes showed very strong alteration of the motion of the substrate binding loop, the α6 helix, and the C-terminal residues HIS267–ALA268, as shown by the RMSF analysis and PCA (Figure 9D,E, Figures S4d and S5d). They appear to make the active site widen and extend (Figure 9D,E) when compared to the apo protein (Figure 9A).

Lastly, PCA showed that the *Tb*PTR1-RUBi018 complex had the largest motions in the substrate binding loop, the α6 helix, the CYS160–TY174 loop region, the C-terminal residues ALA268, the modelled loop 1, and the modelled loop 2 (residues LYS143–SER151) (Figure 9F).

**Hydrogen bond analysis**: RUBi004 formed two hydrogen bonds at a frequency of 0.02 during 200 ns of the MD simulation with *Tb*PTR1 residue LYS13 and the NADPH cofactor (Figure 7A and Figure S2c). RUBi007 formed two hydrogen bonds at a frequency of 0.27 with the protein residues ARG14 and GLY205 (Figure 7B and Figure S3c). RUBi014 formed an average of four hydrogen bonds at a frequency of 0.002 with the protein residues ASP161, ASN175, PRO204, and GLY205 (Figure 7C and Figure S4c). RUBi016 formed three hydrogen bonds at a frequency of 0.12 with the protein during the MD simulation, and these were with ALA96, LEU208, and the NADPH cofactor (Figure 7D and Figure S5c) while RUBi018 formed hydrogen bonds with GLY205 and TRP221 at a frequency of 0.07 (Figure 7E and Figure S6c).

**Binding free energy**: All five compounds bound stably to the protein throughout the MD simulation. A summary of the free binding energies of all compounds in the study is shown in Table 2. RUBi004, RUBi007, RUBi014, RUBi016, and RUBi018 bound to *Tb*PTR1 with free binding energies of −63 ± 14 kJ/mol, −88 ± 10 kJ/mol, −56 ± 12 kJ/mol, −23 ± 10 kJ/mol, and −88 ± 12 kJ/mol, respectively (Table 2).

**Figure 10:**
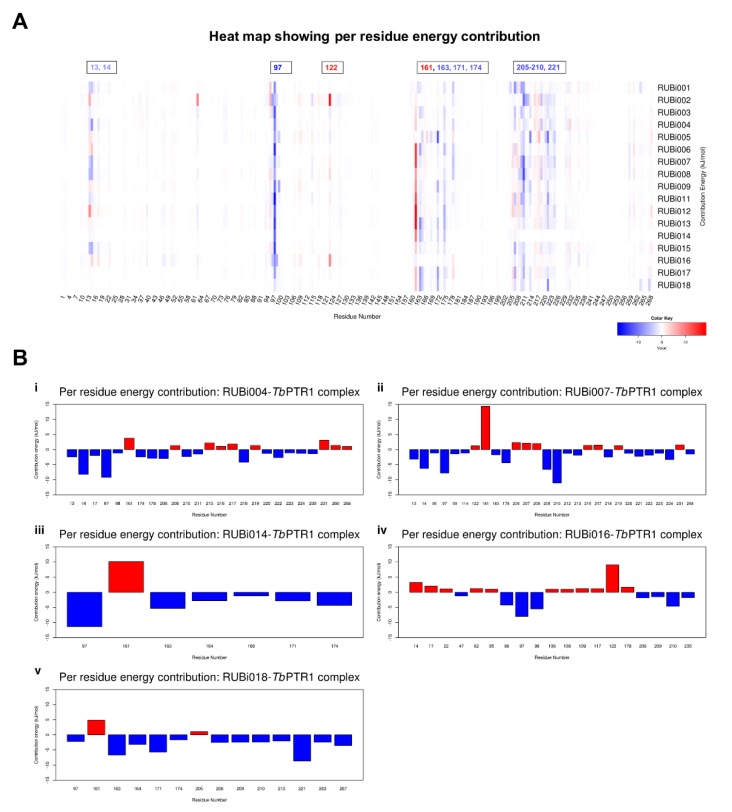
A heat map and histograms showing the per residue energy contributions to binding (with energy in kJ/mol). The compounds bound in similar conformations to known pterin, folates, and PTR1 inhibitors, as is shown by the common contributing residues to binding. (**A**) A heat map showing the per residue energy contributions for all the protein-ligand systems and (**B**) a histogram showing the main residues contributing energetically to binding in (**i**) *Tb*PTR1-RUBi004, (**ii**) *Tb*PTR1-RUBi007, (**iii**) *Tb*PTR1-RUBi014, (**iv**) *Tb*PTR1-RUBi016, and (**v**) *Tb*PTR1-RUBi018 complexes.

The per residue energy contribution offers an interesting insight into the study compounds (Figure 10). Many of the protein-ligand complexes had residues contributing to binding that were of catalytic importance and involved in the binding of known *Tb*PTR1 inhibitors (Figure 10) [16,19,26,27]. Residues LYS13, ARG14, PHE97, MET163, TYR174, and substrate binding residues LEU209–TRP221 appear to enhance ligand interactions as they contribute the most favorably energetically to ligand binding (Figure 10A). RUBi004 and RUBi007 showed NADPH cofactor binding residues LYS13 and ARG14 contributing favorably to binding (Figure 10(Bi,ii)). Active site residue ASP161 generally had poor interactions with the ligands where it gave unfavorable energy contributions to ligand binding (Figure 10). ASP161 forms hydrogen bonds with MET163 and TYR174 and is important in proton transfer to the substrate during catalysis [35]. Notably in RUBi016 where there wasn’t any interaction with residues MET163 or TYR174 as shown in the docking analysis, ASP161 did not give an unfavorable energy contribution (Figure 7D and Figure 10(Biv)). This work provides insight into important *Tb*PTR1 protein-ligand interactions that can be used in rational-based drug design to characterize potential inhibitors with the end goal of designing and optimizing HAT anti-folate drugs.

**Dynamic residue network analysis**: Average shortest path (*Average L*) and average betweenness centrality (*Average BC*) metrics over a MD trajectory [43] were calculated for comparative DRN analysis between ligand-bound and unbound *Tb*PTR1. These metrics were also compared to RMSF data (Figure S13a). As shown previously by Penkler et al., a general trend between *Average BC*, *Average L*^−1^ and RMSF^−1^ has been observed [44,45]. Pearson correlation coefficient values are presented in Table S3. Overall, only very slight changes, especially in the substrate binding loop (SER207–GLU215) and the small α6 helix (GLY214–VAL225), were observed for *Average L* between the apo protein and the *Tb*PTR1-ligand complexes (Figures S1e, S2e, S3e, S4e, S5e, and S6e).

*Average BC* is an important metric used to identify residues critical for communication flow within the protein network [43]. Residues THR9, SER95, and ALA238 showed the highest *Average BC* in the *Tb*PTR1 apo protein (Figure S13a). In the *Tb*PTR1-RUBi004 complex VAL164, SER172, and SER207 showed increases in *Average BC* compared to the apo protein (Figure S2f). In comparison to the apo protein, in the *Tb*PTR1-RUBi007 complex residues ILE15, MET163, SER207, LEU208, and PRO210 showed increases in *Average BC* (Figure S3f). In the *Tb*PTR1-RUBi014 complex, residues CYS160, GLY205, PRO210, and SER233 showed increases in *Average BC* (Figure S4f). While residues SER95 and PHE97 became less central, CYS160, GLY205, PRO210, and SER264 became more central in the *Tb*PTR1-RUBi016 complex (Figure S5f and Table S1). In the *Tb*PTR1-RUBi018 complex GLY16, ASP165, VAL206, LEU208, PRO210, and ALA232 showed increases in *Average BC* (Figure S6f).

Residues THR9, GLY205, and SER207 were conserved among all the PTR1 orthologs while SER95, CYS160, MET163, ASP165, LEU208, PRO210, and SER233 were conserved among the trypanosomatid PTR1 orthologs only (Figure 2). ILE15 and ALA238 were conserved in all the PTR1 orthologs except *Lm*PTR1, where they were replaced by a leucine and serine residue, respectively (Figure 2). Residue VAL206 was only present in *Tb*PTR1 while the other PTR1 orthologs had a leucine instead (Figure 2).

**BC-related residue interaction analysis**: A close examination of the residues THR9, SER95, and ALA238, which were shown to have high *Average BC* values in the *Tb*PTR1 apo protein, indicated that they are functionally important (Figure S1e). Analysis of their residue interaction networks in the apo protein showed that residue THR9 formed vdW interactions with ALA11, ASN92, and ALA94, and hydrogen bonds with VAL91 and ASN93 (Figure S14(1a)). SER95 formed hydrogen bonds with ALA96, ASN127, and the NADPH cofactor (Figure S14(1b)). Furthermore, SER95 formed vdW interactions with TYR174 and PHE97, both of which are involved in ligand binding (Figure 7). Lastly, ALA238 formed an alkyl interaction with ALA18 that is hydrogen bonded to ILE15 (Figure S14(1c)). These interactions with functionally important residues indicate that *Average BC* is helpful in identifying residues crucial to communication flow within the protein-ligand dynamic network.

In addition, the binding of ligands appears to alter the flow of information across the protein network. In the *Tb*PTR1-RUBi004 complex, residues VAL164, SER172, and SER207, all of which were close to residues involved in ligand and NADPH cofactor binding, showed changes in *Average BC* (Figures S2f and S14(2a)). SER207 formed a hydrogen bond with the NADPH cofactor, while RUBi004 formed vdW interactions with residues MET163, TYR174 and VAL206 (Figure 7A and Figure S14(2c)).

In the *Tb*PTR1-RUBi007 complex, residue IL15 formed vdW interactions with the NADPH cofactor while MET163 formed vdW interactions with the RUBi007 ligand (Figure S14(3a,b)). Residues SER207 and LEU208 each formed a hydrogen bond with the NADPH cofactor (Figure S14(3c,d)). Residue LEU208 also had vdW interactions with the RUBi007 ligand and formed a hydrogen bond with ARG14 (Figure S14(3)). Residue PRO210 had a π-alkyl interaction with PHE97, had vdW interactions with ARG14, and formed a hydrogen bond with the RUBi007 ligand (Figure S14(3e)).

In the *Tb*PTR1-RUBi014 complex, residue CYS160 was covalently bound to ASP161 and had vdW interactions with PRO204 (Figure S14(4a)). Residue PRO205 formed a hydrogen bond with RUBi014 and was covalently bonded to residue PRO204 (Figure S14(4b)). Residue PRO210 formed vdW interactions with LEU208 and VAL211 (Figure S14(4c)). Lastly, residue SER233 had vdW interactions with SER207 (Figure S14(4d)). *Tb*PTR1 residues ASP161, PRO204, and GLY205 were shown to be important in RUBi014 binding (Figure 7C), while residues SER207, LEU208, and VAL211 were located in the substrate binding loop (Figure 2).

In the *Tb*PTR1-RUBi016 complex, SER95 formed vdW interactions with the RUBi016 ligand, ALA96, and the NADPH cofactor. Residue CYS160 formed vdW interactions with PRO204 and had alkyl interactions with ALA203 (Figure S14(5b)). Residue GLY205 formed vdW interactions with the NADPH cofactor, ALA203, PRO204, VAL206, and SER264 (Figure S14(5c)). Residue PRO210 formed vdW interactions with the ligand, while SER264 formed vdW interactions with GLY205 and VAL206 (Figure S14(5d)).

Lastly, in the *Tb*PTR1-RUBi018 complex, ASP165 had alkyl interactions with the RUBi018 ligand and formed vdW interactions with MET163 (Figure S14(6b)). Residue VAL206 formed vdW interactions with both the RUBi018 ligand and the NADPH cofactor (Figure S14(6c)). Residue LEU208 had a hydrogen bond with the NADPH cofactor and formed vdW interactions with PRO210 (Figure S14(6d)). Residue PRO210 formed vdW interactions with the RUBi018 ligand and PHE97 (Figure S14(6e)), while residue ALA232 formed a hydrogen bond with VAL206 and SER207 (Figure S14(6f)).

These interactions with functionally important residues indicate that *Average BC* is helpful in identifying residues crucial to communication flow within the protein-ligand dynamic network.

## 3. Discussion

In this study, structure-based molecular docking was used to screen 5742 selected compounds against trypanosomatid PTR1s and a human homolog (*Hs*DHRS4) to identify potential hits for HAT. Eighteen compounds showed good selectivity for trypanosomatid PTR1s, and only compound RUBi006 bound to the *Hs*DHRS4 active site but with a weaker binding energy than the trypanosomatids. MD simulations, DRN calculations, and MMPBSA free energy calculations indicated that all 18 compounds were potentially good hits. Of the 18, 13 commercially available compounds were tested for anti-trypanosomal activity using in vitro inhibition assays. Five compounds out of the 13 (RUBi004, RUBi007, RUBi014, RUBi16, and RUBi018) exhibited anti-trypanosomal activity against trypanosomes in culture, with IC_50_s of 9.6 ± 3.2 μM, 34.9 ± 17.1 μM, 14.6 ± 9.9 μM, 25.4 ± 4.7 μM, and 12.7 ± 3.7 μM, respectively. RUBi007, RUBi016, and RUBi018 showed no significant human (human cervix adenocarcinoma (HeLa)) cell cytotoxicity at 100 μM (HeLa cell viability was >90% at this concentration) while RUBi004 and RUBi014 had cytotoxicity IC_50_s of 23.6 ± 5.8 μM and 32.9 ± 2.2 μM, respectively. When used in combination with WR99210, RUBi004, RUBi007, RUBi014, and RUBi018 displayed antagonistic effects, while RUBi016 showed an additive effect in the isobologram assay.

When anti-folate drugs that target trypanosomatid dihydrofolate reductase-thymidylate synthase DHFR-TS are used, PTR1 is over-expressed, allowing for a by-pass mechanism to ensure parasite survival [8,13]. This is the escape mechanism that has hampered the use of traditional anti-folates against trypanosomatids. PTR1 is an important drug target, as demonstrated by gene knock out in *Leishmania* [8] and knock down in *Trypanosoma brucei* [17,35] studies that show that the enzyme is essential for parasite survival. However, *Tb*PTR1 is less susceptible to inhibition than *Tb*DHFR [21,35,38]. Given the nature of the interaction between *Tb*PTR1 and *Tb*DHFR, a combination therapy would offer several advantages, especially against resistance problems as has been shown by anti-malarial combination treatment strategies [35,46].

Our experimental data allow us to draw the following conclusions. Compounds RUBi004, RUB007, RUBi014, and RUBi018 inhibited parasite growth with IC_50_s of 9.6 ± 3.2 μM, 34.9 ± 17.1 μM, 14.6 ± 9.9 μM, and 12.7 ± 3.7 μM when assayed on their own. When used in combination with WR99210 (with an IC_50_ of 0.55 μM), which is a known *Tb*DHFR inhibitor, each compound showed an antagonistic effect. From our molecular docking studies, we demonstrate that it is reasonable that the compounds RUBi004, RUB007, RUBi014, and RUBi018 can bind both *Tb*PTR1 and *Tb*DHFR with good binding affinities and in binding modes similar to those of traditional folates, pterins, and known inhibitors. Our molecular dynamics simulations also show that the ligands bind to *Tb*PTR1 stably and with acceptable binding energies. In line with these results, we theorize that the compounds could be competing for the *Tb*DHFR active site with WR99210, which would result in the observed antagonism. Further, the resulting over-expression of *Tb*PTR1 would result in a further reduction in compound efficacy.

Compound RUBi016 inhibited parasite growth with an IC_50_ of 25.4 ± 4.7 μM when assayed on its own. From our molecular docking results, it appears that RUBi016 binds *Tb*DHFR with a lower binding affinity than *Tb*PTR1, given the binding affinity values of −7.6 kcal/mol and −8.9 kcal/mol, respectively. During MD simulations it bound to *Tb*PTR1 stably and had a good binding energy. Unlike the four compounds, RUBi016 showed an additive effect when used in combination with WR99210. We theorize that RUBi016 may be more selective for *Tb*PTR1 compared to the other compounds, which may have a tendency to bind to both PTR1 and DHFR. The other compounds thus display antagonistic effects with WR99210, while RUBi016 has an additive effect. In the case of RUBi016, the addition of WR99210 inhibits *Tb*DHFR, further impeding folate reduction and resulting in more inhibition of parasite survival, producing the observed additive effect. Furthermore, the fact that RUBi016 does not have the lowest IC_50_ value does not exclude the possibility that it might be the most selective of the compounds. Interestingly, the ratio of its binding energy for PTR1 and DHFR is the highest (except for RUBi018 for PTR1), which means that docking scores would predict it to be the most selective. For example, RUBi004 has the lowest IC_50_ value and the lowest binding energy, but it also has a very low binding energy for DHFR which means it is not really selective.

We note that compounds RUBi004 and RUBi014 contain pan-assay interference compounds (PAINS) features [47]. While this is a cause for concern, there are over 60 FDA approved drugs that have PAINS features [47,48]. The binding patterns of all five compounds are consistent with those of folates, pterins, and known inhibitors. Further analyses such as ligand structure activity relationship (SAR) analysis and protein-ligand co-crystallizations are required to validate these compounds as potential HAT anti-folate chemotherapeutics [49].

## 4. Materials and Methods

### 4.1. Ligand Library Preparation

The small-molecule ligands were obtained from the South African Natural Compounds database (SANCDB) [50] and the ZINC database (ZINC12) [51]. The compounds in the ZINC dataset already conformed to the Lipinski rules, and are commercially available drug-like compounds [51,52]. In the development of potential HAT chemotherapeutics, it is important to select for BBB permeability because in the second stage of the disease the parasites invade the CNS [53,54]. As such, the ligand filtering strategy was based on drug physicochemical properties that have been shown to be useful in predicting BBB permeability [55,56]. These included lipophilicity as calculated by XlogP, rotatable bonds, hydrogen donors, net charge, and molecular weight [55,56]. The values were determined in accordance with features common in CNS FDA-approved drugs and literature [56,57,58].

The ligand library was prepared by filtering 10,639,555 compounds from the ZINC Drugs Now subset [51,52] for compounds with XlogP ≤ 3, fewer than four rotatable bonds, at least two hydrogen donors, a net charge of zero, and a molecular weight ≤ 490. The ZINC subset was reduced to 5107 compounds after filtering, while the SANCDB contained 635 compounds. The final ligand library comprised of 5742 compounds.

### 4.2. Preparation of Protein-Ligand Complexes

A crystal structure of *Tb*PTR1 that has a resolution of 1.15 Å was retrieved from the RCSB Protein Data Bank (PDB: 2X9N) [26]. Multiple sequence analysis using MUSCLE [59] was carried out to analyze *Tb*PTR1 (Uniprot: O76290) and the homologues sequences, including *T. cruzi* (Uniprot: O44029), *L. major* (Uniprot: Q01782, PDB: 1E92), and the *H. sapiens* dehydrogenase/reductase SDR family member four (DHRS4) (Uniprot: Q9BTZ2, PDB: 3O4R). The crystal structure of *Tb*DHFR was also retrieved and had a resolution of 2.2 Å (PDB: 3QFX) [42]. Homology modelling was done using in-house Python scripts to fix missing residues in 2X9N (residues GLN104–GLY113 and LYS143–SER151) as well as to generate a homotetramer *Tc*PTR1 structure from its PTR2 isoform (Uniprot: Q8I814, PDB: 1MXH). The modelling was done by MODELLER (version 9.19, Departments of Biopharmaceutical Sciences and Pharmaceutical Chemistry, and California Institute for Quantitative Biomedical Research, San Diego, USA) using the ‘automodel’ class and included the NADPH cofactor [60,61]. For both *Tb*PTR1 and *Tc*PTR1, of the 100 models generated, the top models were validated using the ProSA [62] online server (Figure S15). A table gathering a summary of the *Tb*PTR1, *Tc*PTR1, *Lm*PTR1, *Hs*DHRS4, and *Tb*DHFR protein structures is presented in Table S4. The *Tb*PTR1 structure was co-crystallized with cyromazine, which was used to validate the docking procedure.

We carried out blind docking of the ligand library against *Tb*PTR1, *Tc*PTR1, *Lm*PTR1, and *Hs*DHRS4 homotetrameric protein structures that included their NADPH cofactors using Autodock Vina (version 7.4, Scripps Research Institute, San Francisco, USA) [63]. Later, the compounds were blind docked to the *Tb*DHFR (PDB: 3QFX) [42] dimeric structure that included its NADPH cofactors using Autodock Vina. The docking parameters used for each of the proteins are summarized in Table S5. Protein-ligand complexes were then evaluated based on if the ligand was located in the active site, as well as based on binding mode, selectivity, docking energy scores, and hydrogen bonding. The docking energies were further evaluated by re-docking the compounds to their protein targets and then using Xscore to give an independent energy score [32].

### 4.3. Prediction of Blood-Brain Barrier Permeability

To prioritize compounds that can cross the BBB, a PCA was carried out to identify which compounds occupied the same chemical space as known CNS-permeable drugs [64,65,66]. The PCA was based on the molecular descriptors of the top binding compounds, FDA-approved drugs, and FDA-approved CNS-permeable drugs [51]. The molecular descriptors used included XlogP, number of H-bond donors (HBD), number of H-bond acceptors (HBA), net charge (NC), topological polar surface area (tPSA), molecular weight (MWT), number of rotatable bonds (NRB), and polar and apolar desolvation. The first and second principal components were used to create a scatter plot that explained the largest percentage of the variance.

### 4.4. Molecular Dynamics

Eighteen protein-cofactor-ligand complexes were parametrized using the AMBER03 force field utilizing ACPYPE [67] and GROMACS (5.1.4) [68]. Each protein-ligand complex was solvated using a Simple Point Charge (SPC) water model in a cubic box of 5.07 × 5.18 × 5.16 nm with a 2.37 × 2.86 × 3.30 nm center. A minimum distance of 1.5 nm was allowed between any protein or ligand atom with the wall. The systems were then neutralized using Na^+^ and Cl^−^ counter ions. The MD systems also included simulating the protein in complex with the NADPH cofactor without the ligand. The MD simulations were performed using GROMACS 5.1.4 [68]. To correct for any structural distortions, the systems were minimized using a steepest descent algorithm using a 100 kJ/mol/nm tolerance value. This was followed by an equilibration using constant number of particles, pressure, and temperature (NPT) and constant number of particles, volume, and temperature (NVT) ensembles. This was finally followed by a 200 ns production run at 300 K without any restraints. Trajectories were generated every 2 fs with protein bonds involving hydrogens being constrained using a Linear Constraint Solver (LINCS) algorithm and saved after every 10 ps. The MD trajectory analysis included RMSD, Rg, RMSF, and PCA, using the GROMACS toolbox, Visual Molecular Dynamics (VMD) [69], and ProDy [70]. All MD simulations were performed at the Center for High Performance Computing (CHPC) (Cape Town, South Africa) using 240 cores (CPU: Intel ^®^ Xeon ^®^).

Graphs and diagrams were generated using, JChemPaint [63], PyMOL [29] and GRACE software (http://plasma-gate.weizmann.ac.il/Grace/). Protein-ligand complex structures were generated from the equilibrated trajectories at the end of the simulation. These structures were then used to analyze the protein-ligand interactions as well as residue interactions using Discovery Studio [31].

### 4.5. MM-PBSA Free Energy Calculations

The last 50 ns of the equilibrated MD trajectories were used to perform binding free energy (BFE) calculations of the ligand-protein complexes using the g_mmpbsa package (version 1.6, Jawaharlal Nehru University, New Delhi, India) [71]. The BFE calculation was based on the MM-PBSA method [72,73]. The BFE of the protein-ligand complexes was calculated using the equations below (in general terms):

Δ*G*_binding_ = *G*_complex_ − (*G*_protein_ + *G*_ligand_), (1)

*G*_x_ = <E_MM_> − TS + <*G*_solvation_> (2)

*E*_MM_ = *E*_bonded_ + *E*_nonbonded_ = *E*_bonded_ + (*E*_electrostatic_ + *E*_vdW_) (3)

*G*_solvation_ = *G*_polar_ + *G*_nonpolar_, (4)

(1) The binding free energy of the protein-ligand complex in solvent (Δ*G*_binding_) where *G*_complex_ is described as the total energy of the protein-ligand complex, *G*_protein_ as the isolated free energy of the protein and *G*_ligand_ as the isolated free energy of the ligand.

(2) The free energy of either the ligand, protein, or protein-ligand complex (*G*_x_), where the average mechanical potential in a vacuum is described as <E_MM_>, TS as the entropic contribution (T is temperature and S is entropy), and *G*_solvation_ as the free energy of solvation.

(3) The vacuum molecular mechanics potential energy (*E*_MM_), where *E*_bonded_ represents bonded interactions such as bonds, dihedrals, angles and improper interactions. The non-bonded interactions (*E*_nonbonded_) are modelled using the Coulomb and Lennard-Jones (LJ) potential functions. They include electrostatic interactions (*E*_electrostatic_) and van der Waals interactions (*E*_vdW_).

(4) The energy required to transfer the protein-ligand solute from a vacuum into a solvent is described as the free energy of solvation (*G*_solvation_), where *G*_polar_ and *G*_nonpolar_ describe the electrostatic and non-electrostatic energy contributions, respectively [71].

Furthermore, to determine the energy contribution of each protein residue that binds with the ligand, a free energy decomposition was carried out using g_mmpbsa [71]. This allowed for a better understanding of the protein-ligand interactions and helped identify PTR1 binding residues of functional significance.

### 4.6. Average Shortest Path, and Average Betweenness Centrality

We carried out dynamic network analysis on the equilibrated (after 50 ns) apo protein and protein-ligand MD trajectories using MD-TASK [43] in order to identify changes in the topological properties of the proteins brought about by the ligand interactions. This was used to glean the impacts of ligand binding on protein dynamics, function, and conformation. A cut off of 6.7 Å was used in the creation of the dynamic residue networks in MD-TASK. The average shortest path gave the density of shortest paths (*l*) between all node pairs [43]. The average betweenness centrality was used to identify residues in the dynamic network that were important for communication flow. Additionally, by comparing the apo protein and the protein-ligand complexes, we were able to use *Average BC* to assess how communication flow across the dynamic network was altered by ligand binding during the MD simulations. We generated equilibrated structures at the end of the simulations in order to map the interaction networks of any important residues identified using Discovery Studio [31].

### 4.7. Trypanosoma In Vitro Inhibition Assay

Compounds RUBi001, RUBi005, and RUBi015 were purchased from MCULE while RUBi002, RUBi004, RUBi007, RUBi008, RUBi010, RUBi011, RUBi012, RUBi014, RUBi016, and RUBi018 were obtained from MolPort (not all the compounds were commercially available). These compounds were assayed for trypanocidal activity by adding 20 μM of each compound to cultures of *T. b. brucei* (strain Lister 427) in 96-well plates. The parasites were maintained at 37 °C and 5% CO_2_ in an IMDM medium containing 25 mM HEPES, 10% fetal bovine serum, 1 mM hypoxanthine, 0.05 mM bathocuproine disulfonic acid, 1.5 mM cysteine, 1.25 mM pyruvic acid, 0.09 mM uracil, 0.09 mM cytosine, 0.16 mM thymidine, and 0.014 % 2-mercaptoethanol. Parasites were diluted to 2.4 × 10^4^ cells in a volume of 200 µL per well and incubated with the test compounds for 24 h. Parasite percentage viability was determined using the resazurin method [74]. Resazurin (0.5 mM in phosphate-buffered saline; 20 µL/well) was added to each well and incubation continued for a further 24 h, after which fluorescence (Ex_560_/Em_590_) was read in a Spectramax M3 microplate reader (Molecular Devices). Trypanocidal activity of the compounds was reported as the percentage of viable parasites in the compound-treated wells when compared to untreated controls (% viability). Pentamidine, an FDA-approved trypanocidal drug, was used as the control drug standard [75]. For compounds that produced <20% viability, IC_50_ values were subsequently determined. The assays were conducted as described above, except that parasites were incubated with 3-fold serial dilutions of the test compounds and IC_50_ values derived from % parasite viability vs. log[compound] dose-response plots by non-linear regression analysis using GraphPad Prism (version 5.02, GraphPad Software Inc., San Diego, USA). IC_50_ evaluations were carried out on three independent occasions and the mean IC_50_ ± standard deviation is reported in the text. To assess compound interactions, the compounds were assayed for trypanocidal activity when used in combination with WR99210, a known *Tb*DHFR inhibitor [42]. For combination assays, IC_50_ values were determined for RUBi004, RUBi007, RUBi014, RUBi016, and RUBi018 as well as WR99210 alone using a starting concentration of 100 µM and 20 µM for the RUBi compounds and WR99210, respectively, and in combinations at ratios of 75:25, 50:50 and 25:75, respectively (thus starting with concentrations of 75 µM/5 µM, 50 µM/10 µM, and 25 µM/15 µM for the RUBi compounds/WR99210). For isobologram analysis, the fractional inhibitory concentrations of the RUBi compounds and WR99210 were calculated by dividing the IC_50_s obtained for the compounds at the various combination ratios with the IC_50_ obtained for the compounds in the absence of the partner drug, and the FIC values plotted against each other, i.e., RUBi compound FIC versus WR99210 FIC.

### 4.8. In Vitro Human Cytotoxicity Assay

The compounds assayed for trypanocidal activity were also tested to determine if they caused adverse effects against human cells in vitro. For this assay, HeLa cells were used. The cells were cultured in DMEM supplemented with 10% fetal calf serum and antibiotics (penicillin/streptomycin/amphotericin B) at 37 °C in a 5 % CO_2_ incubator. Cells were plated at a density of 2 × 10^4^ cells/well and after an overnight incubation the compounds were assayed for cytotoxic activity by adding three-fold serial dilutions of each compound (with a 100 µM starting concentration) to the 96-well plates, followed by incubation for 48 h. Cell viability was determined using the resazurin method [74]. Resazurin (0.5 mM in phosphate-buffered saline; 20 µL/well) was added to the cells and, after a 2-h incubation, fluorescence was read in a Spectramax M3 plate reader at excitation and emission wavelengths of 560 nm and 590 nm, respectively. Fluorescence readings were converted to percentage cell viability relative to control wells untreated with compounds. Plots of % cell viability versus log[compound] were used to determine IC_50_ values by non-linear regression using GraphPad Prism (version 5.02, GraphPad Software Inc., San Diego, USA). Three repeats of the experiment were carried out. Emetine, a drug that induces cell apoptosis, was used as a control [76], and produced an IC_50_ of 0.013 µM.

### 4.9. Pan-Assay Interference Compounds Assay

Compounds that showed trypanocidal activity were also subjected to a PAINS assay using the web server located at http://www.cbligand.org/PAINS/. This was done to identify and flag any compounds that contained PAINS features [47,77].

## 5. Conclusions

Dual inhibition of *Tb*PTR1 and *Tb*DHFR is a promising approach to successfully developing safe and effective anti-folate based anti-trypanosomal chemotherapeutics. As shown in this study, computation-based approaches are useful in fast and rapid rational drug design. Furthermore, in the discovery of novel TbPTR1 inhibitors, when the compounds are assayed in combination with known DHFR inhibitors, careful interpretation of isobologram assays is required to obtain an optimal outcome. When used in combination with WR99210, a known *Tb*DHFR inhibitor, compounds RUBi004, RUBi007, RUBi014, and RUBi018 showed moderate to strong antagonism as demonstrated by isobologram results, which would indicate that they might be binding to both *Tb*PTR1 and *Tb*DHFR. RUBi016, as shown by its additive effect and molecular docking results, appears to selectively bind to TbPTR1. The five compounds assayed showed anti-trypanosomal activity with no significant human cell cytotoxicity in vitro. The merging of these scaffolds could yield to the development of even more potent and selective *Tb*PTR1 inhibitors.

## Figures and Tables

**Table 1:** The chemical structures of the top *Tb*PTR1 docking compounds and Autodock Vina molecular docking results. Protein Data Bank is abbreviated to PDB.

Compound Information			Docking Binding Energy (kcal/mol)			
Code Name	Chemical Structure	Database ID	*T. brucei* PDB: 2X9N	*T. cruzi* Homology model	*L. major* PDB: 1E92	*H. sapiens* PDB: 3O4R
RUBi001	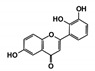	ZINC00057846	−10.1	−9.6	−9.2	-
RUBi002	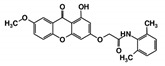	ZINC08992677	−10.2	−10.1	−9.8	-
RUBi003	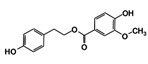	SANC00368	−9.1	−8.6	−8.1	-
RUBi004	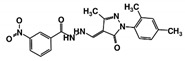	ZINC00809143	−10.3	−10.1	−9.1	-
RUBi005	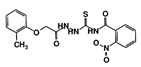	ZINC02690799	−9.0	−8.8	−8.6	-
RUBi006	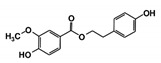	SANC00470	−10.2	−9.5	−8.6	−7.7 ^1^
RUBi007	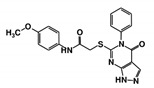	ZINC00630525	−9.6	−8.8	−9.1	-
RUBi008	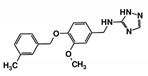	ZINC06556964	−8.5	−8.9	−8.6	-
RUBi009	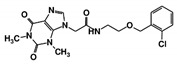	ZINC02177983	−8.9	−8.3	−8.2	-
RUBi010	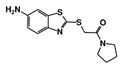	ZINC00359797	−6.9	−7.6	−7.8	-
RUBi011	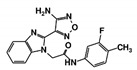	ZINC00677623	−9.7	−9.8	−9.7	-
RUBi012	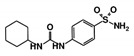	ZINC01003765	−9.1	−8.1	−7.9	-
RUBi013	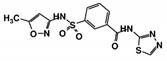	ZINC02184332	−8.7	−9.4	−7.9	-
RUBi014	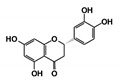	ZINC0058117/SANC00320	−9.7	−9.1	−9.7	-
RUBi015	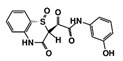	ZINC04671320	−9.1	−9.1	−8.3	-
RUBi016	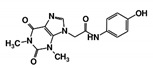	ZINC00612219	−8.9	−8.9	−7.7	-
RUBi017	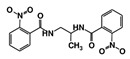	ZINC04523829	−8.8	−9.1	−8.4	-
RUBi018	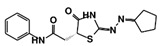	ZINC04313814	−8.4	−8.4	−8.8	-

^1^ RUBi006 was the only compound that bound to the human ortholog.

**Table 2:** A decomposition of the binding energy components obtained from MM-PBSA.

Ligand	Van der Waal Energy (kJ/mol)	Electrostatic Energy (kJ/mol)	Polar Solvation Energy (kJ/mol)	Solvent Accessible Surface Area (SASA) Energy (kJ/mol)	Binding Energy (kJ/mol)
RUBi001	−147 ± 10	−73 ± 8	114 ± 7	−13 ± 1	−120 ± 9
RUBi002	−158 ± 11	−55 ± 17	167 ± 25	−20 ± 1	−66 ± 18
RUBi003	−120 ± 10	−45 ± 16	89 ± 15	−12 ± 1	−89 ± 10
RUBi004	−89 ± 9	−2 ± 20	40 ± 29	−12 ± 1	−63 ± 14
RUBi005	−177 ± 13	−22 ± 11	106 ± 13	−19 ± 1	−112 ± 15
RUBi006	−130 ± 9	−3 ± 7	73 ± 7	−15 ± 1	−74 ± 9
RUBi007	−125 ± 8	−45 ± 7	97 ± 10	−15 ± 1	−88 ± 10
RUBi008	−134 ± 8	−46 ± 11	111 ± 15	−16 ± 1	−85 ± 11
RUBi009	−144 ± 19	2 ± 20	94 ± 13	−18 ± 2	−66 ± 22
RUBi010	−144 ± 11	−138 ± 32	231 ± 48	−16 ± 1	−68 ± 23
RUBi011	−159 ± 10	−22 ± 13	106 ± 16	−17 ± 1	−92 ± 12
RUBi012	−116 ± 12	41 ± 9	103 ± 21	−13 ± 1	15 ± 13
RUBi013	−140 ± 11	−49 ± 28	142 ± 31	−16 ± 1	−62 ± 13
RUBi014	−99 ± 17	−17 ± 20	71 ± 28	−11 ± 2	−56 ± 12
RUBi015	−106 ± 11	−29 ± 11	76 ± 17	−13 ± 1	−72 ± 12
RUBi016	−87 ± 9	21 ± 8	54 ± 11	−11 ± 1	−23 ± 10
RUBi017	−130 ± 12	−33 ± 14	92 ± 19	−15 ± 1	−86 ± 12
RUBi018	−132 ± 12	−15 ± 9	75 ± 11	−16 ± 1	−88 ± 12

## Supplementary Materials

The following are available online.

## References

[1] Achcar F., Kerkhoven E.J., Barrett M.P. (2013). *Trypanosoma brucei*: Meet the system. Curr. Opin. Microbiol..

[2] Franco J.R., Simarro P.P., Diarra A., Jannin J.G. (2014). Epidemiology of human African trypanosomiasis. Clin. Epidemiol..

[3] Funk S., Nishiura H., Heesterbeek H., Edmunds W.J., Checchi F. (2013). Identifying Transmission Cycles at the Human-Animal Interface: The Role of Animal Reservoirs in Maintaining Gambiense Human African Trypanosomiasis. PLoS Comput. Biol..

[4] Simarro P.P., Diarra A., Ruiz Postigo J.A., Franco J.R., Jannin J.G. (2011). The Human African trypanosomiasis control and surveillance programme of the World Health Organization 2000–2009: The way forward. PLoS Negl. Trop. Dis..

[5] Simarro P.P., Cecchi G., Paone M., Franco J.R., Diarra A., Ruiz J.A., Fèvre E.M., Courtin F., Mattioli R.C., Jannin J.G. (2010). The Atlas of human African trypanosomiasis: A contribution to global mapping of neglected tropical diseases. Int. J. Health Geogr..

[6] Barrett M.P., Gilbert I.H. (2006). Targeting of Toxic Compounds to the Trypanosome’s Interior. Adv. Parasitol..

[7] Fox J.T., Stover P.J. (2008). Folate-mediated one-carbon metabolism. Vitam. Horm..

[8] Bello A.R., Nare B., Freedman D., Hardy L., Beverley S.M. (1994). PTR1: A reductase mediating salvage of oxidized pteridines and methotrexate resistance in the protozoan parasite Leishmania major. Proc. Natl. Acad. Sci. USA.

[9] Berriman M., Ghedin E., Hertz-Fowler C., Blandin G., Renauld H., Bartholomeu D.C., Lennard N.J., Caler E., Hamlin N.E., Haas B. (2005). The genome of the African trypanosome *Trypanosoma brucei*. Science.

[10] Gangjee A., Jain H.D., Kurup S. (2008). Recent advances in classical and non-classical antifolates as antitumor and antiopportunistic infection agents: Part II. Anticancer Agents Med. Chem..

[11] Zuccotto F., Martin A.C.R., Laskowski R.A., Thornton J.M., Gilbert I.H. (1998). Dihydrofolate reductase: A potential drug target in trypanosomes and leishmania. J. Comput. Aided Mol. Des..

[12] Sienkiewicz N., Jarosławski S., Wyllie S., Fairlamb A.H. (2008). Chemical and genetic validation of dihydrofolate reductase-thymidylate synthase as a drug target in African trypanosomes. Mol. Microbiol..

[13] Nare B., Hardy L.W., Beverley S.M., Ptrs L. (1997). The Roles of Pteridine Reductase 1 and Dihydrofolate Reductase-Thymidylate Synthase in Pteridine Metabolism in the Protozoan Parasite Leishmania major. J. Biol. Chem..

[14] Vickers T.J., Beverley S.M. (2011). Folate metabolic pathways in Leishmania. Essays Biochem..

[15] Robello C., Navarro P., Castanys S., Gamarro F. (1997). A pteridine reductase gene ptr1 contiguous to a P-glycoprotein confers resistance to antifolates in Trypanosoma cruzi. Mol. Biochem. Parasitol..

[16] Gourley D.G., Schüttelkopf A.W., Leonard G.A., Luba J., Hardy L.W., Beverley S.M., Hunter W.N. (2001). Pteridine reductase mechanism correlates pterin metabolism with drug resistance in trypanosomatid parasites. Nat. Struct. Biol..

[17] Sienkiewicz N., Ong H.B., Fairlamb A.H. (2010). *Trypanosoma brucei* pteridine reductase 1 is essential for survival in vitro and for virulence in mice. Mol. Microbiol..

[18] Cavazzuti A., Paglietti G., Hunter W.N., Gamarro F., Piras S., Loriga M., Allecca S., Corona P., McLuskey K., Tulloch L. (2008). Discovery of potent pteridine reductase inhibitors to guide antiparasite drug development. Proc. Natl. Acad. Sci. USA.

[19] Tulloch L.B., Martini V.P., Iulek J., Huggan J.K., Lee J.H., Gibson C.L., Smith T.K., Suckling C.J., Hunter W.N. (2010). Structure-based design of pteridine reductase inhibitors targeting African sleeping sickness and the leishmaniases. J. Med. Chem..

[20] Mpamhanga C.P., Spinks D., Tulloch L.B., Shanks E.J., Robinson D.A., Collie I.T., Fairlamb A.H., Wyatt P.G., Frearson J.A., Hunter W.N. (2009). One scaffold, three binding modes: Novel and selective pteridine reductase 1 inhibitors derived from fragment hits discovered by virtual screening. J. Med. Chem..

[21] Hardy L.W., Matthews W., Nare B., Beverley S.M. (1997). Biochemical and genetic tests for inhibitors of Leishmania pteridine pathways. Exp. Parasitol..

[22] Nare B., Luba J., Hardy L.W., Beverley S. (1997). New approaches to Leishmania chemotherapy: Pteridine reductase 1 (PTR1) as a target and modulator of antifolate sensitivity. Parasitology.

[23] Matovu E., Seebeck T., Enyaru J.C.K., Kaminsky R. (2001). Drug resistance in *Trypanosoma brucei* spp., the causative agents of sleeping sickness in man and nagana in cattle. Microbes Infect..

[24] Babokhov P., Sanyaolu A.O., Oyibo W., Fagbenro-Beyioku A.F., Iriemenam N.C. (2013). A current analysis of chemotherapy strategies for the treatment of human African trypanosomiasis. Pathog. Glob. Health.

[25] Mesu V.K.B.K., Kalonji W.M., Bardonneau C., Mordt O.V., Blesson S., Simon F., Delhomme S., Bernhard S., Kuziena W., Lubaki J.P.F. (2018). Oral fexinidazole for late-stage African *Trypanosoma brucei* gambiense trypanosomiasis: A pivotal multicentre, randomised, non-inferiority trial. Lancet.

[26] Dawson A., Tulloch L.B., Barrack K.L., Hunter W.N. (2010). High-resolution structures of *Trypanosoma brucei* pteridine reductase ligand complexes inform on the placement of new molecular entities in the active site of a potential drug target. Acta Crystallogr. Sect. D Biol. Crystallogr..

[27] Luba J., Nare B., Liang P.H., Anderson K.S., Beverley S.M., Hardy L.W. (1998). Leishmania major pteridine reductase 1 belongs to the short chain dehydrogenase family: Stereochemical and kinetic evidence. Biochemistry.

[28] Schormann N., Pal B., Senkovich O., Carson M., Howard A., Smith C., DeLucas L., Chattopadhyay D. (2005). Crystal structure of Trypanosoma cruzi pteridine reductase 2 in complex with a substrate and an inhibitor. J. Struct. Biol..

[29] Fiser A., Šali A. (2003). MODELLER: Generation and Refinement of Homology-Based Protein Structure Models. Methods Enzymol..

[30] DeLano W.L. The PyMOL Molecular Graphics System. http://www.pymol.

[31] Accelrys Software Inc. (2012). Discovery Studio Modeling Environment, Release 3.5.

[32] Obiol-Pardo C., Rubio-Martinez J. (2007). Comparative evaluation of MMPBSA and XSCORE to compute binding free energy in XIAP-peptide complexes. J. Chem. Inf. Model..

[33] Legros D., Ollivier G., Gastellu-Etchegorry M., Paquet C., Burri C., Jannin J., Büscher P. (2002). Treatment of human African trypanosomiasis—Present situation and needs for research and development. Lancet Infect. Dis..

[34] David C.C., Jacobs D.J. (2014). Principal component analysis: A method for determining the essential dynamics of proteins. Methods Mol. Biol..

[35] Dawson A., Gibellini F., Sienkiewicz N., Tulloch L.B., Fyfe P.K., McLuskey K., Fairlamb A.H., Hunter W.N. (2006). Structure and reactivity of *Trypanosoma brucei* pteridine reductase: Inhibition by the archetypal antifolate methotrexate. Mol. Microbiol..

[36] Sawaya M.R., Kraut J. (1997). Loop and subdomain movements in the mechanism of Escherichia coli dihydrofolate reductase: Crystallographic evidence. Biochemistry.

[37] Schnell J.R., Dyson H.J., Wright P.E. (2004). Structure, Dynamics, and Catalytic Function of Dihydrofolate Reductase. Annu. Rev. Biophys. Biomol. Struct..

[38] Schüttelkopf A.W., Hardy L.W., Beverley S.M., Hunter W.N. (2005). Structures of Leishmania major pteridine reductase complexes reveal the active site features important for ligand binding and to guide inhibitor design. J. Mol. Biol..

[39] van Baren C., Martino V., Di Leo Lira P., Debenedetti S., Houghton P., Croft S., Martino V. (2006). Triterpenic Acids and Flavonoids from Satureja parvifolia. Evaluation of their Antiprotozoal Activity. Zeitschrift fur Naturforsch—Sect. C J. Biosci..

[40] Tasdemir D., Kaiser M., Brun R., Yardley V., Schmidt T.J., Tosun F., Rüedi P. (2006). Antitrypanosomal and antileishmanial activities of flavonoids and their analogues: In vitro, in vivo, structure-activity relationship, and quantitative structure-activity relationship studies. Antimicrob. Agents Chemother..

[41] Hassan A.A., Ibrahim Y.R., El-Sheref E.M., Abdel-Aziz M., Braese S., Nieger M. (2013). Synthesis and antibacterial activity of 4-Aryl-2-(1-substituted ethylidene)thiazoles. Arch. Pharm. (Weinheim).

[42] Vanichtanankul J., Taweechai S., Yuvaniyama J., Vilaivan T., Chitnumsub P., Kamchonwongpaisan S., Yuthavong Y. (2011). Trypanosomal dihydrofolate reductase reveals natural antifolate resistance. ACS Chem. Biol..

[43] Brown D.K., Penkler D.L., Sheik Amamuddy O., Ross C., Atilgan A.R., Atilgan C., Tastan Bishop Ö. (2017). MD-TASK: A software suite for analyzing molecular dynamics trajectories. Bioinformatics.

[44] Penkler D., Atilgan C., Tastan Bishop O. (2018). Allosteric Modulation of Human Hsp90α Conformational Dynamics. J. Chem. Inf. Model..

[45] Atilgan A.R., Akan P., Baysal C. (2004). Small-World Communication of Residues and Significance for Protein Dynamics. Biophys. J..

[46] Olliaro P.L. (2003). Antimalarial compounds: From bench to bedside. J. Exp. Biol..

[47] Kenny P.W. (2017). Comment on the Ecstasy and Agony of Assay Interference Compounds. J. Chem. Inf. Model..

[48] Kilchmann F., Marcaida M.J., Kotak S., Schick T., Boss S.D., Awale M., Gonczy P., Reymond J.L. (2016). Discovery of a Selective Aurora A Kinase Inhibitor by Virtual Screening. J. Med. Chem..

[49] Dahlin J.L., Walters M.A. (2016). How to Triage PAINS-Full Research. Assay Drug Dev. Technol..

[50] Hatherley R., Brown D.K., Musyoka T.M., Penkler D.L., Faya N., Lobb K.A., Bishop Ö.T. (2015). SANCDB: A South African natural compound database. J. Cheminform..

[51] Irwin J.J., Shoichet B.K. (2005). ZINC—A free database of commercially available compounds for virtual screening. J. Chem. Inf. Model..

[52] Lipinski C.A. (2000). Drug-like properties and the causes of poor solubility and poor permeability. J. Pharmacol. Toxicol. Methods.

[53] Bacchi C.J. (2009). Chemotherapy of Human African Trypanosomiasis. Interdiscip. Perspect. Infect. Dis..

[54] World Health Organization WHO (2016). Trypanosomiasis, Human African (Sleeping Sickness).

[55] Hitchcock S.A. (2008). Blood-brain barrier permeability considerations for CNS-targeted compound library design. Curr. Opin. Chem. Biol..

[56] Geldenhuys W.J., Mohammad A.S., Adkins C.E., Lockman P.R. (2015). Molecular determinants of blood-brain barrier permeation. Ther. Deliv..

[57] Wager T.T., Hou X., Verhoest P.R., Villalobos A. (2010). Moving beyond rules: The development of a central nervous system multiparameter optimization (CNS MPO) approach to enable alignment of druglike properties. ACS Chem. Neurosci..

[58] Ghose A.K., Herbertz T., Hudkins R.L., Dorsey B.D., Mallamo J.P. (2012). Knowledge-based, central nervous system (CNS) lead selection and lead optimization for CNS drug discovery. ACS Chem. Neurosci..

[59] Edgar R.C. (2004). MUSCLE: Multiple sequence alignment with high accuracy and high throughput. Nucl. Acids Res..

[60] Sali A., Blundell T.L. (1993). Comparative protein modelling by satisfaction of spatial restraints. J. Mol. Biol..

[61] Eswar N., Webb B., Marti-Renom M., Madhusudhan M.S., Eramian D., Shen M.Y., Pieper U., Sali A. (2007). Comparative protein structure modeling using MODELLER. Curr. Protoc. Protein Sci..

[62] Wiederstein M., Sippl M.J. (2007). ProSA-web: Interactive web service for the recognition of errors in three-dimensional structures of proteins. Nucl. Acids Res..

[63] Trott O., Olson A. (2010). AutoDock Vina: Inproving the speed and accuracy of docking with a new scoring function, efficient optimization and multithreading. J. Comput. Chem..

[64] Clemons P.A., Wilson J.A., Dancik V., Muller S., Carrinski H.A., Wagner B.K., Koehler A.N., Schreiber S.L. (2011). Quantifying structure and performance diversity for sets of small molecules comprising small-molecule screening collections. Proc. Natl. Acad. Sci. USA.

[65] Singh N., Guha R., Giulianotti M.A., Pinilla C., Houghten R.A., Medina-Franco J.L. (2009). Chemoinformatic analysis of combinatorial libraries, drugs, natural products, and molecular libraries Small Molecule Repository. J. Chem. Inf. Model..

[66] Doniger S., Hofmann T., Yeh J. (2002). Predicting CNS permeability of drug molecules: Comparison of neural network and support vector machine algorithms. J. Comput. Biol..

[67] Sousa da Silva A.W., Vranken W.F., Wang J. (2012). ACPYPE—AnteChamber PYthon Parser interfacE. BMC Res. Notes.

[68] Van Der Spoel D., Lindahl E., Hess B., Groenhof G., Mark A.E., Berendsen H.J. (2005). GROMACS: Fast, flexible, and free. J. Comput. Chem..

[69] Humphrey W., Dalke A., Schulten K. (1996). VMD: Visual molecular dynamics. J. Mol. Graph..

[70] Bakan A., Meireles L.M., Bahar I. (2011). ProDy: Protein dynamics inferred from theory and experiments. Bioinformatics.

[71] Kumari R., Kumar R., Lynn A. (2014). G-mmpbsa-A GROMACS tool for high-throughput MM-PBSA calculations. J. Chem. Inf. Model..

[72] Homeyer N., Gohlke H. (2012). Free energy calculations by the Molecular Mechanics Poisson-Boltzmann Surface Area method. Mol. Inform..

[73] Hou T., Wang J., Li Y., Wang W. (2011). Assessing the performance of the MM/PBSA and MM/GBSA methods. 1. The accuracy of binding free energy calculations based on molecular dynamics simulations. J. Chem. Inf. Model..

[74] Bowling T., Mercer L., Don R., Jacobs R., Nare B. (2012). Application of a resazurin-based high-throughput screening assay for the identification and progression of new treatments for human African trypanosomiasis. Int. J. Parasitol. Drugs Drug Resist..

[75] Doua F., Miezan T.W., Sanon Singaro J.R., Yapo F.B., Baltz T. (1996). The efficacy of pentamidine in the treatment of early-late stage *Trypanosoma brucei* gambiense trypanosomiasis. Am. J. Trop. Med. Hyg..

[76] Akinboye E. (2011). Biological Activities of Emetine. Open Nat. Prod. J..

[77] Baell J.B., Holloway G.A. (2010). New substructure filters for removal of pan assay interference compounds (PAINS) from screening libraries and for their exclusion in bioassays. J. Med. Chem..

