# Supplementary material for: Identification of Novel Potential Inhibitors of Pteridine Reductase 1 in Trypanosoma brucei via Computational Structure-Based Approaches and in Vitro Inhibition Assays
Source: Molecules. 2019 Jan 1;24(1):142. doi: 10.3390/molecules24010142 (PMC6337619; doi:10.3390/molecules24010142)
Supplement: Supplementary file 1 [file molecules-24-00142-s001.pdf]

Identification of Novel Potential Inhibitors of Pteridine Reductase 1 in *Trypanosoma brucei* via Computational Structure-Based Approaches and *in vitro* Inhibition Assays

Magambo Phillip Kimuda [1,2,3], Dustin Laming [4,5], Heinrich C. Hoppe [4,5] and Özlem Tastan Bishop[1,*]

[1] Research Unit in Bioinformatics (RUBi), Department of Biochemistry and Microbiology, Rhodes University, P.O. Box 94, Grahamstown 6140, South Africa; pjkanywa@gmail.com (M.P.K.); o.tastanbishop@ru.ac.za (O.T.B.)

[2] College of Veterinary Medicine, Animal Resources and Biosecurity (COVAB), Makerere University, P.O. Box 7062, Kampala, Uganda

[3] Member of the Trypanogen Consortium, www.trypanogen.net

[4] Department of Biochemistry and Microbiology, Rhodes University, Grahamstown 6140, South Africa; dustinlaming89@gmail.com (D.L.); H.Hoppe@ru.ac.za (H.C.H.)

[5] Centre for Chemico- and Biomedicinal Research, Rhodes University, Grahamstown 6140, South Africa;

*****Correspondence: o.tastanbishop@ru.ac.za; Tel.: +27-46-603-8072

Supplementary Figure S1: Analysis of the apo protein during 200 ns all atom MD simulation at 300 K. A) Root Mean Square Deviation of the protein back bone heavy atoms, B) Radius of Gyration, C) Per residue Root Mean Square Deviation, D) Average Shortest Path, and E) Average Betweenness Centrality

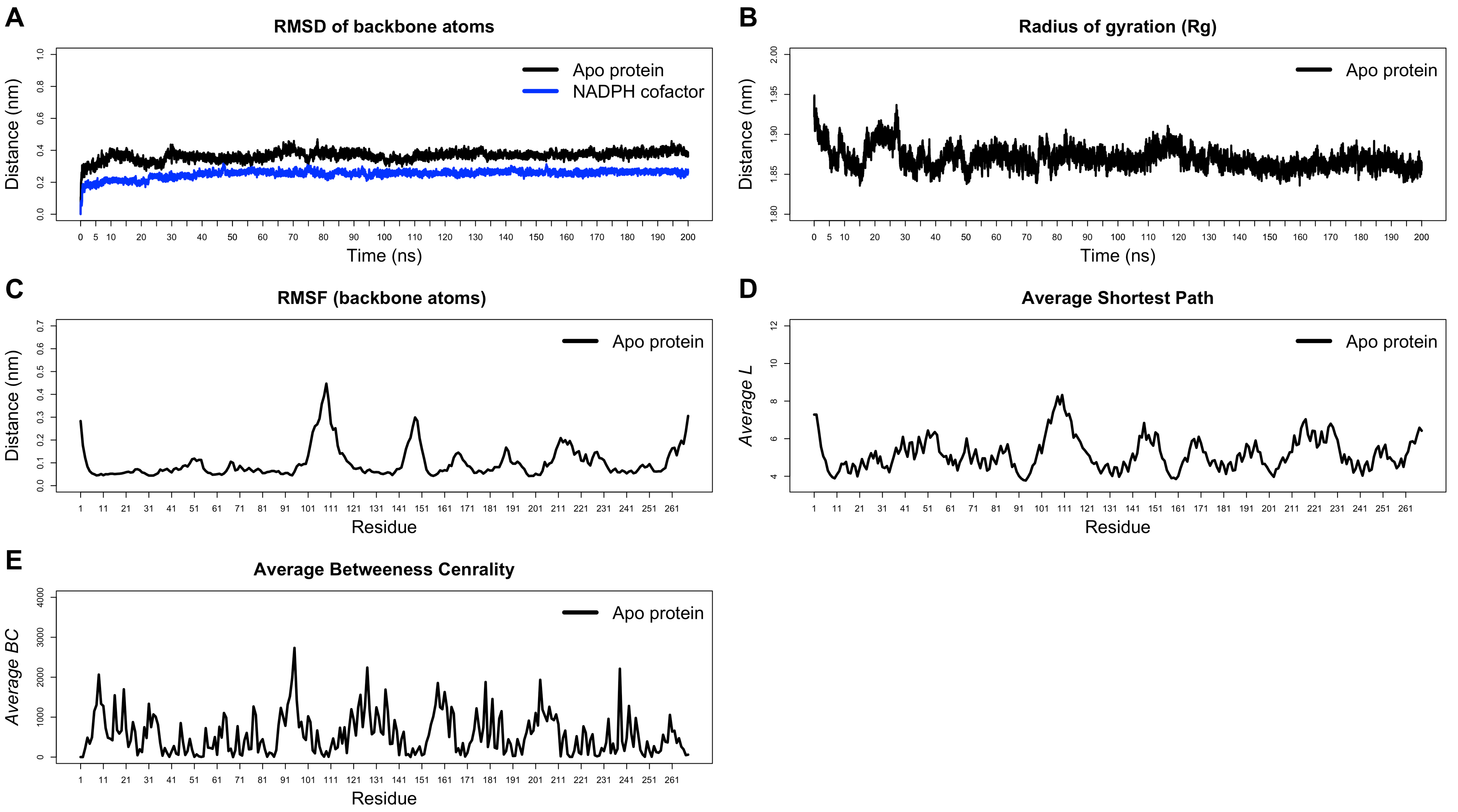

Supplementary Figure S2: Analysis of the TbPTR1-RUBi004 complex during 200 ns all atom MD simulation at 300 K. A) Root Mean Square Deviation of the protein back bone heavy atoms, B) Radius of Gyration, C) Protein-ligand Hydrogen bond analysis, D) Per residue Root Mean Square Deviation, E) Average Shortest Path, and F) Average Betweenness Centrality

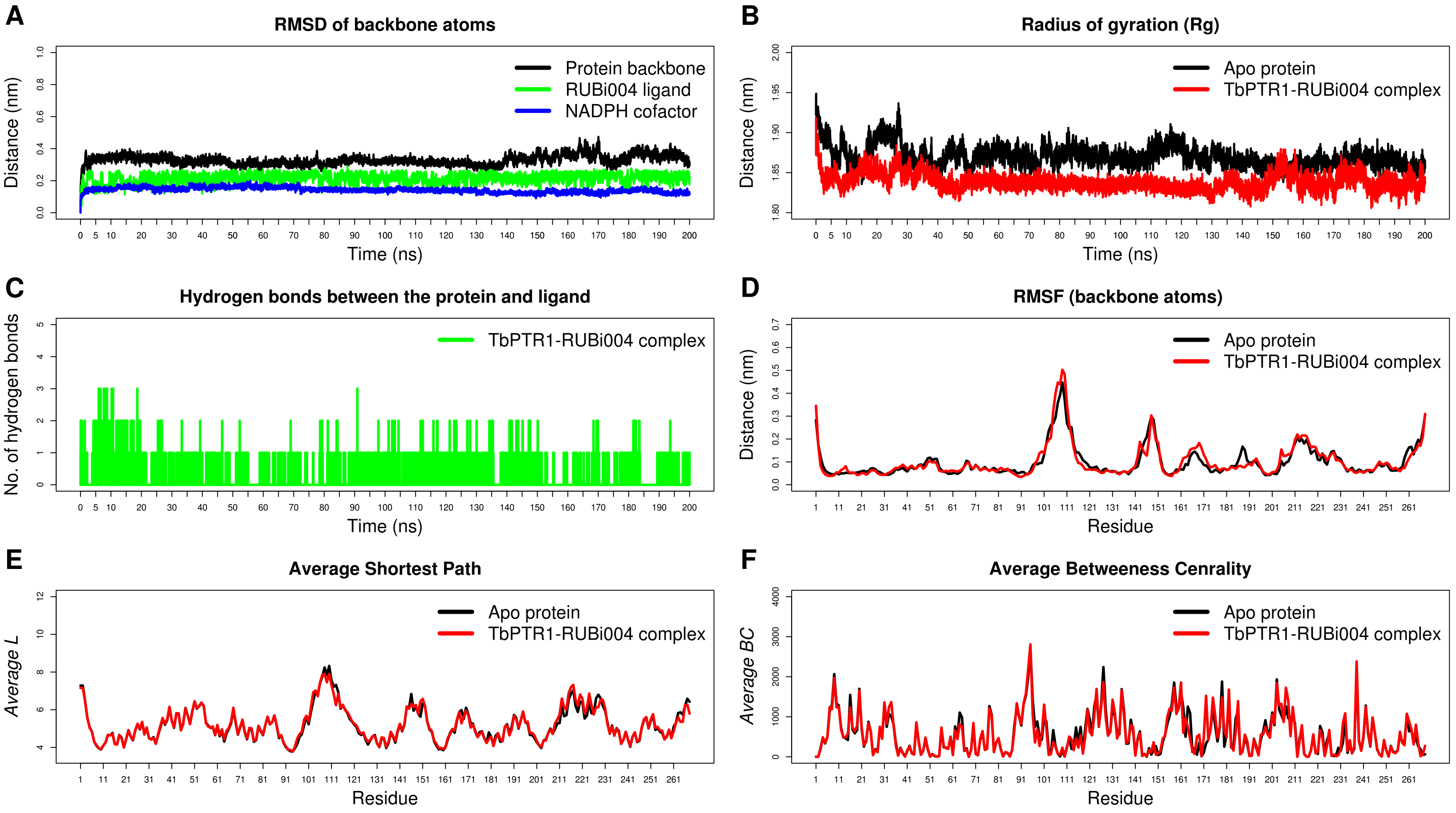

Supplementary Figure S3: Analysis of the TbPTR1-RUBi007 complex during 200 ns all atom MD simulation at 300 K. A) Root Mean Square Deviation of the protein back bone heavy atoms, B) Radius of Gyration, C) Protein-ligand Hydrogen bond analysis, D) Per residue Root Mean Square Deviation, E) Average Shortest Path, and F) Average Betweenness Centrality

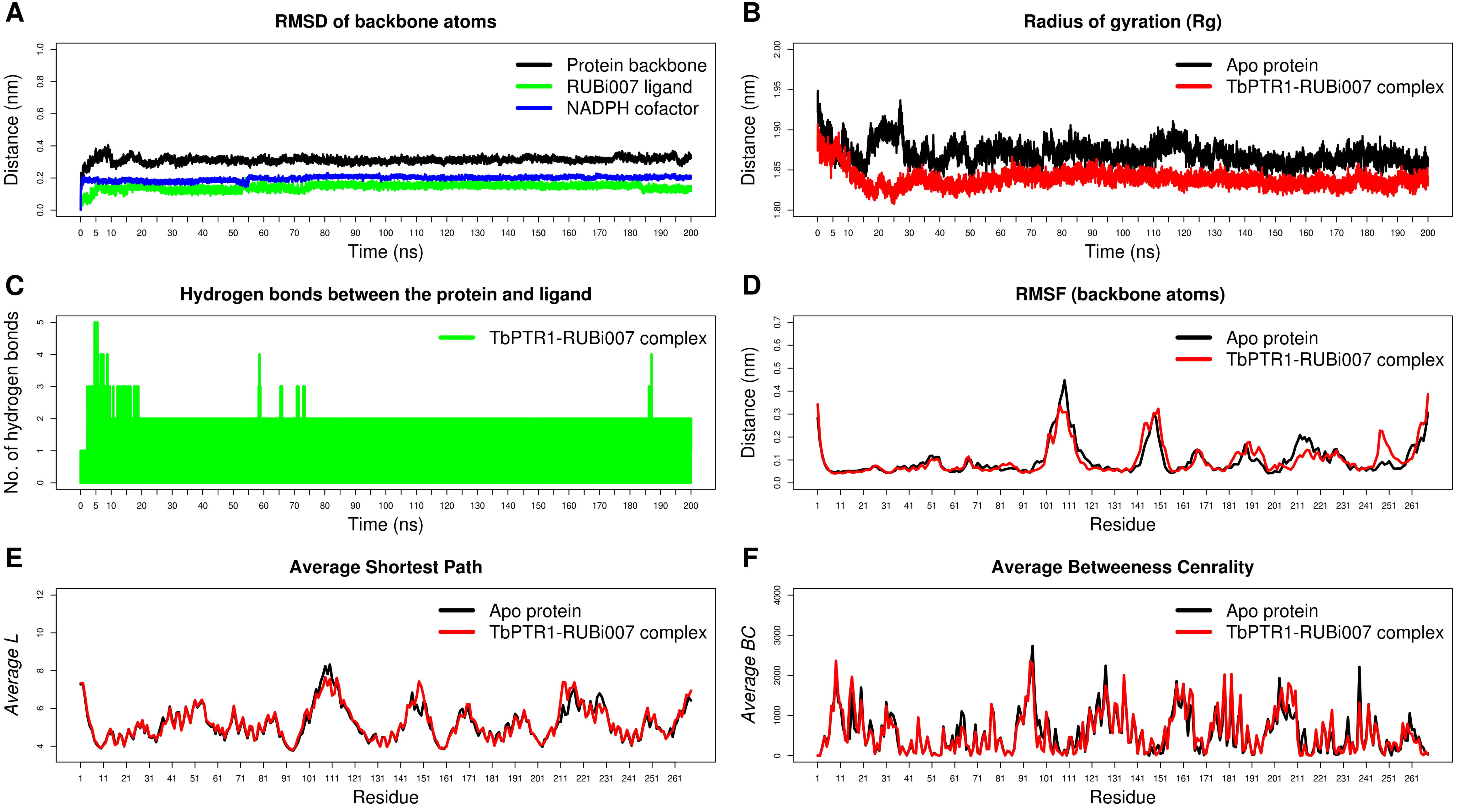

Supplementary Figure S4: Analysis of the TbPTR1-RUBi014 complex during 200 ns all atom MD simulation at 300 K. A) Root Mean Square Deviation of the protein back bone heavy atoms, B) Radius of Gyration, C) Protein-ligand Hydrogen bond analysis, D) Per residue Root Mean Square Deviation, E) Average Shortest Path, and F) Average Betweenness Centrality

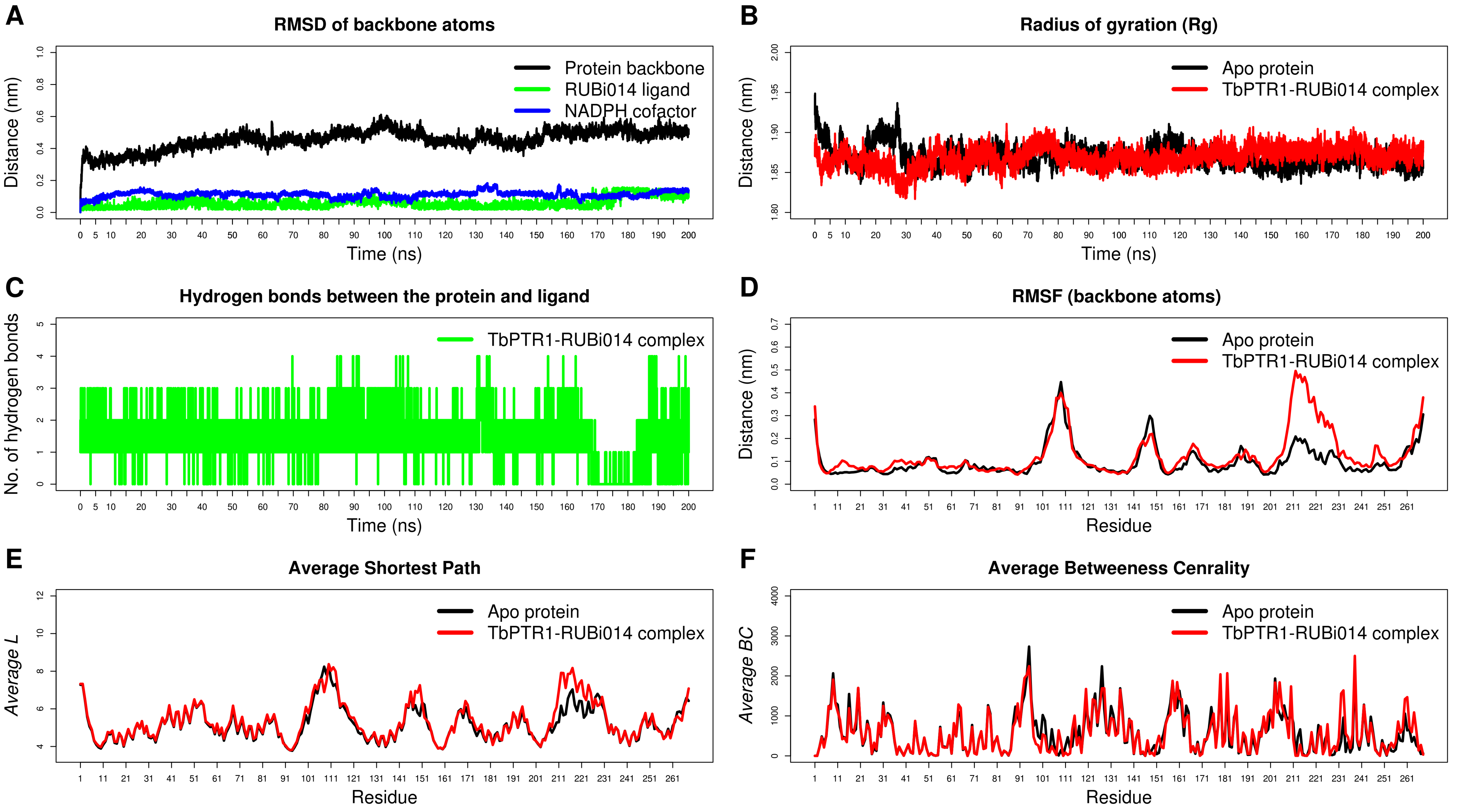

Supplementary Figure S5: Analysis of the TbPTR1-RUBi016 complex during 200 ns all atom MD simulation at 300 K. A) Root Mean Square Deviation of the protein back bone heavy atoms, B) Radius of Gyration, C) Protein-ligand Hydrogen bond analysis, D) Per residue Root Mean Square Deviation, E) Average Shortest Path, and F) Average Betweenness Centrality

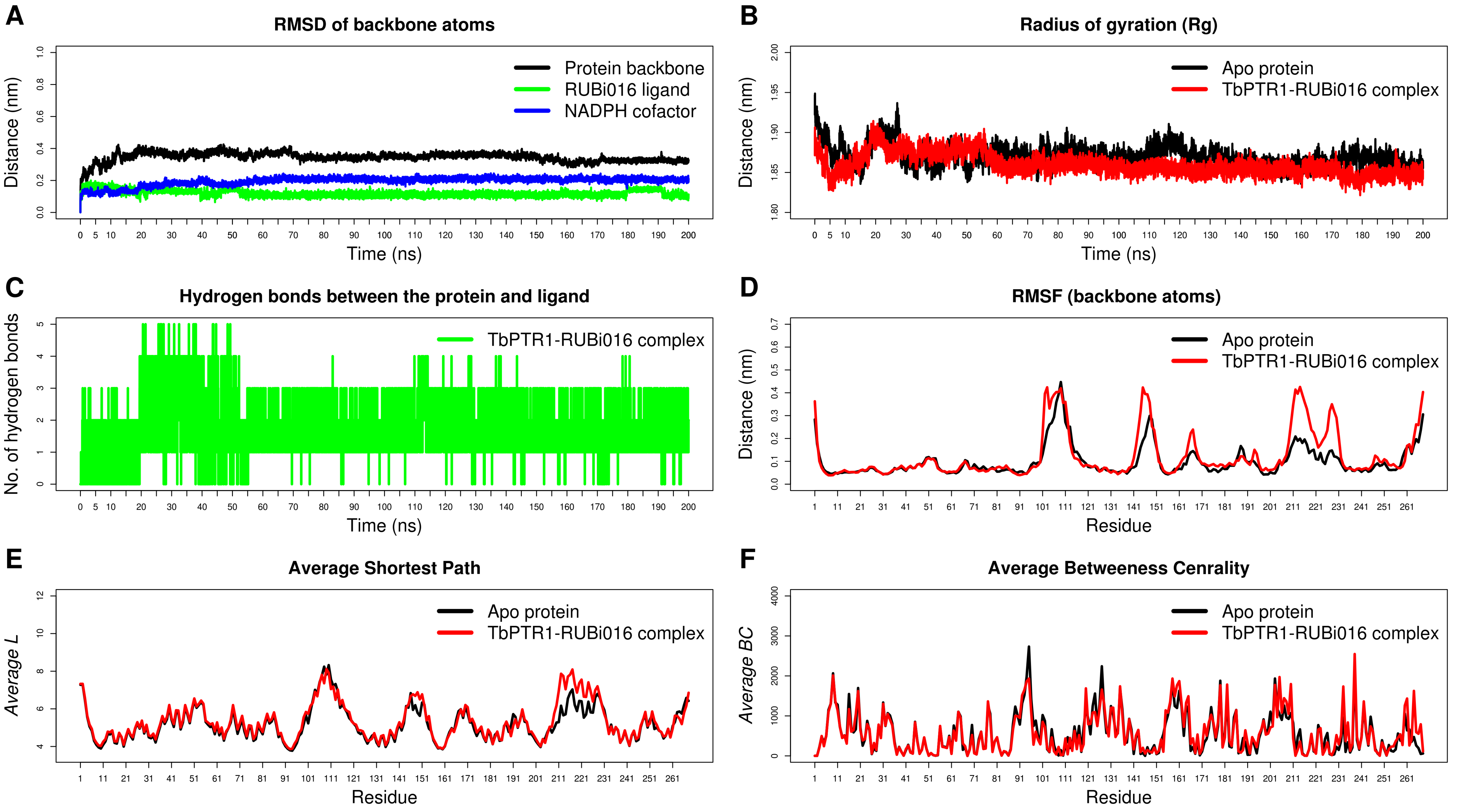

Supplementary Figure S6: Analysis of the TbPTR1-RUBi018 complex during 200 ns all atom MD simulation at 300 K. A) Root Mean Square Deviation of the protein back bone heavy atoms, B) Radius of Gyration, C) Protein-ligand Hydrogen bond analysis, D) Per residue Root Mean Square Deviation, E) Average Shortest Path, and F) Average Betweenness Centrality

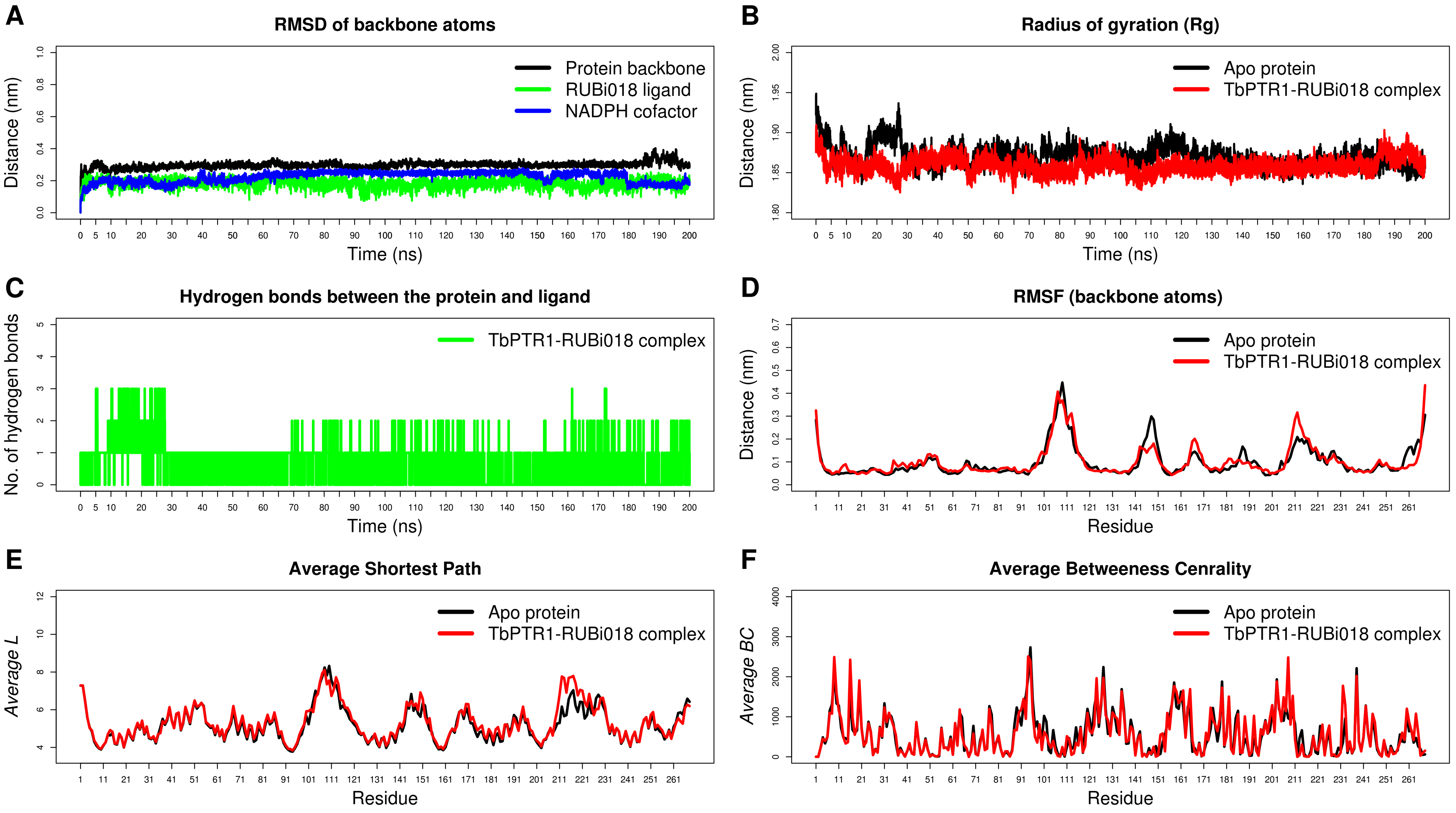

Supplementary Figure S7: RMSD values of *Tb*PTR1 and 18 *Tb*PTR1-ligand complexes

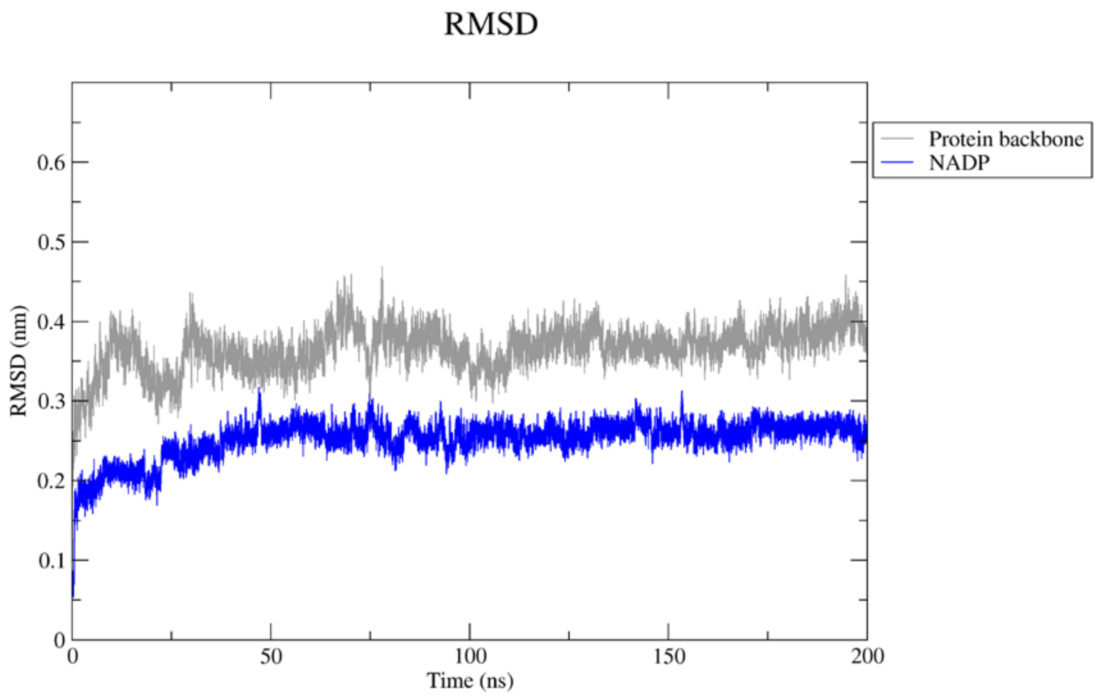

Figure 7A: Time dependent Root Mean Square Deviation (RMSD) of the backbone atoms of the apo protein, and NADP cofactor. The protein is coloured is coloured grey, and the NADP cofactor coloured blue.

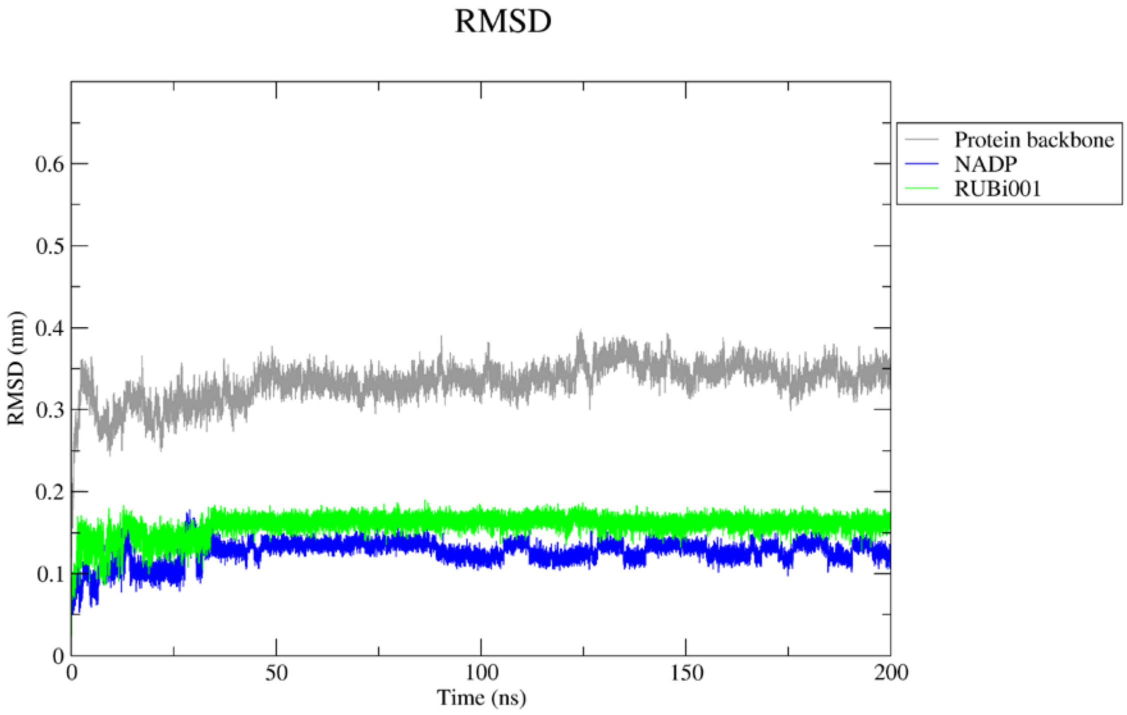

Figure 7B: Time dependent Root Mean Square Deviation (RMSD) of the backbone atoms of the apo protein, NADP, and ligand. The protein is coloured is coloured grey, the NADP cofactor coloured blue, and RUBi001 green.

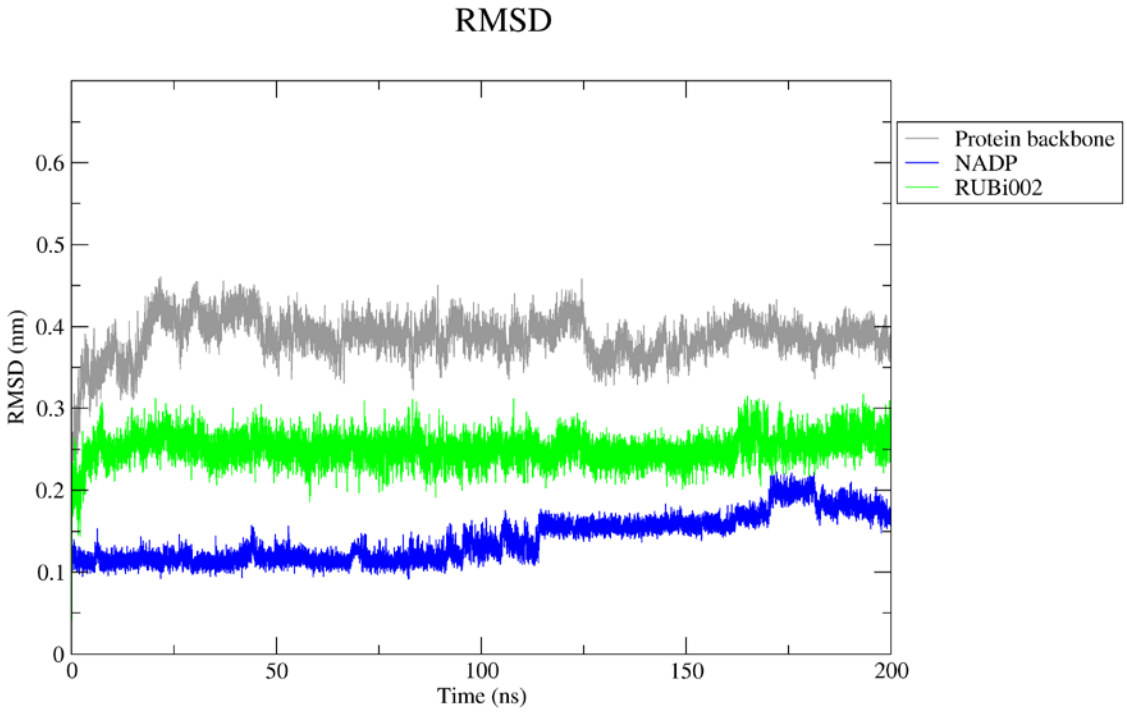

Figure 7C: Time dependent Root Mean Square Deviation (RMSD) of the backbone atoms of the apo protein, NADP, and ligand. The protein is coloured is coloured grey, the NADP cofactor coloured blue, and RUBi002 green.

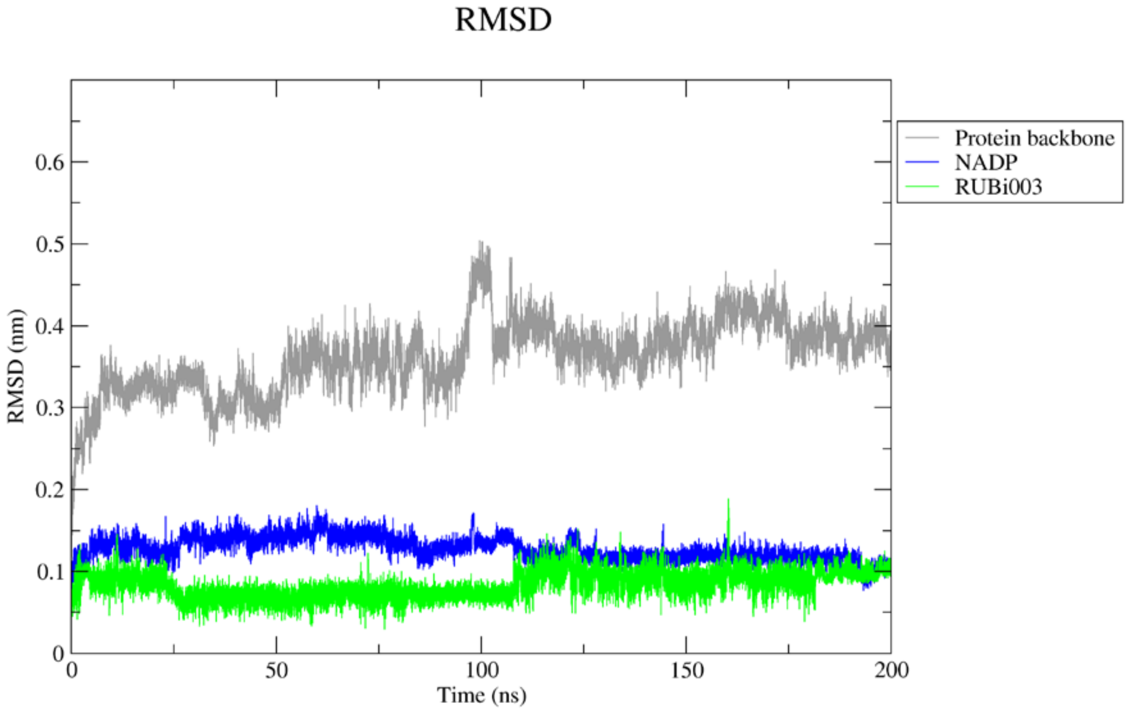

Figure 7D: Time dependent Root Mean Square Deviation (RMSD) of the backbone atoms of the apo protein, NADP, and ligand. The protein is coloured is coloured grey, the NADP cofactor coloured blue, and RUBi003 green.

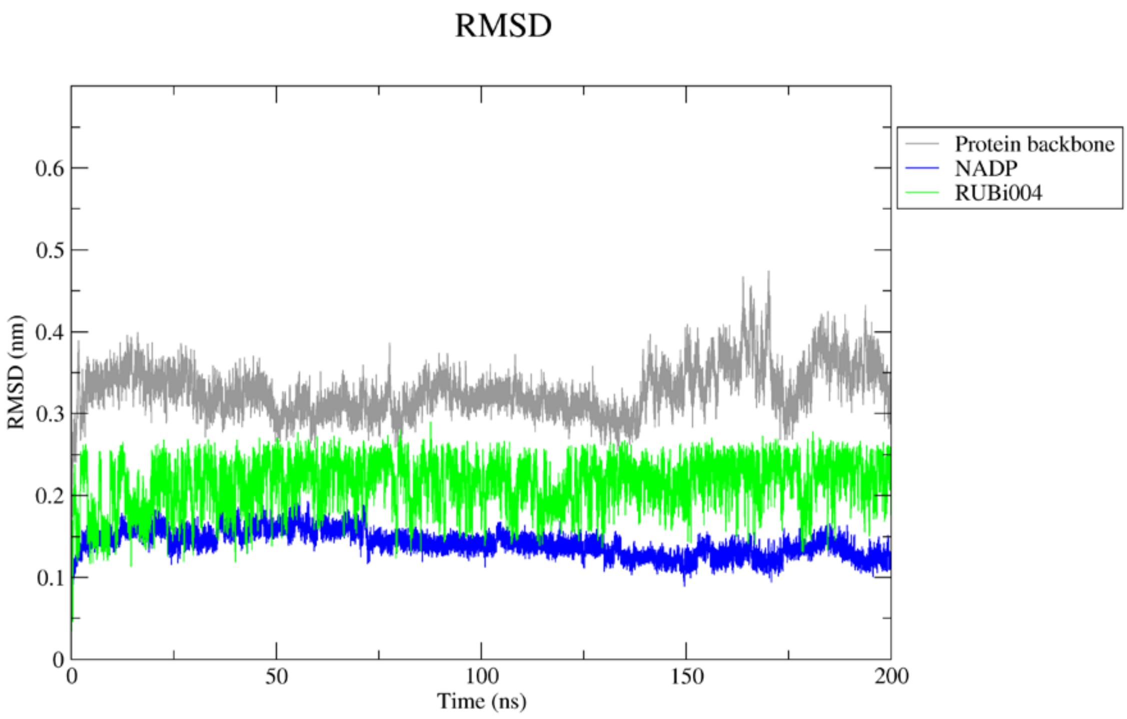

Figure 7E: Time dependent Root Mean Square Deviation (RMSD) of the backbone atoms of the apo protein, NADP, and ligand. The protein is coloured is coloured grey, the NADP cofactor coloured blue, and RUBi004 green.

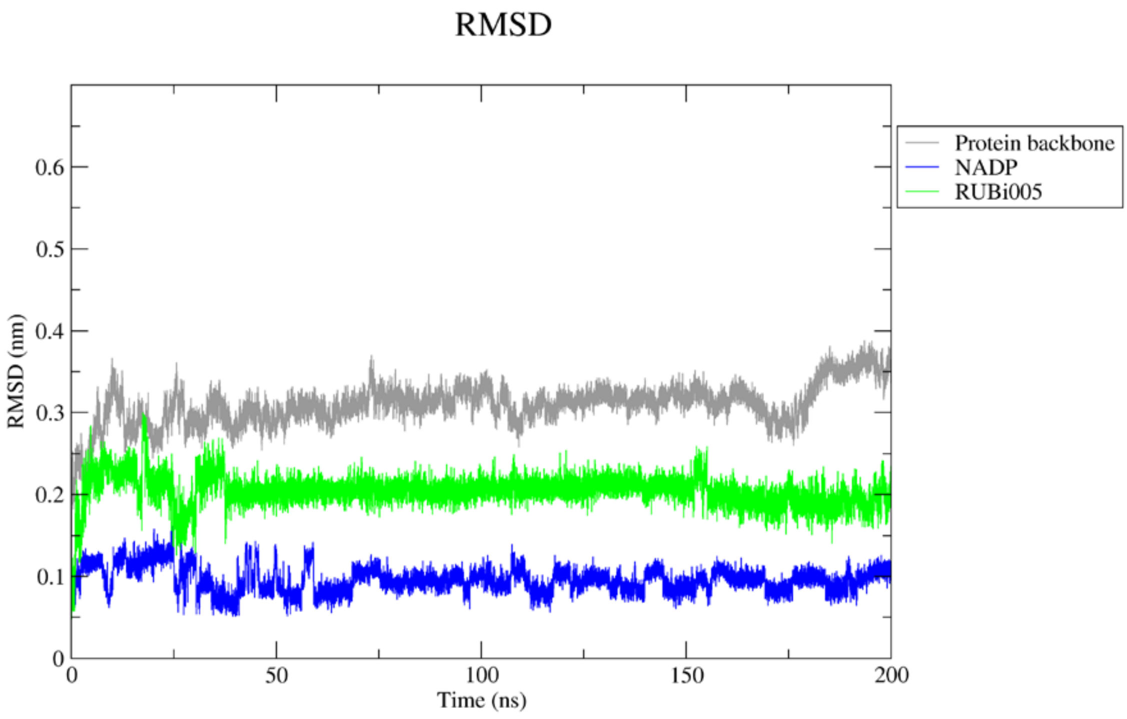

Figure 7F: Time dependent Root Mean Square Deviation (RMSD) of the backbone atoms of the apo protein, NADP, and ligand. The protein is coloured is coloured grey, the NADP cofactor coloured blue, and RUBi005 green.

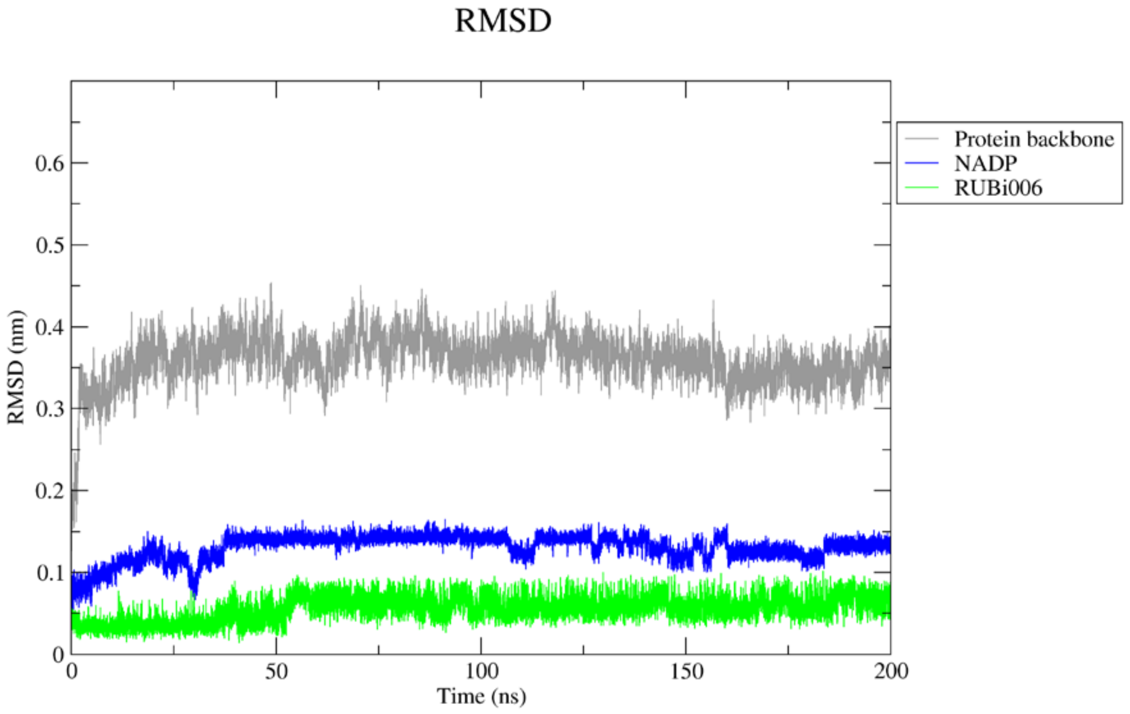

Figure 7G: Time dependent Root Mean Square Deviation (RMSD) of the backbone atoms of the apo protein, NADP, and ligand. The protein is coloured is coloured grey, the NADP cofactor coloured blue, and RUBi006 green.

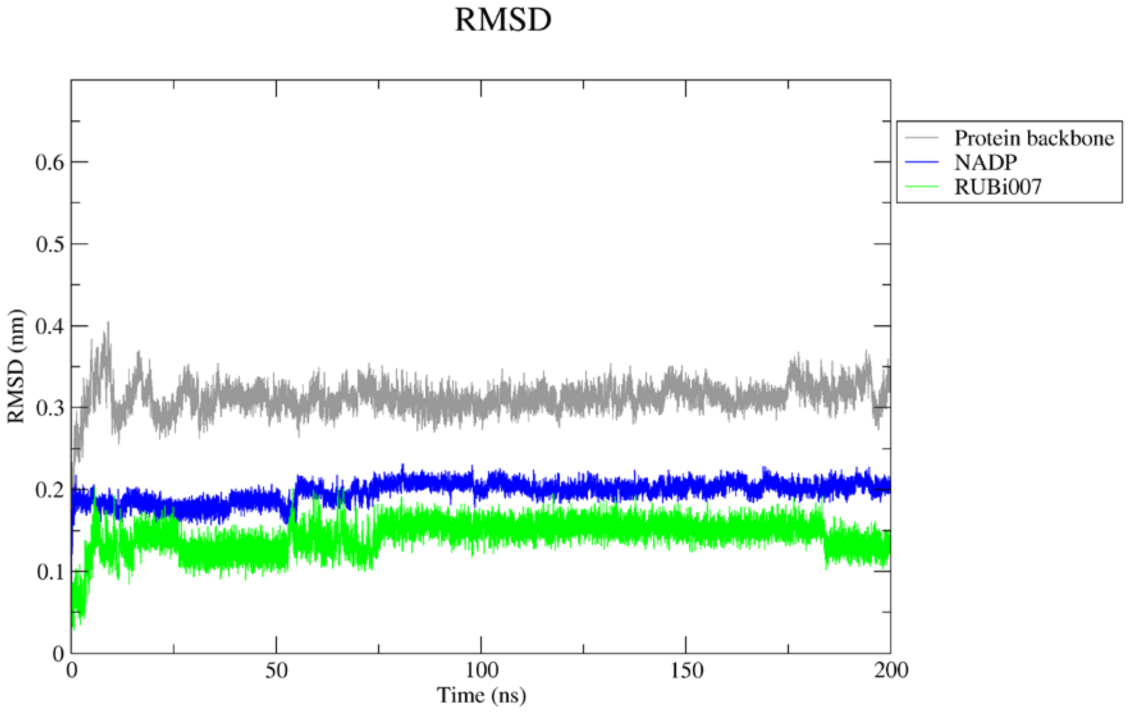

Figure 7H: Time dependent Root Mean Square Deviation (RMSD) of the backbone atoms of the apo protein, NADP, and ligand. The protein is coloured is coloured grey, the NADP cofactor coloured blue, and RUBi007 green.

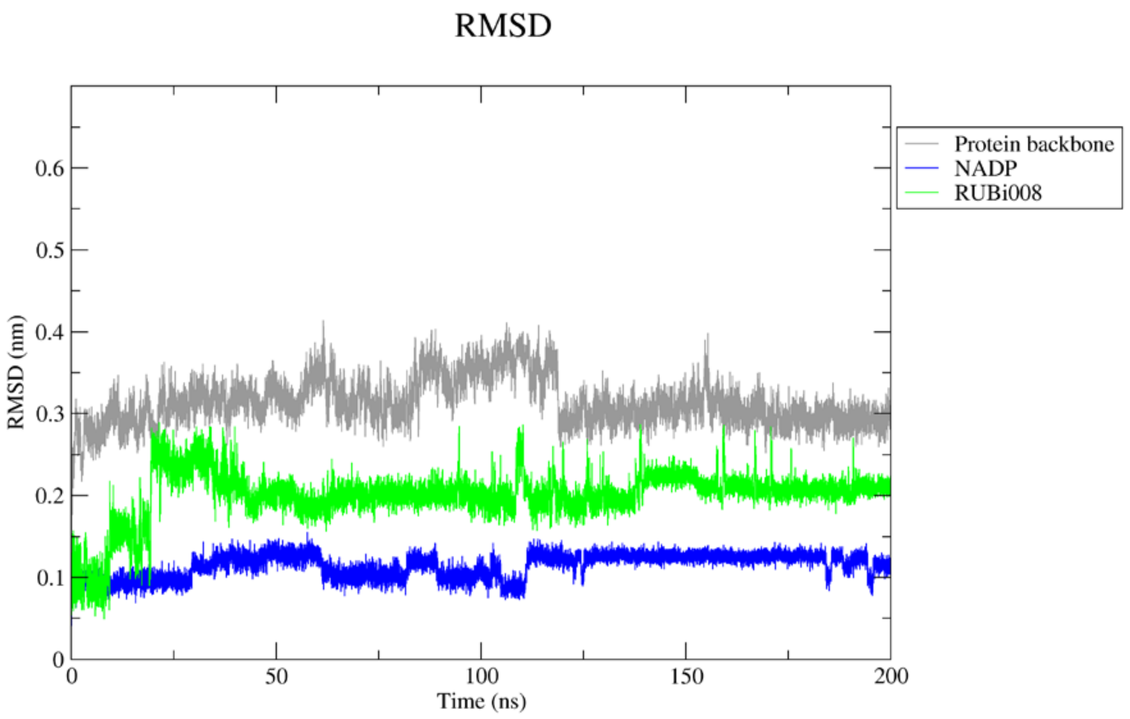

Figure 7J: Time dependent Root Mean Square Deviation (RMSD) of the backbone atoms of the apo protein, NADP, and ligand. The protein is coloured is coloured grey, the NADP cofactor coloured blue, and RUBi008 green.

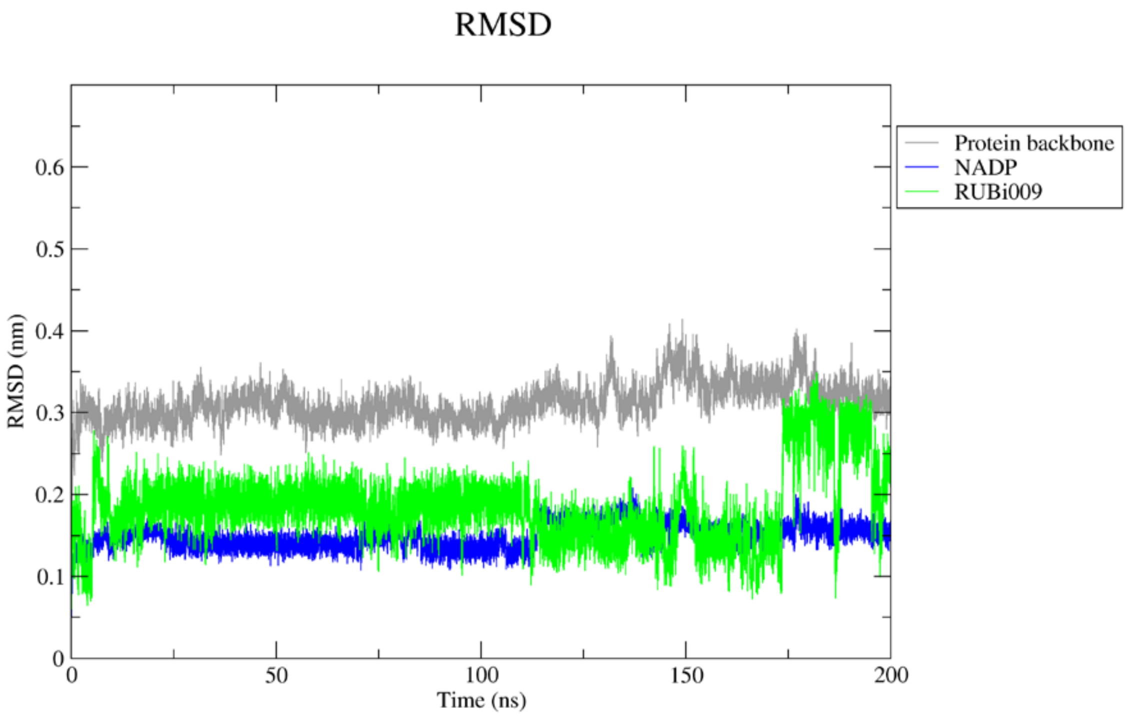

Figure 7K: Time dependent Root Mean Square Deviation (RMSD) of the backbone atoms of the apo protein, NADP, and ligand. The protein is coloured is coloured grey, the NADP cofactor coloured blue, and RUBi009 green.

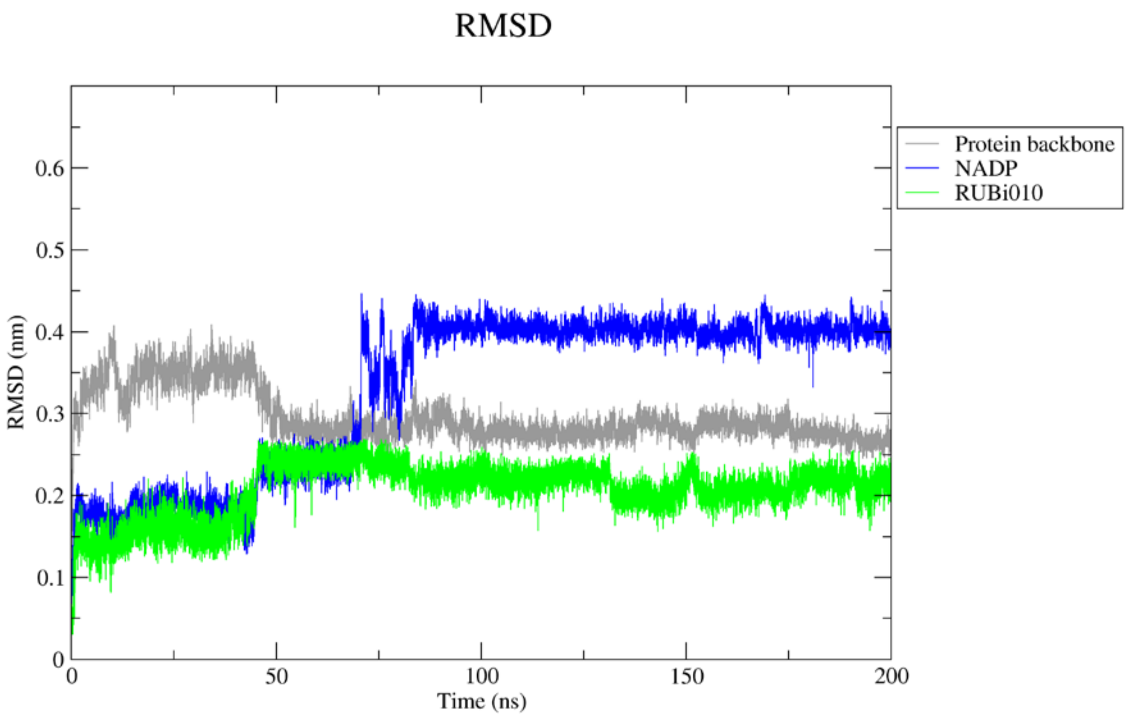

Figure 7L: Time dependent Root Mean Square Deviation (RMSD) of the backbone atoms of the apo protein, NADP, and ligand. The protein is coloured is coloured grey, the NADP cofactor coloured blue, and RUBi010 green.

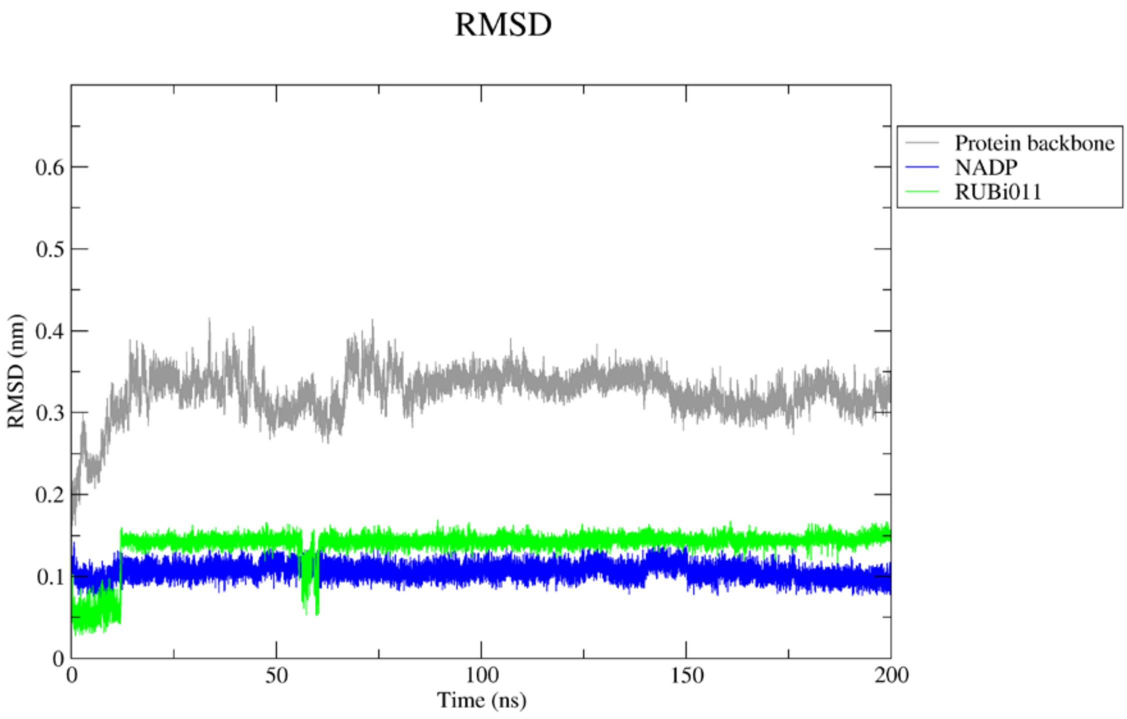

Figure 7M: Time dependent Root Mean Square Deviation (RMSD) of the backbone atoms of the apo protein, NADP, and ligand. The protein is coloured is coloured grey, the NADP cofactor coloured blue, and RUBi011 green.

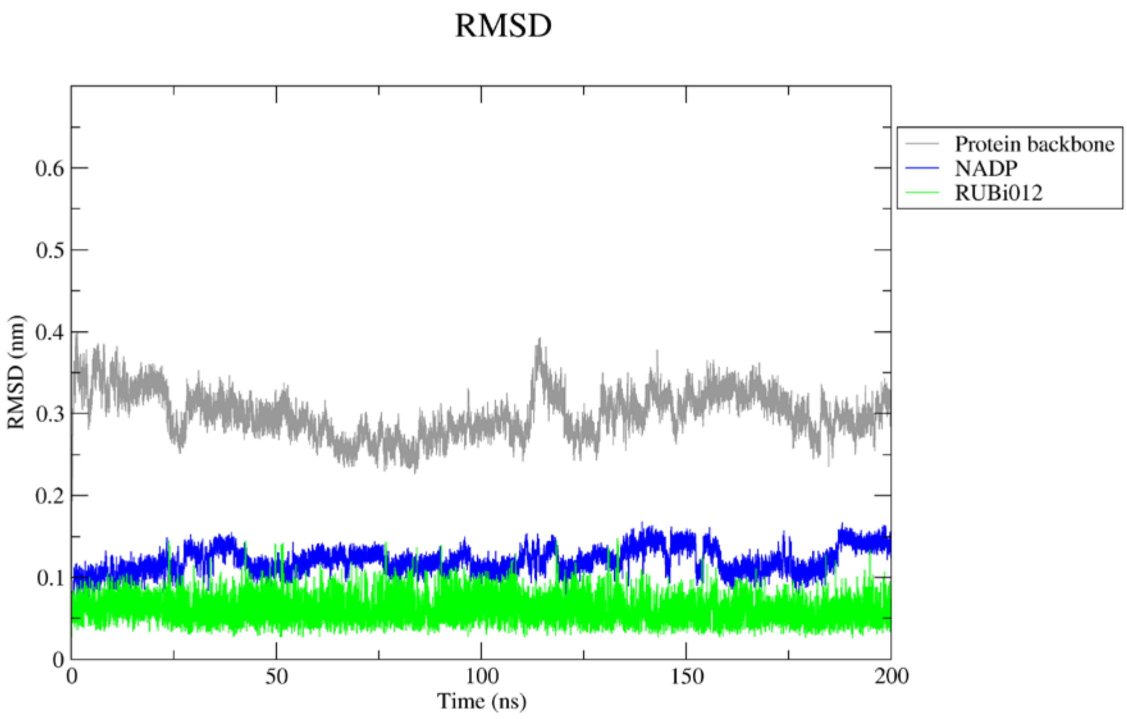

Figure 7N: Time dependent Root Mean Square Deviation (RMSD) of the backbone atoms of the apo protein, NADP, and ligand. The protein is coloured is coloured grey, the NADP cofactor coloured blue, and RUBi012 green.

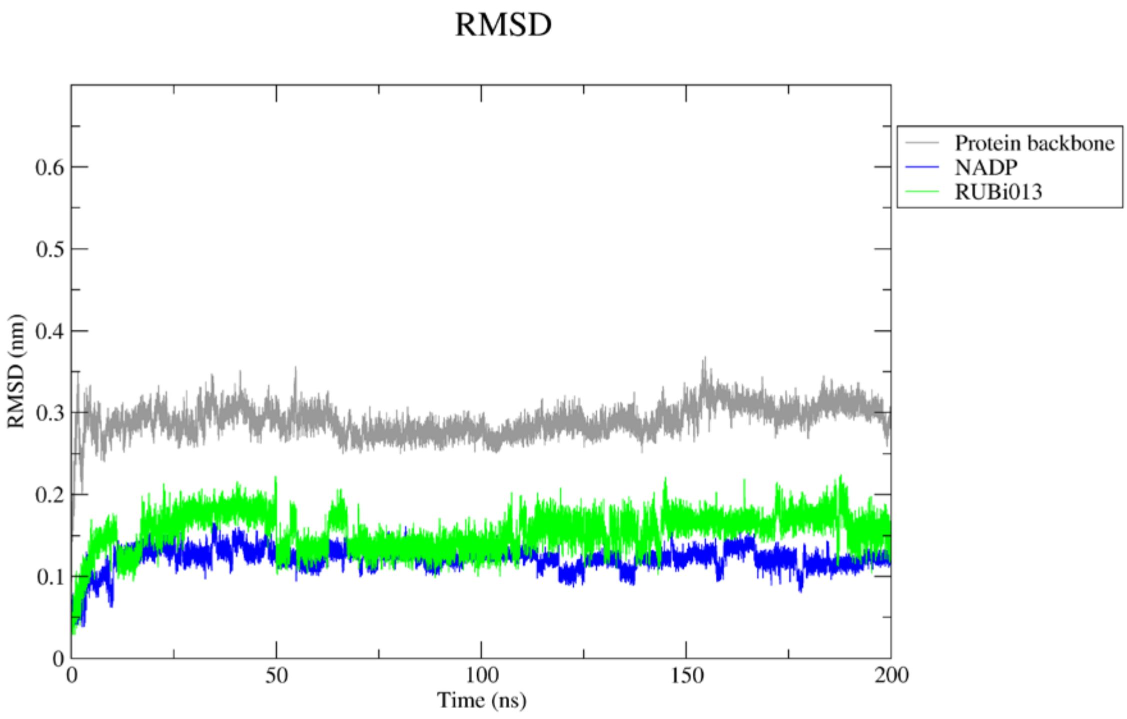

Figure 7O: Time dependent Root Mean Square Deviation (RMSD) of the backbone atoms of the apo protein, NADP, and ligand. The protein is coloured is coloured grey, the NADP cofactor coloured blue, and RUBi013 green.

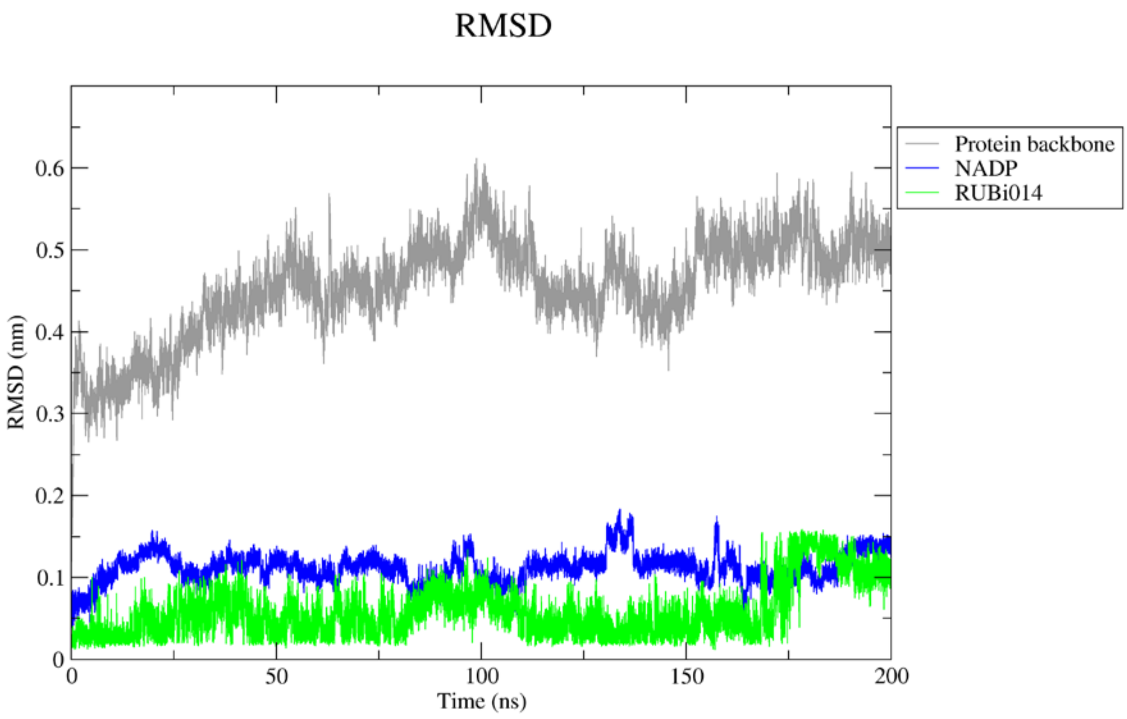

Figure 7P: Time dependent Root Mean Square Deviation (RMSD) of the backbone atoms of the apo protein, NADP, and ligand. The protein is coloured is coloured grey, the NADP cofactor coloured blue, and RUBi014 green.

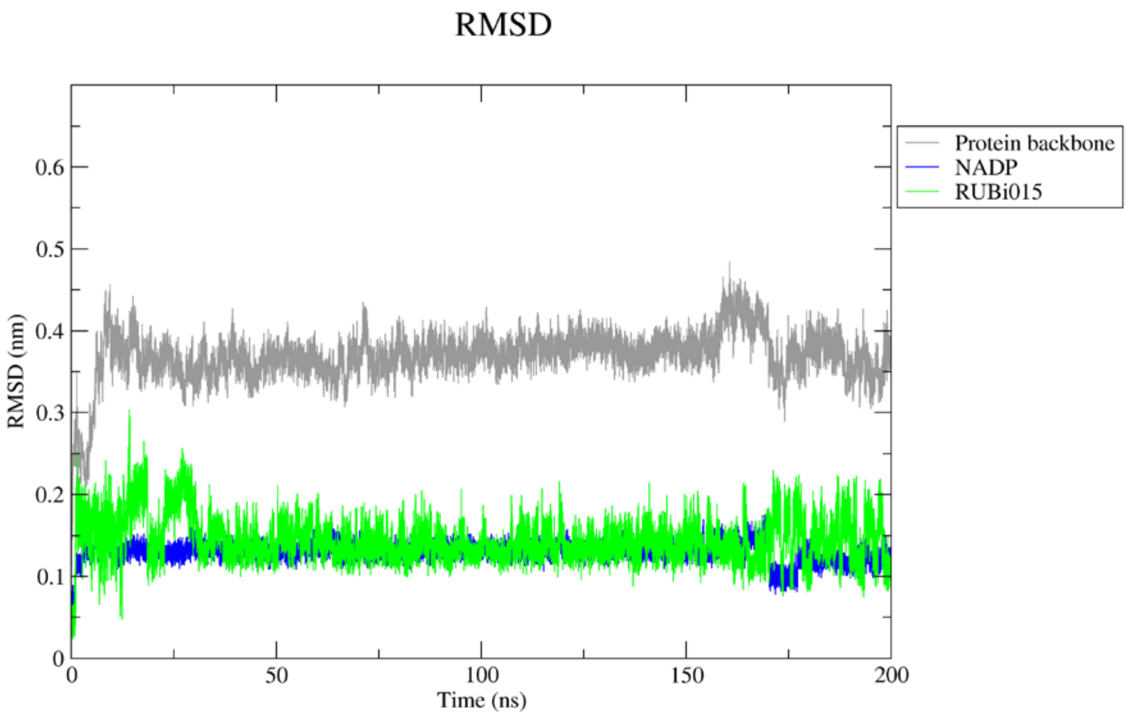

Figure 7R: Time dependent Root Mean Square Deviation (RMSD) of the backbone atoms of the apo protein, NADP, and ligand. The protein is coloured is coloured grey, the NADP cofactor coloured blue, and RUBi015 green.

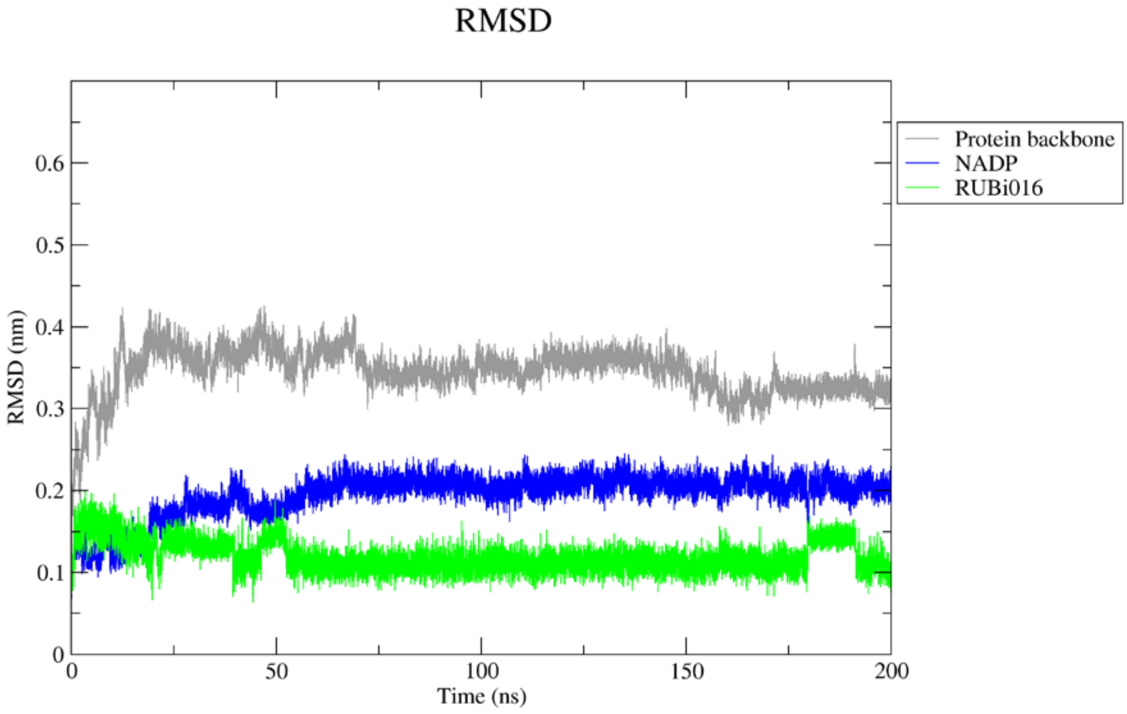

Figure 7S: Time dependent Root Mean Square Deviation (RMSD) of the backbone atoms of the apo protein, NADP, and ligand. The protein is coloured is coloured grey, the NADP cofactor coloured blue, and RUBi016 green.

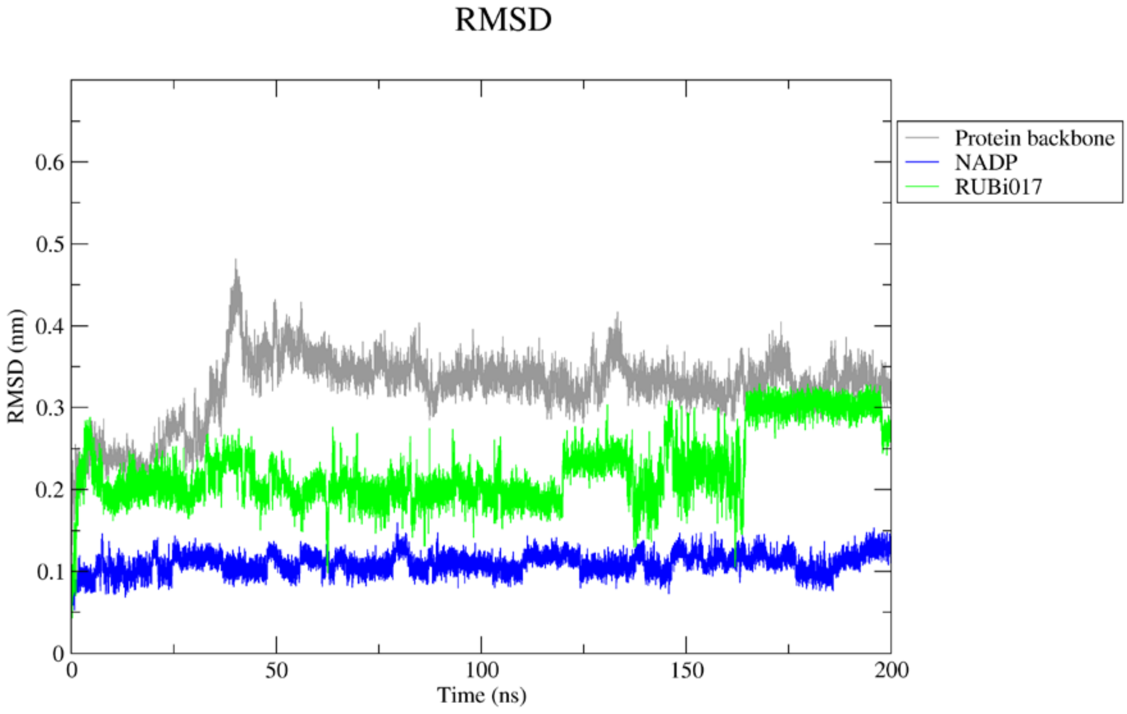

Figure 7T: Time dependent Root Mean Square Deviation (RMSD) of the backbone atoms of the apo protein, NADP, and ligand. The protein is coloured is coloured grey, the NADP cofactor coloured blue, and RUBi017 green.

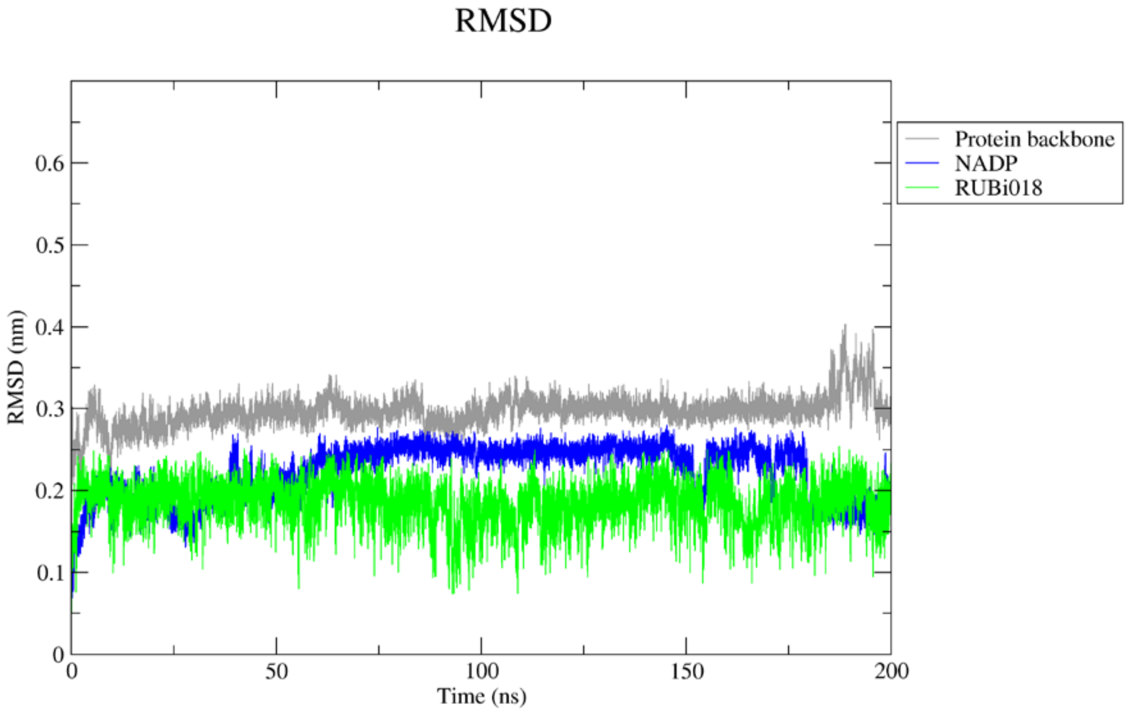

Figure 7U: Time dependent Root Mean Square Deviation (RMSD) of the backbone atoms of the apo protein, NADP cofactor and ligand. The protein is coloured is coloured grey, the NADP cofactor coloured blue, and RUBi018 green.

Supplementary Figure S8: Rg values of 18 *Tb*PTR1-ligand complexes compared to apo protein

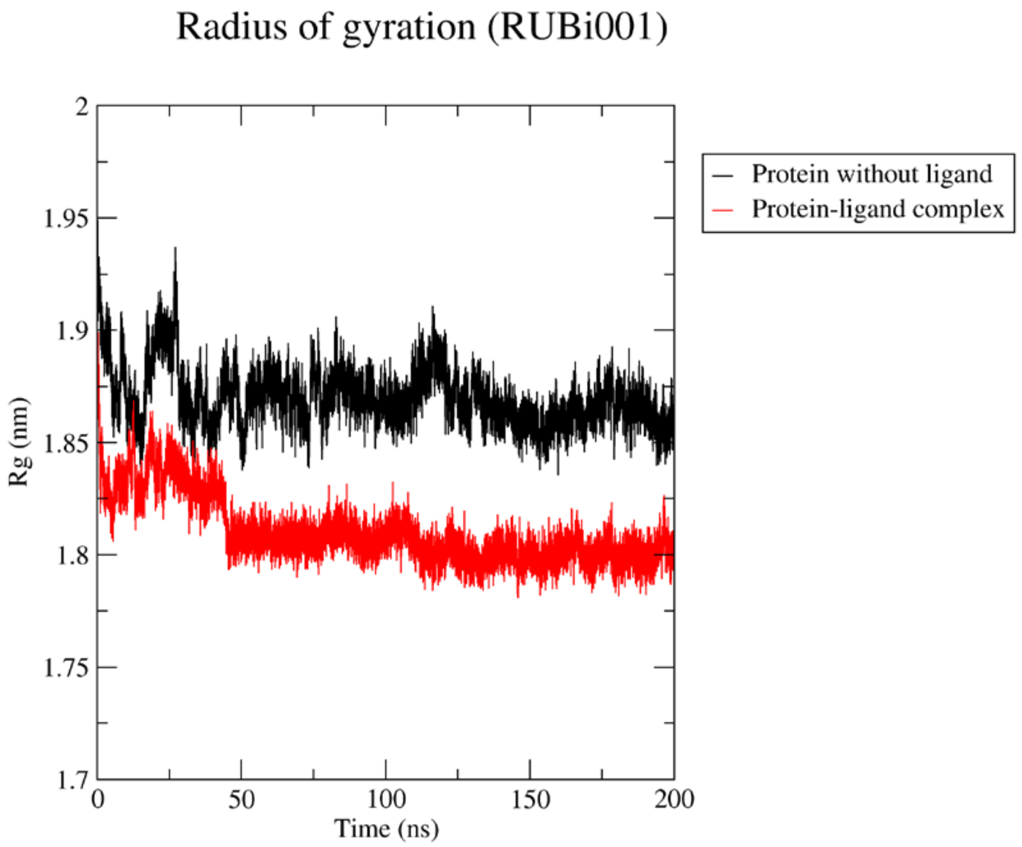

Figure 8A: The *Tb*PTR1 radius of gyration (Rg) of based on C-alpha atoms versus time at 300K. The protein without the ligand is colored black while the *Tb*PTR1-RUBi001 complex is colored red.

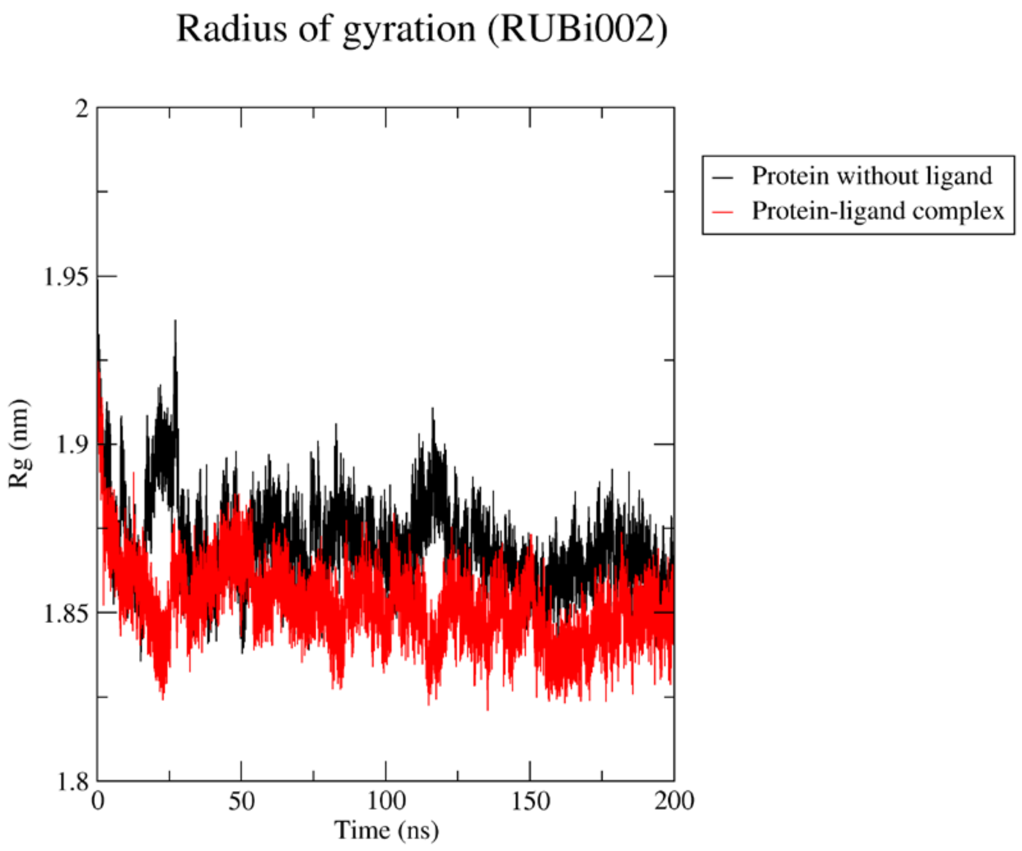

Figure 8B: The *Tb*PTR1 radius of gyration (Rg) of based on C-alpha atoms versus time at 300K. The protein without the ligand is colored black while the *Tb*PTR1-RUBi002 complex is colored red.

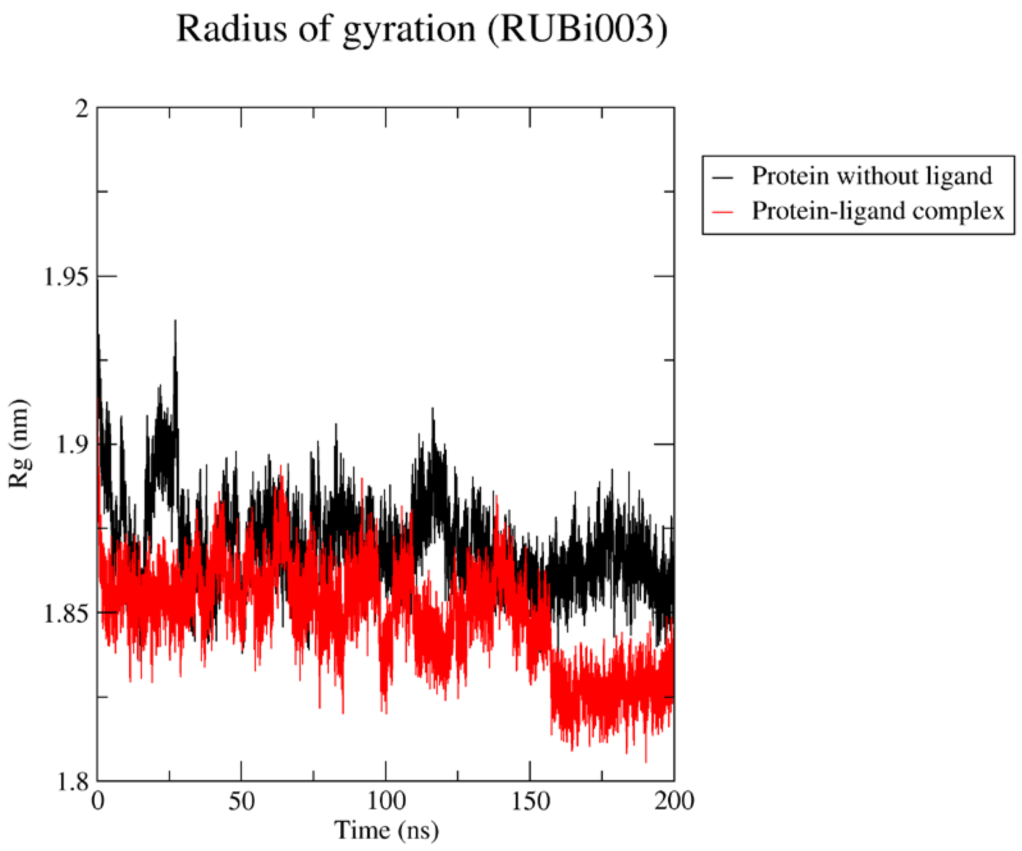

Figure 8C: The *Tb*PTR1 radius of gyration (Rg) of based on C-alpha atoms versus time at 300K. The protein without the ligand is colored black while the *Tb*PTR1-RUBi003 complex is colored red.

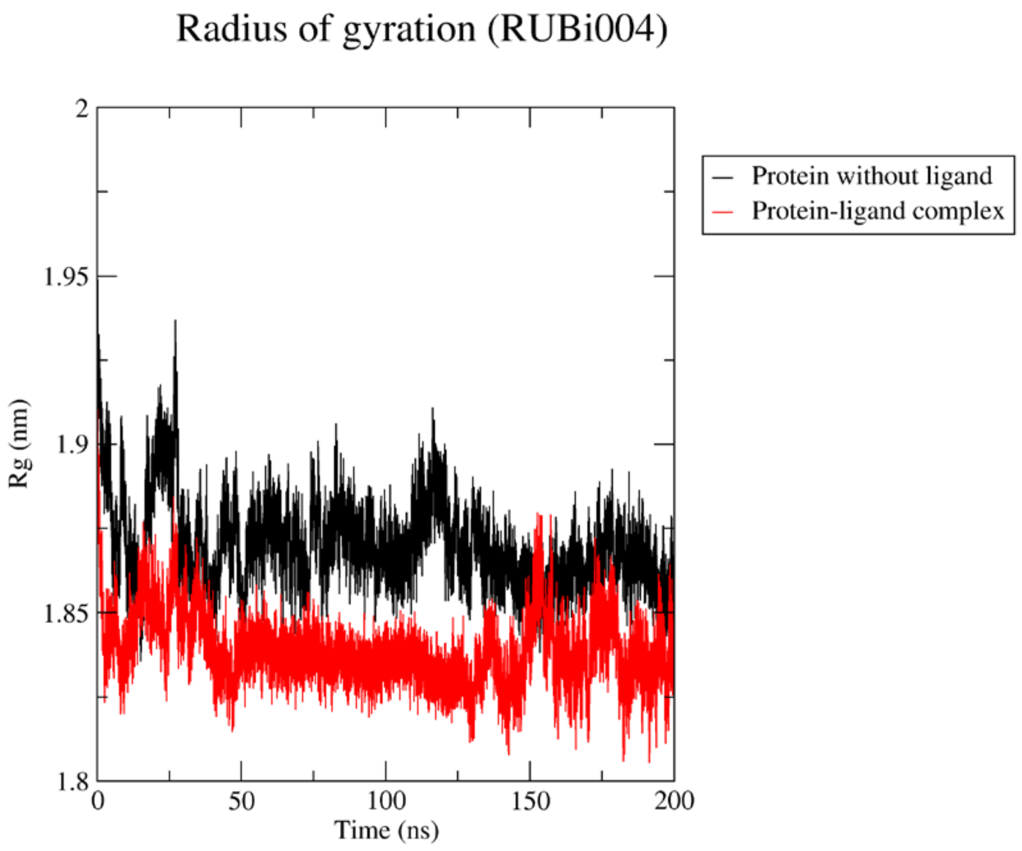

Figure 8D: The *Tb*PTR1 radius of gyration (Rg) of based on C-alpha atoms versus time at 300K. The protein without the ligand is colored black while the *Tb*PTR1-RUBi004 complex is colored red.

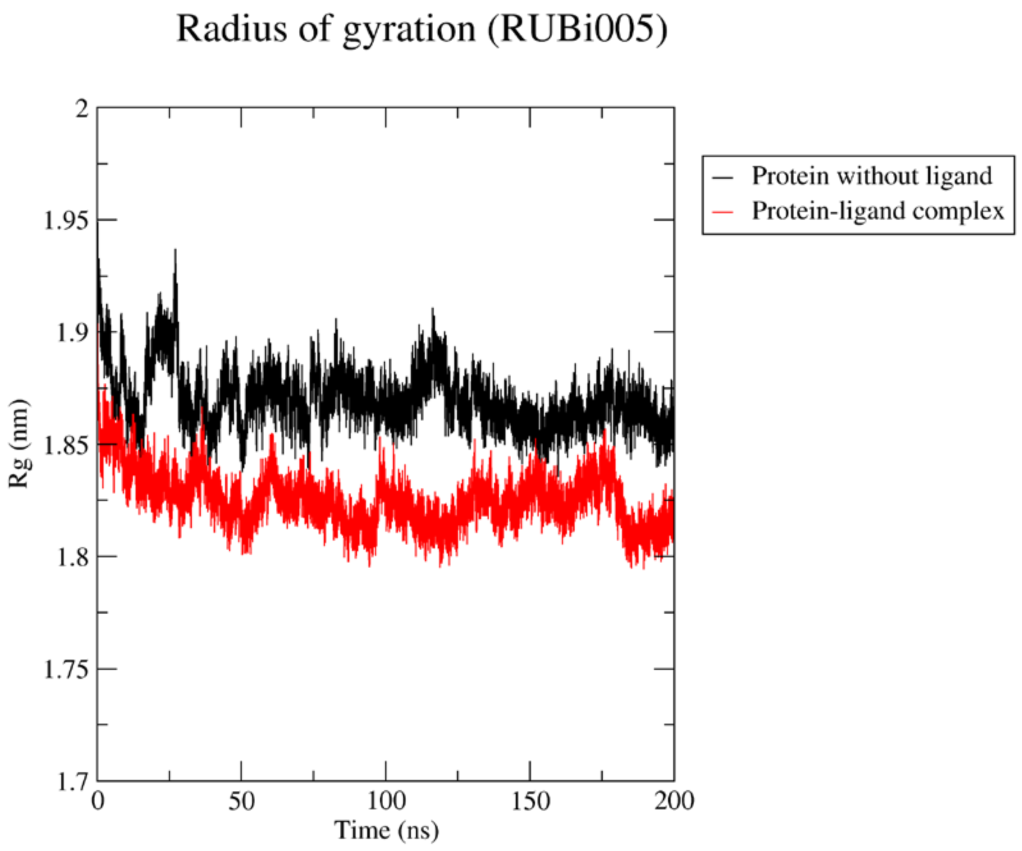

Figure 8E: The *Tb*PTR1 radius of gyration (Rg) of based on C-alpha atoms versus time at 300K. The protein without the ligand is colored black while the *Tb*PTR1-RUBi005 complex is colored red.

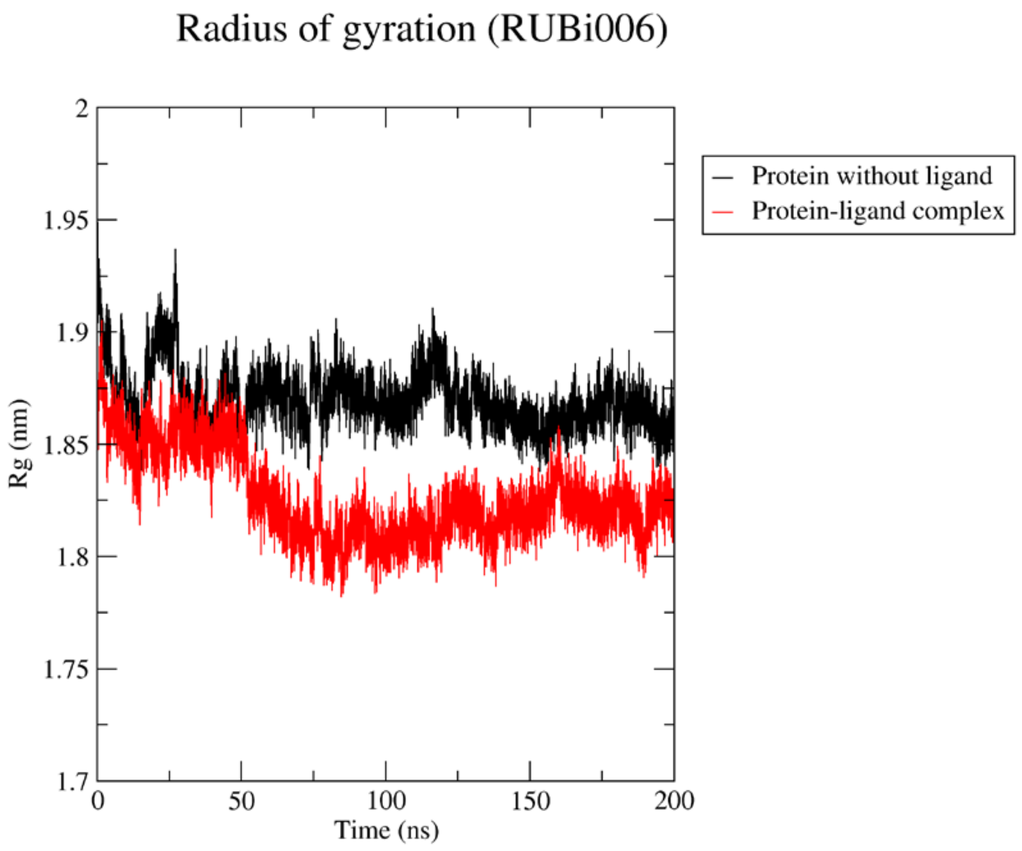

Figure 8F: The *Tb*PTR1 radius of gyration (Rg) of based on C-alpha atoms versus time at 300K. The protein without the ligand is colored black while the *Tb*PTR1-RUBi006 complex is colored red.

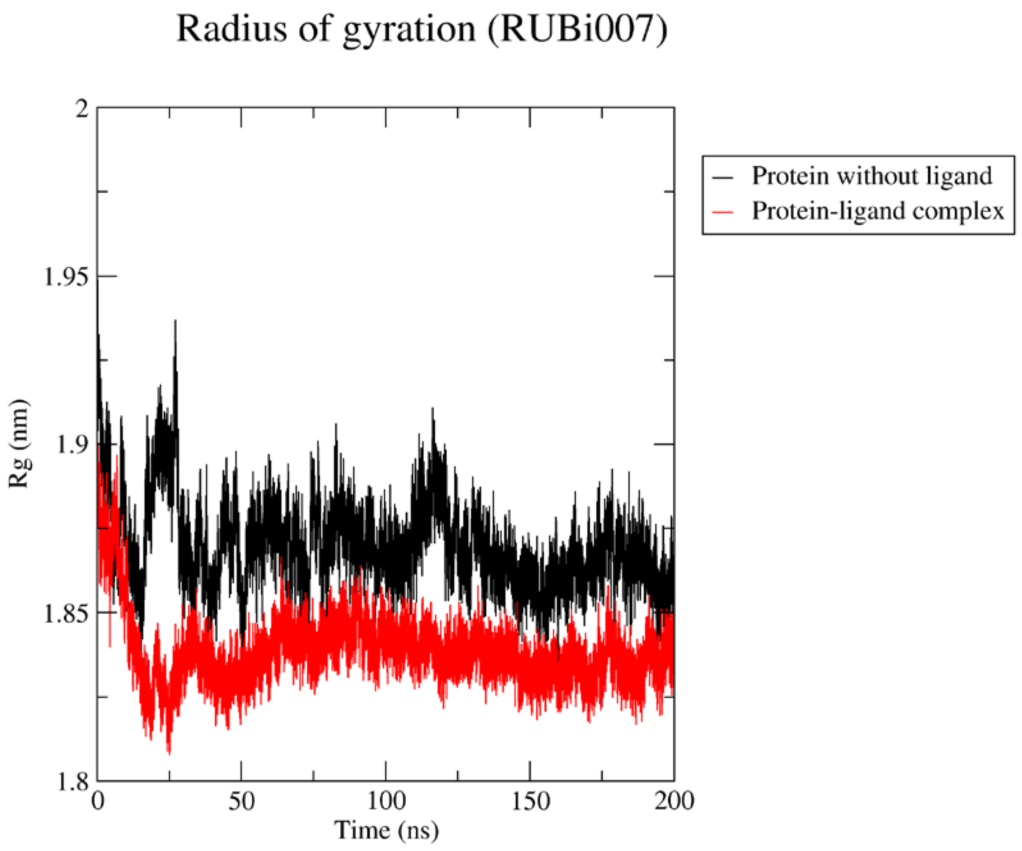

Figure 8G: The *Tb*PTR1 radius of gyration (Rg) of based on C-alpha atoms versus time at 300K. The protein without the ligand is colored black while the *Tb*PTR1-RUBi007 complex is colored red.

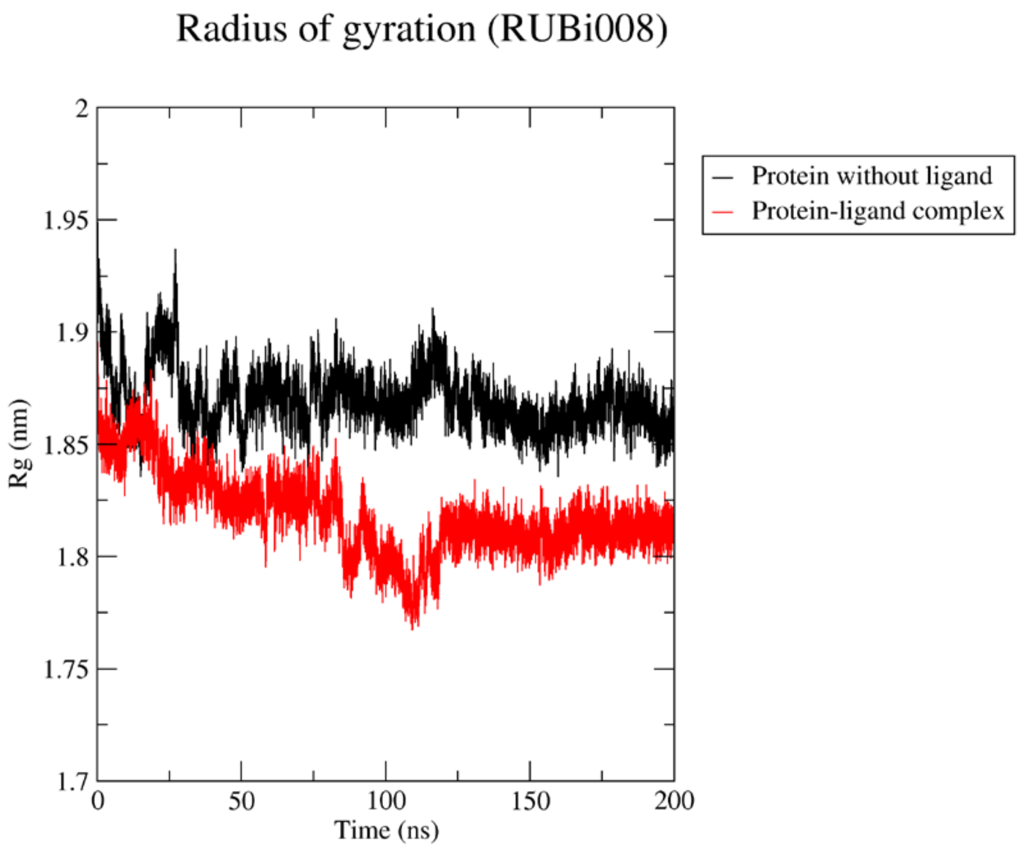

Figure 8H: The *Tb*PTR1 radius of gyration (Rg) of based on C-alpha atoms versus time at 300K. The protein without the ligand is colored black while the *Tb*PTR1-RUBi008 complex is colored red.

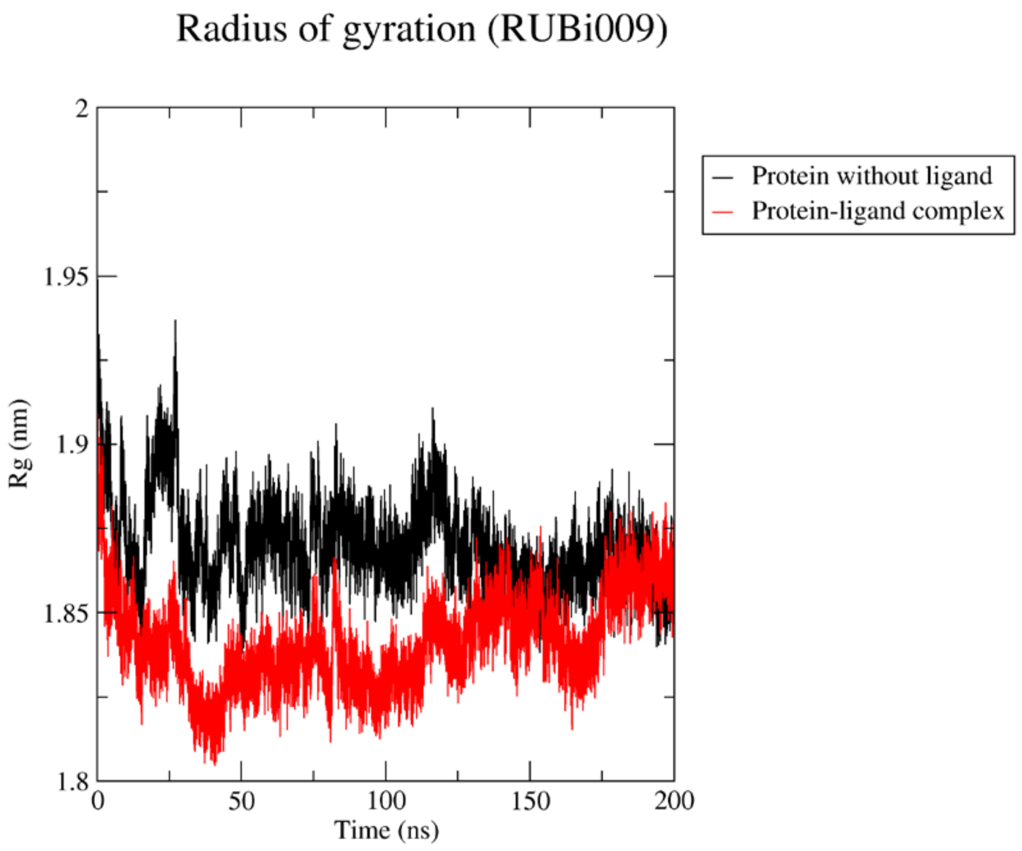

Figure 8I: The *Tb*PTR1 radius of gyration (Rg) of based on C-alpha atoms versus time at 300K. The protein without the ligand is colored black while the *Tb*PTR1-RUBi009 complex is colored red.

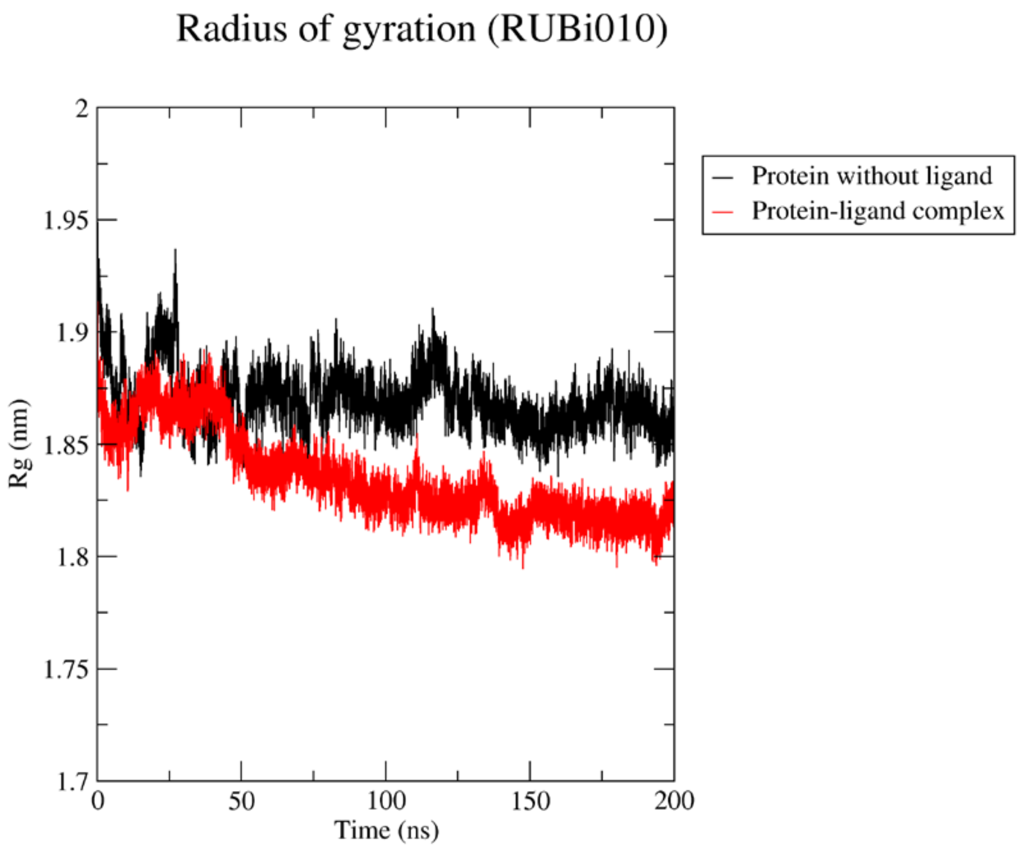

Figure 8J: The *Tb*PTR1 radius of gyration (Rg) of based on C-alpha atoms versus time at 300K. The protein without the ligand is colored black while the *Tb*PTR1-RUBi010 complex is colored red.

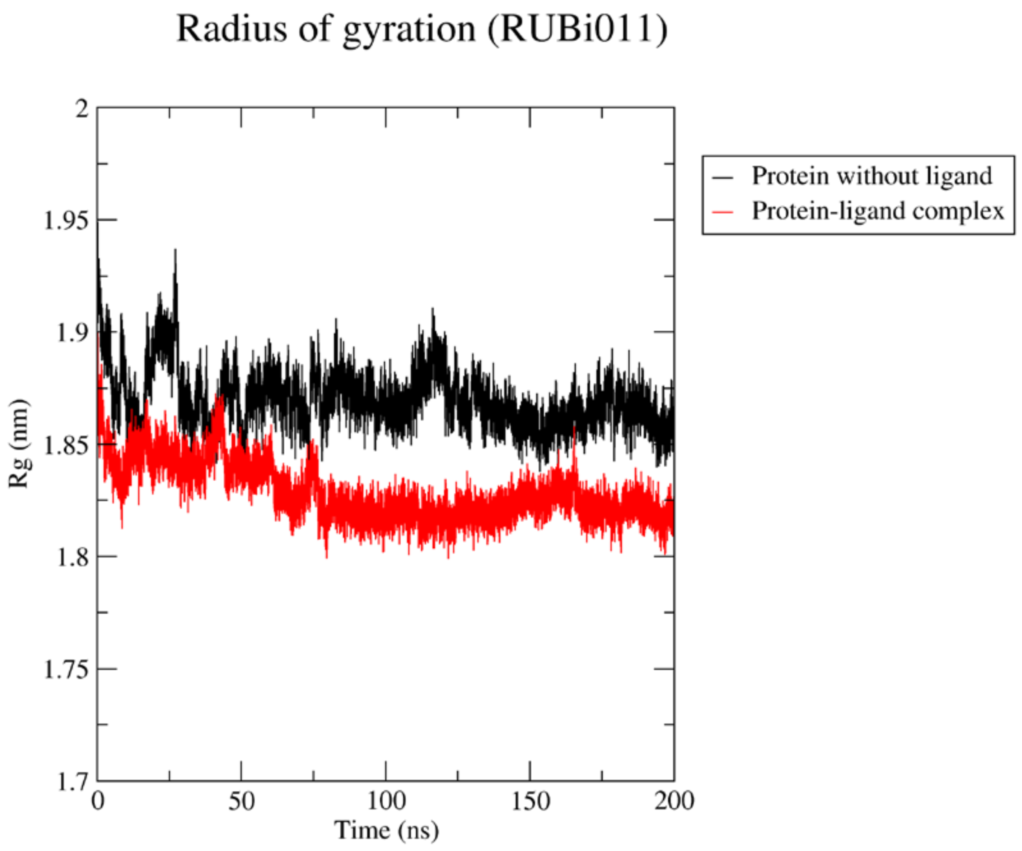

Figure 8K: The *Tb*PTR1 radius of gyration (Rg) of based on C-alpha atoms versus time at 300K. The protein without the ligand is colored black while the *Tb*PTR1-RUBi011 complex is colored red.

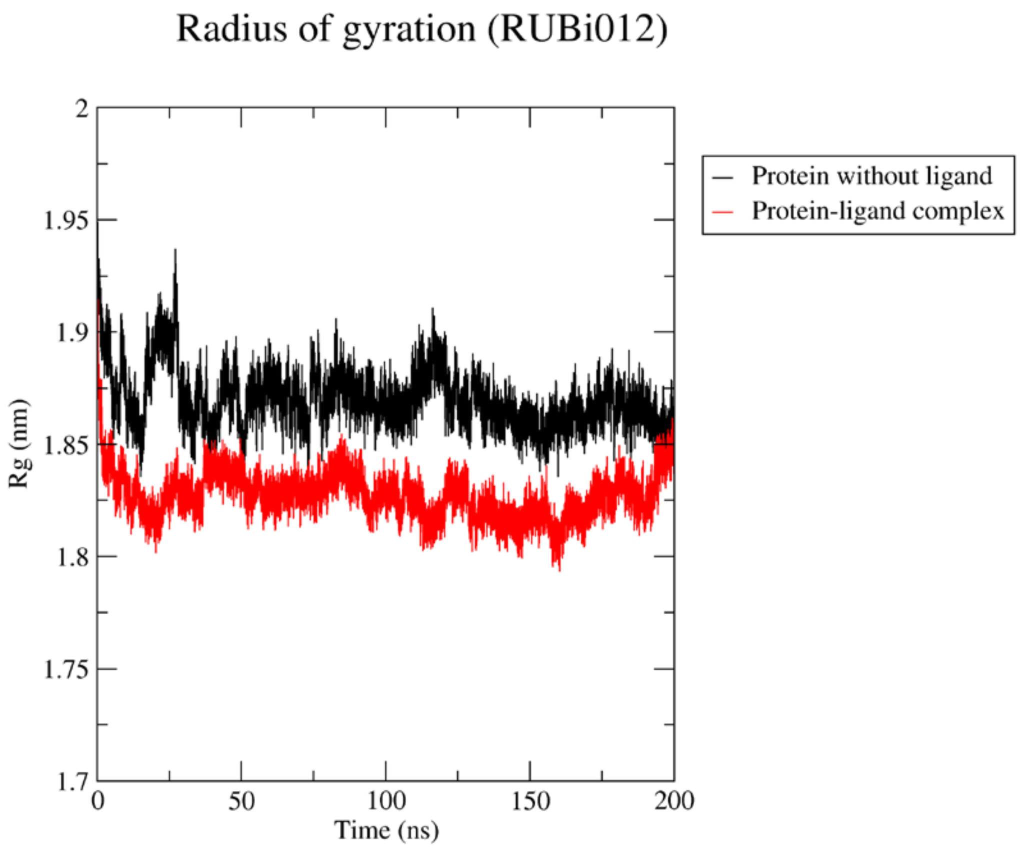

Figure 8L: The *Tb*PTR1 radius of gyration (Rg) of based on C-alpha atoms versus time at 300K. The protein without the ligand is colored black while the *Tb*PTR1-RUBi012 complex is colored red.

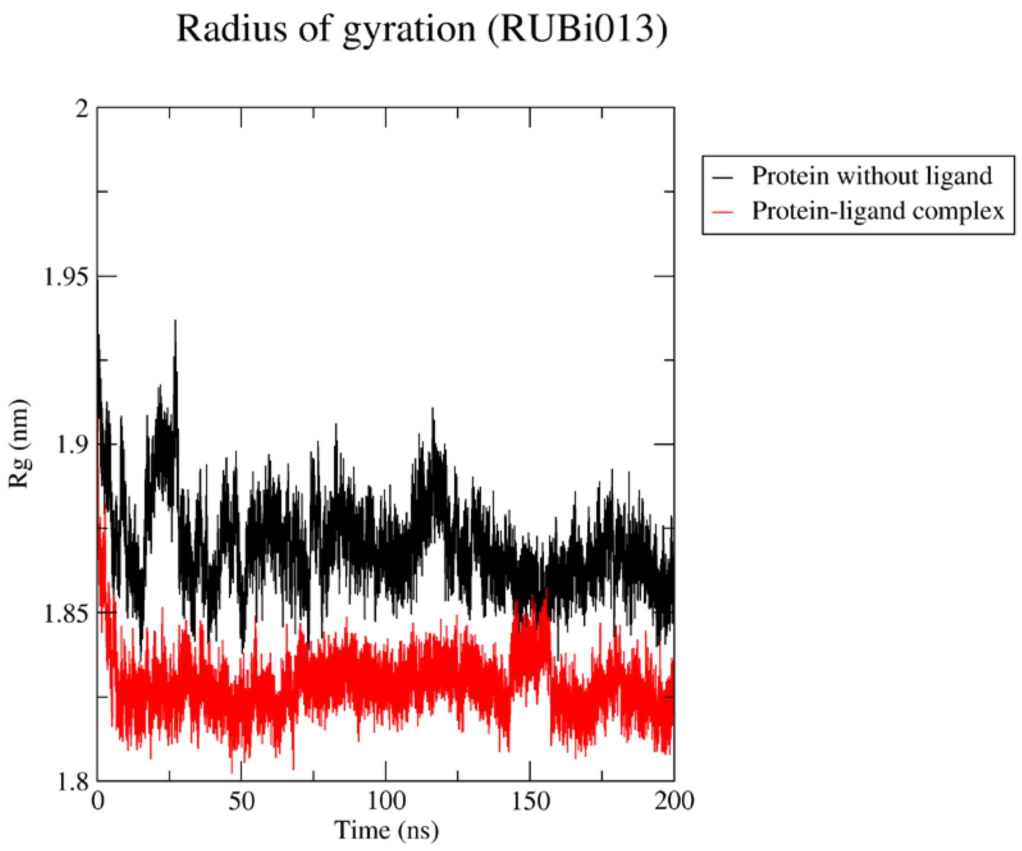

Figure 8M: The *Tb*PTR1 radius of gyration (Rg) of based on C-alpha atoms versus time at 300K. The protein without the ligand is colored black while the *Tb*PTR1-RUBi013 complex is colored red.

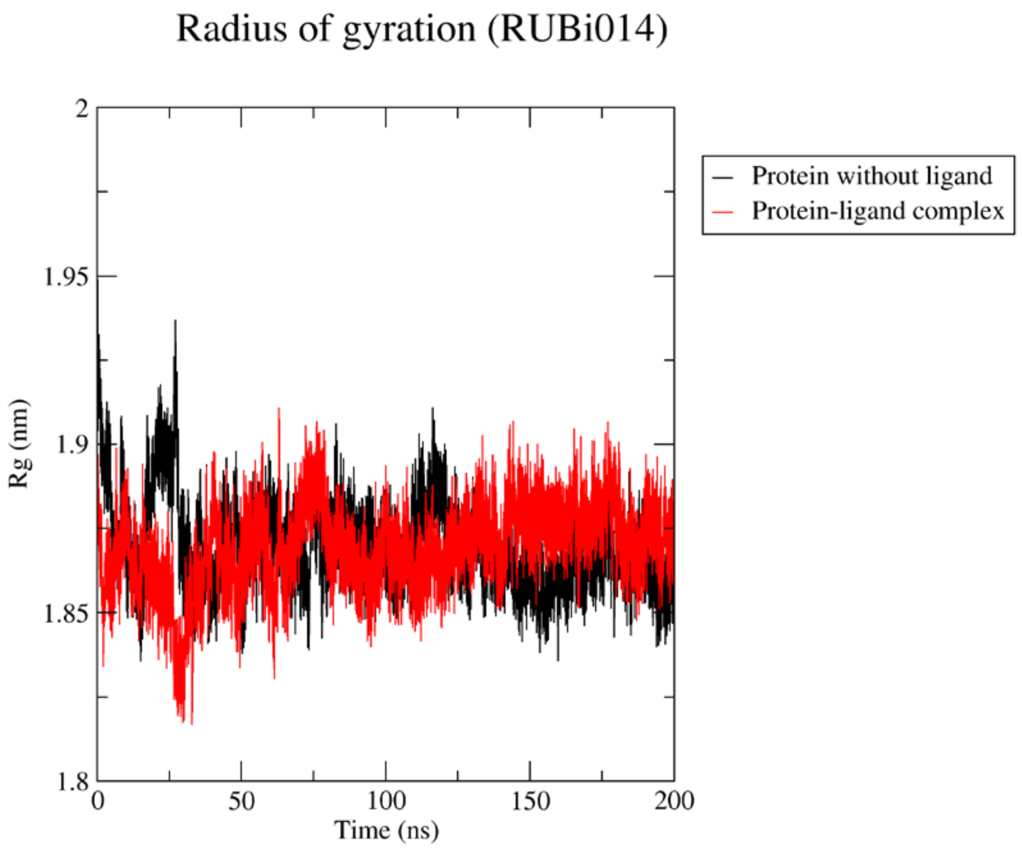

Figure 8N: The *Tb*PTR1 radius of gyration (Rg) of based on C-alpha atoms versus time at 300K. The protein without the ligand is colored black while the *Tb*PTR1-RUBi014 complex is colored red.

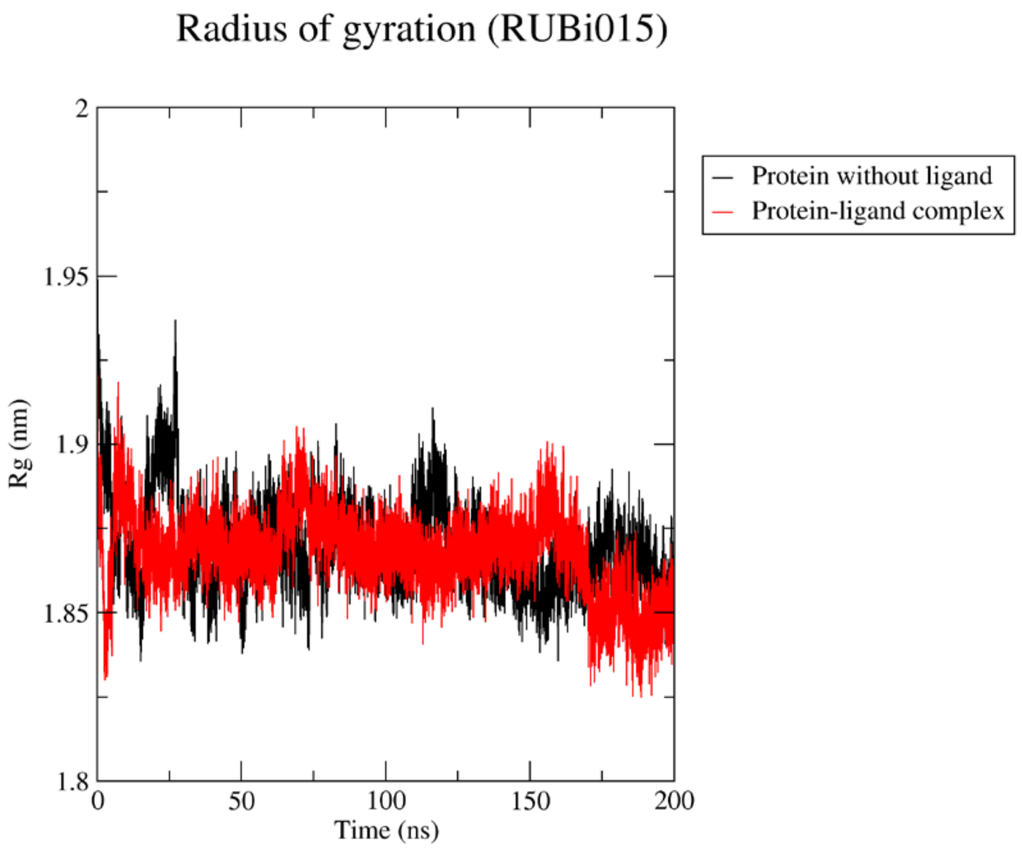

Figure 8O: The *Tb*PTR1 radius of gyration (Rg) of based on C-alpha atoms versus time at 300K. The protein without the ligand is colored black while the *Tb*PTR1-RUBi015 complex is colored red.

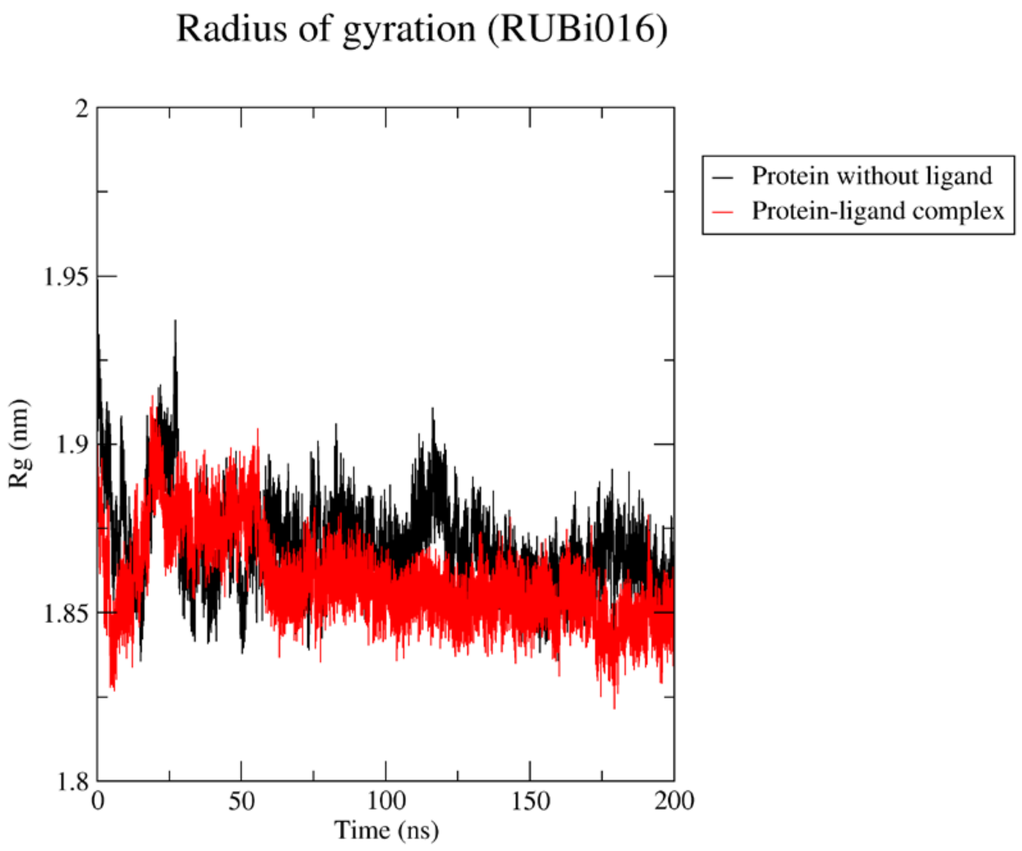

Figure 8P: The *Tb*PTR1 radius of gyration (Rg) of based on C-alpha atoms versus time at 300K. The protein without the ligand is colored black while the *Tb*PTR1-RUBi016 complex is colored red.

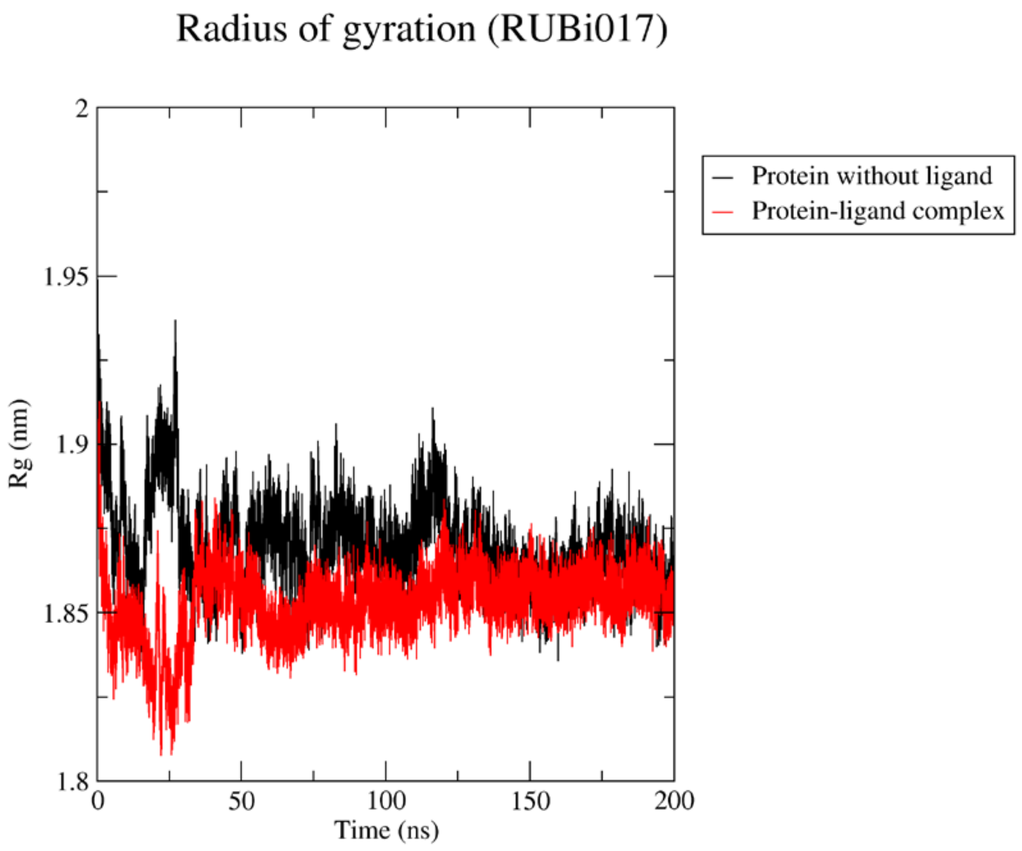

Figure 8R: The *Tb*PTR1 radius of gyration (Rg) of based on C-alpha atoms versus time at 300K. The protein without the ligand is colored black while the *Tb*PTR1-RUBi017 complex is colored red.

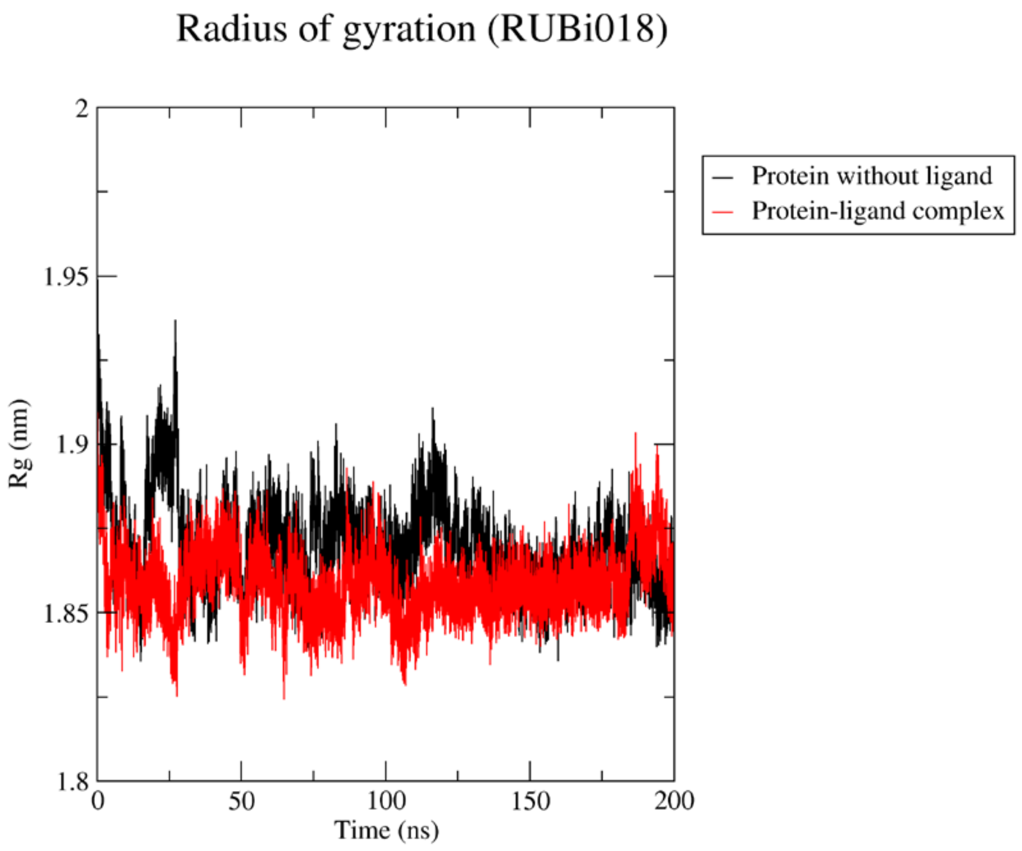

Figure 8S: The *Tb*PTR1 radius of gyration (Rg) of based on C-alpha atoms versus time at 300K. The protein without the ligand is colored black while the *Tb*PTR1-RUBi018 complex is colored red.

Supplementary Figure S9: The time evolution of the number of intermolecular hydrogen bonds formed between the compounds and *Tb*PTR1 protein during simulation

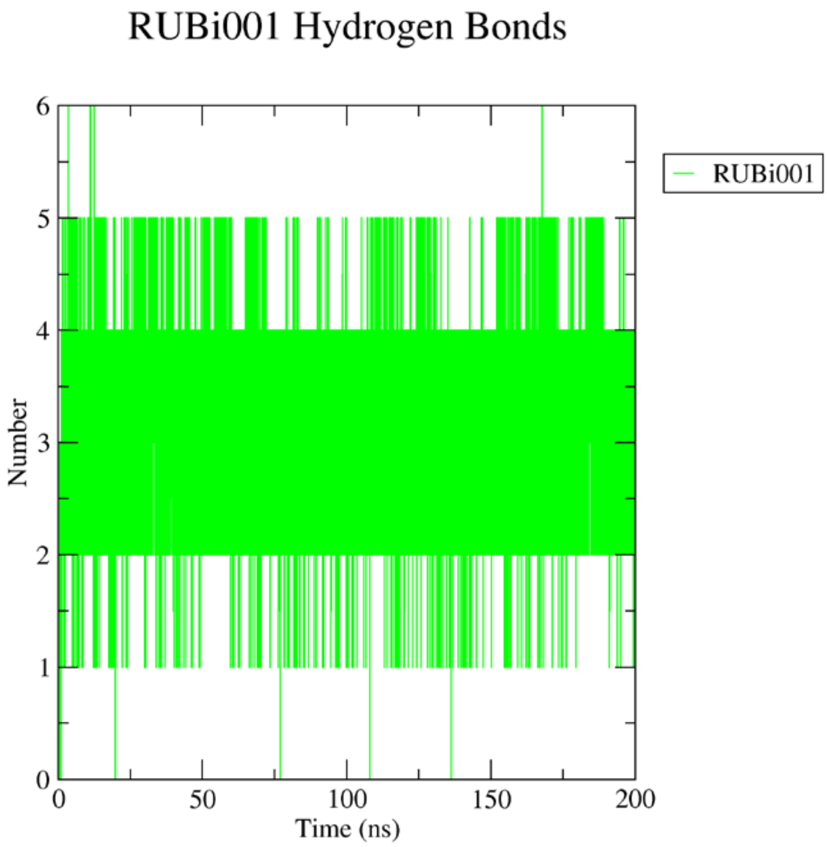

Figure 9A: The time evolution of the number of intermolecular hydrogen bonds formed between the RUBi001 and *Tb*PTR1 protein in the *Tb*PTR1-RUBi001 complex

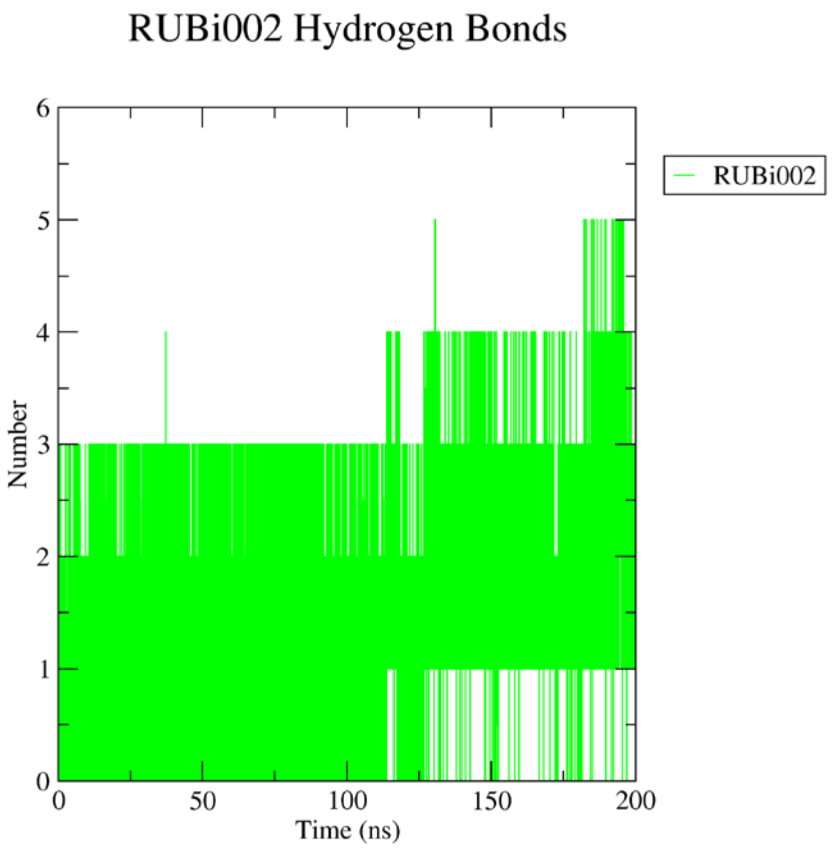

Figure 9B: The time evolution of the number of intermolecular hydrogen bonds formed between the RUBi002 and *Tb*PTR1 protein in the *Tb*PTR1-RUBi002 complex

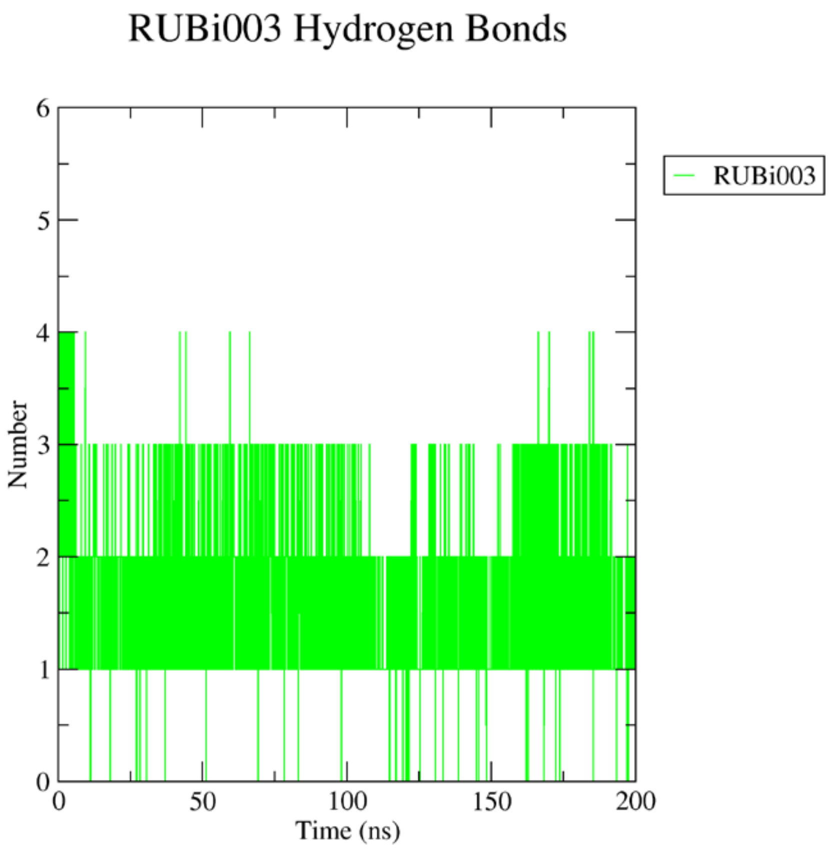

Figure 9C: The time evolution of the number of intermolecular hydrogen bonds formed between the RUBi003 and *Tb*PTR1 protein in the *Tb*PTR1-RUBi003 complex

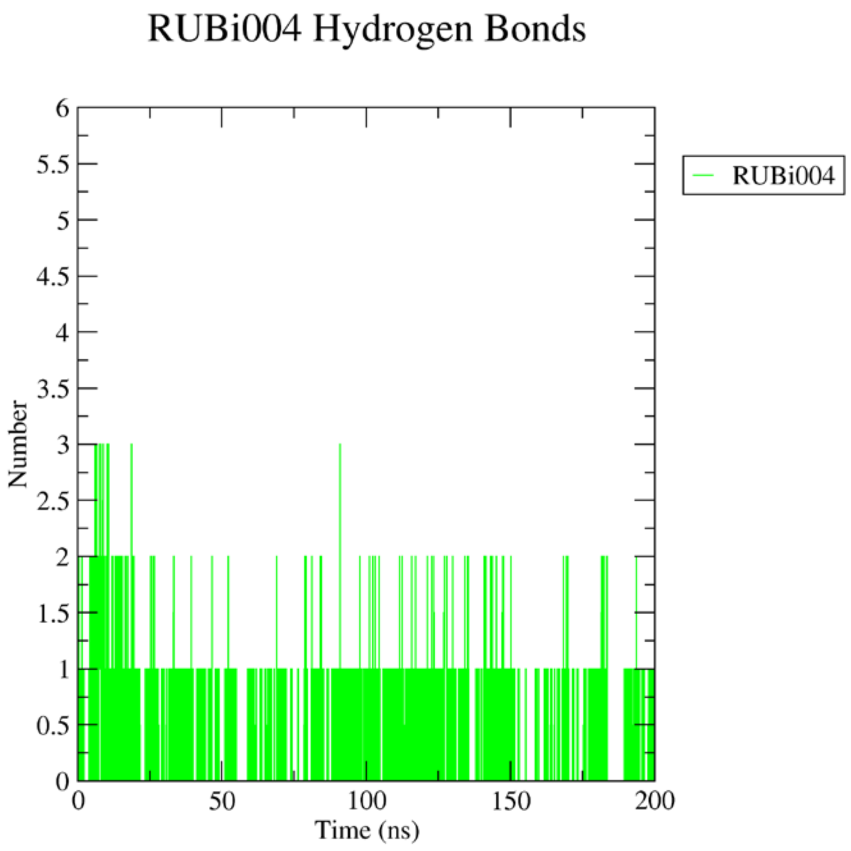

Figure 9D: The time evolution of the number of intermolecular hydrogen bonds formed between the RUBi004 and *Tb*PTR1 protein in the *Tb*PTR1-RUBi004 complex

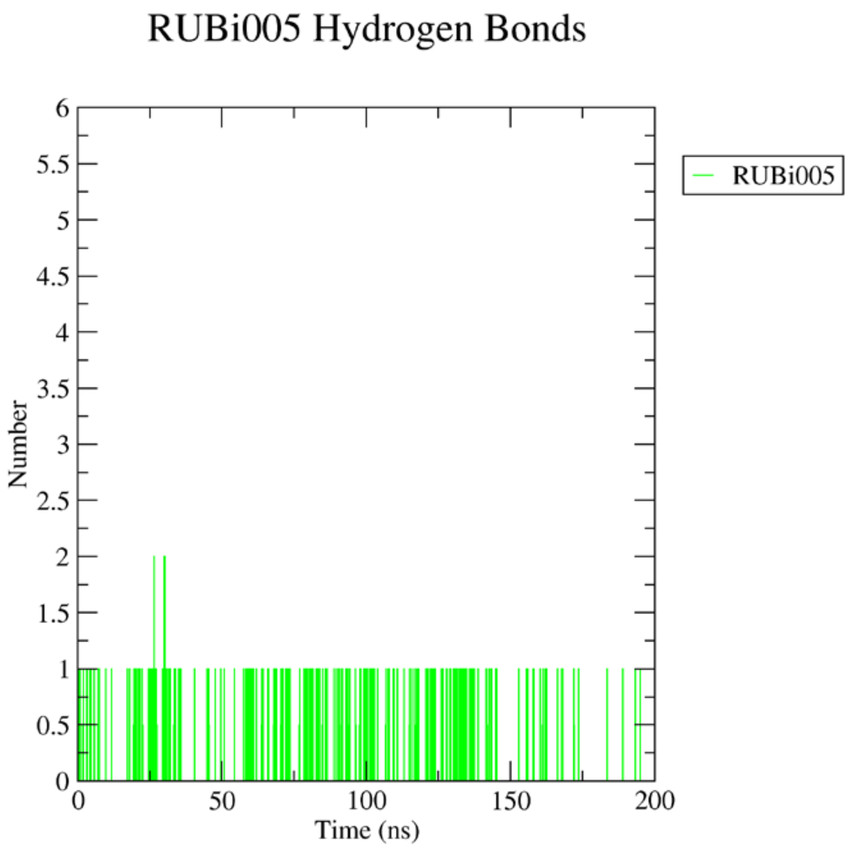

Figure 9E: The time evolution of the number of intermolecular hydrogen bonds formed between the RUBi005 and *Tb*PTR1 protein in the *Tb*PTR1-RUBi005 complex

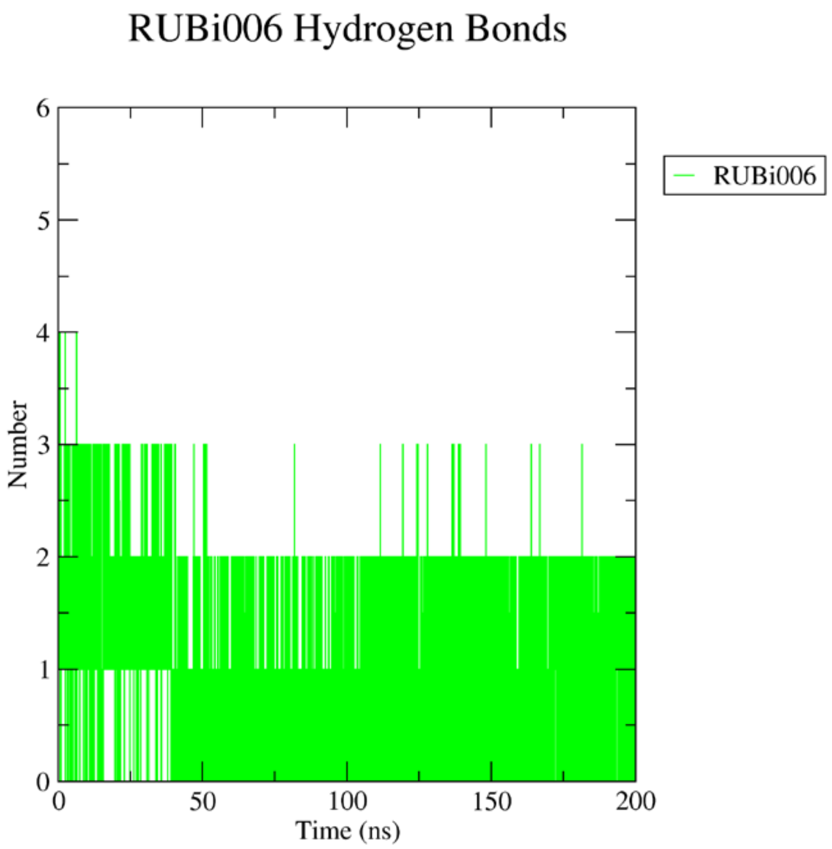

Figure 9F: The time evolution of the number of intermolecular hydrogen bonds formed between the RUBi006 and *Tb*PTR1 protein in the *Tb*PTR1-RUBi006 complex

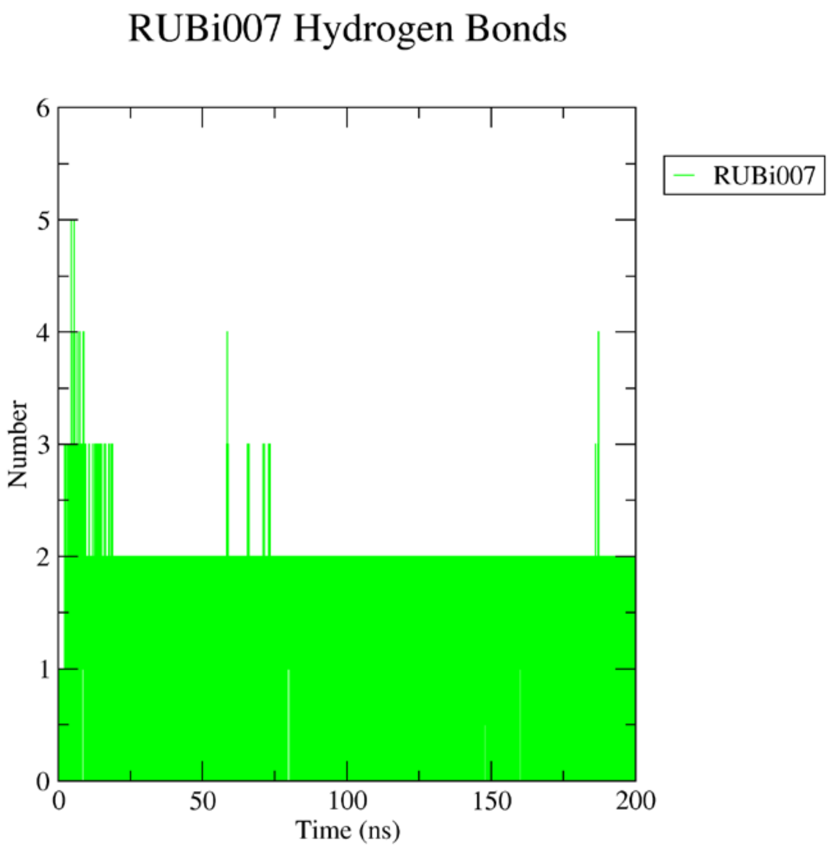

Figure 9G: The time evolution of the number of intermolecular hydrogen bonds formed between the RUBi007 and *Tb*PTR1 protein in the *Tb*PTR1-RUBi007 complex

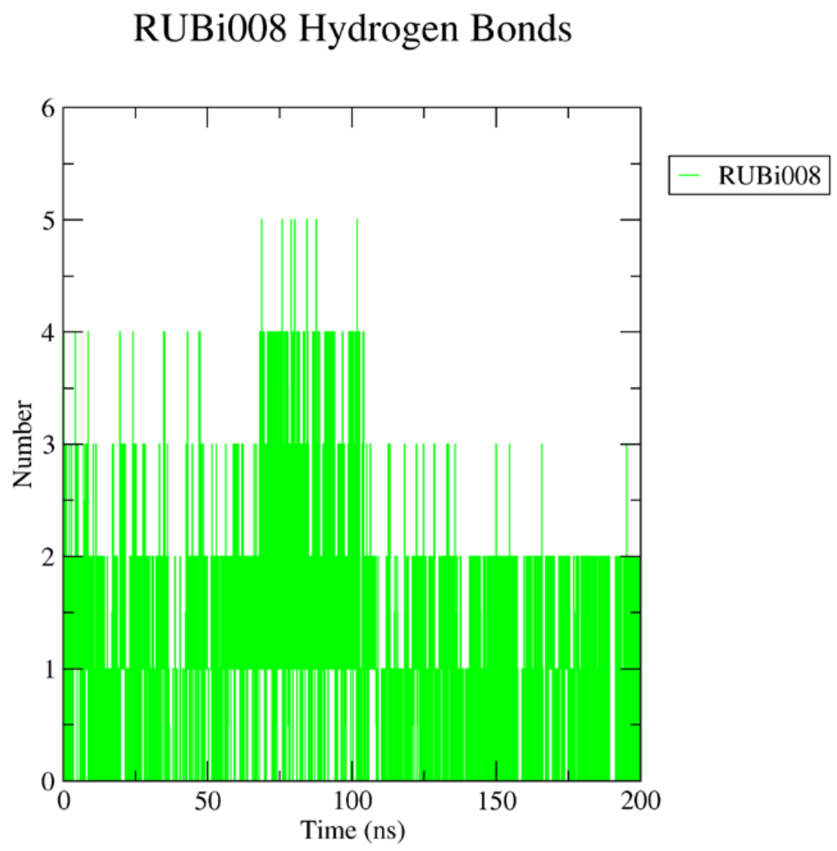

Figure 9H: The time evolution of the number of intermolecular hydrogen bonds formed between the RUBi008 and *Tb*PTR1 protein in the *Tb*PTR1-RUBi008 complex

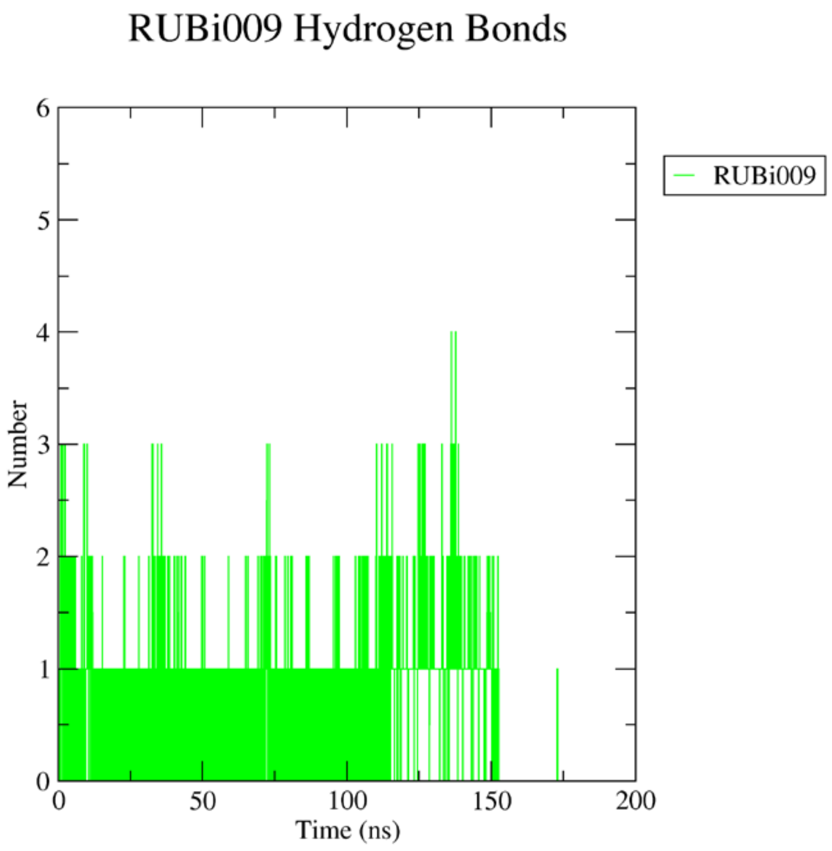

Figure 9I: The time evolution of the number of intermolecular hydrogen bonds formed between the RUBi009 and *Tb*PTR1 protein in the *Tb*PTR1-RUBi009 complex

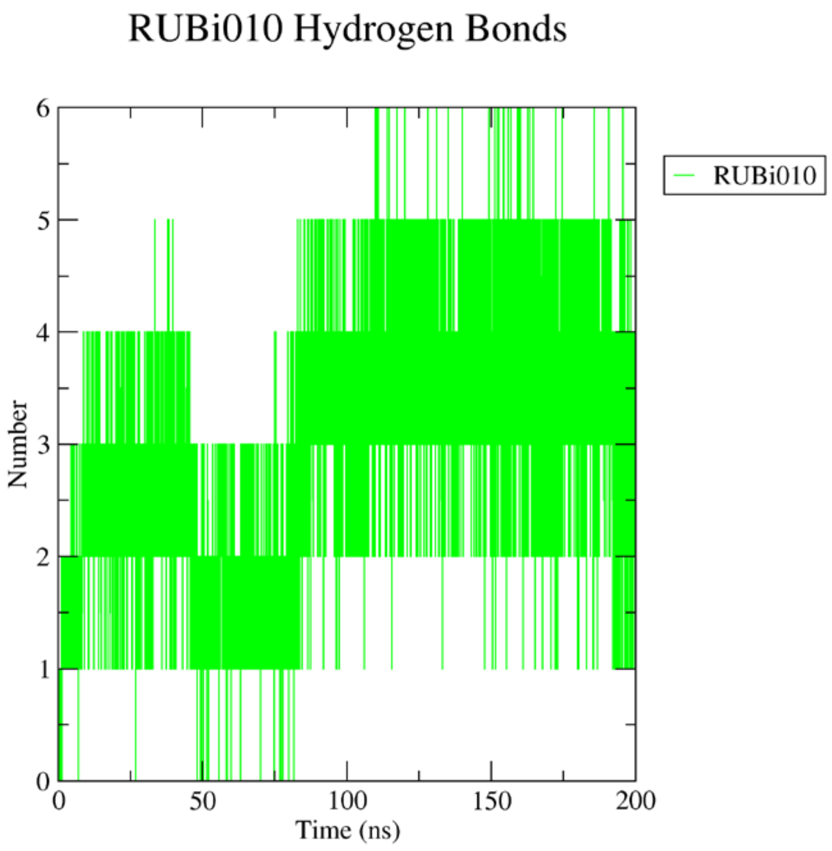

Figure 9J: The time evolution of the number of intermolecular hydrogen bonds formed between the RUBi010 and *Tb*PTR1 protein in the *Tb*PTR1-RUBi010 complex

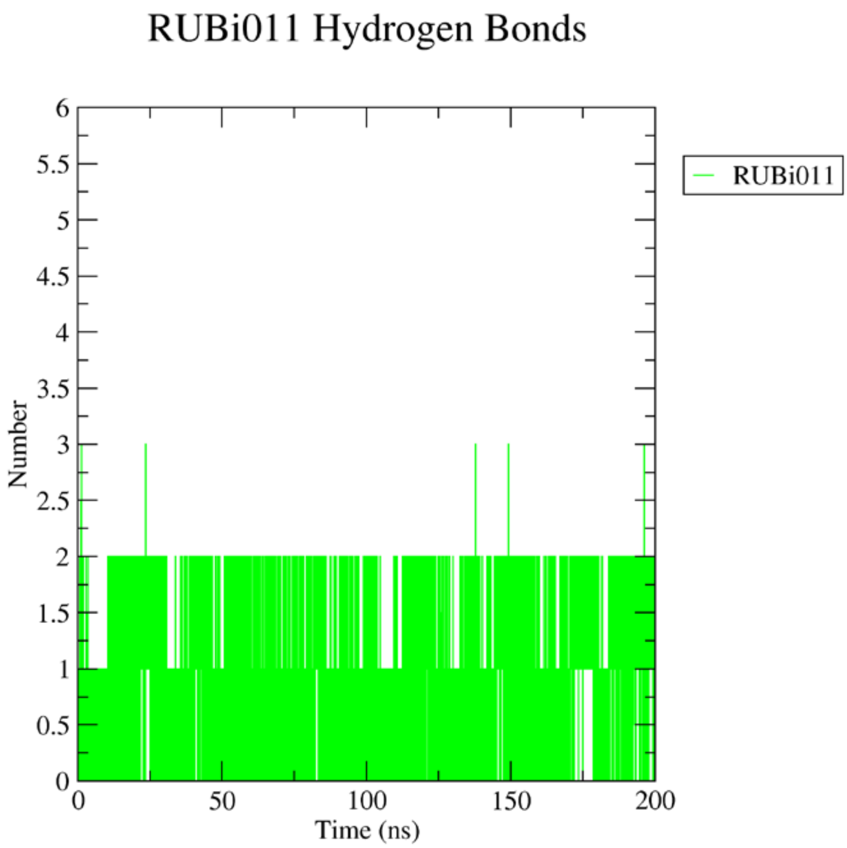

Figure 9K: The time evolution of the number of intermolecular hydrogen bonds formed between the RUBi011 and *Tb*PTR1 protein in the *Tb*PTR1-RUBi011 complex

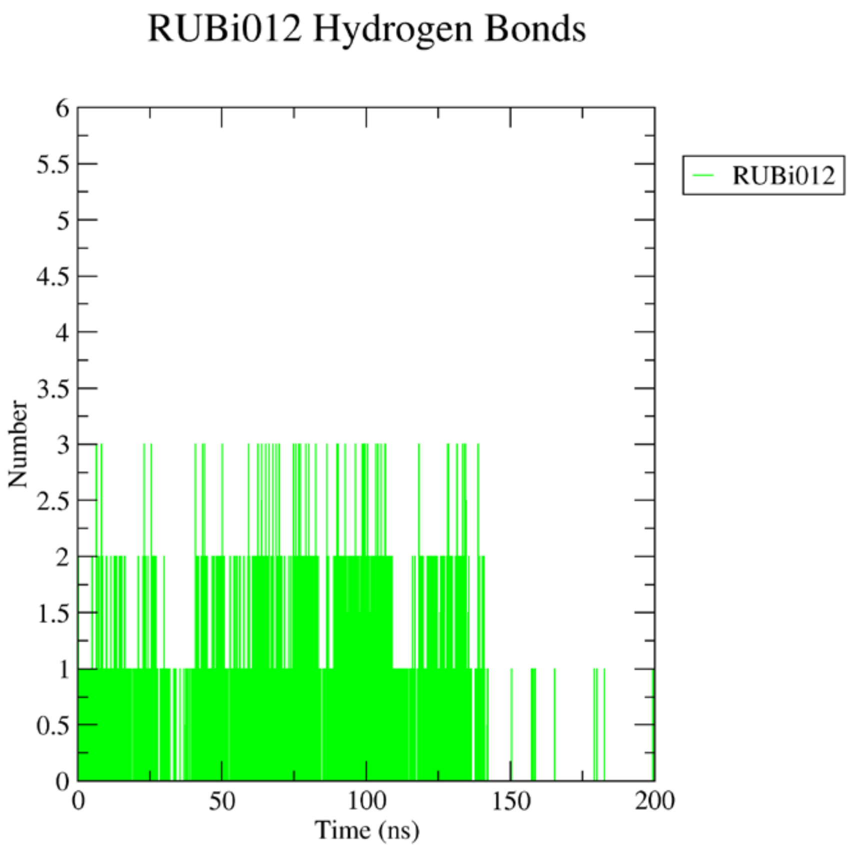

Figure 9L: The time evolution of the number of intermolecular hydrogen bonds formed between the RUBi012 and *Tb*PTR1 protein in the *Tb*PTR1-RUBi012 complex

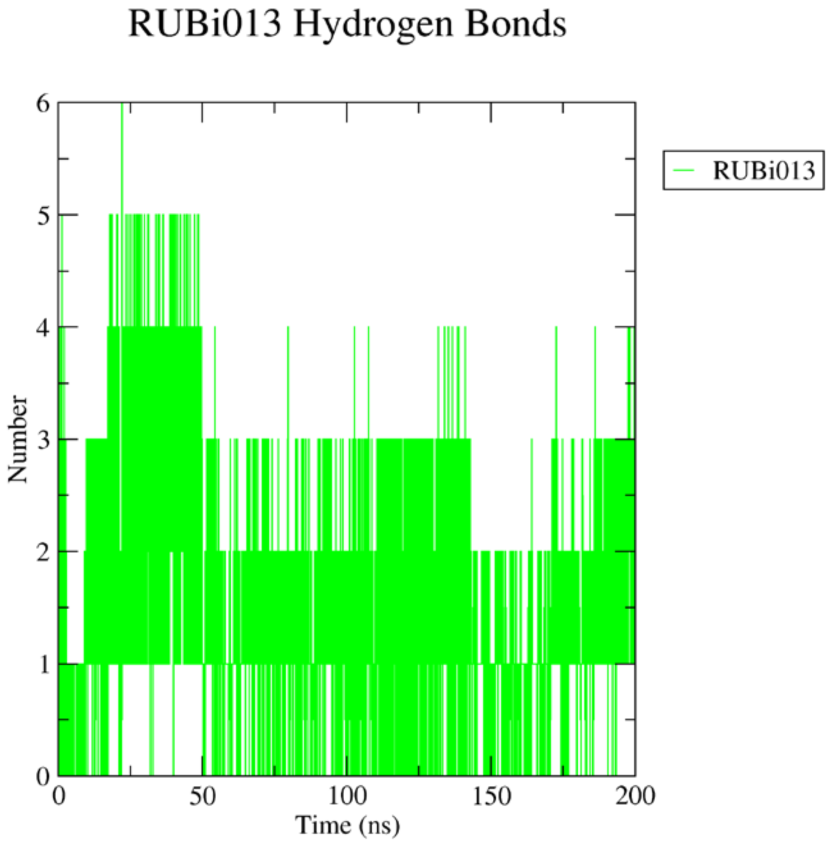

Figure 9M: The time evolution of the number of intermolecular hydrogen bonds formed between the RUBi013 and *Tb*PTR1 protein in the *Tb*PTR1-RUBi013 complex

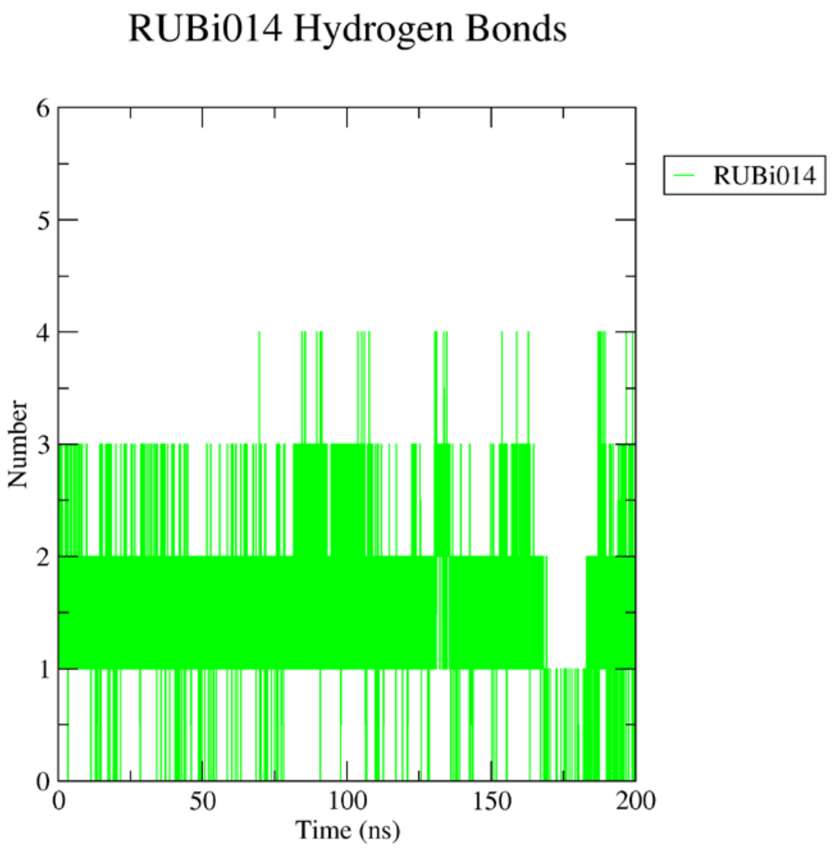

Figure 9N: The time evolution of the number of intermolecular hydrogen bonds formed between the RUBi014 and *Tb*PTR1 protein in the *Tb*PTR1-RUBi014 complex

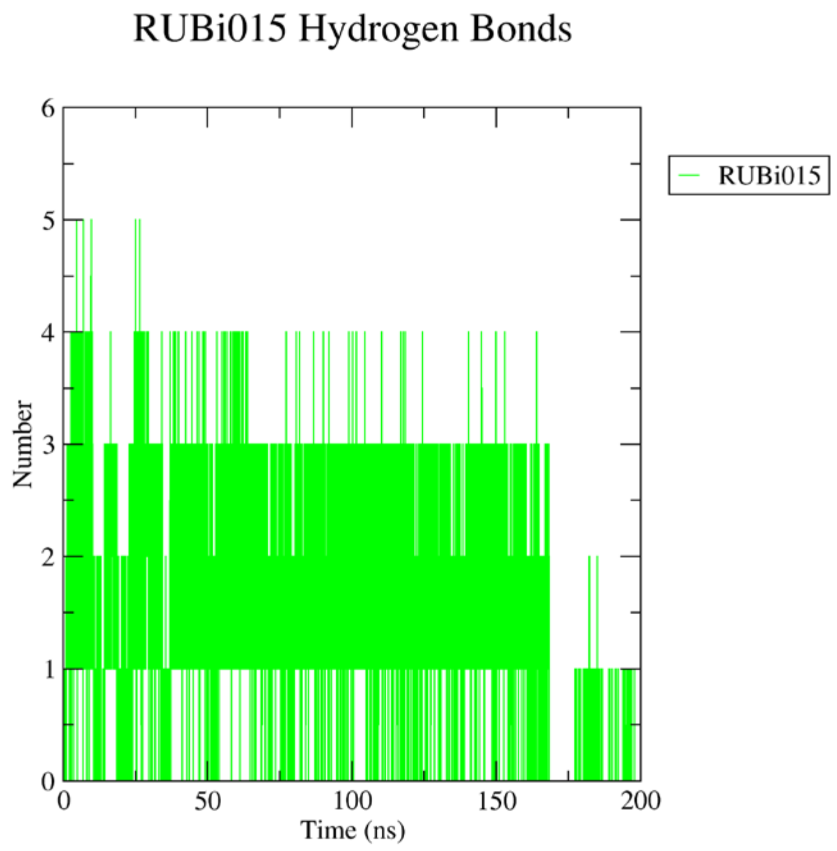

Figure 9O: The time evolution of the number of intermolecular hydrogen bonds formed between the RUBi015 and *Tb*PTR1 protein in the *Tb*PTR1-RUBi015 complex

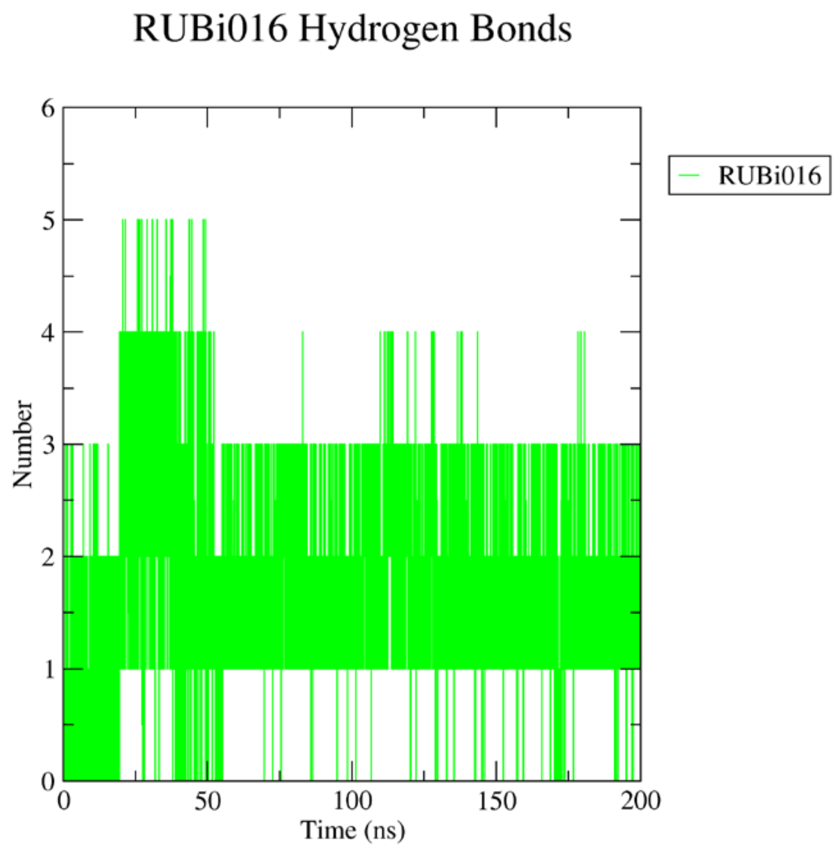

Figure 9P: The time evolution of the number of intermolecular hydrogen bonds formed between the RUBi016 and *Tb*PTR1 protein in the *Tb*PTR1-RUBi016 complex

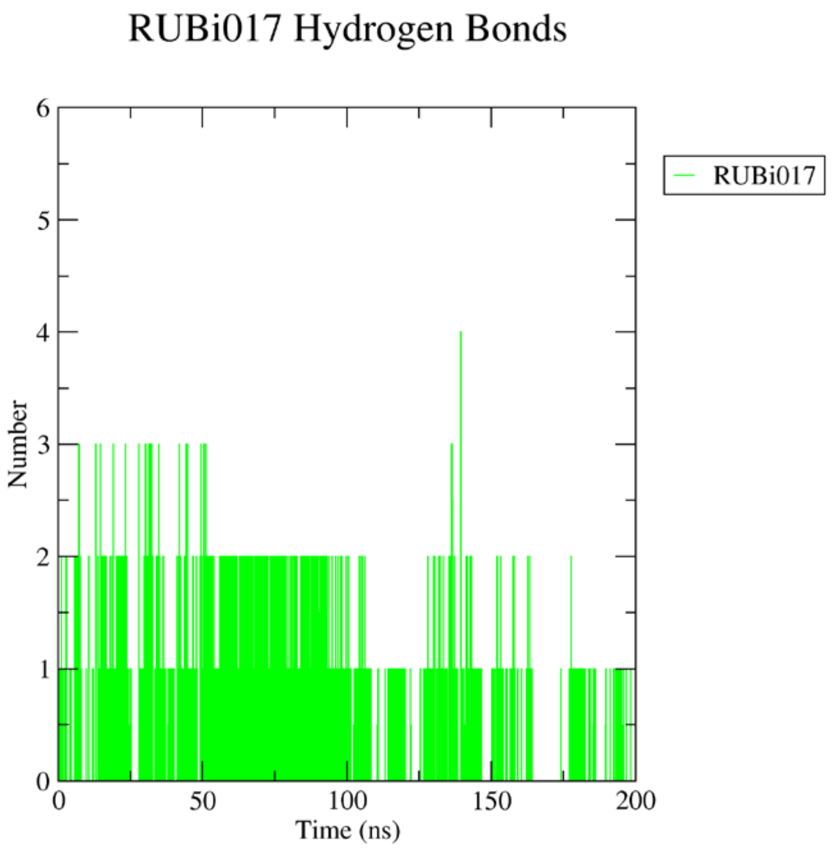

Figure 9R: The time evolution of the number of intermolecular hydrogen bonds formed between the RUBi017 and *Tb*PTR1 protein in the *Tb*PTR1-RUBi017 complex

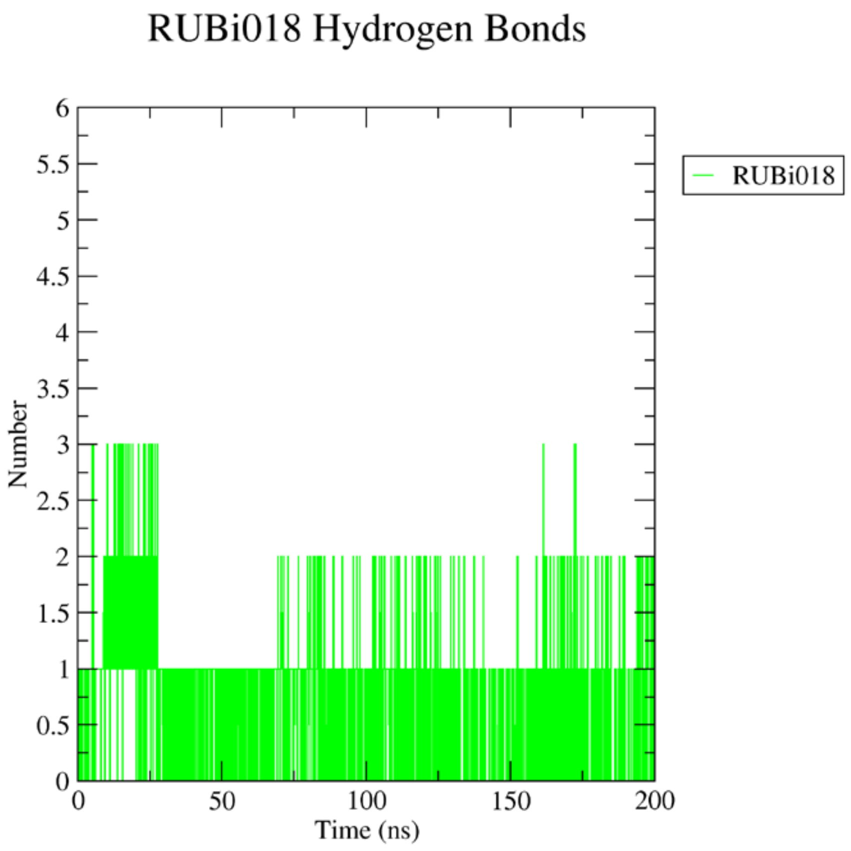

Figure 9S: The time evolution of the number of intermolecular hydrogen bonds formed between the RUBi018 and *Tb*PTR1 protein in the *Tb*PTR1-RUBi018 complex

Supplementary Figure S10: The Root Mean Square Fluctuation (RMSF) of all the protein in the protein-ligand complexes.

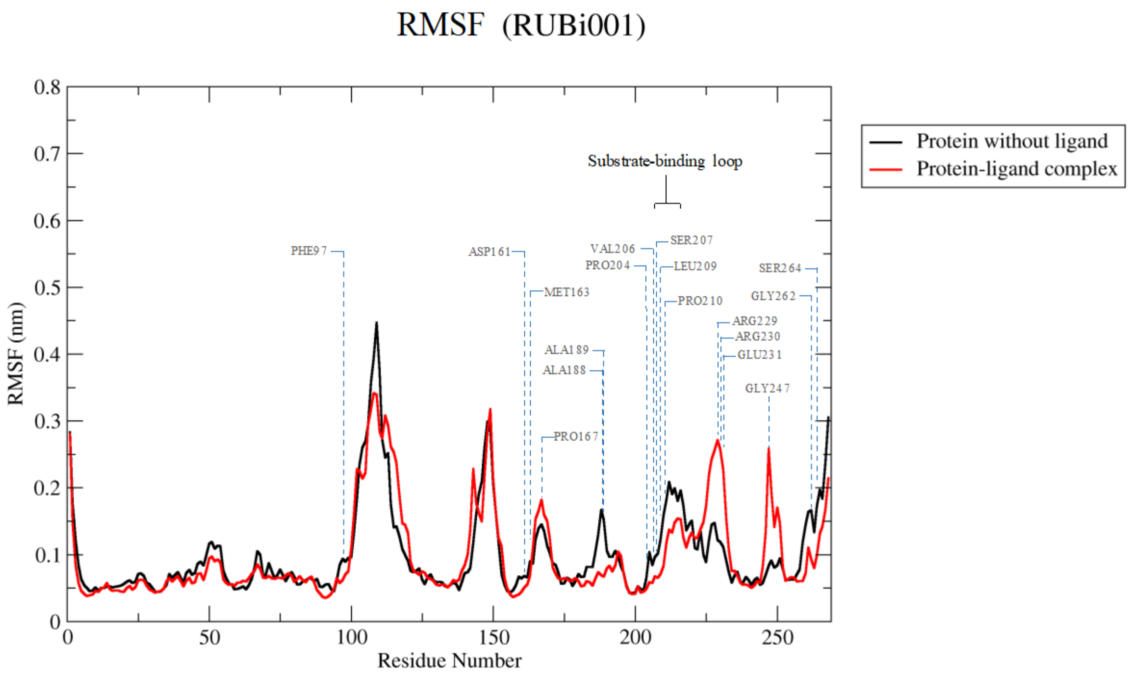

Figure 10A: The RMSF of backbone protein in the protein-ligand complex shown as a function of time. The apo protein was colored black and the *Tb*PTR1-RUBi001 complex red.

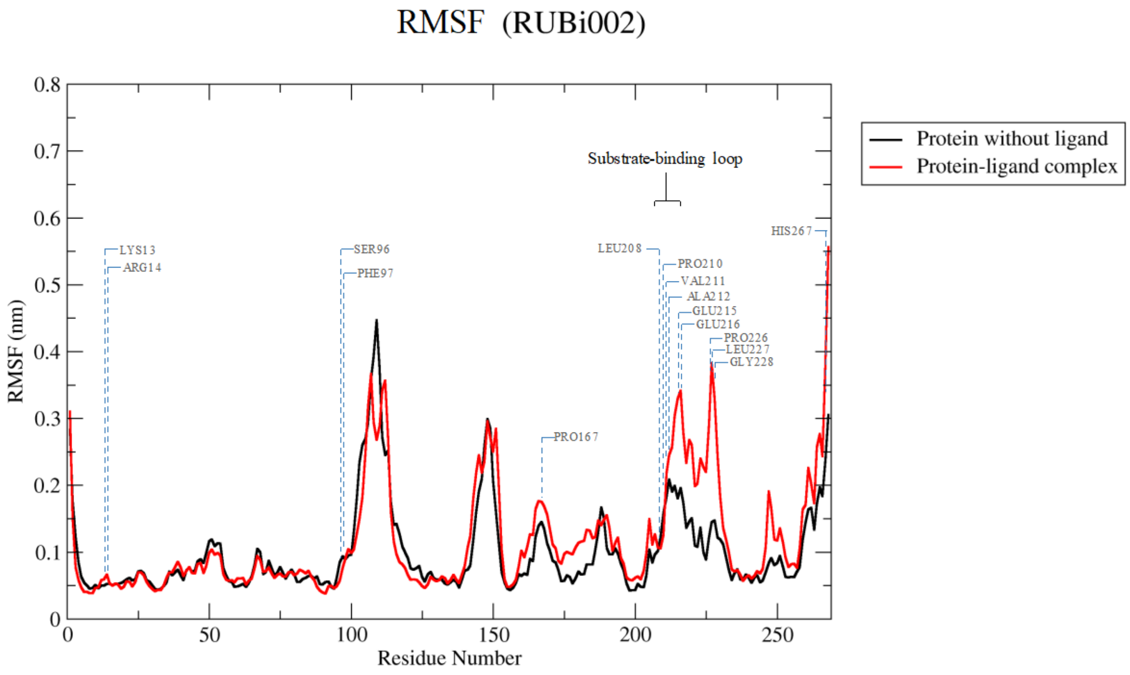

Figure 10B: The RMSF of backbone protein in the protein-ligand complex shown as a function of time. The apo protein was colored black and the *Tb*PTR1-RUBi002 complex red.

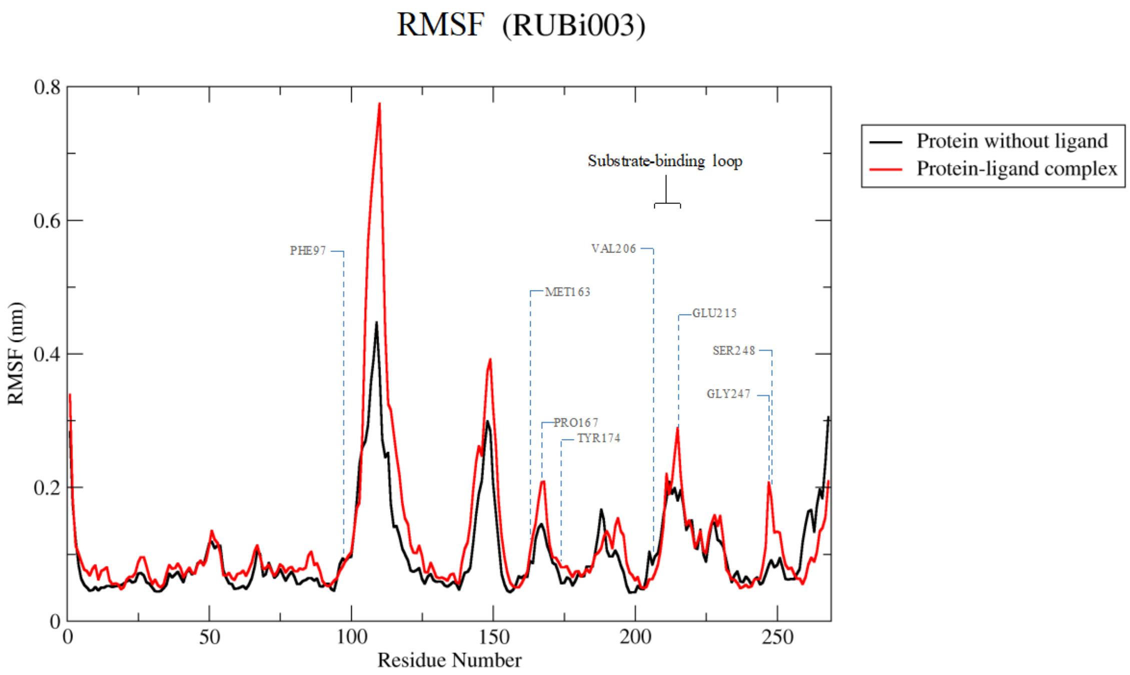

Figure 10C: The RMSF of backbone protein in the protein-ligand complex shown as a function of time. The apo protein was colored black and the *Tb*PTR1-RUBi003 complex red.

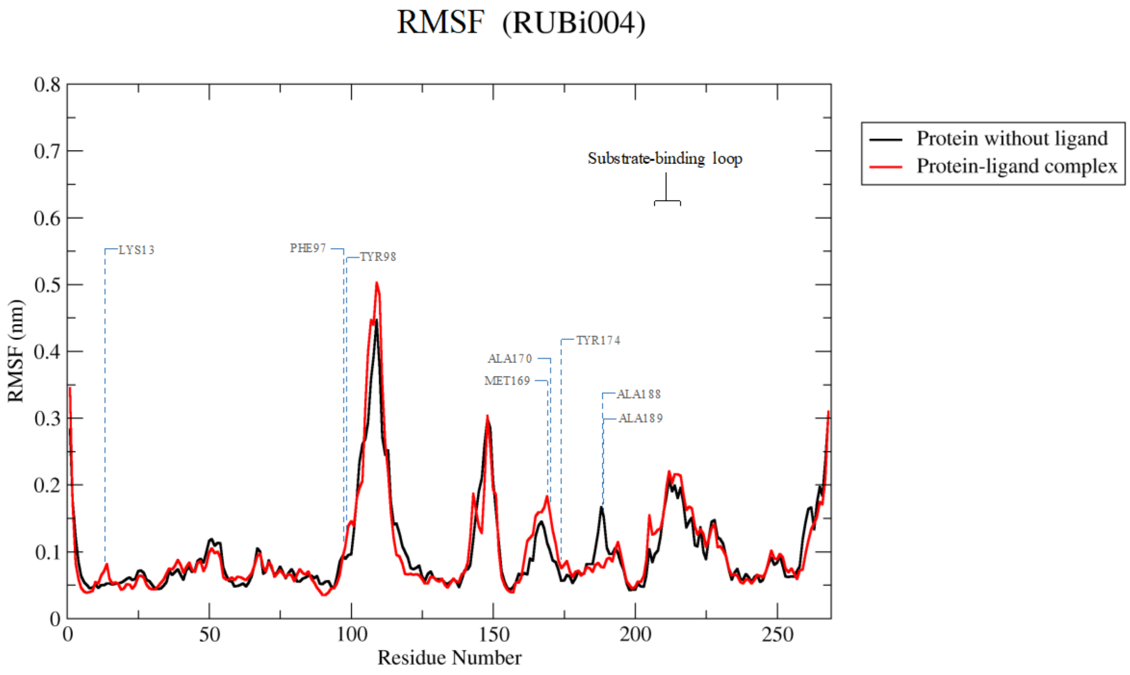

Figure 10D: The RMSF of backbone protein in the protein-ligand complex shown as a function of time. The apo protein was colored black and the *Tb*PTR1-RUBi004 complex red.

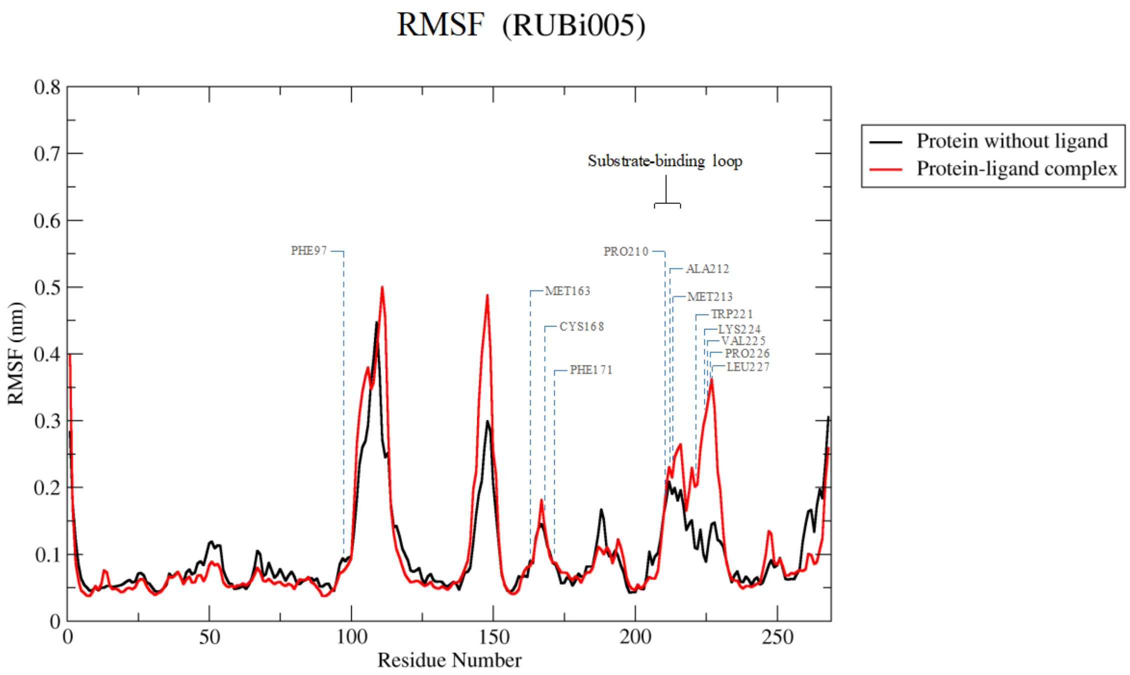

Figure 10E: The RMSF of backbone protein in the protein-ligand complex shown as a function of time. The apo protein was colored black and the *Tb*PTR1-RUBi005 complex red.

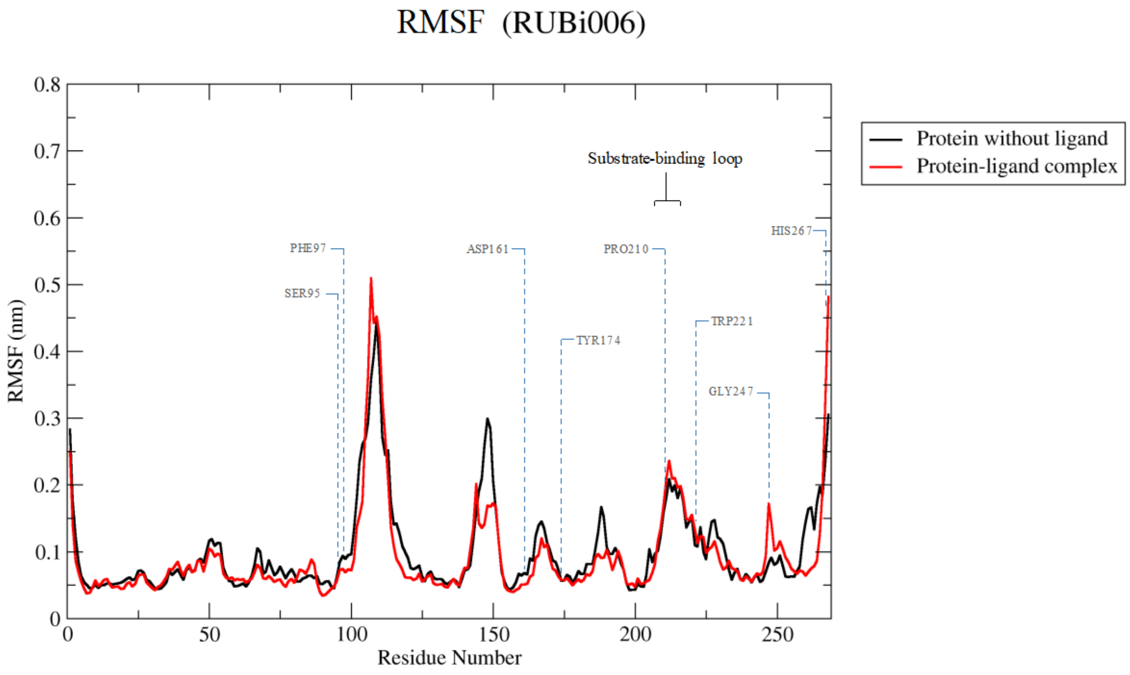

Figure 10F: The RMSF of backbone protein in the protein-ligand complex shown as a function of time. The apo protein was colored black and the *Tb*PTR1-RUBi006 complex red.

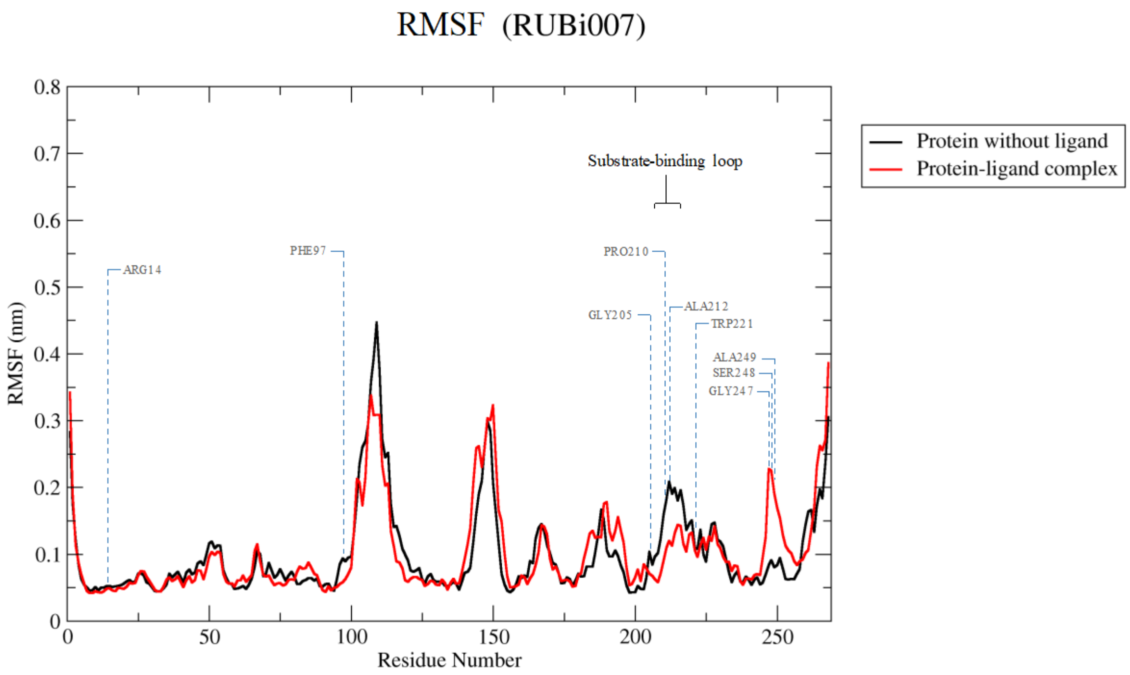

Figure 10G: The RMSF of backbone protein in the protein-ligand complex shown as a function of time. The apo protein was colored black and the *Tb*PTR1-RUBi007 complex red.

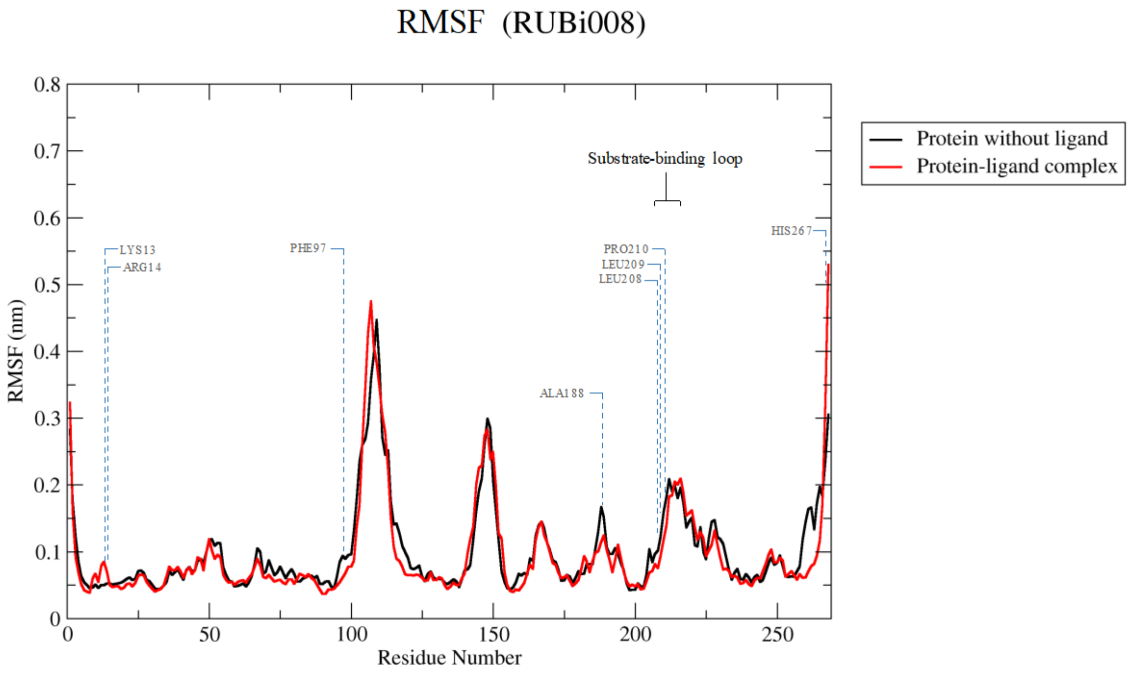

Figure 10H: The RMSF of backbone protein in the protein-ligand complex shown as a function of time. The apo protein was colored black and the *Tb*PTR1-RUBi008 complex red.

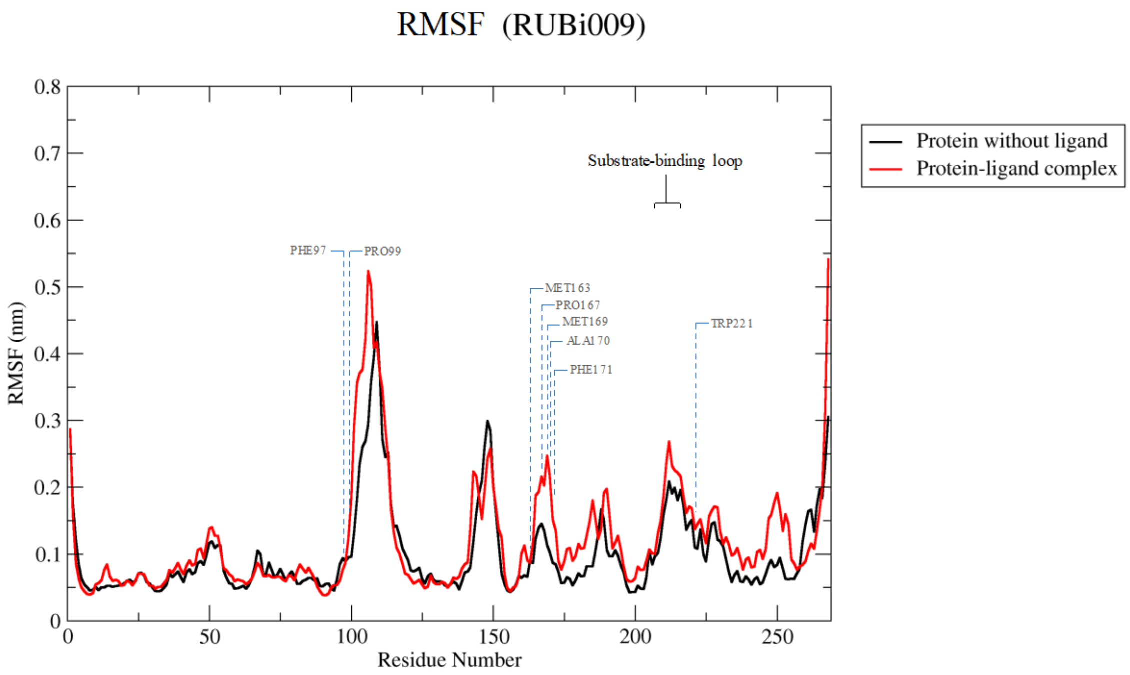

Figure 10I: The RMSF of backbone protein in the protein-ligand complex shown as a function of time. The apo protein was colored black and the *Tb*PTR1-RUBi009 complex red.

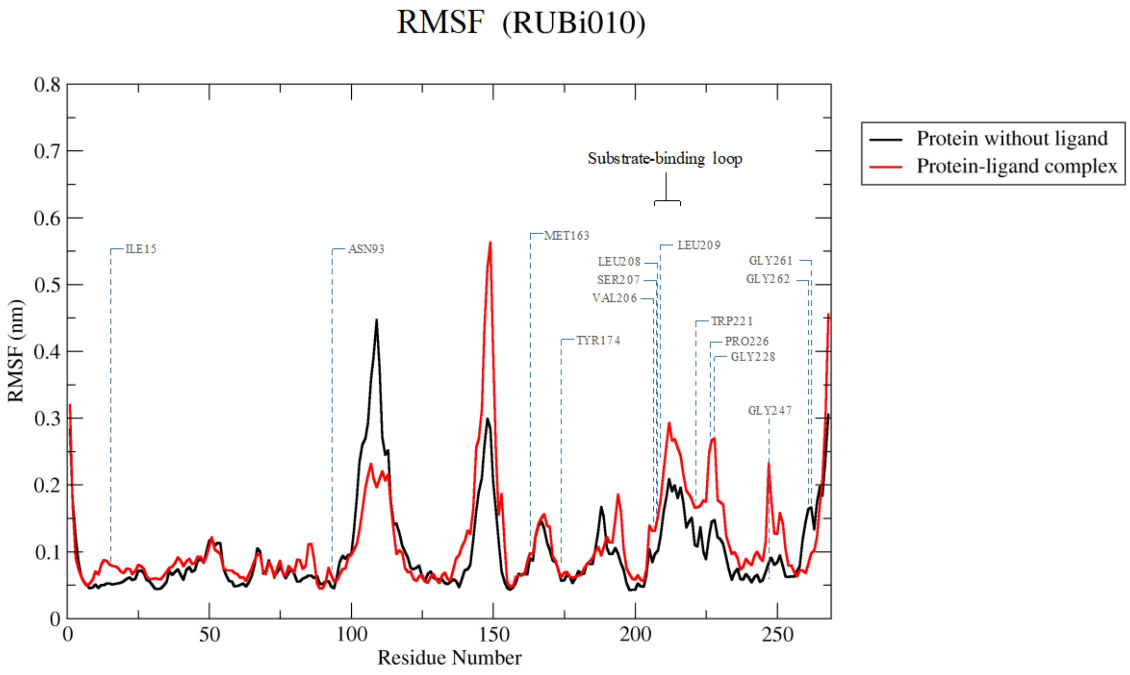

Figure 10J: The RMSF of backbone protein in the protein-ligand complex shown as a function of time. The apo protein was colored black and the *Tb*PTR1-RUBi010 complex red.

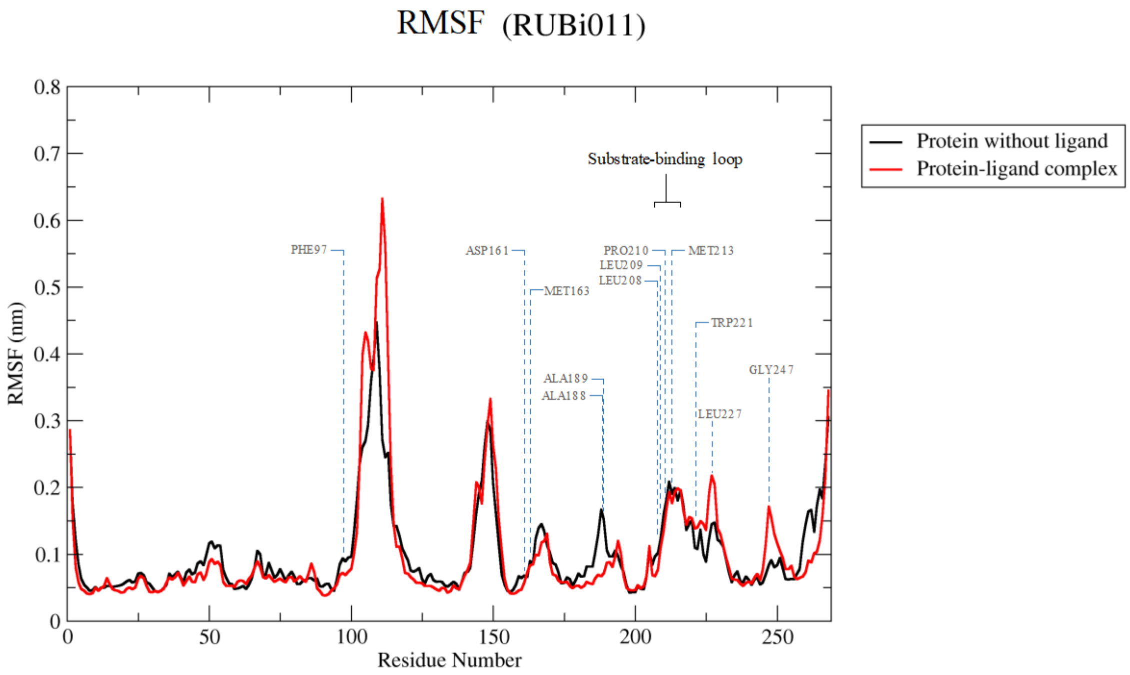

Figure 10K: The RMSF of backbone protein in the protein-ligand complex shown as a function of time. The apo protein was colored black and the *Tb*PTR1-RUBi011 complex red.

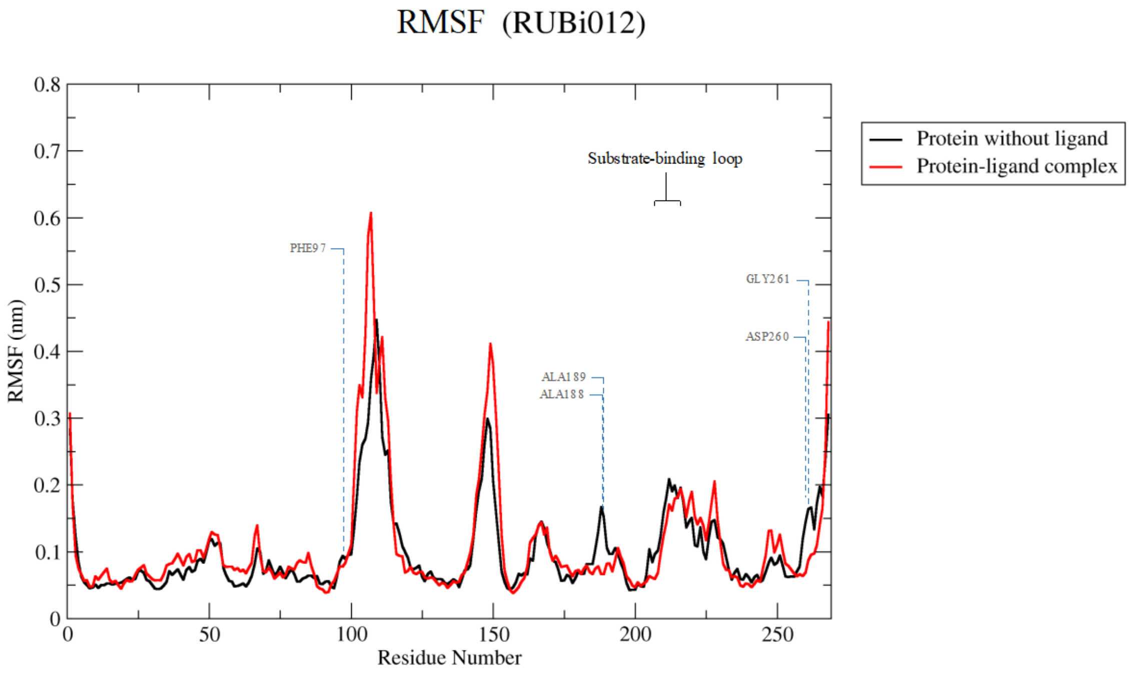

Figure 10L: The RMSF of backbone protein in the protein-ligand complex shown as a function of time. The apo protein was colored black and the *Tb*PTR1-RUBi012 complex red.

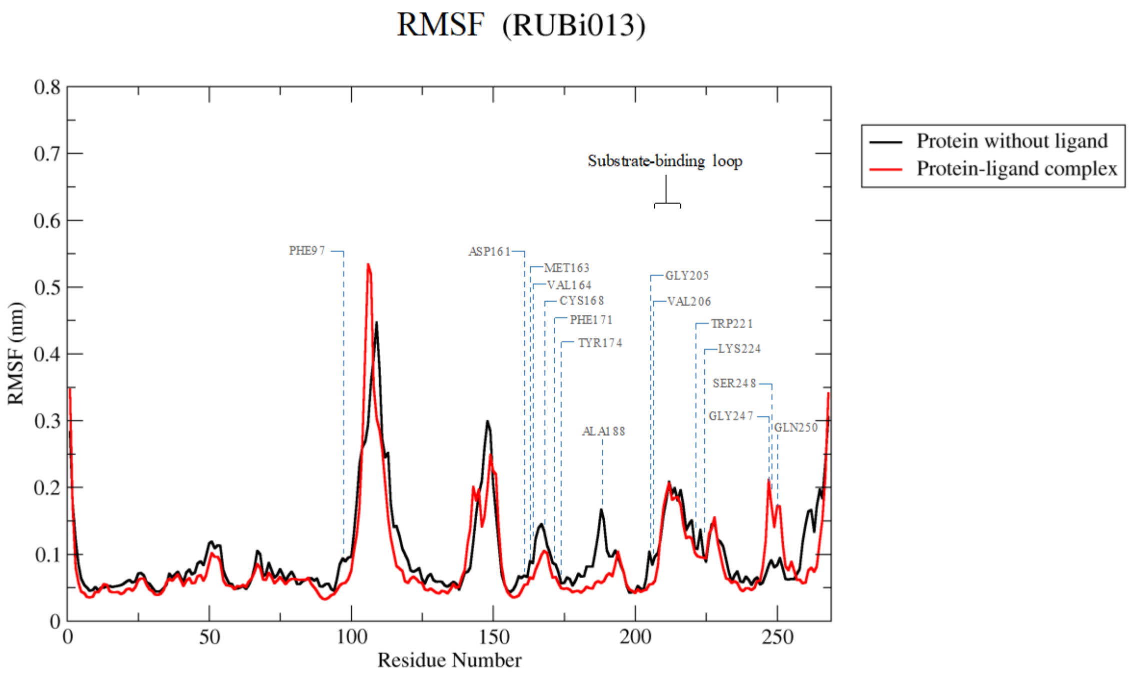

Figure 10M: The RMSF of backbone protein in the protein-ligand complex shown as a function of time. The apo protein was colored black and the *Tb*PTR1-RUBi013 complex red.

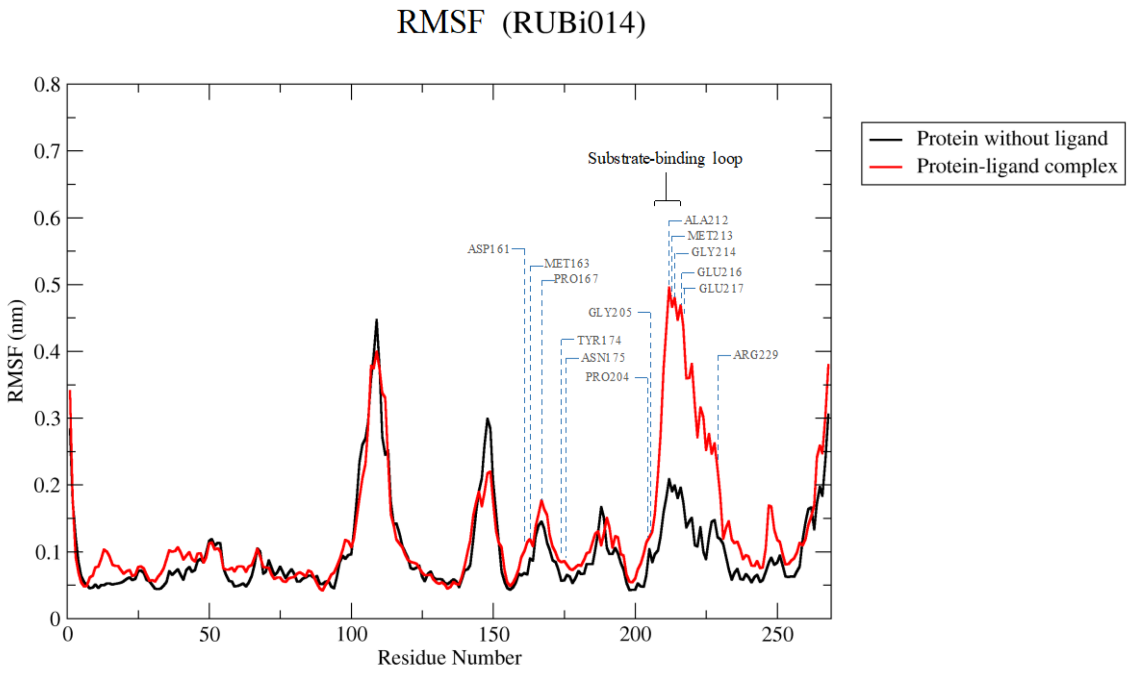

Figure 10N: The RMSF of backbone protein in the protein-ligand complex shown as a function of time. The apo protein was colored black and the *Tb*PTR1-RUBi014 complex red.

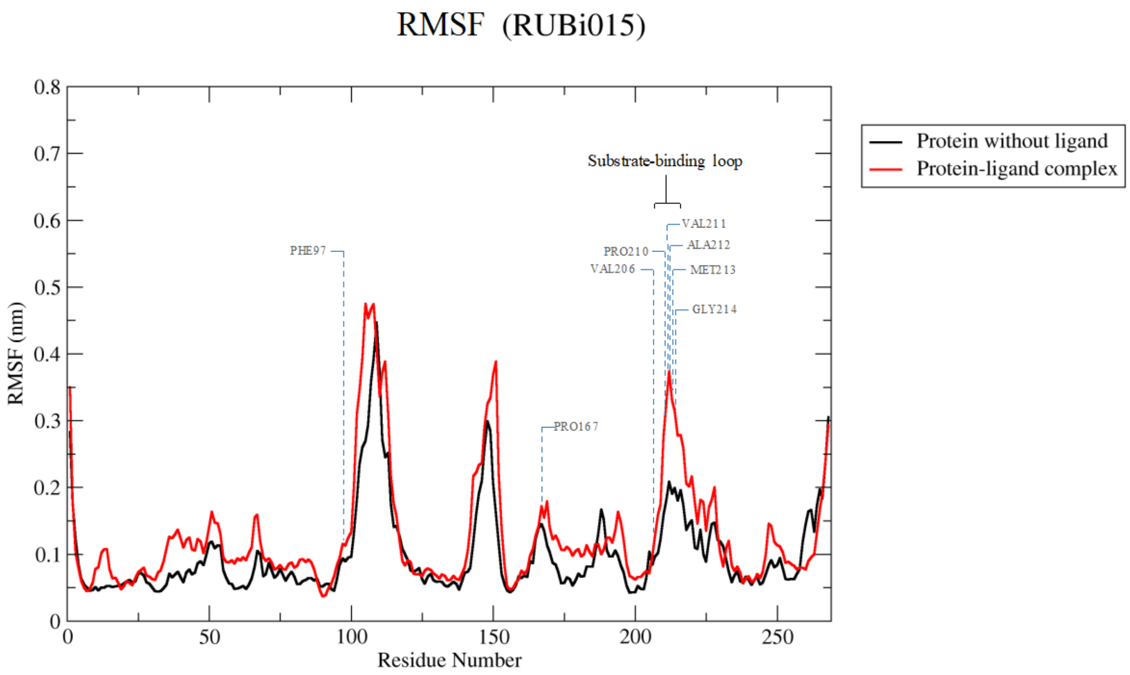

Figure 10O: The RMSF of backbone protein in the protein-ligand complex shown as a function of time. The apo protein was colored black and the *Tb*PTR1-RUBi015 complex red.

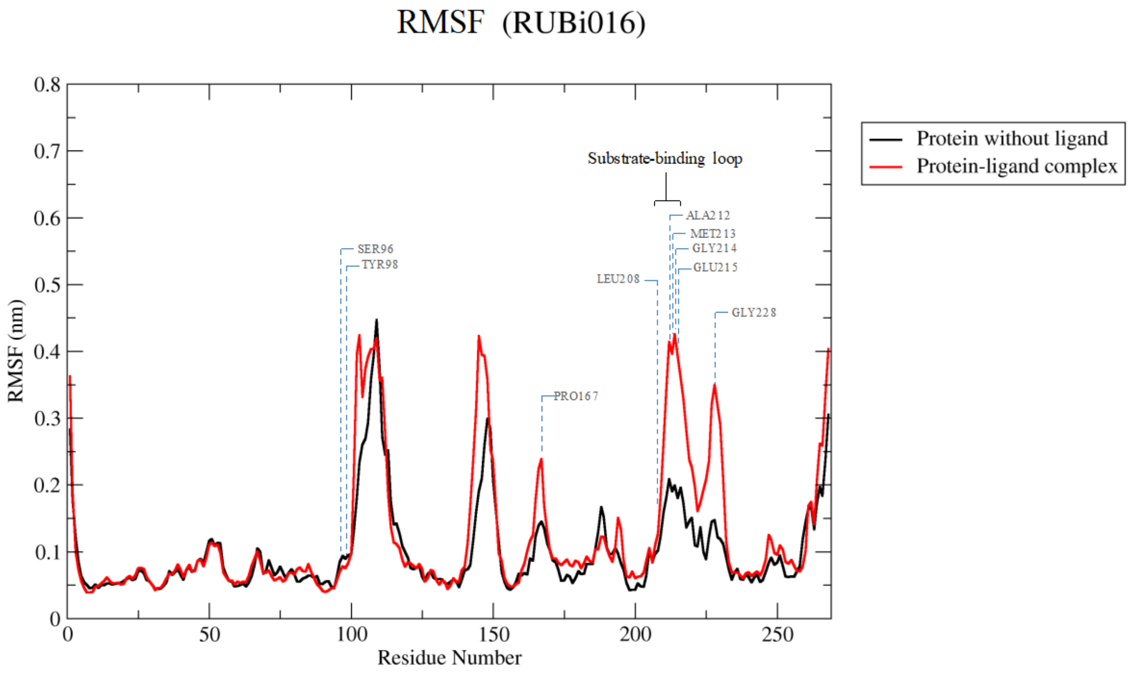

Figure 10P: The RMSF of backbone protein in the protein-ligand complex shown as a function of time. The apo protein was colored black and the *Tb*PTR1-RUBi016 complex red.

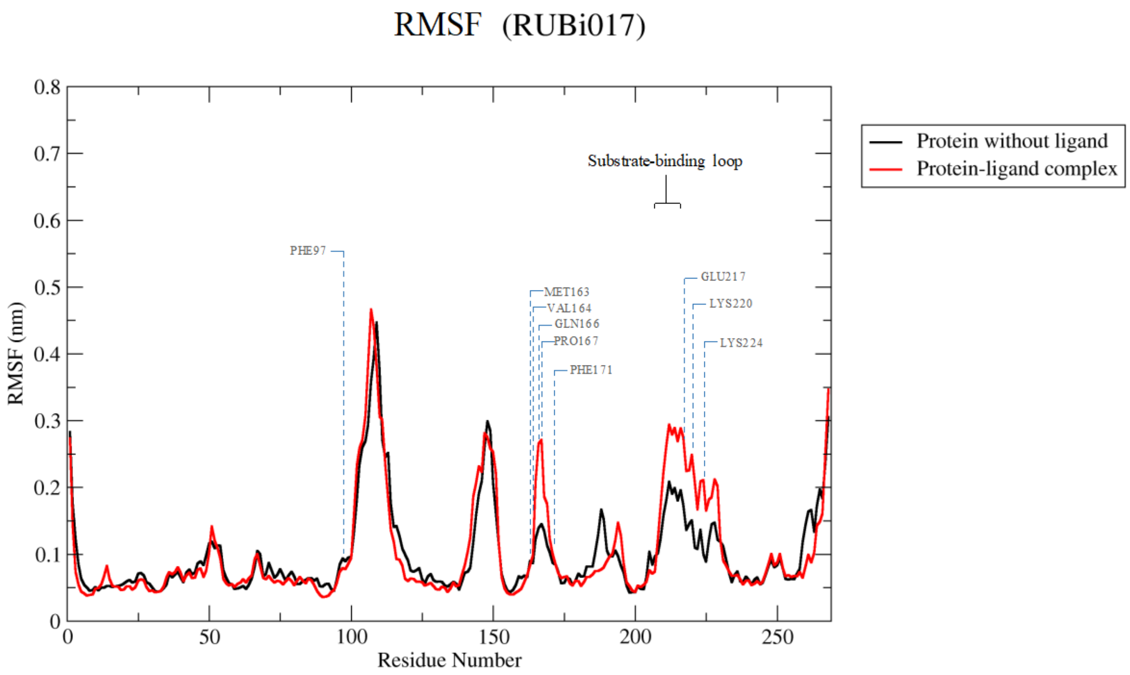

Figure 10R: The RMSF of backbone protein in the protein-ligand complex shown as a function of time. The apo protein was colored black and the *Tb*PTR1-RUBi017 complex red.

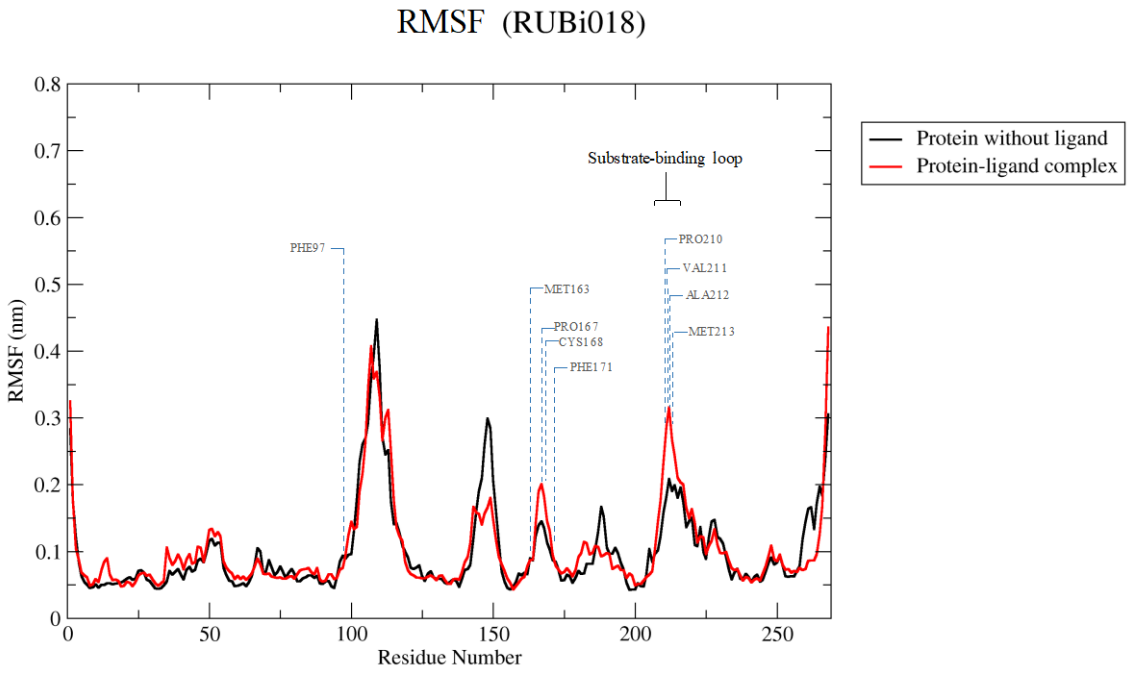

Figure 10S: The RMSF of backbone protein in the protein-ligand complex shown as a function of time. The apo protein was colored black and the *Tb*PTR1-RUBi018 complex red.

Supplementary Figure S11: Energetic contribution of the of *Tb*PTR1 residues in binding of the protein-ligand complexes.

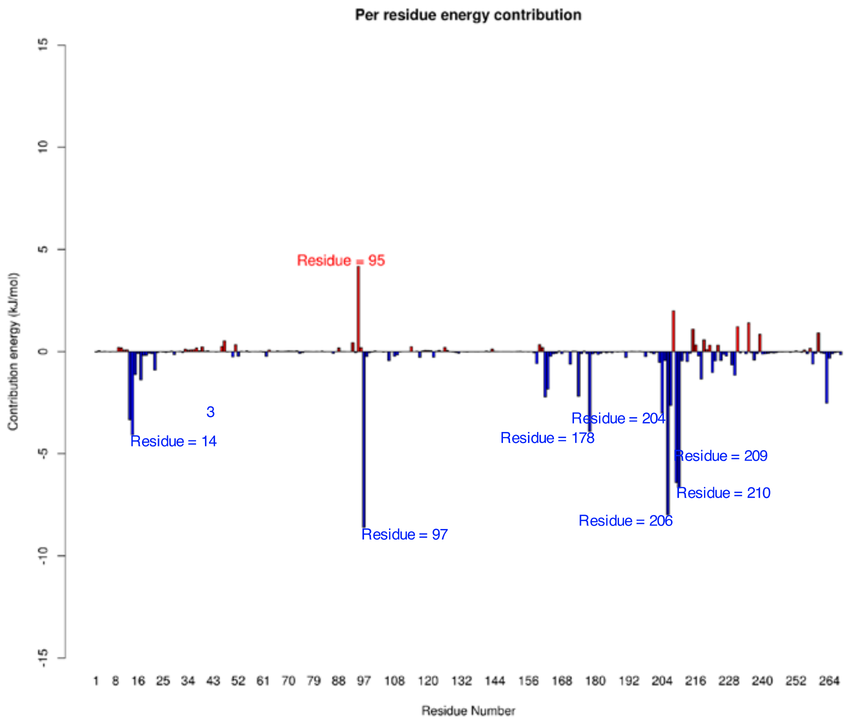

Figure 11A: Energetic contribution of the of *Tb*PTR1 residues in binding. The energies of the *Tb*PTR1-RUBi001 complex are given as kilojoules per mole. A) Δ_E_MM, Δ_G_polar, Δ_G_nonpolar and Δ_G_binding as a function of time B) Binding energy contribution energy for each residue.

A)

B)

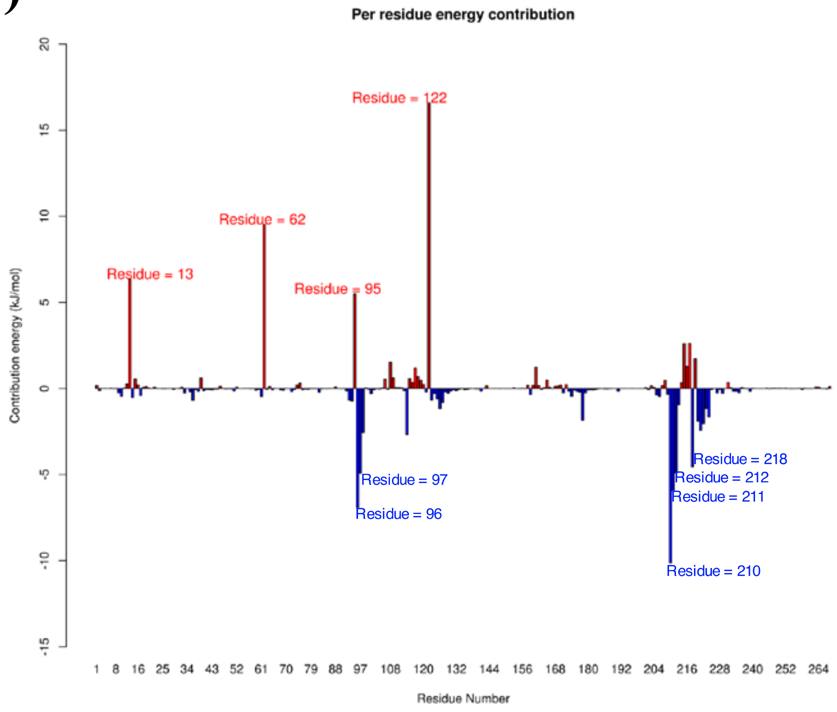

Figure 11B: Energetic contribution of the of *Tb*PTR1 residues in binding. The energies of the *Tb*PTR1-RUBi002 complex are given as kilojoules per mole. A) Δ_E_MM, Δ_G_polar, Δ_G_nonpolar and Δ_G_binding as a function of time B) Binding energy contribution energy for each residue.

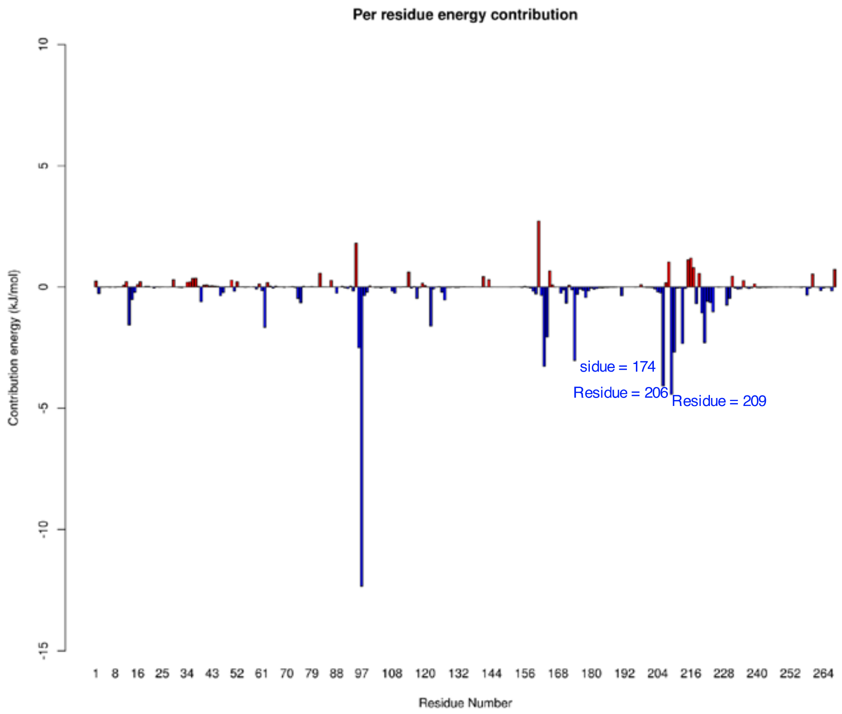

Figure 11C: Energetic contribution of the of *Tb*PTR1 residues in binding. The energies of the *Tb*PTR1-RUBi003 complex are given as kilojoules per mole. A) Δ_E_MM, Δ_G_polar, Δ_G_nonpolar and Δ_G_binding as a function of time B) Binding energy contribution energy for each residue.

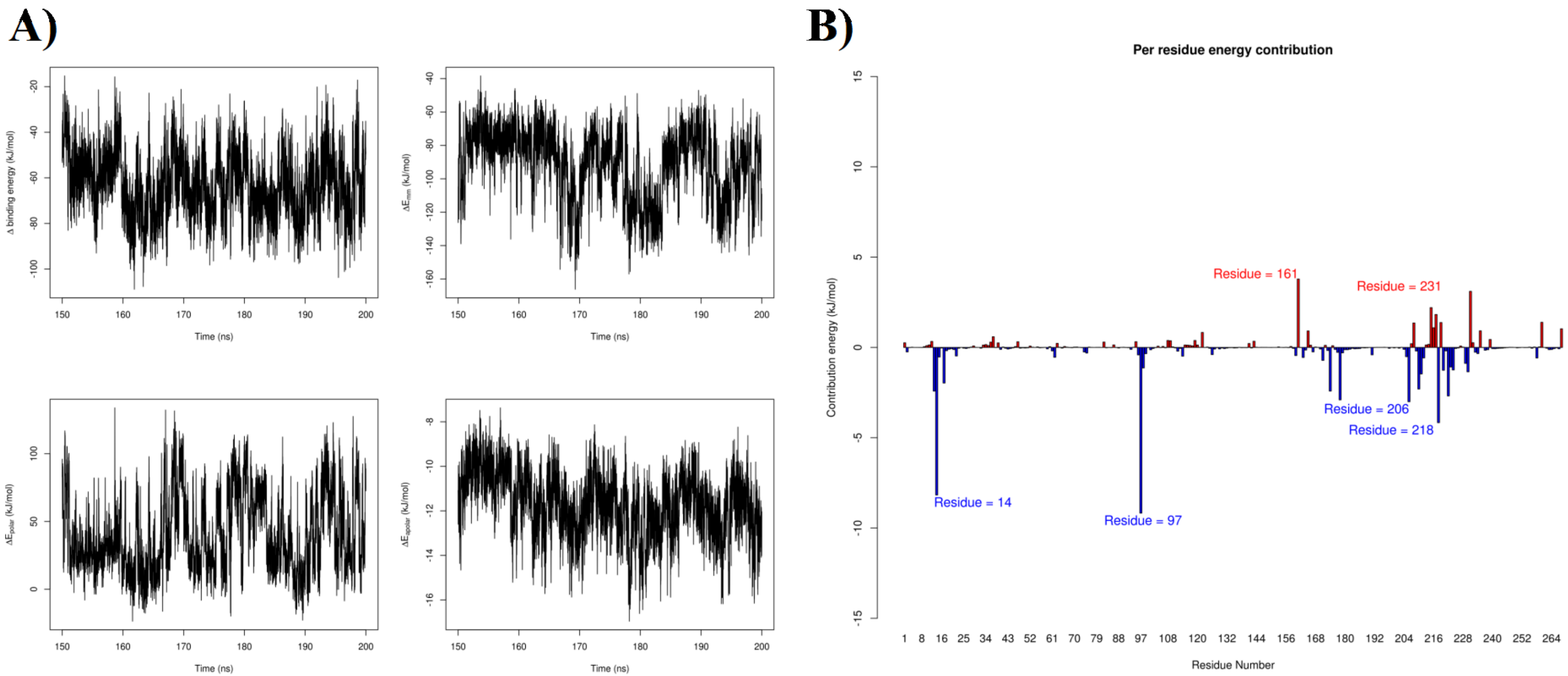

Figure 11D: Energetic contribution of the of *Tb*PTR1 residues in binding. The energies of the *Tb*PTR1-RUBi004 complex are given as kilojoules per mole. A) Δ_E_MM, Δ_G_polar, Δ_G_nonpolar and Δ_G_binding as a function of time B) Binding energy contribution energy for each residue.

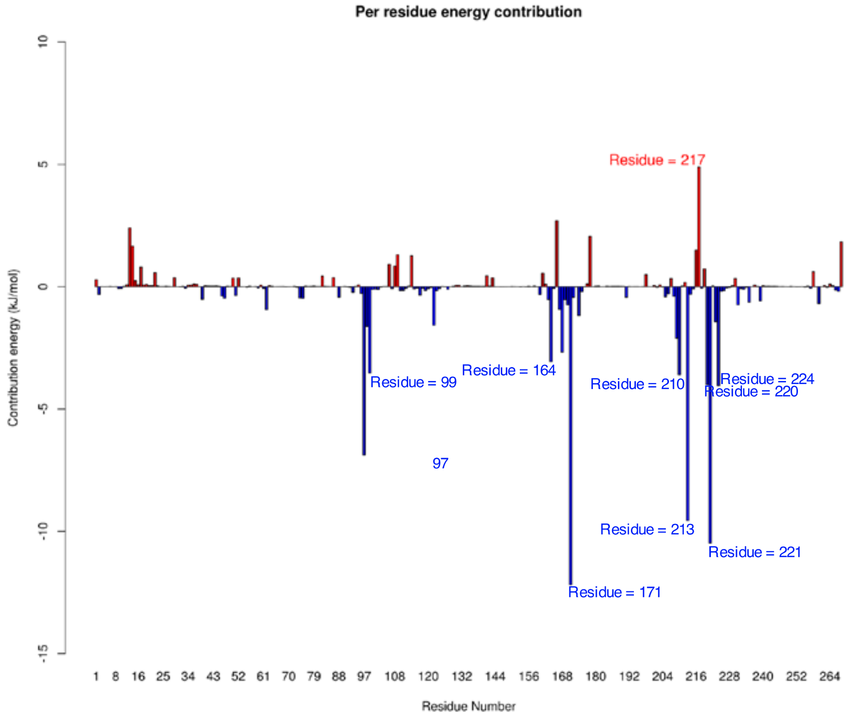

Figure 11E: Energetic contribution of the of *Tb*PTR1 residues in binding. The energies of the *Tb*PTR1-RUBi005 complex are given as kilojoules per mole. A) Δ_E_MM, Δ_G_polar, Δ_G_nonpolar and Δ_G_binding as a function of time B) Binding energy contribution energy for each residue.

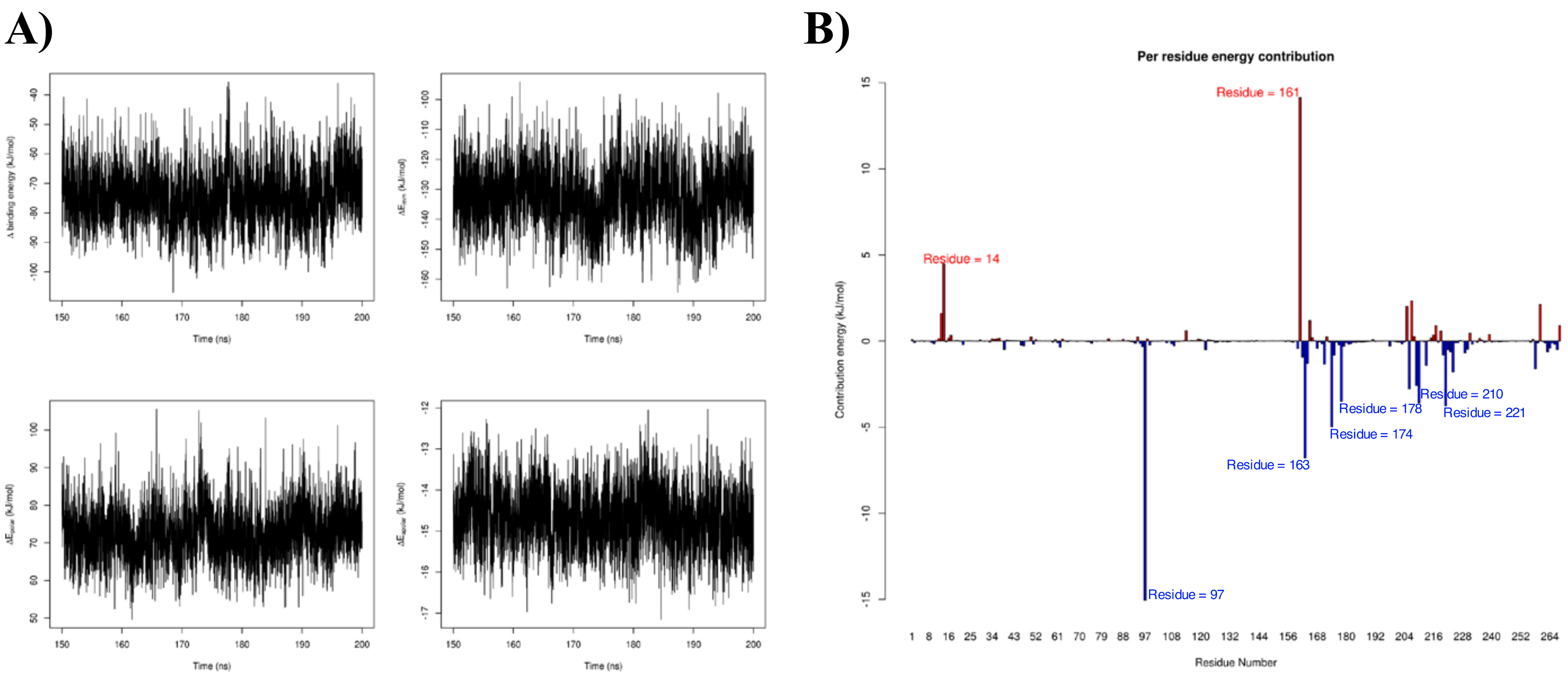

Figure 11F: Energetic contribution of the of *Tb*PTR1 residues in binding. The energies of the *Tb*PTR1-RUBi006 complex are given as kilojoules per mole. A) Δ_E_MM, Δ_G_polar, Δ_G_nonpolar and Δ_G_binding as a function of time B) Binding energy contribution energy for each residue.

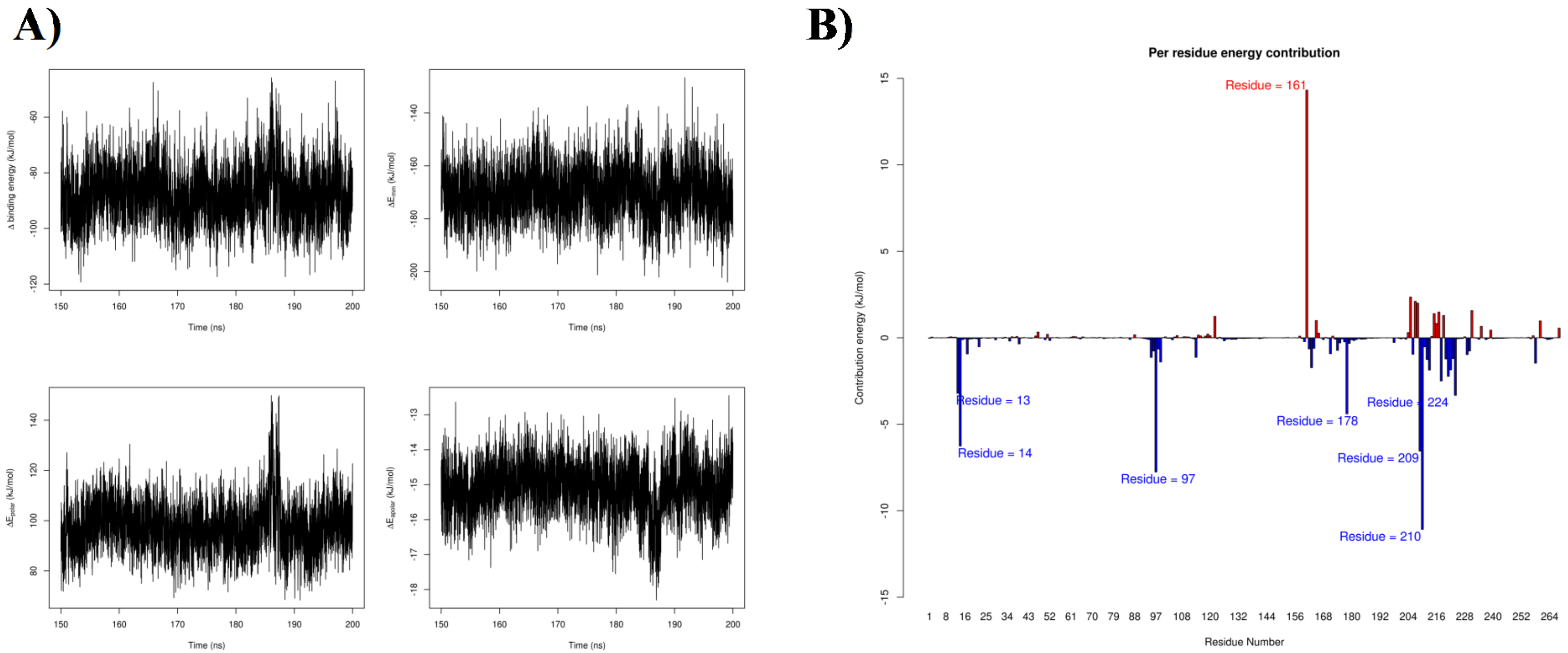

Figure 11G: Energetic contribution of the of *Tb*PTR1 residues in binding. The energies of the *Tb*PTR1-RUBi007 complex are given as kilojoules per mole. A) Δ_E_MM, Δ_G_polar, Δ_G_nonpolar and Δ_G_binding as a function of time B) Binding energy contribution energy for each residue.

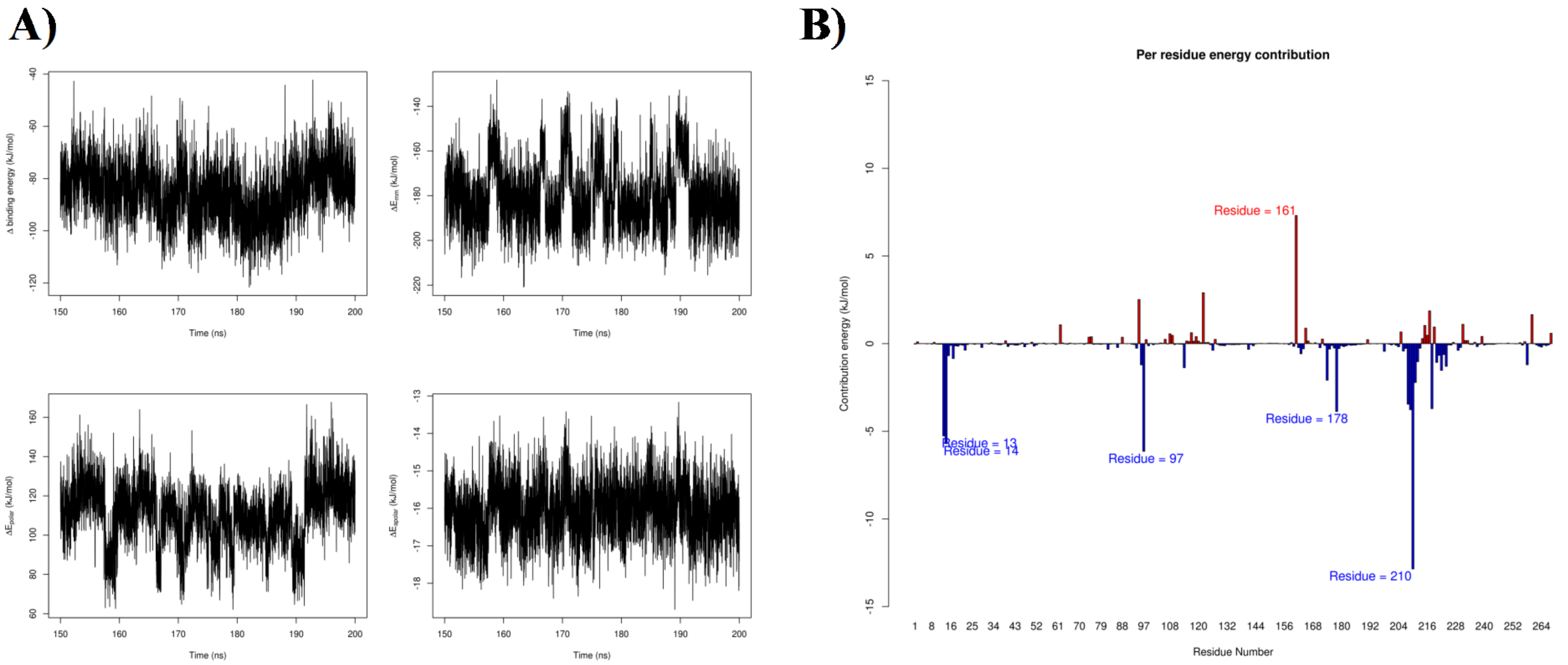

Figure 11H: Energetic contribution of the of *Tb*PTR1 residues in binding. The energies of the *Tb*PTR1-RUBi008 complex are given as kilojoules per mole. A) Δ_E_MM, Δ_G_polar, Δ_G_nonpolar and Δ_G_binding as a function of time B) Binding energy contribution energy for each residue.

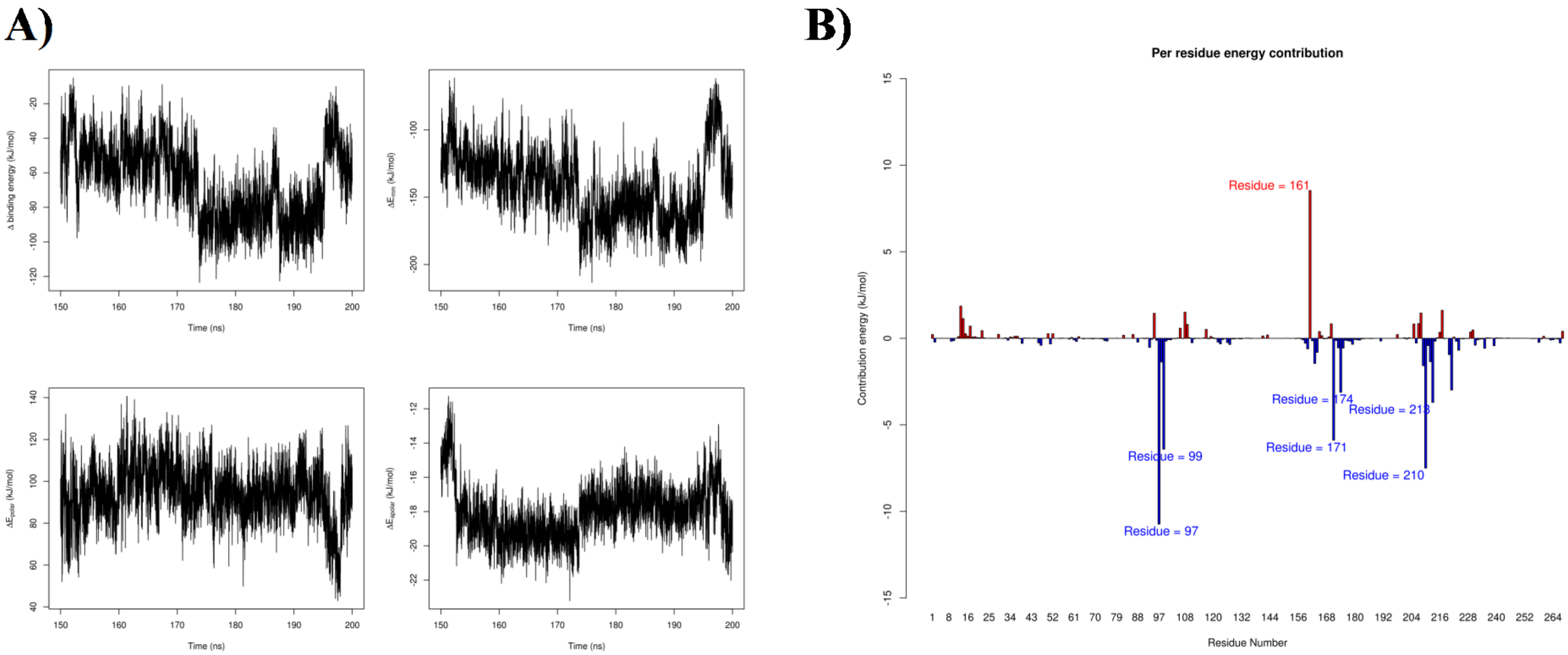

Figure 11I: Energetic contribution of the of *Tb*PTR1 residues in binding. The energies of the *Tb*PTR1-RUBi009 complex are given as kilojoules per mole. A) Δ_E_MM, Δ_G_polar, Δ_G_nonpolar and Δ_G_binding as a function of time B) Binding energy contribution energy for each residue.

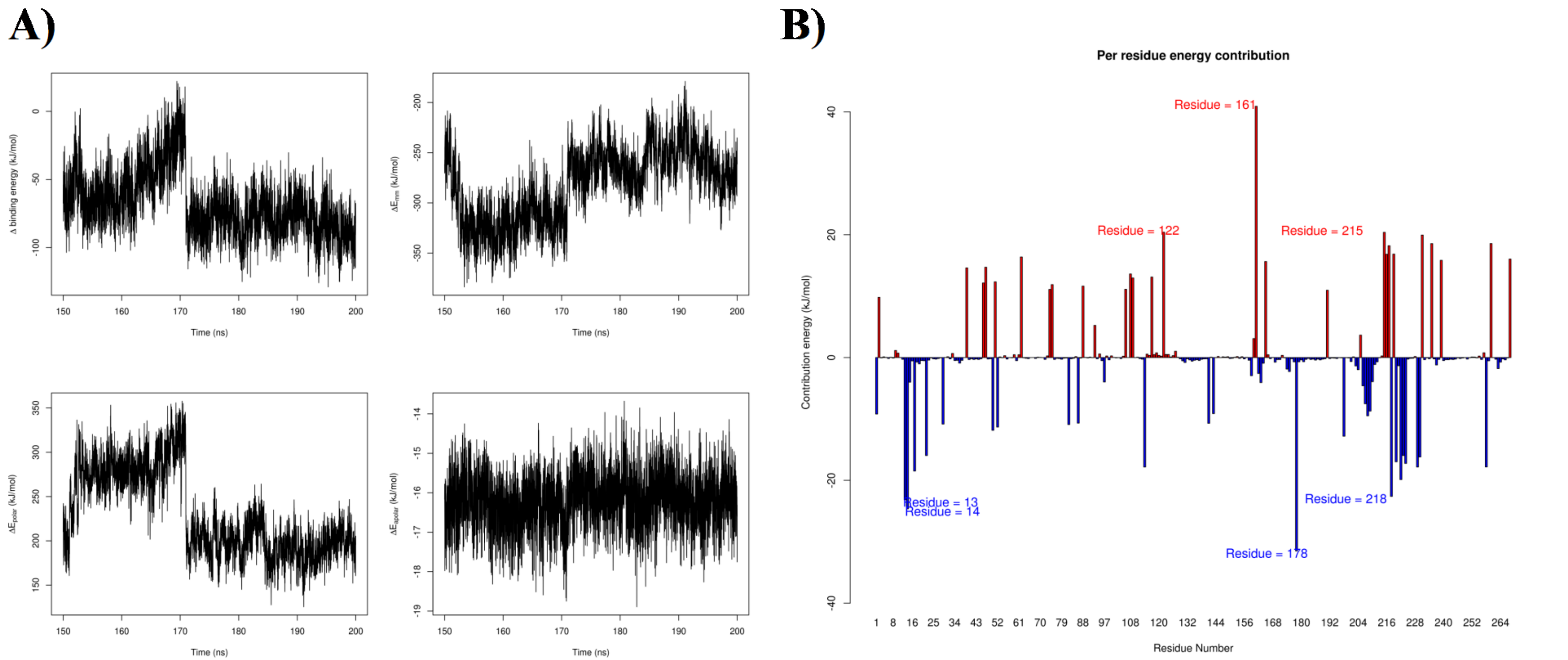

Figure 11J: Energetic contribution of the of *Tb*PTR1 residues in binding. The energies of the *Tb*PTR1-RUBi010 complex are given as kilojoules per mole. A) Δ_E_MM, Δ_G_polar, Δ_G_nonpolar and Δ_G_binding as a function of time B) Binding energy contribution energy for each residue.

A) B)

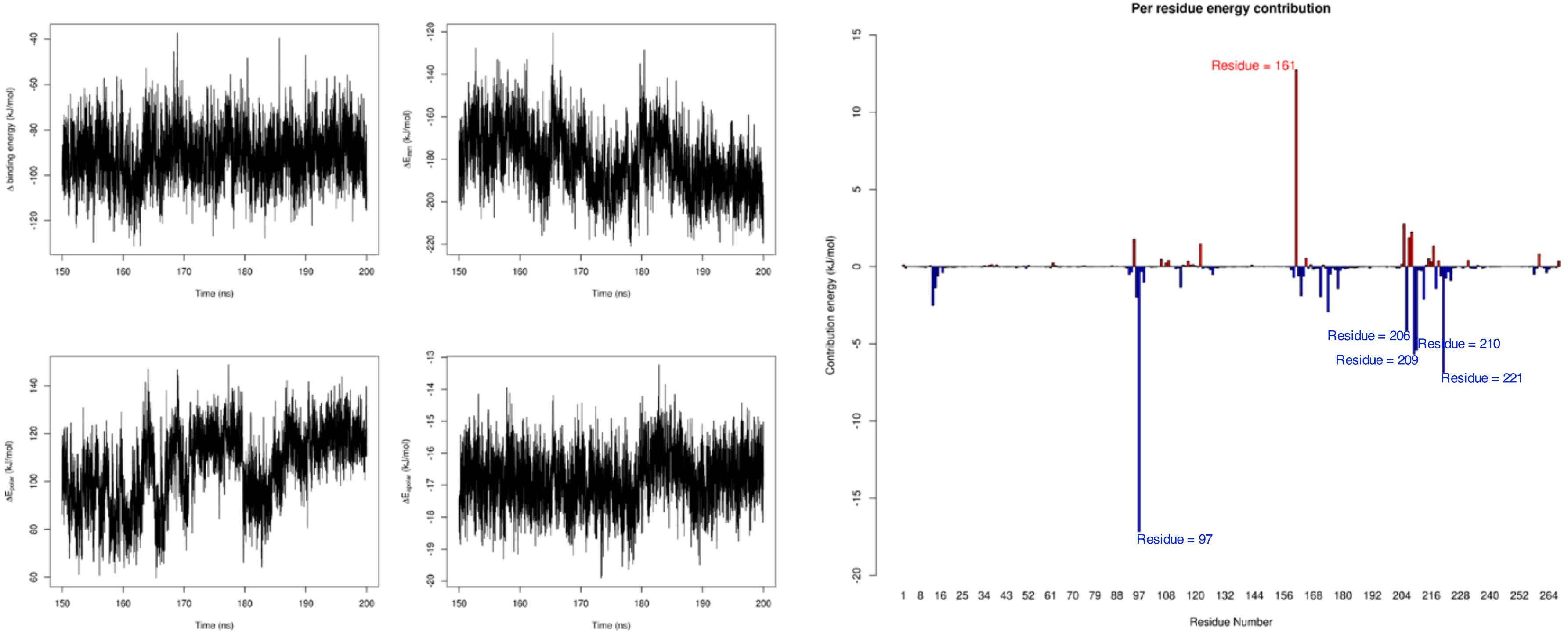

Figure 11K: Energetic contribution of the of *Tb*PTR1 residues in binding. The energies of the *Tb*PTR1-RUBi011 complex are given as kilojoules per mole. A) Δ_E_MM, Δ_G_polar, Δ_G_nonpolar and Δ_G_binding as a function of time B) Binding energy contribution energy for each residue.

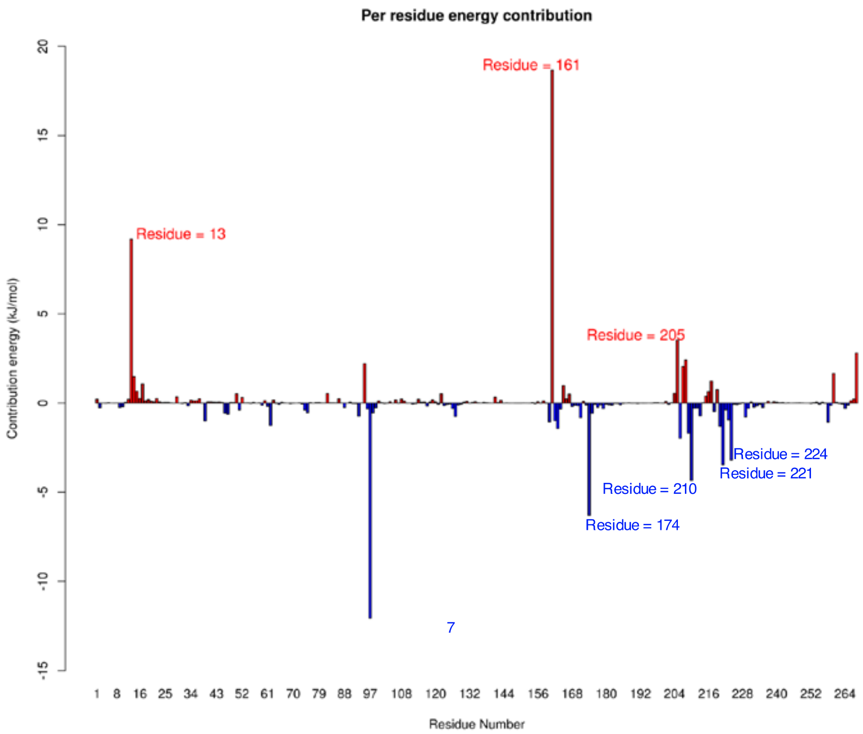

Figure 11L: Energetic contribution of the of *Tb*PTR1 residues in binding. The energies of the *Tb*PTR1-RUBi012 complex are given as kilojoules per mole. A) Δ_E_MM, Δ_G_polar, Δ_G_nonpolar and Δ_G_binding as a function of time B) Binding energy contribution energy for each residue.

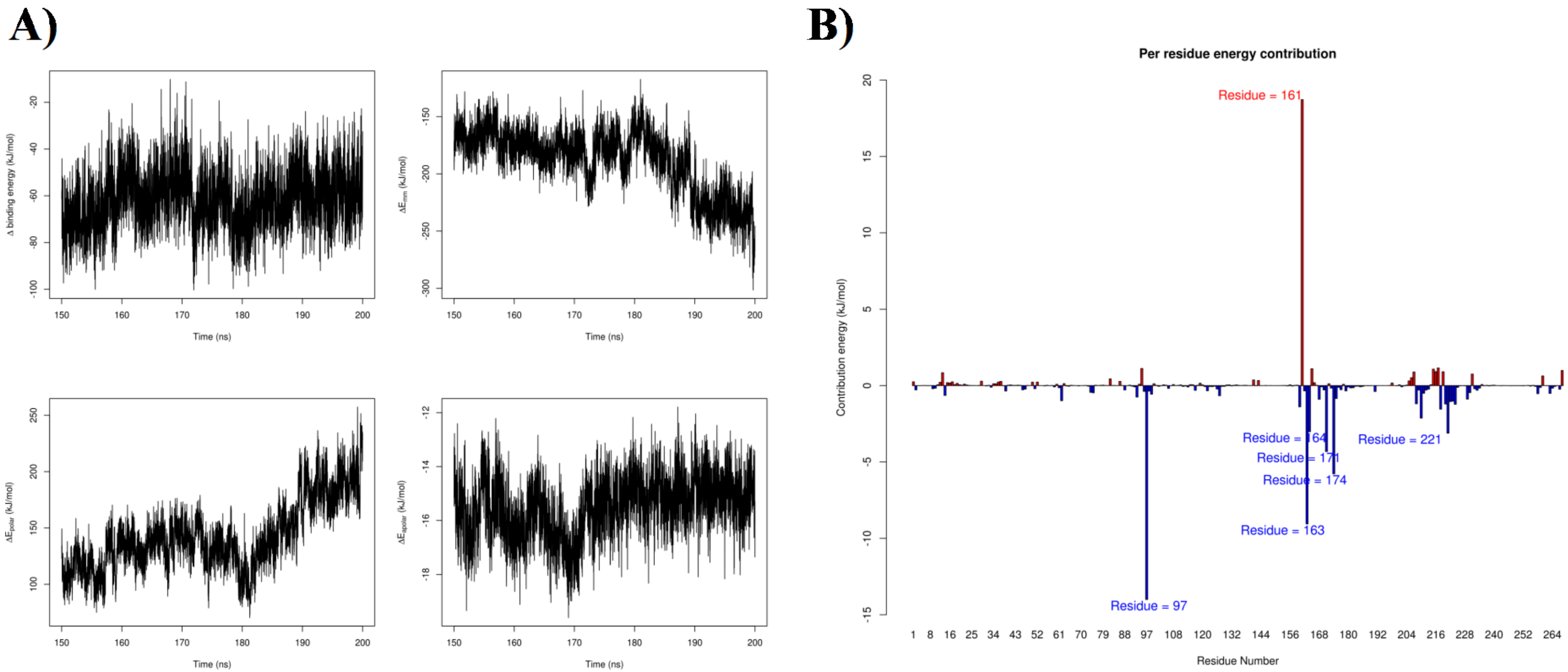

Figure 11M: Energetic contribution of the of *Tb*PTR1 residues in binding. The energies of the *Tb*PTR1-RUBi013 complex are given as kilojoules per mole. A) Δ_E_MM, Δ_G_polar, Δ_G_nonpolar and Δ_G_binding as a function of time B) Binding energy contribution energy for each residue.

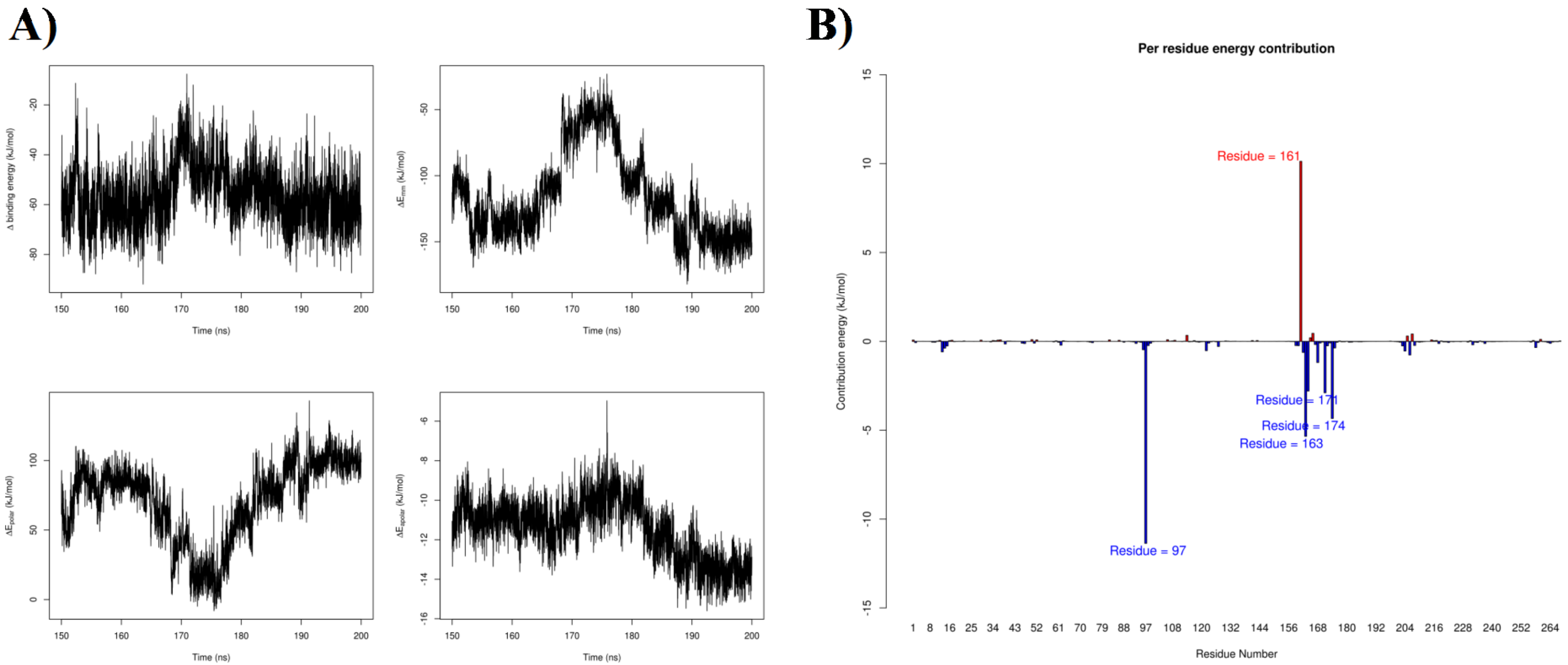

Figure 11N: Energetic contribution of the of *Tb*PTR1 residues in binding. The energies of the *Tb*PTR1-RUBi014 complex are given as kilojoules per mole. A) Δ_E_MM, Δ_G_polar, Δ_G_nonpolar and Δ_G_binding as a function of time B) Binding energy contribution energy for each residue.

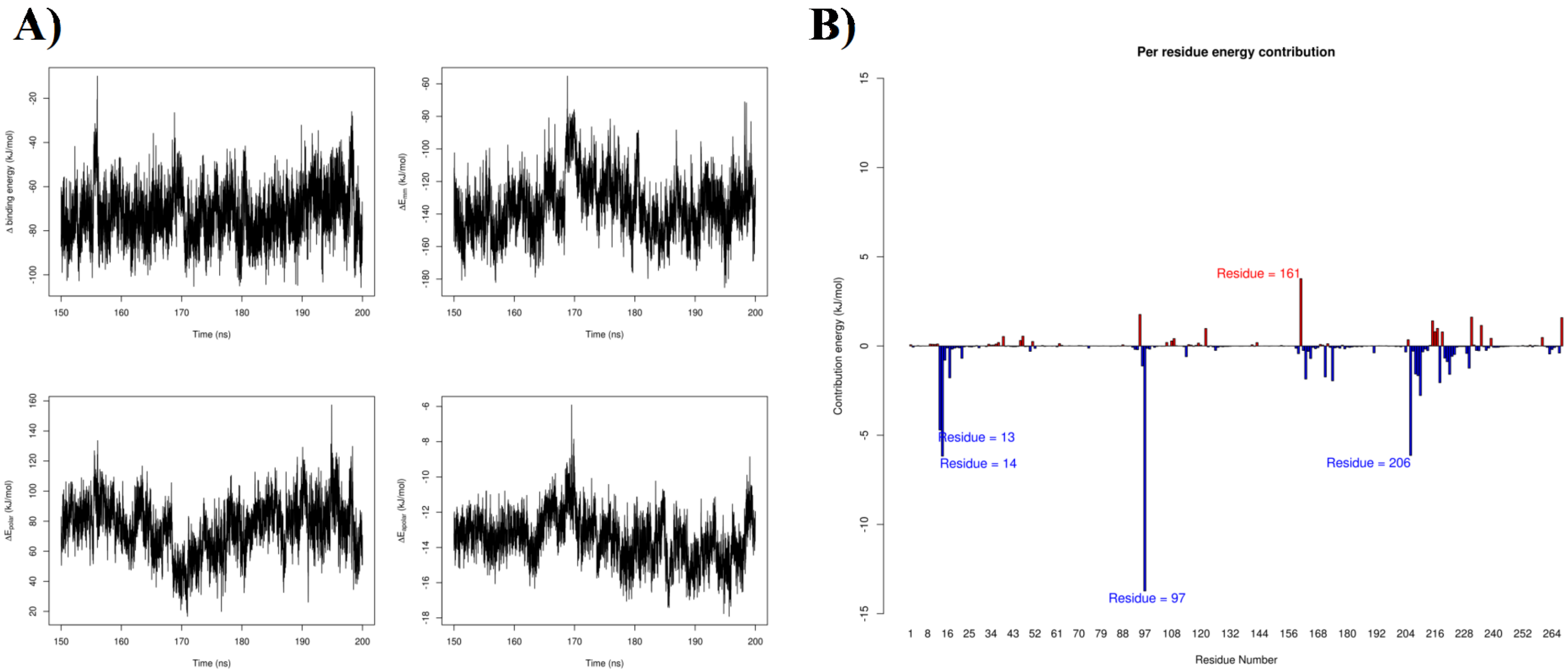

Figure 11O: Energetic contribution of the of *Tb*PTR1 residues in binding. The energies of the *Tb*PTR1-RUBi015 complex are given as kilojoules per mole. A) Δ_E_MM, Δ_G_polar, Δ_G_nonpolar and Δ_G_binding as a function of time B) Binding energy contribution energy for each residue.

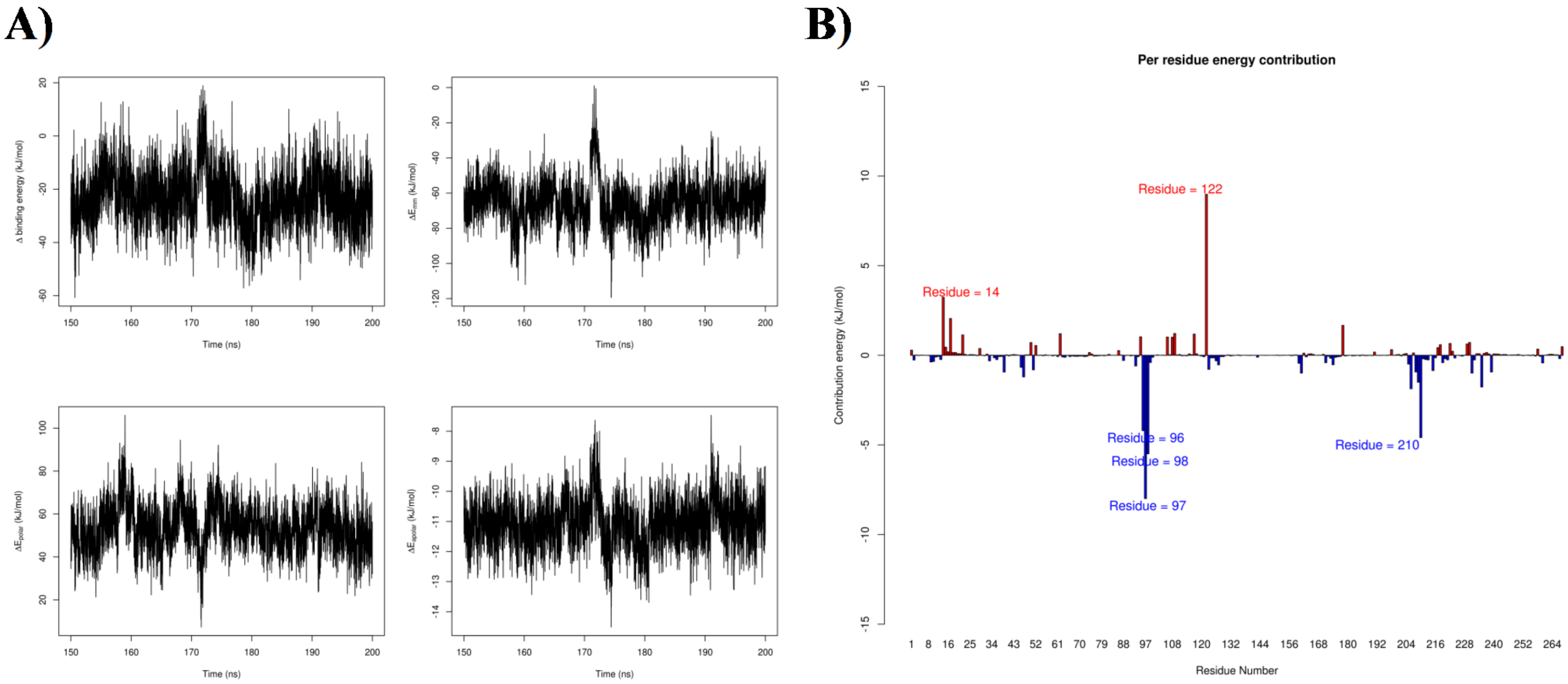

Figure 11P: Energetic contribution of the of *Tb*PTR1 residues in binding. The energies of the *Tb*PTR1-RUBi016 complex are given as kilojoules per mole. A) Δ_E_MM, Δ_G_polar, Δ_G_nonpolar and Δ_G_binding as a function of time B) Binding energy contribution energy for each residue.

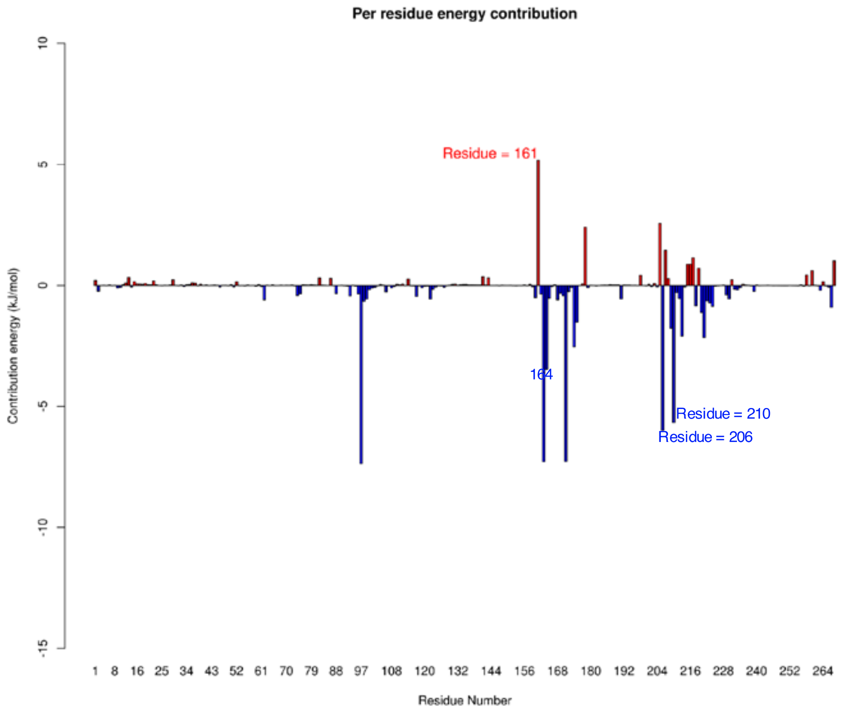

Figure 11R: Energetic contribution of the of *Tb*PTR1 residues in binding. The energies of the *Tb*PTR1-RUBi017 complex are given as kilojoules per mole. A) Δ_E_MM, Δ_G_polar, Δ_G_nonpolar and Δ_G_binding as a function of time B) Binding energy contribution energy for each residue.

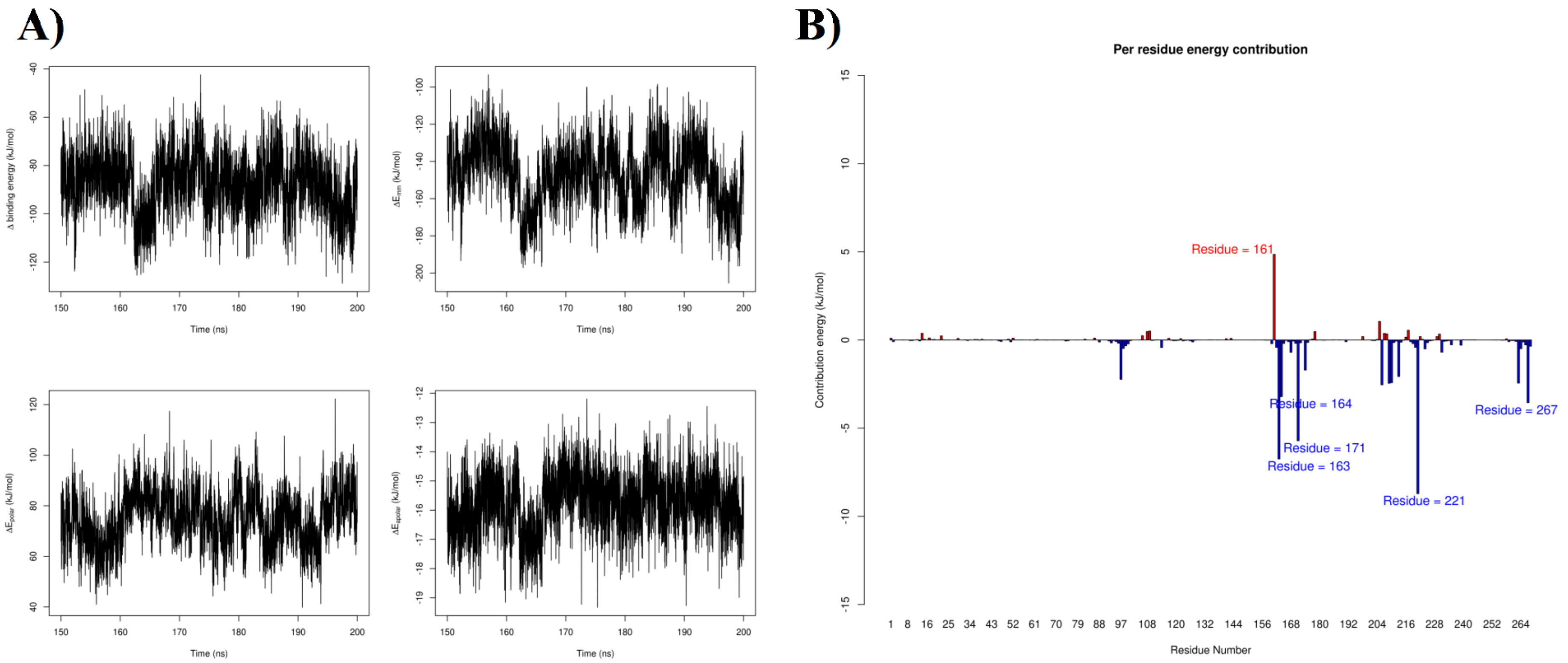

Figure 11S: Energetic contribution of the of *Tb*PTR1 residues in binding. The energies of the *Tb*PTR1-RUBi018 complex are given as kilojoules per mole. A) Δ_E_MM, Δ_G_polar, Δ_G_nonpolar and Δ_G_binding as a function of time B) Binding energy contribution energy for each residue.

Supplementary Figure S12: Cytotoxicity IC_{50}s for RUBi004 and RUBi014

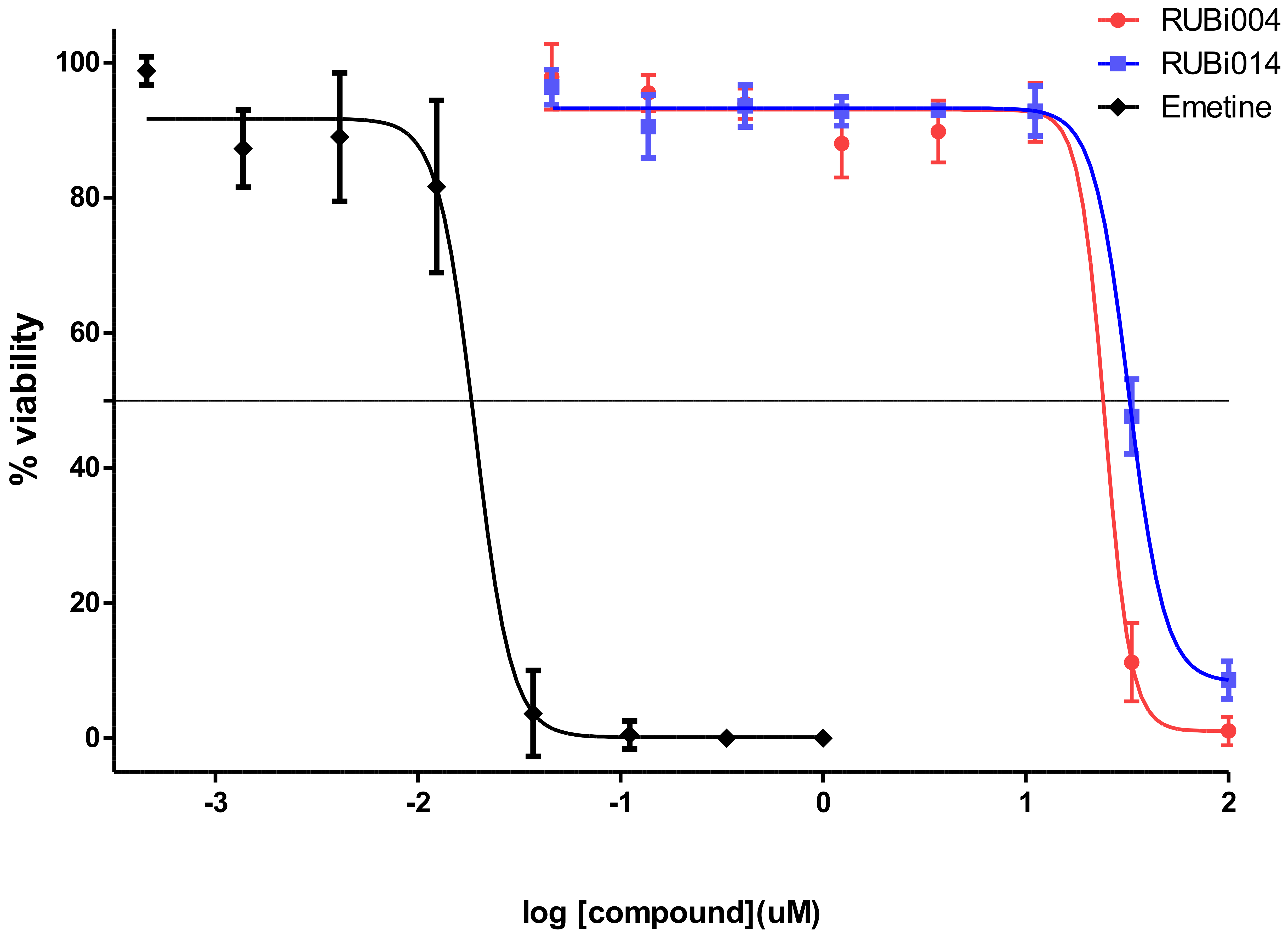

Supplementary Figure S13: Per residue Root mean square fluctuations (RMSF), average Betweenness Centrality (BC) and average shortest path (L) of *Tb*PTR1 residues.

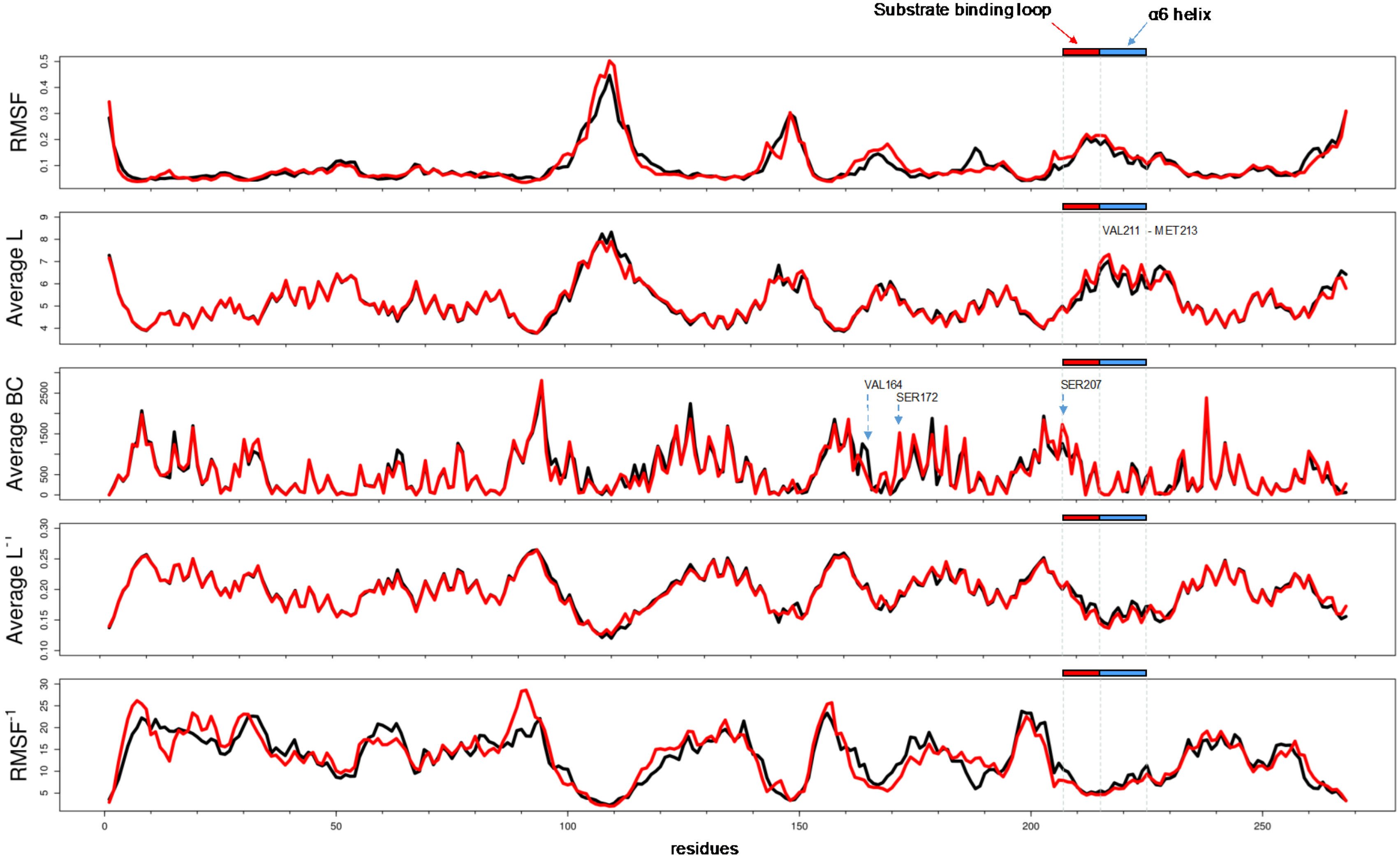

Figure 13A: The RMSF, average BC, average L, RMSF^{-1}, and average L^{-1} of the backbone protein in the protein-ligand complex shown as a function of time. The apo protein was colored black and the *Tb*PTR1-RUBi004 complex red.

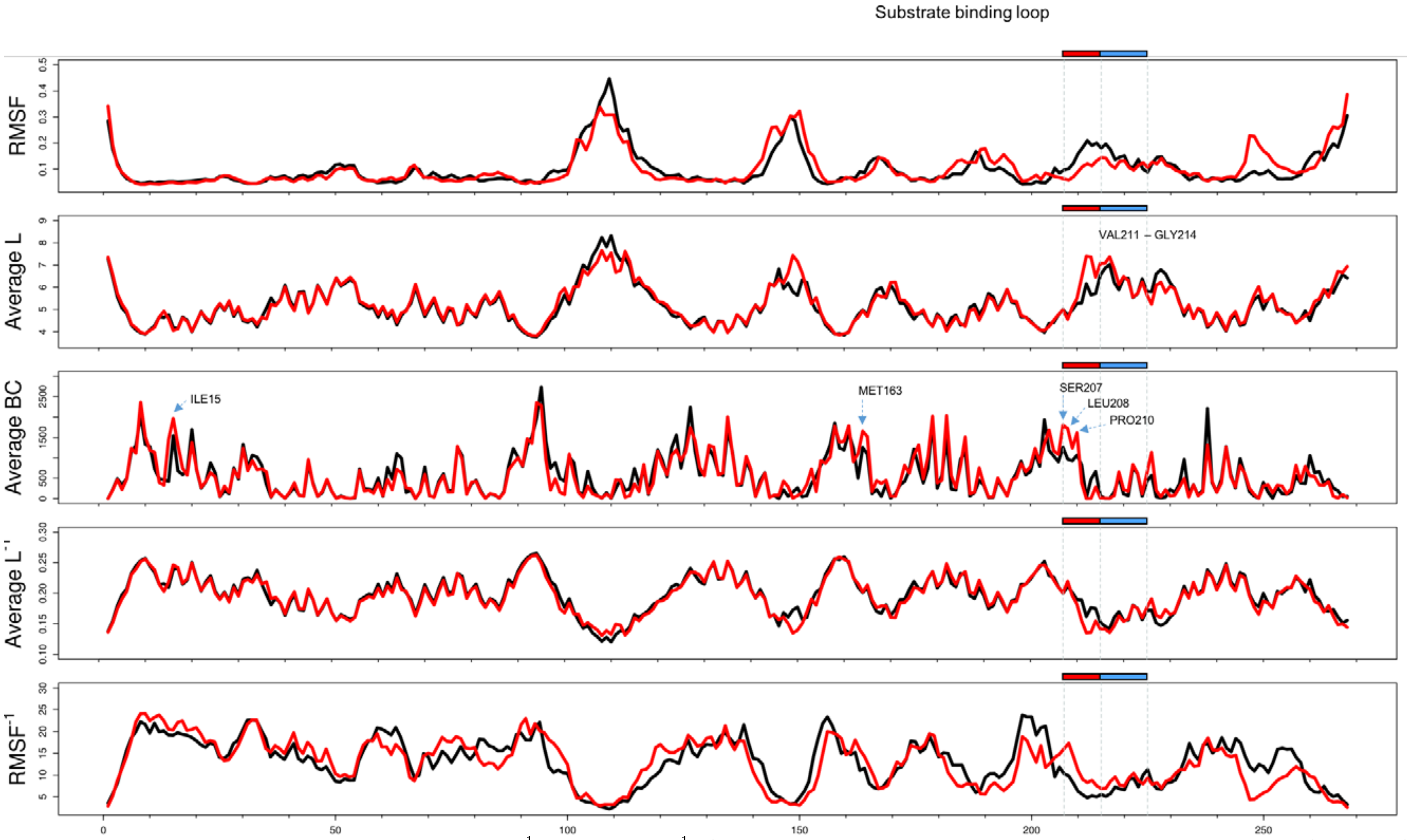

Figure 13B: The RMSF, average BC, average L, RMSF^{-1}, and average L^{-1} of the backbone protein in the protein-ligand complex shown as a function of time. The apo protein was colored black and the *Tb*PTR1-RUBi007 complex red.

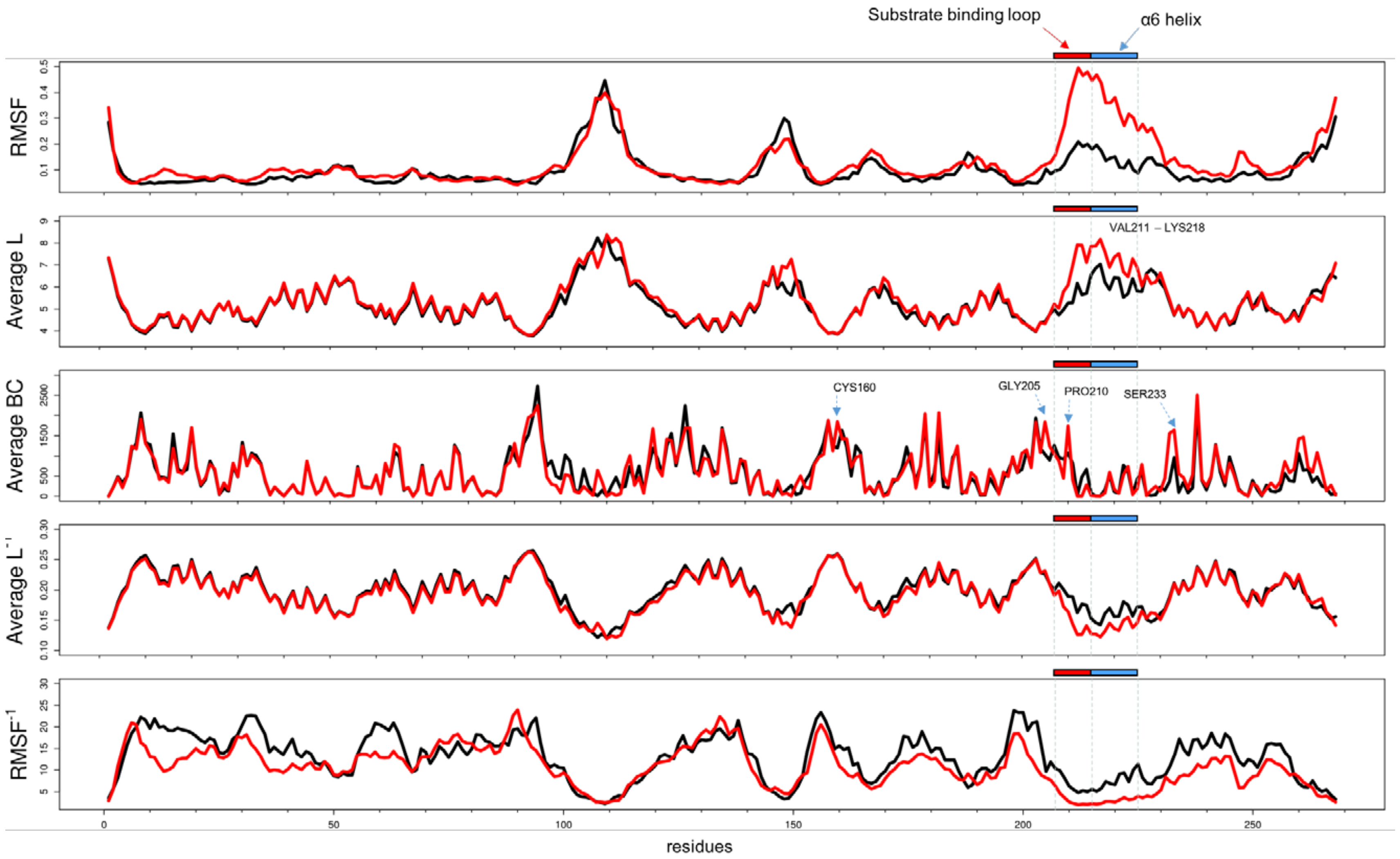

Figure 13C: The RMSF, average BC, average L, RMSF^{-1}, and average L^{-1} of the backbone protein in the protein-ligand complex shown as a function of time. The apo protein was colored black and the *Tb*PTR1-RUBi014 complex red.

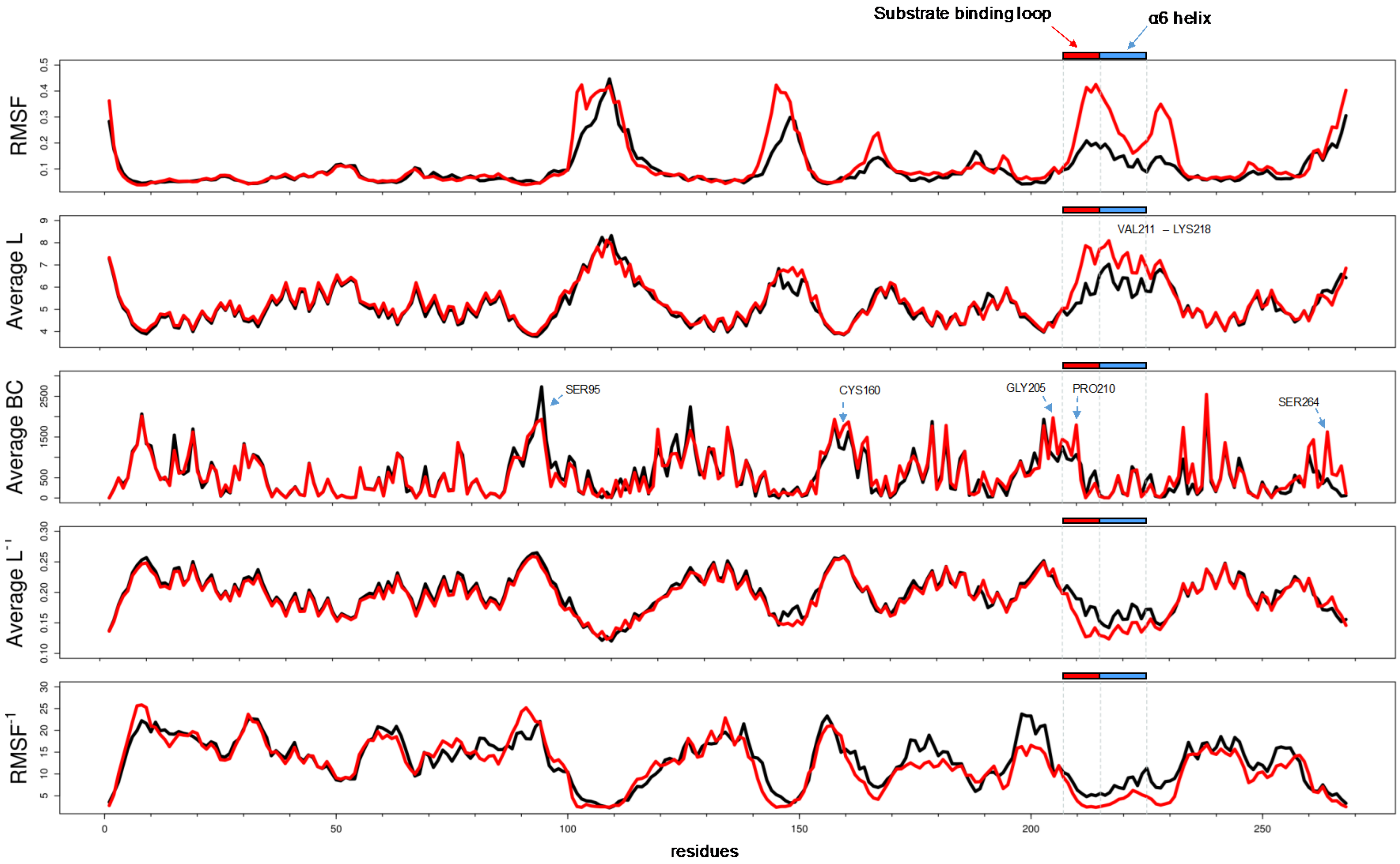

Figure 13D: The RMSF, average BC, average L, $RMSF^{-1}$, and average L^{-1} of the backbone protein in the protein-ligand complex shown as a function of time. The apo protein was colored black and the *Tb*PTR1-RUBi016 complex red.

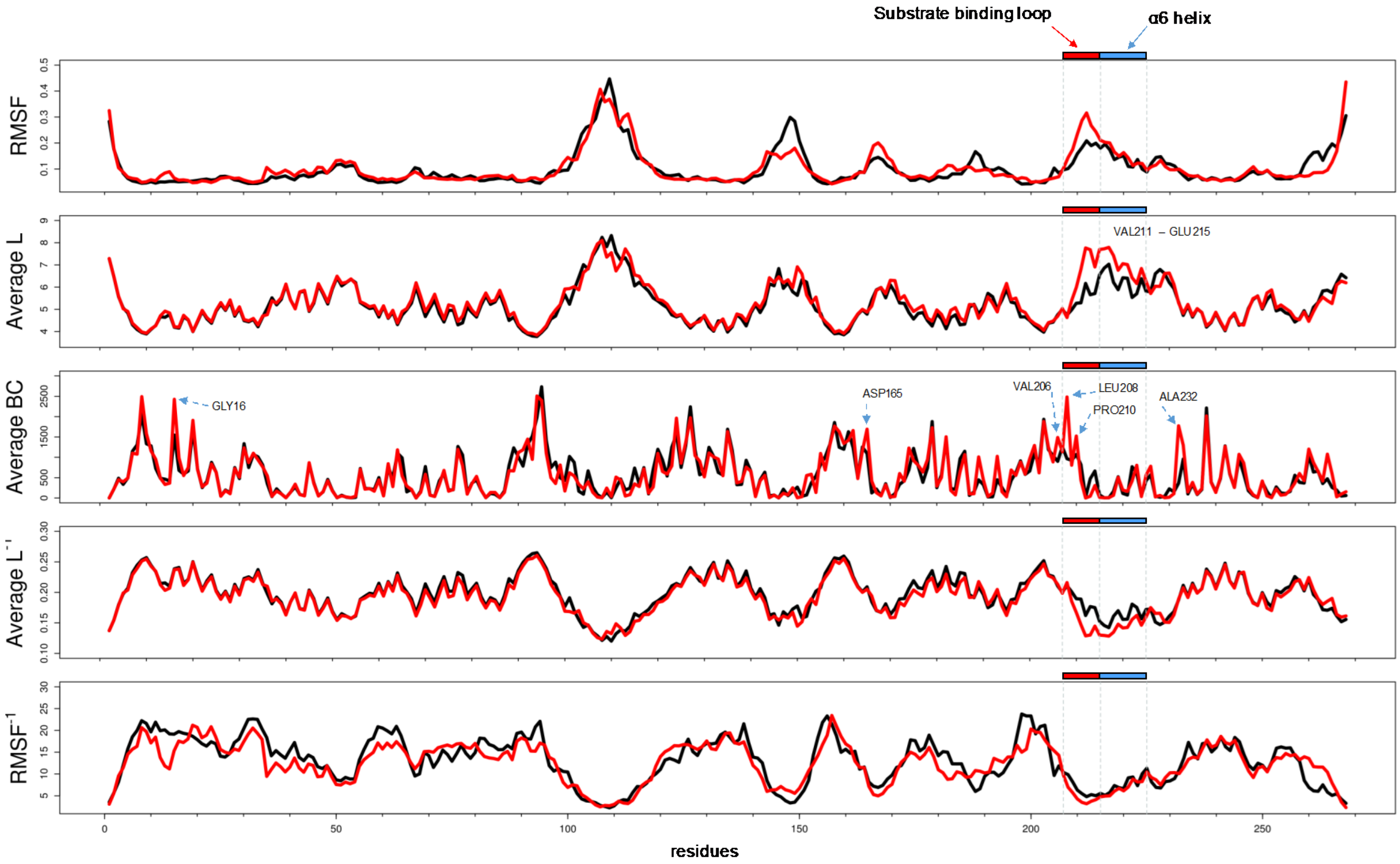

Figure 13E: The RMSF, average BC, average L, RMSF^{-1}, and average L^{-1} of the backbone protein in the protein-ligand complex shown as a function of time. The apo protein was colored black and the *Tb*PTR1-RUBi018 complex red.

Supplementary Figure S14: 2D interaction diagram around selected residues

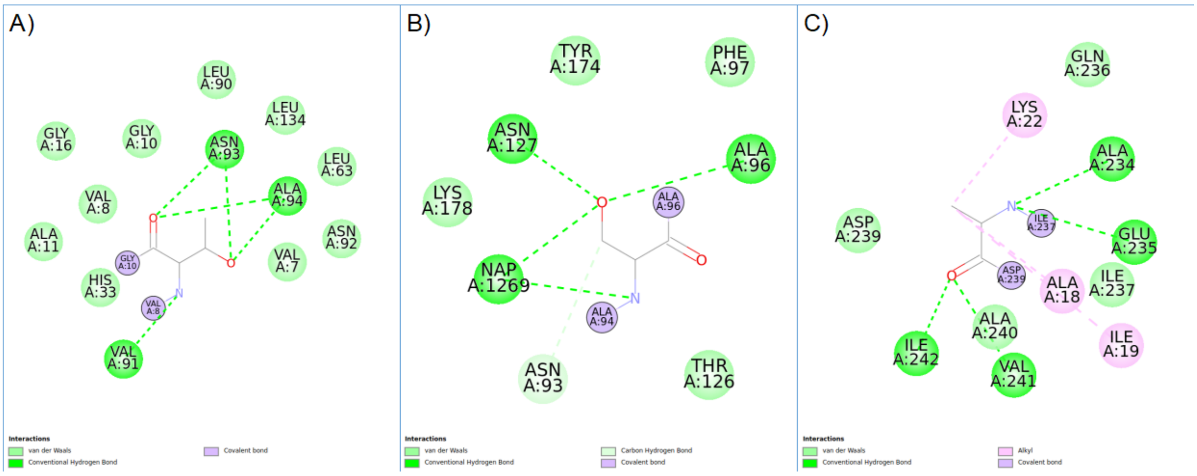

Figure 1: 2D interaction network around residues in *Tb*PTR1 (PDB:2X9N). A) THR9, B) SER95, and C) ALA238

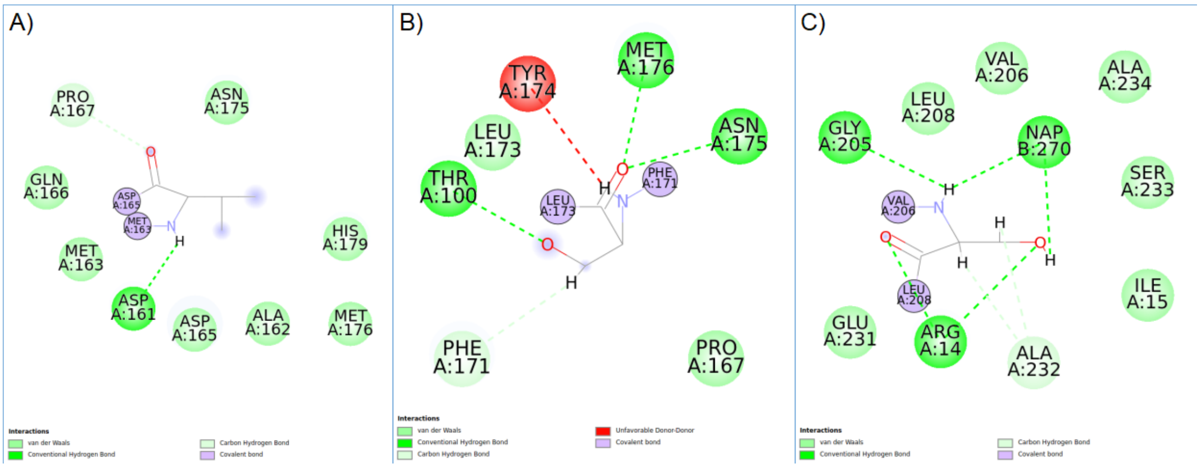

Figure 2: 2D interaction network around residues RUBi004-*Tb*PTR1 complex (PDB:2X9N). A) VAL164, B) SER172, and C) SER207

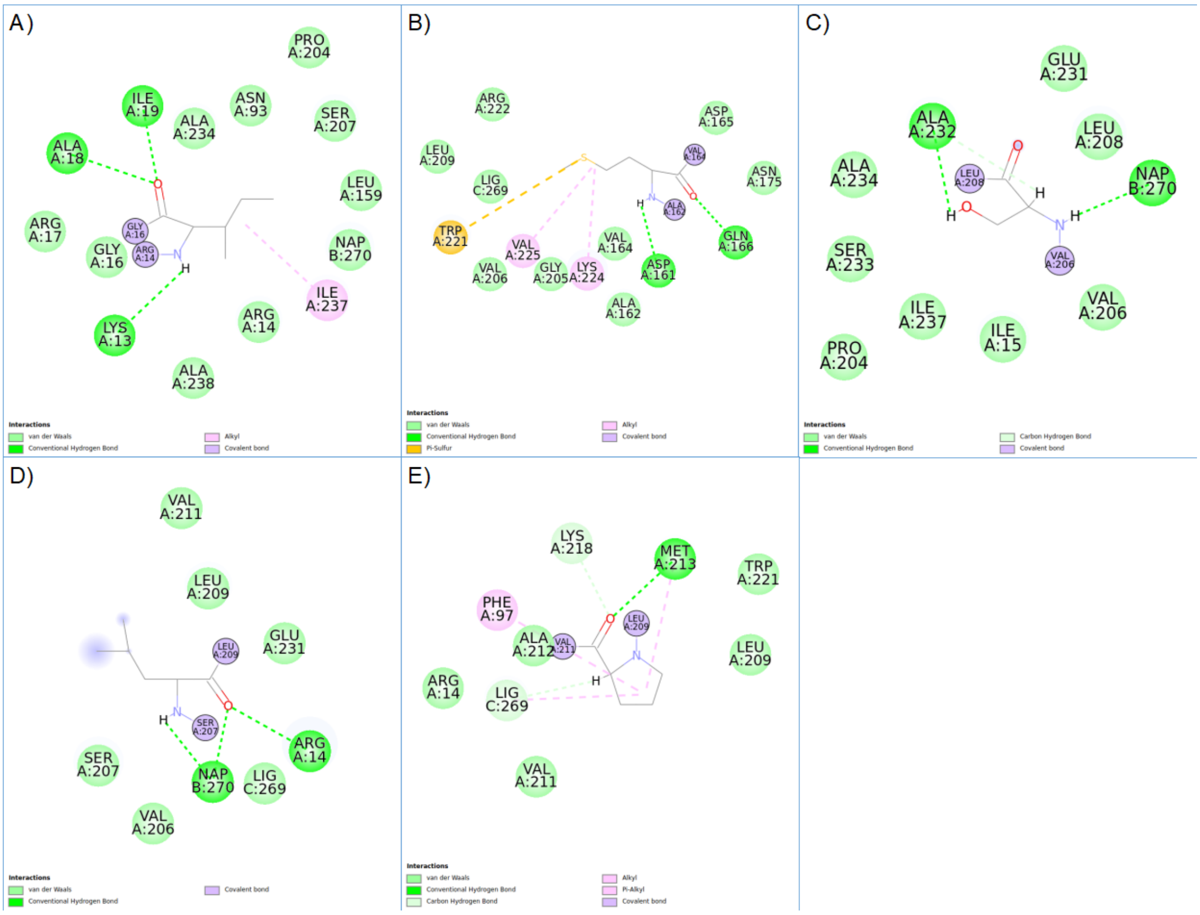

Figure 3: 2D interaction network around residues RUBi007-*Tb*PTR1 complex (PDB:2X9N). A) ILE15, B) MET163, C) SER207, D) LEU208, and E) PRO210

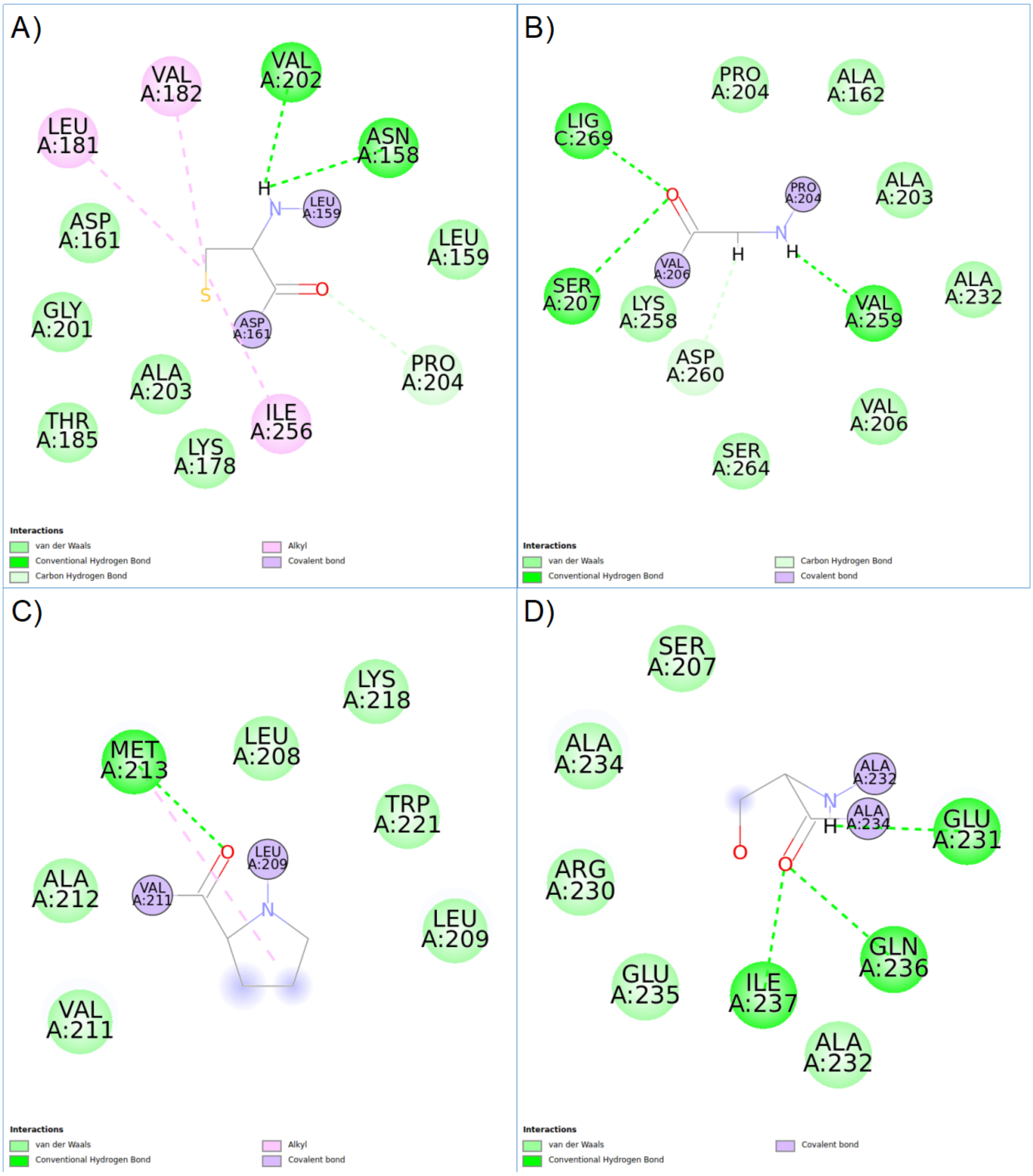

Figure 4: 2D interaction network around residues RUBi014-*Tb*PTR1 complex (PDB:2X9N). A) CYS160, B) GLY205, C) PRO210, and D) SER233

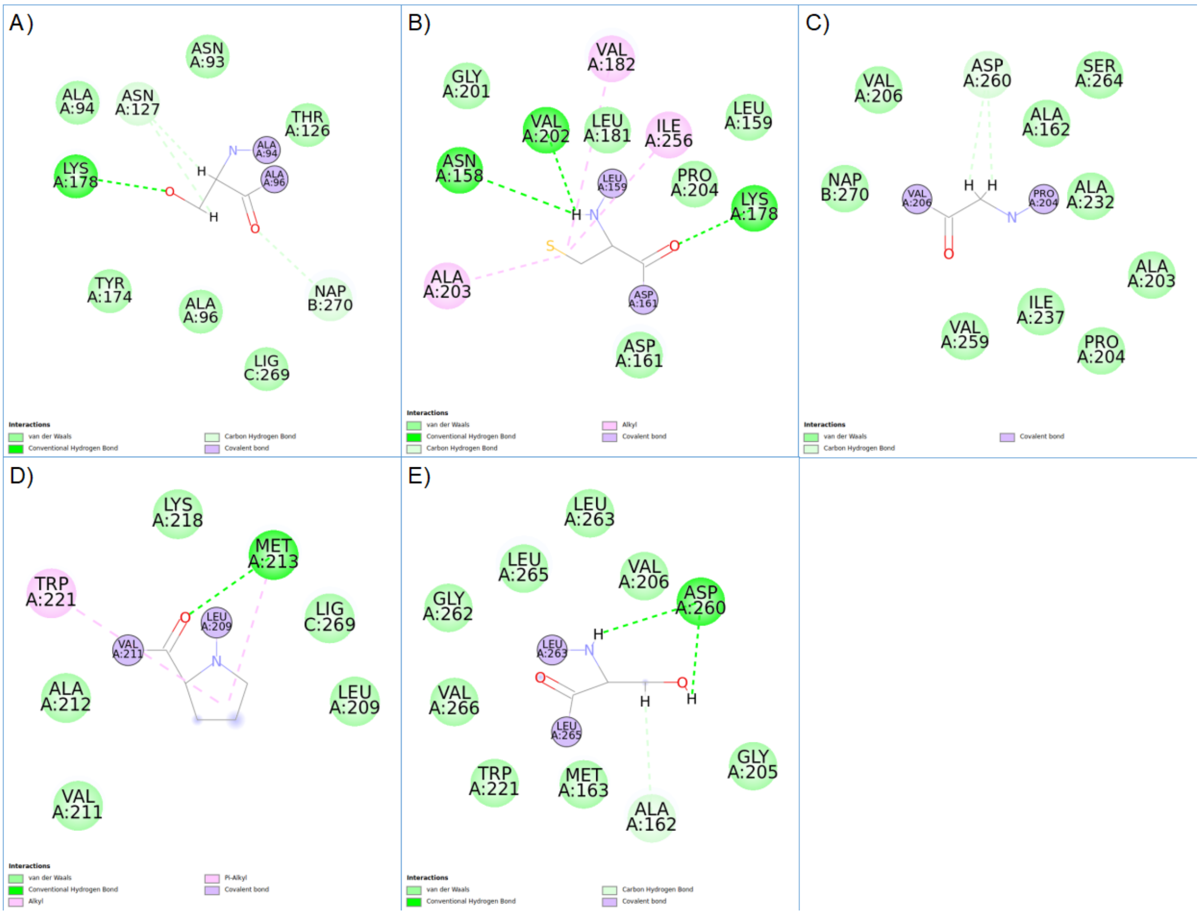

Figure 5: 2D interaction network around residues RUBi016-*Tb*PTR1 complex (PDB:2X9N). A) SER95, B) CYS160, C) GLY205, D) PRO210, and E) SER264

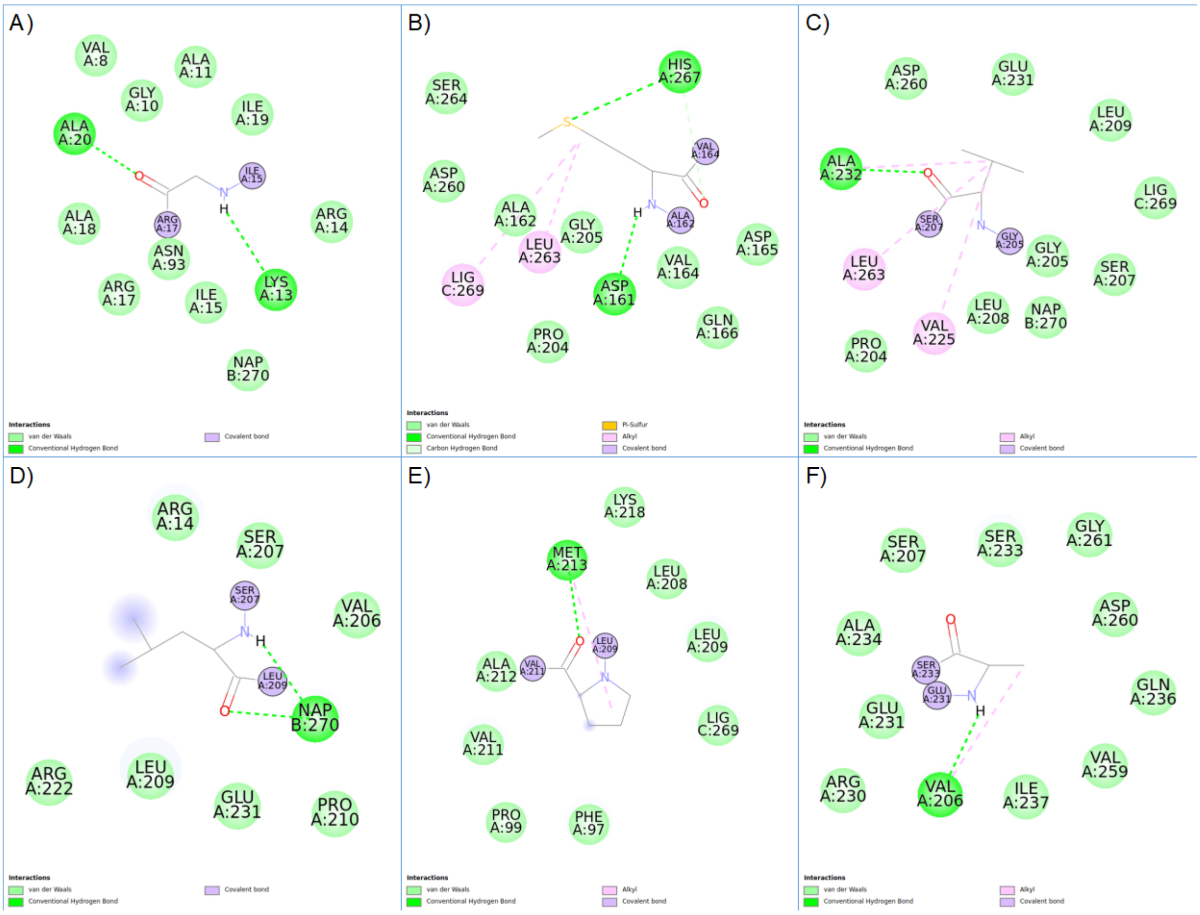

Figure 6: 2D interaction network around residues RUBi018-*Tb*PTR1 complex (PDB:2X9N). A) GLY16, B) ASP165, C) VAL206, D) LEU208, E) PRO210, and F) ALA232

Supplementary Figure S15: Validation of *T. brucei* and *T. cruzi* PTR1 homology models using z-DOPE score and residue score using ProSA. The structural validation of *Tb*PTR1 is shown in A) and B) while that of *Tc*PTR1 in C) and D). Both models show overall reliable structural conformations.

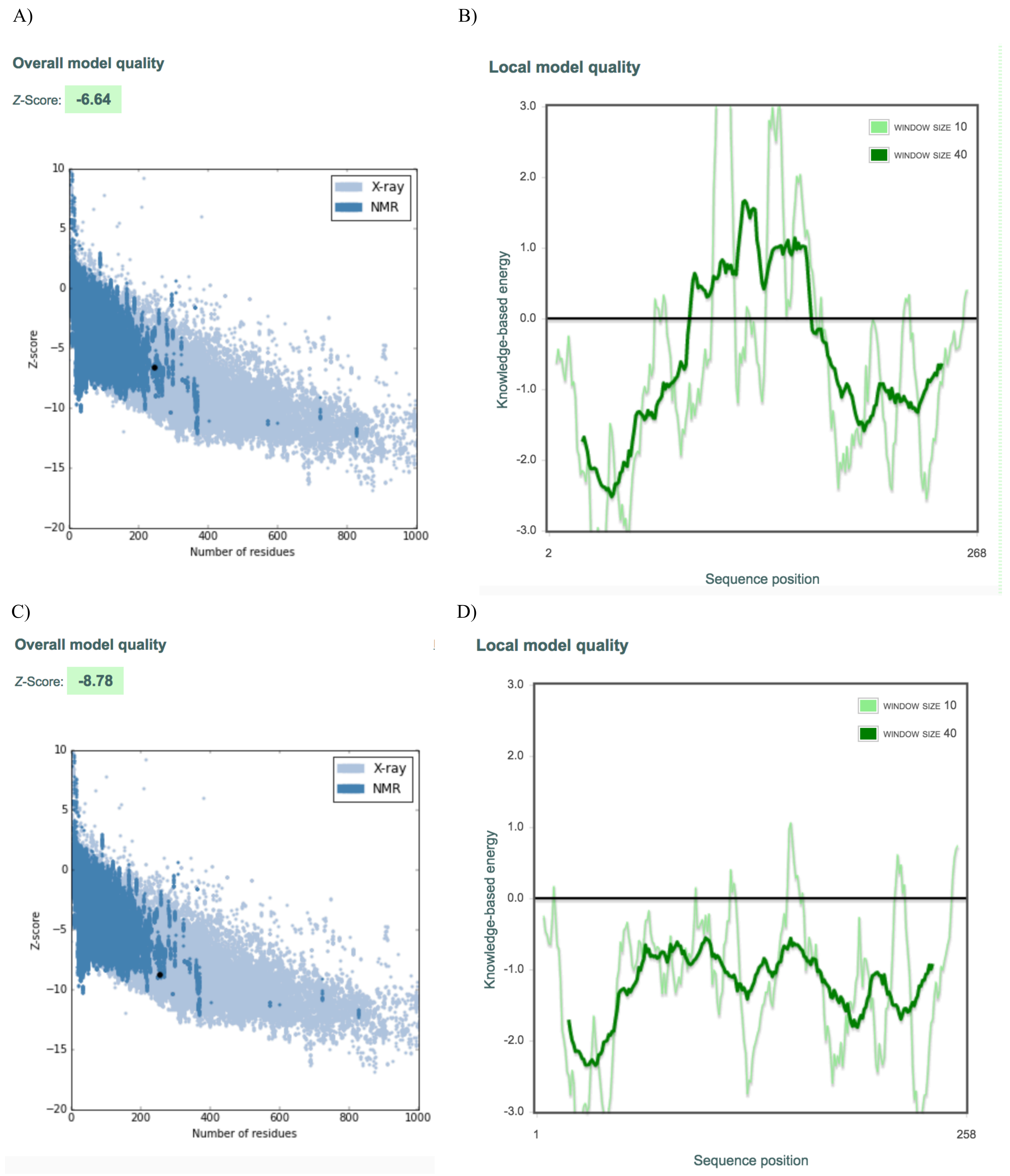

Table S1: Binding modes of top *Tb*PTR1 docked compounds

Compound	Binding mode	Interacting residues	Autodock Vina (Kcal/mol)	Xscore (Kcal/mol)
RUBi001	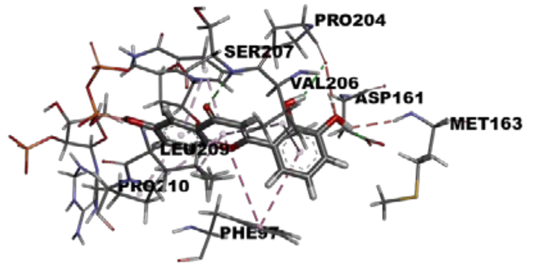	PHE97, ASP161, MET163, PRO204, VAL206, SER207, LEU209, PRO210	-10.1	-8.3
RUBi002	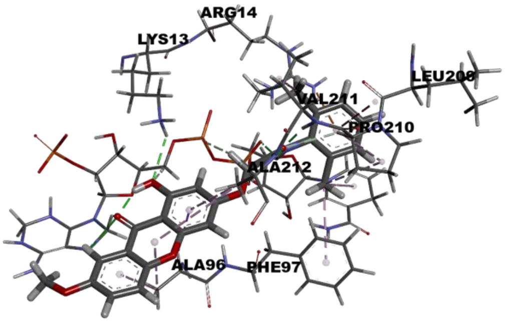	LYS13, ARG14, ALA96, PHE97, LEU208, PRO210, VAL211, ALA212	-10.2	-8.8
RUBi003	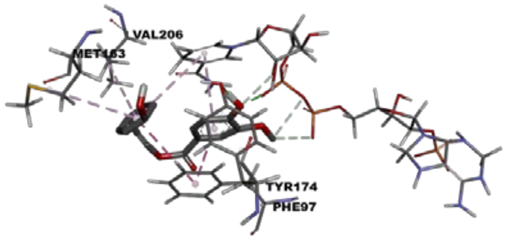	PHE97, MET163, TYR174, VAL206	-9.1	-8.2
**RUBi004**	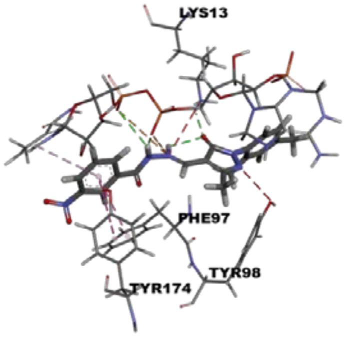	LYS13, PHE97, TYR98, TYR174	-10.3	-8.8
RUBi005	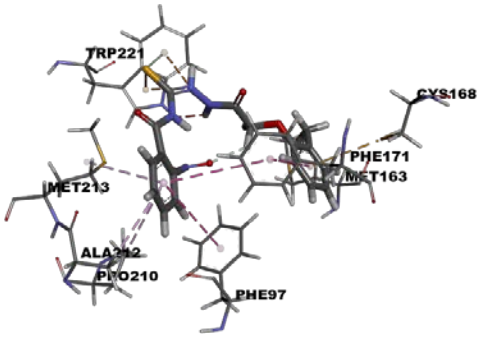	PHE97, MET163, CYS168, PHE171, PRO210, ALA212, MET213, TRP221	-9.0	-8.6

RUBi006	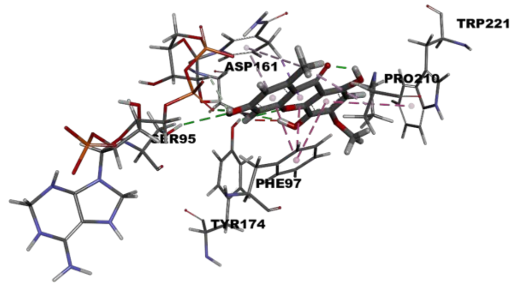	SER95,<br>PHE97,<br>ASP161,<br>TYR174,<br>PRO210,<br>TRP221	-10.2	-8.1
**RUBi007**	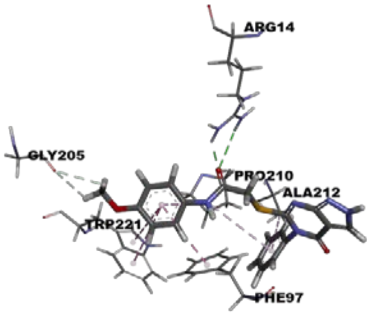	ARG14,<br>PHE97,<br>GLY205,<br>PRO210,<br>ALA212,<br>TRP221	-9.6	-8.0
RUBi008	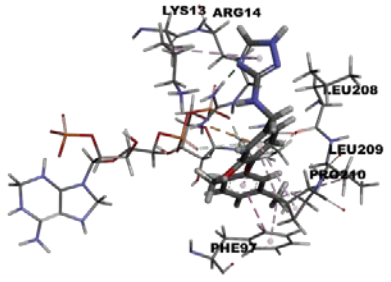	LYS13,<br><br>ARG14,<br>PHE97,<br>LEU208,<br>LEU209,<br>PRO210	-8.5	-7.8
RUBi009	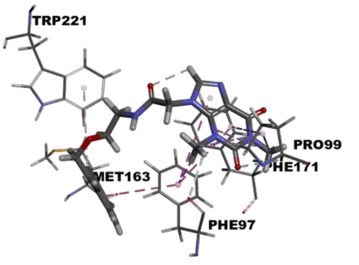	PHE97,<br>PRO99,<br>MET163,<br>PHE171,<br>TRP221	-8.9	-7.7
RUBi010	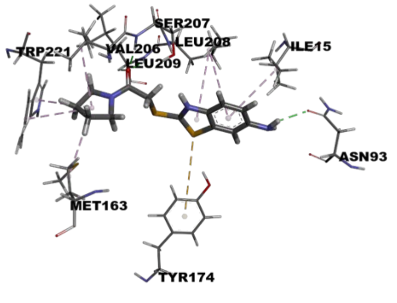	ILE15,<br>ASN93,<br>MET163,<br>TYR174,<br>VAL206,<br>SER207,<br>LEU208,<br>LEU209,<br>TRP221	-6.9	-7.9

RUBi011	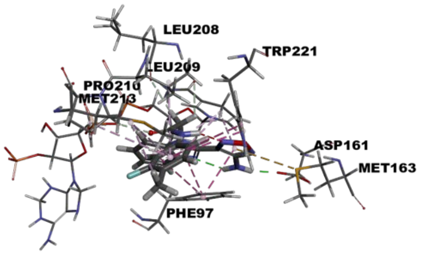	PHE97, ASP161, MET163, LEU208, LEU209, PRO210, MET213, TRP221	-9.7	-8.8
RUBi012	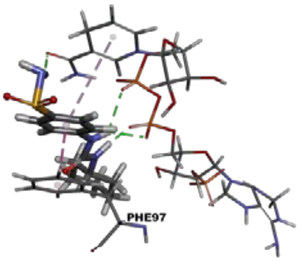	PHE97	-9.1	-8.2
RUBi013	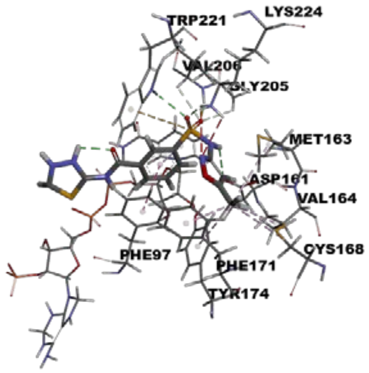	PHE97, ASP161, MET163, VAL164, CYS168, PHE171, TYR174, GLY205, VAL206, TRP221, LYS224	-8.7	-8.4
**RUBi014**	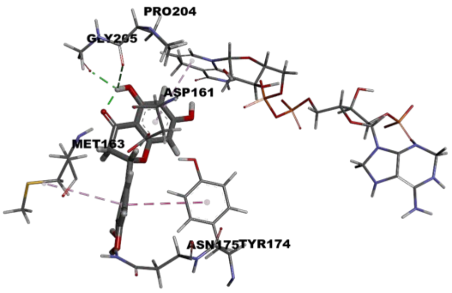	ASP161, MET163, TYR174, ASN175, PRO204, GLY205	-9.7	-8.2
RUBi015	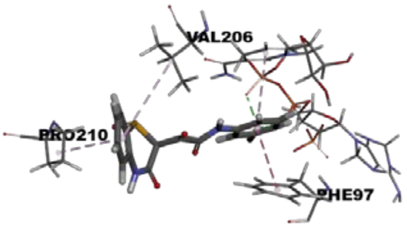	PHE97, VAL206, PRO210	-9.1	-7.9

**RUBi016**	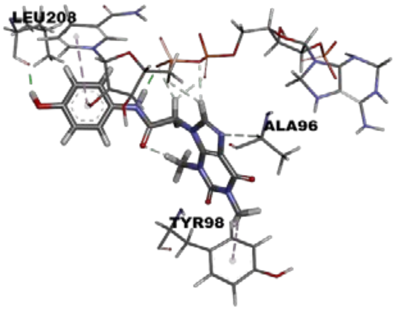	ALA96,<br>TYR98,<br>LEU208	-8.9	-7.53
**RUBi017**	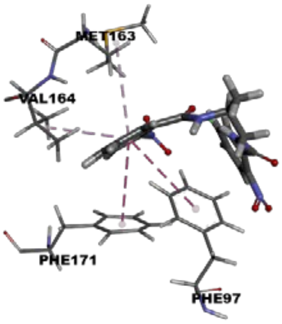	PHE97,<br>MET163,<br>VAL164,<br>PHE171	-8.8	-8.7
**RUBi018**	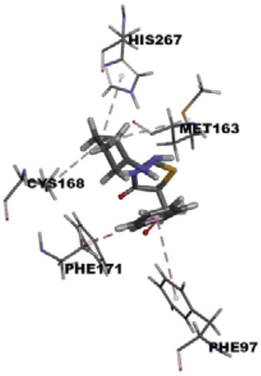	PHE97,<br>MET163,<br>CYS168,<br>PHE171,<br>HIS267	-8.4	-8.8

Table S2. The IUPAC names of the top *Tb*PTR1 docking compounds

Compound Information		
**Code Name**	**IUPAC Name**	**Database ID**
RUBi001	2-(2,3-dihydroxyphenyl)-6-hydroxychromen-4-one	ZINC00057846
RUBi002	N-(2,6-dimethylphenyl)-2-(1-hydroxy-7-methoxy-9-oxo-xanthen-3-yl)oxy-acetamide	ZINC08992677
RUBi003	2-(4-Hydroxyphenyl)ethyl 4-hydroxy-3-methoxybenzoate	SANC00368
RUBi004	N'-{[1-(2,4-dimethylphenyl)-3-methyl-5-oxo-1,5-dihydro-4H-pyrazol-4-ylidene]methyl}-3-nitrobenzohydrazide	ZINC00809143
RUBi005	2-nitro-N-({2-[(2-methylphenoxy)acetyl]hydrazino}carbothioyl)benzamide	ZINC02690799
RUBi006	1,4,6-Trihydroxy-3-methoxy-8-methyl-9H-xanthen-9-one	SANC00470
RUBi007	N-(4-methoxyphenyl)-2-[(4-oxo-5-phenyl-4,5-dihydro-1H-pyrazolo[3,4-d]pyrimidin-6-yl)sulfanyl]acetamide	ZINC00630525
RUBi008	[3-Methoxy-4-(3-methyl-benzyloxy)-benzyl]-(1H-[1,2,4]triazol-3-yl)-amine	ZINC06556964
RUBi009	N-{2-[(2-chlorobenzyl)oxy]ethyl}-2-(1,3-dimethyl-2,6-dioxo-1,2,3,6-tetrahydro-9H-purin-9-yl)acetamide	ZINC02177983
RUBi010	2-{[2-oxo-2-(1-pyrrolidinyl)ethyl]sulfanyl}-1,3-benzothiazol-6-ylamine	ZINC00359797
RUBi011	2-[2-(4-amino-1,2,5-oxadiazol-3-yl)-1H-benzimidazol-1-yl]-N-(3-fluoro-4-methylphenyl)acetamide	ZINC00677623
RUBi012	1-cyclohexyl-3-(4-sulfamoylphenyl)urea	ZINC01003765
RUBi013	3-{[(5-methyl-3-isoxazolyl)amino]sulfonyl}-N-(1,3,4-thiadiazol-2-yl)benzamide	ZINC02184332
RUBi014	2-(3,4-dihydroxyphenyl)-5,7-dihydroxy-3,4-dihydro-2H-1-benzopyran-4-one	ZINC0058117 / SANC00320
RUBi015	N-(3-hydroxyphenyl)-2-(1-oxido-3-oxo-3,4-dihydro-2H-1,4-benzothiazin-2-yl)-2-oxoacetamide	ZINC04671320
RUBi016	2-(1,3-dimethyl-2,6-dioxo-1,2,3,6-tetrahydro-9H-purin-9-yl)-N-(4-hydroxyphenyl)acetamide	ZINC00612219
RUBi017	2-nitro-N-{2-[(2-nitrophenyl)formamido]propyl}benzamide	ZINC04523829
RUBi018	2-[2-(cyclopentylidenehydrazono)-4-hydroxy-2,5-dihydro-1,3-thiazol-5-yl]-N-phenylacetamide	ZINC04313814

Table S3: The Pearson correlation coefficients for RMSF vs Average L, Average BC vs 1/(Average L), and Average BC vs 1/(RMSF).

	**Pearson correlation coefficient**		
**System**	**RMSF vs Average L**	**Average BC vs 1/Average L**	**Average BC vs 1/RMSF**
Apo protein	0.716	0.742	0.434
RUBi004	0.743	0.664	0.424
RUBi007	0.714	0.682	0.505
RUBi014	0.714	0.632	0.326
RUBi016	0.761	0.636	0.383
RUBi018	0.808	0.646	0.443

Table S4: Protein structures used in molecular docking

Enzyme	ORGANISM	UNIPROT	PDB	Resolution (Angstroms)	Chains	Residues	RMS (Protein vs 2X9N)
Pteridine reductase 1	*Trypanosoma brucei brucei*	O76290	2X9N	1.15	A,B,C,D	288	0
Pteridine reductase 1	*Trypanosoma cruzi*	O44029	-	Homology model based on 1MXH (2.2)	A,B,C,D	276	0.5
Pteridine reductase 1	*Leishmania major*	Q01782	1E92	2.2	A,B,C,D	288	0.5
Dehydrogenase/reductase SDR family member 4	*Homo sapiens*	Q9BTZ2	3O4R	1.7	A,B,C,D	261	1.6
Dihydrofolate reductase	*Trypanosoma brucei brucei*	Q27783	3QFX	2.2	A, B	241	13.5

Table S5: Molecular Docking Parameters for Autodock Vina

**Parameter**	***T brucei*** PTR1 (PDB: 2X9N)	***T cruzi*** PTR1 homology model	***L major*** PTR1 (PDB: 1E92)	***H. Sapiens*** DHRS4 (PDB: 3O4R)	***T brucei*** (PDB: 3QFX)
Protein center X	30.859	15.330	11.600	29.790	-19.789
Protein center Y	-0.064	32.050	45.880	2.200	23.569
Protein center Z	92.956	-0.021	69.630	17.480	8.146
Box size X	126	126	126	126	126
Box size Y	126	126	126	126	126
Box size Z	126	126	126	126	126
Energy range	4	4	4	4	4
Exhaustiveness	120	120	120	120	120
CPU	24	24	24	24	24
